# Appraising the relevance of DNA copy number loss and gain in prostate cancer using whole genome DNA sequence data

**DOI:** 10.1371/journal.pgen.1007001

**Published:** 2017-09-25

**Authors:** Niedzica Camacho, Peter Van Loo, Sandra Edwards, Jonathan D. Kay, Lucy Matthews, Kerstin Haase, Jeremy Clark, Nening Dennis, Sarah Thomas, Barbara Kremeyer, Jorge Zamora, Adam P. Butler, Gunes Gundem, Sue Merson, Hayley Luxton, Steve Hawkins, Mohammed Ghori, Luke Marsden, Adam Lambert, Katalin Karaszi, Gill Pelvender, Charlie E. Massie, Zsofia Kote-Jarai, Keiran Raine, David Jones, William J. Howat, Steven Hazell, Naomi Livni, Cyril Fisher, Christopher Ogden, Pardeep Kumar, Alan Thompson, David Nicol, Erik Mayer, Tim Dudderidge, Yongwei Yu, Hongwei Zhang, Nimish C. Shah, Vincent J. Gnanapragasam, The CRUK-ICGC Prostate Group, William Isaacs, Tapio Visakorpi, Freddie Hamdy, Dan Berney, Clare Verrill, Anne Y. Warren, David C. Wedge, Andrew G. Lynch, Christopher S. Foster, Yong Jie Lu, G. Steven Bova, Hayley C. Whitaker, Ultan McDermott, David E. Neal, Rosalind Eeles, Colin S. Cooper, Daniel S. Brewer

**Affiliations:** 1 Division of Genetics and Epidemiology, The Institute Of Cancer Research, London, United Kingdom; 2 Human Oncology and Pathogenesis Program, Memorial Sloan Kettering Cancer Center, New York, New York, United States of America; 3 Marie-Josée and Henry R. Kravis Center for Molecular Oncology, Memorial Sloan Kettering Cancer Center, New York, New York, United States of America; 4 Cancer Genomics Laboratory, The Francis Crick Institute, London, United Kingdom; 5 Department of Human Genetics, University of Leuven, Leuven, Belgium; 6 Uro-Oncology Research Group, Cancer Research UK Cambridge Institute, Cambridge, Cambridgeshire, United Kingdom; 7 Molecular Diagnostics and Therapeutics Group, University College London, London, United Kingdom; 8 Norwich Medical School, University of East Anglia, Norwich, Norfolk, United Kingdom; 9 Cancer Genetics Unit, Royal Marsden NHS Foundation Trust, London, United Kingdom; 10 Cancer, Ageing and Somatic Mutation, Wellcome Trust Sanger Institute, Hinxton, Cambridgeshire, United Kingdom; 11 Epidemiology & Biostatistics, Memorial Sloan-Kettering Cancer Center, New York, New York, United States of America; 12 Department of Physiology, University of Oxford, Oxford, Oxfordshire, United Kingdom; 13 Department of Oncology, CRUK/MRC Oxford Institute for Radiation Oncology, Oxford, Oxfordshire, United Kingdom; 14 Nuffield Department of Surgical Sciences, University of Oxford, Oxford, Oxfordshire, United Kingdom; 15 CRUK Cambridge Centre, Early Detection Programme, Urological Malignancies Programme, Hutchison-MRC Research Centre, Cambridge, Cambridgeshire, United Kingdom; 16 Histopathology and *in situ* hybridization Research Group, Cancer Research UK Cambridge Institute, Cambridge, Cambridgeshire, United Kingdom; 17 Department of Epidemiology, Second Military Medical University, Shanghai, China; 18 Department of Histopathology, Cambridge University Hospitals NHS Foundation Trust, Cambridge, Cambridgeshire, United Kingdom; 19 Academic Urology Group, Department of Surgery, University of Cambridge, Cambridge, Cambridgeshire, United Kingdom; 20 School of Medicine, Johns Hopkins University, Baltimore, Maryland, United States of America; 21 Faculty of Medicine and Life Sciences and BioMediTech Institute, University of Tampere and Tampere University Hospital, Tampere, Finland; 22 Centre for Molecular Oncology, Barts Cancer Institute, The Barts and London School of Medicine and Dentistry, Queen Mary University of London, London, United Kingdom; 23 Department of Cellular Pathology and Oxford Biomedical Research Centre, Oxford University Hospitals NHS Trust, Oxford, Oxfordshire, United Kingdom; 24 Oxford Big Data Institute & Oxford Centre for Cancer Gene Research, Wellcome Trust Centre for Human Genetics, Oxford, Oxfordshire, United Kingdom; 25 Statistics and Computational Biology Laboratory, Cancer Research UK Cambridge Institute, Cambridge, Cambridgeshire, United Kingdom; 26 School of Mathematics and Statistics/School of Medicine, University of St Andrews, St Andrews, Fife, Scotland; 27 HCA Pathology Laboratories, HCA Healthcare, London, United Kingdom; 28 Organisms and Ecosystems, The Earlham Institute, Norwich, Norfolk, United Kingdom; Broad Institute, UNITED STATES

## Abstract

A variety of models have been proposed to explain regions of recurrent somatic copy number alteration (SCNA) in human cancer. Our study employs Whole Genome DNA Sequence (WGS) data from tumor samples (n = 103) to comprehensively assess the role of the Knudson two hit genetic model in SCNA generation in prostate cancer. 64 recurrent regions of loss and gain were detected, of which 28 were novel, including regions of loss with more than 15% frequency at Chr4p15.2-p15.1 (15.53%), Chr6q27 (16.50%) and Chr18q12.3 (17.48%). Comprehensive mutation screens of genes, lincRNA encoding sequences, control regions and conserved domains within SCNAs demonstrated that a two-hit genetic model was supported in only a minor proportion of recurrent SCNA losses examined (15/40). We found that recurrent breakpoints and regions of inversion often occur within Knudson model SCNAs, leading to the identification of *ZNF292* as a target gene for the deletion at 6q14.3-q15 and *NKX3*.*1* as a two-hit target at 8p21.3-p21.2. The importance of alterations of lincRNA sequences was illustrated by the identification of a novel mutational hotspot at the *KCCAT42*, *FENDRR*, *CAT1886* and *STCAT2* loci at the 16q23.1-q24.3 loss. Our data confirm that the burden of SCNAs is predictive of biochemical recurrence, define nine individual regions that are associated with relapse, and highlight the possible importance of ion channel and G-protein coupled-receptor (GPCR) pathways in cancer development. We concluded that a two-hit genetic model accounts for about one third of SCNA indicating that mechanisms, such haploinsufficiency and epigenetic inactivation, account for the remaining SCNA losses.

## Author summary

Cancer is a genetic disease where changes in DNA cause alterations in the control of cellular systems leading to unchecked growth. Copy number changes, including duplications, amplifications, and deletions, are a common type of DNA change observed in cancer cells but it is not always clear which of the changes are important in driving cancer development. We have examined this class of genetic alteration in prostate cancer by DNA sequencing the whole genome in 103 cancers. 64 recurrent copy number changes were detected, of which 28 were new. For genetic losses our study comprehensively assessed the role of a model called the “Knudson two-hit genetic model” where alterations in both alleles of a gene is required to generate functional alterations. This model was only supported a minor proportion of recurrent deletions (15/40). This observation indicates that other mechanisms, such haploinsufficiency and epigenetic inactivation, may account for the majority of deletions. Our studies highlight several novel changes including those in non-coding lincRNA sequences, the identification *ZNF292* as a target gene for a recurrent deletion on chromosome 6, and the common Knudson deletions at the *NKX3*.*1* loci on chromosome 8.

## Introduction

Somatic copy-number alterations (SCNAs) occur very frequently in human cancer and exactly how these alterations contribute to cancer development is a subject of considerable interest. Mapping of SCNAs has identified recurrent sites of alterations in many cancer types, but only a small proportion of such sites have unambiguously been assigned to specific cancer genes [1].

Several models based on clonal evolution and selection may be invoked to explain recurrent regions of chromosomal loss in the autosomes and sex chromosomes. In the classic model of cancer development proposed by Knudson [2], mutations are required in each of the two copies of a single gene: the loss of an allele is considered as one mutation and the remaining allele would be altered by loss (homozygous deletion), mutation or rearrangement. This is due to strong positive selective pressure for alteration of both alleles. In principle, inactivation of the remaining allele might also involve epigenetic inactivation through DNA methylation. Haploinsufficiency is also an established mechanism of cancer development, where loss of only a single allele is required for cancer development; expression of the normal allele is retained in the cancer, albeit at a lower level. This model is supported in prostate cancer by transgenic mouse studies for *NKX3*.*1* and *p27*^*Kip1*^ [3,4].

Solimini *et al*. [5] have provided a model, where the collective contribution of many genes may provide selective advantage to a cancer cell, possibly overlapping with the Knudson model. De *et al*. and Fudenberg *et al*. have proposed a model where 3D chromatin organization and spatial co-localization of DNA regions during replication may explain the generation of copy number alterations [6,7]. Another explanation is that alterations are simply the hallmark of an unstable genome and have no particular functional significance, for example reflecting fragile genomic sites. Similar consideration can be given to regions of genetic gain and amplification where overexpression of one or more genes is believed in many cases to drive cancer development [8].

Prostate cancer is the second most common cancer in men worldwide and in 2012 an estimated 307,000 men died from prostate cancer worldwide [9]. Several studies have investigated SCNA in prostate cancer [10–12], and critically, it has been established that the burden of SCNA is associated with subsequent biochemical recurrence (rising Prostate Specific Antigen, PSA, levels after radical prostatectomy) and metastasis independent of initial PSA levels and Gleason scores. Similar observations were reported in a recent study where a 100-loci (276 genes) copy number signature was predictive of biochemical recurrence [13]. However, only a small proportion of the gains and losses have been unambiguously assigned to specific driving genes.

The current study is the first to implement a targeted approach in prostate cancer where Whole Genome DNA Sequencing (WGS) data is used to comprehensively examine mutation data in relation to the presence of SCNAs. We used WGS data from each patient to screen recurrent SCNA regions for potentially functional alterations not only in protein coding genes, but also in genomic regions encoding lincRNAs, in control regions, and in other conserved DNA sequences. Understanding mechanisms of SCNA generation and the identification of target genes, linked to clinical outcomes, may assist in identifying novel biomarkers and therapeutic targets.

## Results

### Genome wide copy number profiles

ASCAT 2.2 [14,15] was used to identify somatic copy number alterations (SCNAs; S2 Table; types of alteration defined in S1 Table) in WGS data for malignant samples taken from 103 prostate cancer patients (S2 Table). In cases of patients with multiple tumor samples a single profile was used. The relationship between samples within a patient and the tumour evolution for the 13 patients where there were multiple tumour samples has been previously investigated [16,17]. ETS gene status was inferred from WGS data (S3 Table).

**Fig 1:**
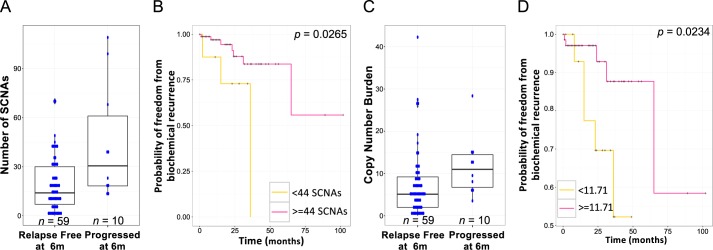
Somatic copy number alterations in 103 patients. (A) Somatic copy number alterations in relapse free (n = 59) and progressed patients (n = 10) after radical prostatectomy at six months and (B) association with time to biochemical recurrence (the two groups were defined relative to the mean number of SCNAs in progressed patients (44 SCNAs) within the cohort). Progressed patients had significantly more SCNAs than relapse free patients (a mean of 19 compared to 44; Mann-Whitney *U p* = 0.0133). (C) Copy number burden in relapse free and progressed patients after radical prostatectomy at six months and (D) association with time to biochemical recurrence (the two groups were defined relative to the mean copy number burden in progressed patients within the cohort). Progressed patients had lower copy number burden (mean of 7.359 compared to a mean of 11.710; Mann-Whitney *U p* = 0.0166).

When compared to prostatectomy cases, metastatic cancers had higher proportions of tetraploid genomes as defined by ASCAT (53% vs 19%; Fisher’s exact test, *p* = 0.0042; S4 Table), significantly larger numbers of SCNAs (mean 83 vs 21; Mann-Whitney *U p =* 2.17x10^-08^), higher copy number burden (percentage of the genome altered) (mean 31.24% vs 7.694%; Mann-Whitney *U p =* 1.30x10^-08^), and longer average SCNA size (Mann-Whitney *U p* = 4.37x10^-04^). Patients with more than 44 SCNAS (the mean number of SCNAs in patients that had progressed within six months) had worse prognosis (Log-rank test *p* = 0.027, median follow up of 16.5 month; Fig 1A and 1B) than patients with fewer SCNAs, consistent with other publications [13,18,19] linking higher numbers of SCNAs to poorer outcome. Patients with higher copy number burden had worse prognosis (Log-rank test *p* = 0.023; Fig 1C and 1D).

### Classification of prostate cancer based on copy number profiles

**Fig 2:**
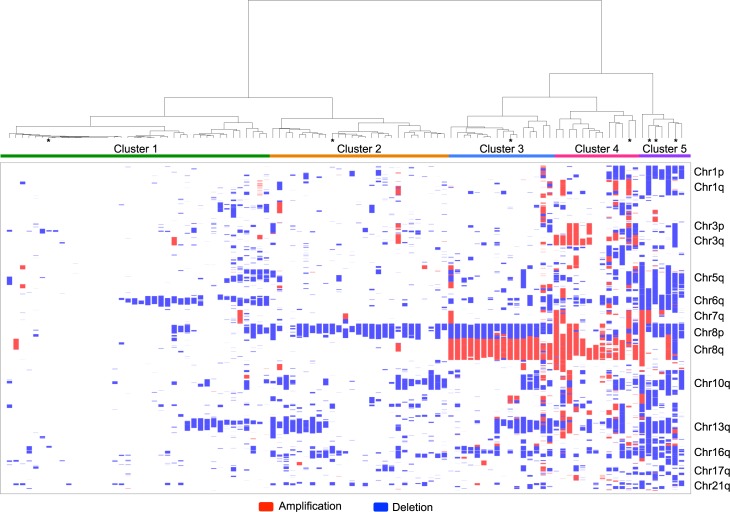
Cluster dendogram and heatmap of somatic copy number alterations in 103 patients. Cluster dendogram and heatmap of amplifications (red) and deletions (blue) representing the somatic copy number profile of the 103 patients calculated using unsupervised hierarchical clustering with Manhattan distance and complete inter-cluster linkage. Samples with SNP6.0 data are indicated with an asterisk. Confidence intervals determined by multiscale bootstrap resampling are displayed in S2 Fig.

**Fig 3:**
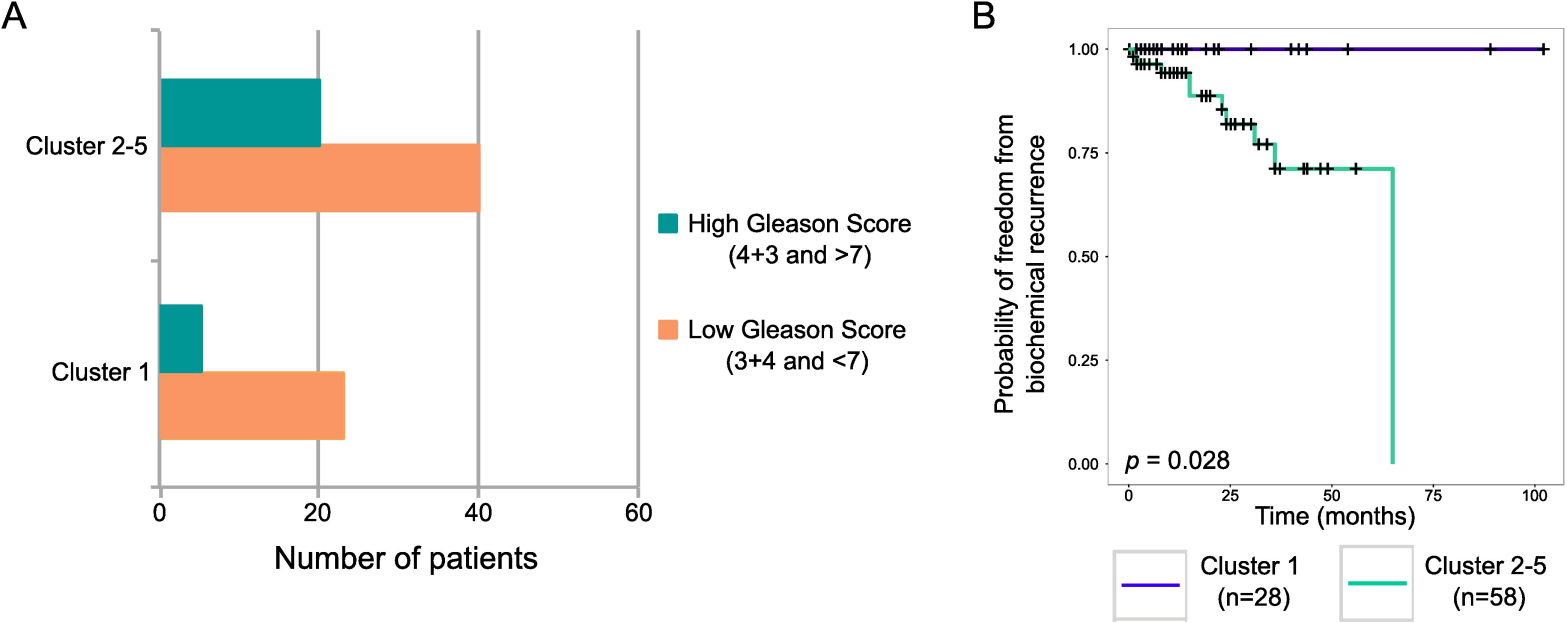
Cluster associations with clinical factors. (A) Clusters association with Gleason Score (prostatectomies and TURP samples only). (B) Clusters association to risk of biochemical recurrence within C1 and C2-C5. At the time of writing the single prostatectomy case in C5 had a follow up time of 23 months and had not progressed.

Hierarchical clustering analysis was applied on overlapping SCNAs present in at least five patients. A binary matrix was constructed on the basis of a patient having a region of amplification or deletion (1) or not (0). Five major clusters were observed: C1-C5 (Fig 2; S5 Table). C5 and C4 were composed mainly of metastatic cases (6/13 and 5/7) while the remaining metastatic cases were placed in C2 (3/39) and C3 (2/16). Patients in C1 had fewer SCNAs than those in C2-C5 (mean 6.79 vs 40.32 SCNAs) and lower copy number burden (mean 1.64% vs 14.98%). Prostatectomy/TURP patients in C1 had no significant difference in Gleason Scores (*X*^2^ test *p* = 0.213; Fig 3A) or in their levels of PSA at diagnosis (Mann-Whitney *U p* = 0.929). Prostatectomy patients in C1 had a significantly better prognosis (Log-rank test *p* = 0.028; Fig 3B).

### Detecting regions of recurrent alteration

**Fig 4:**
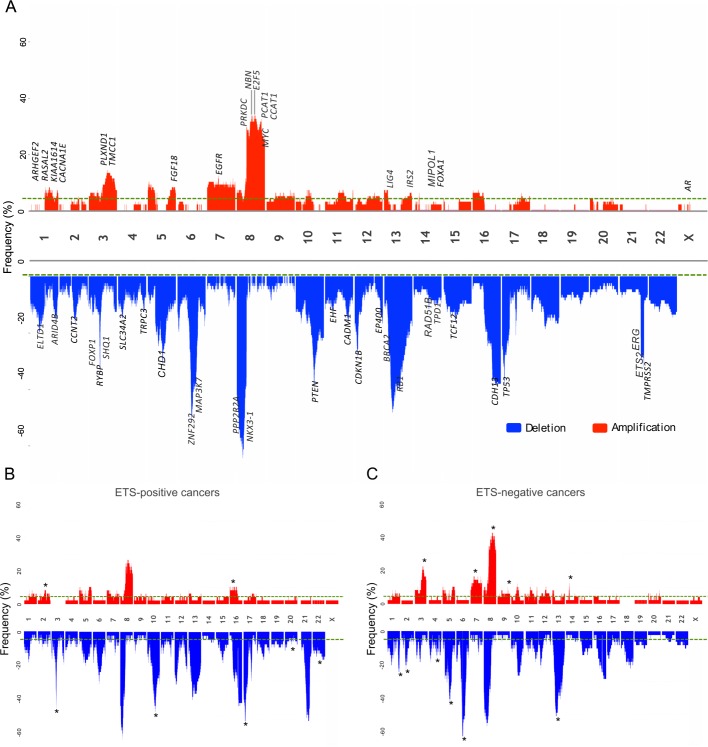
Frequency plot representing regions of amplification (red) and deletion (blue). Across samples from (A) all 103 patients, (B) ETS-positive cancers, and (C) ETS-negative cancers. SCNAs present in more than one patient were plotted. The green lines represent the cut-off (5 patients) for defining recurrently altered regions. Chromosome numbers are indicated in black. Genes potentially involved in prostate cancer development in the minimal regions of alteration are annotated in black.

We defined a recurrent region of alteration as the minimal region of overlap that contains supporting alterations from five or more patients (minimal MRA, Fig 4A). 40 regions of deletion and 24 regions of gain were identified (S6 Table). To identify potential driver genes, we screened sequencing data to determine whether each MRA contained point substitutions, insertions and/or deletions within the coding region of genes (Table 1, S7 Table), in cancer-related and conserved lincRNAs from MiTranscriptome [20] (S8 Table), in promoter regions (S9 Table) and in DNA High-occupancy target (HOT) regions [21] (S10 Table). To reduce the effect of outlier measurements in our data, when screening for mutations we used a larger minimal common region of alteration, referred to as the extended MRA, which is defined by removing the two alterations that were closest to the 5’ boundary of the minimal MRA and similarly the two alterations closest to the 3’ boundary (S1 Fig, S6 Table). We also applied the statistical method GISTIC [22] to define significant regions of gain and loss (15 gains and 19 losses, residual *q* < 0.05, S11 Table). 14 losses and one gain were common to our list of minimal MRAs and 5 losses and 14 gains were only detected by GISTIC (S6 Table). 16 out of 34 significant GISTIC regions (residual *q <* 0.05) were also detected in other prostate cancer studies that applied GISTIC [23,24], only four of which were not detected by our approach.

**Table 1:** Genes that are recurrently altered by coding-changing mutations in regions of recurrent somatic copy number alteration defined by the extended MRAs.

Gene	Gene name	Number of mutations	Patients with a SCNA and mutation	Patients with SCNA (%)	Type of SCNA
*FOXA1* (14q21.1)	Forkhead box protein A1	11	1	6 (5.83)	Amp
*TP53* (17p13.1)	Tumor protein p53	9	9	37 (35.92)	Del
*PTEN* (10q23.31)	Phosphatase and tensin homolog	9	7	39 (37.86)	Del
*HMCN1* (1q25.3)	Hemicentin-1	6	1	8 (7.77)	Amp
*KIF26B* (1q44)	Kinesin family member 26B	4	2	7 (6.80)	Amp
*KIAA1614* (1q25.3)	Uncharacterized Protein KIAA1614	4	1	8 (7.77)	Amp
*TCF12* (15q21.3)	Transcription factor 12	3	1	15 (14.56)	Del
*CACNA1E* (1q25.3)	Calcium channel, voltage-dependent, R type, alpha 1E subunit	3	1	8 (7.77)	Amp
*PHYKPL/AGXT2L2* (5q35.3)	5-Phosphohydroxy-L-lysine phospho-lyase	3	0	8 (7.77)	Amp
*SCN4A* (17q23.3)	Sodium channel protein type 4 subunit alpha	3	0	4 (3.88)	Amp
*C17orf58* (17q24.2)	Chromosome 17 open reading frame 58	3	0	4 (3.88)	Amp
*RYR2* (1q43)	Ryanodine receptor 2	3	0	12 (11.65)	Amp
*ZFHX4* (8q21.11)	Zinc finger homeobox 4	2	2	31 (30.10)	Amp
*TRPA1* (8q13.3)	Transient receptor potential cation channel, subfamily A, member 1	2	2	31 (30.10)	Amp
*NBN/NBS1* (8q21.3)	Nibrin	2	2	32 (31.07)	Amp
*COL27A1* (9q32)	Collagen, type XXVII, alpha 1	2	1	5 (4.85)	Amp
*AMBP* (9q32)	Alpha-1-microglobulin/Bikunin precursor	2	1	5 (4.85)	Amp
*UIMC1* (5q35.2)	Ubiquitin interaction motif containing 1	2	1	6 (5.83)	Amp
*SLC26A2* (5q32)	Solute carrier family 26 (anion exchanger), member 2	2	1	8 (7.77)	Amp

### Known regions of genetic alteration

**Fig 5:**
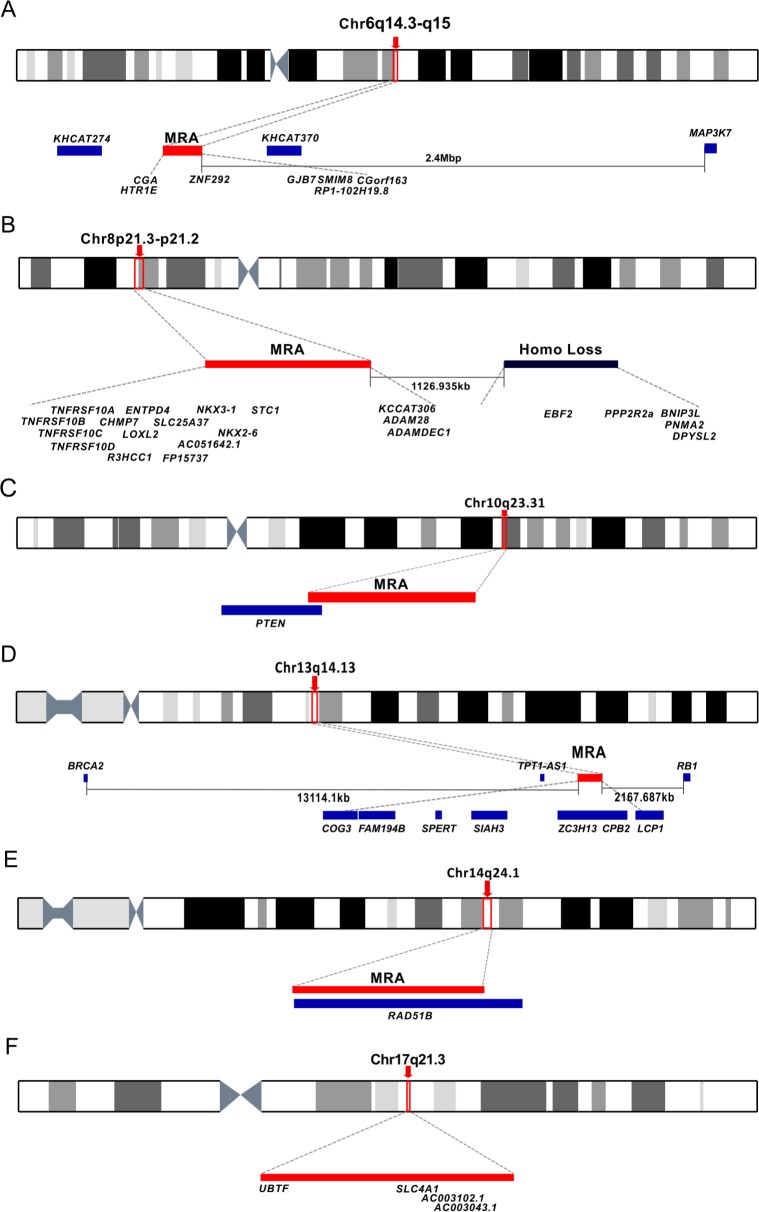
Examples of minimal recurrent deletions. Deletions at (A) 6q14.3-q15, (B) 8p21.3-p21.2, (C) 10q23.31, (D) 13q14.13, (E) 14q24.1 and (F) 17q21.31. The genomic location of the MRA and linked genes are displayed. For the loss at 8p a recurrent region of homozygous loss (8:25417422–26386565) close to the MRA is indicated.

The most frequent MRAs were located at 8p21.3-p21.2 (60% loss), 6q15 (49%), 13q14.13 (46%) and 10q23.31 (39%), in agreement with previous studies [18,24–26] (Fig 5, S3 Fig). 8p21.3-p21.2 is an example of a loss where haploinsufficiency has been proposed as the primary mechanism. The minimal MRA contains 16 coding genes, including *NKX3*.*1*, a suggested target gene for this region [24,25] (Fig 5B). The lincRNA *KCCAT306* (MiTranscriptiome ID) was the only sequence mutated more than once (S8 Table). *NKX3*.*1* was however, affected by a homozygous deletion in three patients (S12 Table). 1.1MBp from the minimal MRA there is a region affected by homozygous loss in five patients containing *PPP2R2a*, *EBF2*, *BNIP3L*, *PNMA2* and *DPYSL2*. *BNIP3L*, which counteracts the apoptotic inducer *BNIP3*, is another proposed target [26].

By comparison, 10q23.31 provided an example where alterations in both alleles are observed, consistent with the Knudson two-hit model [2], with *PTEN* as the target. A high proportion of the deletions were homozygous losses (11/39, S12 Table, Fig 5C). In seven cases when one allele was deleted, a mutation was found at the remaining *PTEN* allele (Table 1). *PTEN* alterations were more common in metastatic disease than in the prostatectomy series (93% vs 29%; Fisher’s exact test, *p* = 1.0x10^-08^). Consistent with previous studies [27,28], sometimes only the 5’ end of the *PTEN* gene was lost. 17p13.1 (35%) is another example of this class (S2T Fig, Table 1) where *TP53* was affected by mutation in nine patients who all had an associated SCNA and homozygous loss was seen in two patients.

*MAP3K7*, a proposed target for the 6q14.3-q15 deletion [25,26] is located 2.4Mbp away from the extended MRA (Fig 5A). *ZNF292* was mutated on the remaining allele in one patient and has been found to be rearranged in prostate cancer [29]. The lincRNAs *KHCAT274* and *KHCAT370* (MiTranscriptiome IDs) in the extended MRA were mutated four times on the allele that remained after deletion (Fig 5A, S8 Table). The known deletion at 17q21.31 [18,23,26] (Fig 5F) had a minimal MRA that contained *UBTF*, a known fusion partner of *ETV4* [30].

*RB1* and *BRCA2* are two proposed candidates for the MRA at Chromosome 13q14.13 (Fig 5D) [18,25,26]. *RB1* is located 1.3MBp from the minimal MRA and within the extended MRA. In three patients, a region of homozygous loss spans *RB1* (S12 Table). *BRCA2* was 9.5MBp outside the extended MRA.

Deletions containing only one gene in the minimal MRA, were detected at 3p13 containing *RYBP* (33.01%, S2D Fig), 12p13.1 containing *CDKN1B* (27.18%, S2N Fig), and 14q24.1 containing *RAD51B* (10.68%, Fig 5E) but none had mutations in the remaining allele (Table 1, S6 Table).

**Fig 6:**
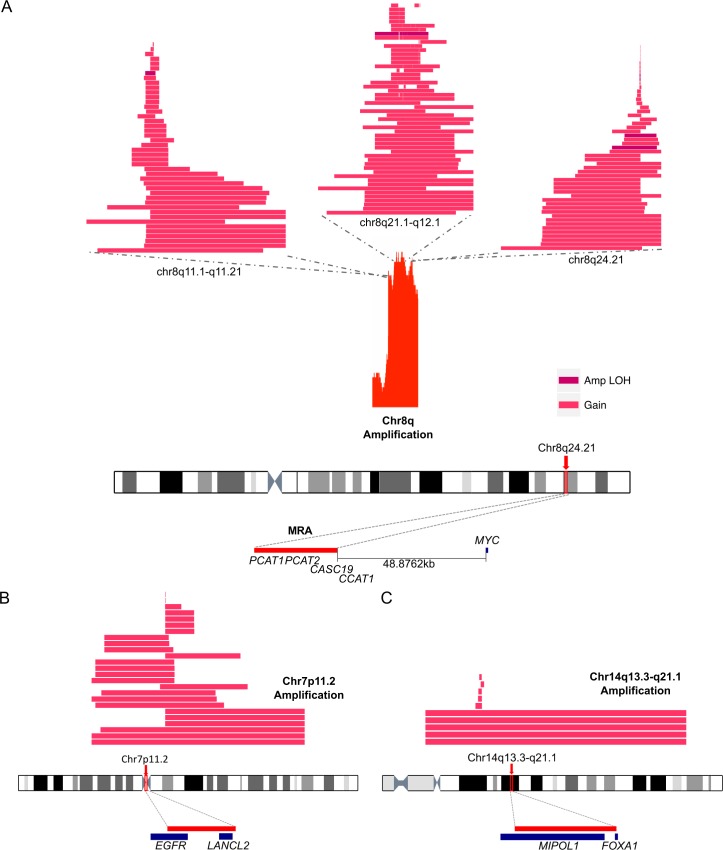
Examples of gains. Gains within the (A) q arm in Chr8, (B) focal amplifications at 7p11.2, and (C) 14p11.2. Three spatially separated regions of gain were present at 8q24.21. The chromosome gain events are represented with distinct colour blocks depending on the type of SCNA: gain (any gain in the number of normal allele copies) and amplification LOH (loss of one allele with any gain of the remaining allele). The regions of SCNA are ordered by length: top-smallest, bottom-largest. The genomic location of the MRAs and linked genes are additionally displayed.

Gains at Chr8q exhibited a complex structure with three broad peaks (Fig 6A, S4 Fig). The proposed target of 8q24.21, *MYC*, was located 48kb from the minimal MRA and was present in the extended MRA. Thirteen mutations were found in total at *PCAT1* and *CCAT1* with seven accompanying chromosome gain (S8 Table). The amplification at 7p11.2, present in 11.65% of patients, contained *EGFR* in the minimal MRA (Fig 6B). The extended MRA region contained a total of seven mutations with the highest number in lincRNA *CAT941*. Amplification of 14q13.3-q21.1 (Fig 6C) contained only *FOXA1* and *MIPOL1*.

### Novel regions of recurrent genetic alteration

24 additional novel recurrent regions of loss and gain were detected (S6 Table, S1 Appendix), including regions of loss with more than 15% frequency at 4p15.2-p15.1 (15.53%), 6q27 (16.50%) and 18q12.3 (17.48%). Regions of gain with the highest frequencies were at 1q25.3 (8.74%), 5p15.31 (9.71%), and 10q21.1-q21.3 (8.74%). For two of these regions, 22q12.1-q12.2 and 17q22-q23.1, Fraser *et al*. found a very close recurrent region of copy number alteration [24]. Within the extended MRA no more than a single mutation was observed. These novel regions of recurrent genetic alteration require confirmation in other, larger series.

### Novel targets in regions of genetic alteration

Apart from *PTEN* and *TP53* the most frequently mutated genes in extended MRAs are *FOXA1* (Fig 4C), *HMCN1*, *KIF26B*, and *KIAA1614*, indicating potential targets (Table 1). For *KIF26B*, *NBN*, *TRPA1* and *ZFHX4* the alleles were commonly both amplified and mutated. Gains in *NBN* are predictive of biochemical relapse in patients treated with radiotherapy [31]. Several lincRNAs were identified as potential novel targets (S8 Table). *KCCAT42*, *FENDRR*, *CAT1886* and *STCAT2* provided a hotspot of mutations within the loss at 16q23.1-q24.3: 17/33 mutations occurred in the remaining allele following loss. *KCCAT91*, *CCAT1*, *CAT1886* and *KCCAT199* were all found frequently mutated (> = 7 patients with mutation) and found to be concurrent with the associated SCNA more that 50% of the time. Single mutations in the promoters of only two genes (*NKX2-6* and *TPBGL*) occurred concurrently with heterozygous loss (S9 Table). XOT.290 was the only DNA High-occupancy target (HOT) region found to be mutated in more than two patients (S10 Table).

The most frequently altered genes within regions of homozygous loss outside the MRAs were *PCDH17*, *SYNE1*, *ASXL2*, *DNAH9*, *KIF1A*, *LMO7* and *LRP1B* (S7 Table). Except for *ASXL2* and *LMO7*, all these genes are reported to be frequently methylated in prostate and/or other cancers [32–37]. Deletion and mutations in *ASXL2*, a chromatin/histone modifier gene that interacts with AR, are associated with castration resistant prostate cancer [38]. *PCDH17* methylation is predictive of biochemical recurrence after radical prostatectomy [39,40].

### Conserved regions of DNA in regions of genetic alteration

Conserved regions of DNA were determined from multiple alignments of 45 vertebrate genomes to the hg19 human genome (UCSC phastCons *p*-value > 0.95). Deletion MRAs were significantly enriched for conserved regions (*p* = 0.0058; Wilcoxon signed rank test) while gains were not (*p* = 0.53). Deletions at extended MRAs 12p13.1 (28 patients), 17p13.1 (37 patients) and 17q21 (20 patients) had the highest percentage of conserved sequence (>10%, S6 Table). Recurrent mutations were found in 20 conserved regions (S13 Table). Of potential interest is the conserved region at 14:38061176–38061238 within the *FOXA1* gene with five mutations, which are exclusively found in patients without the SCNA. One of these, *FOXA1* M253R, was observed at the forkhead domain, a site of common mutation clustering [23]. Established tumor suppressor genes *PTEN* and *TP53* had recurrent mutations in conserved coding regions (S13 Table). 100% (9/9) of the mutations detected in *PTEN* were found in a conserved region. Two of these mutations, PTEN Y68H and PTEN R173C, are known to inhibit the activity of the phosphatase catalytic domain of PTEN [41] and occur frequently in gliomas and endometrial cancers [42]. In addition, *KIF26B* and *HMCN1* also had recurrent mutations in three or more patients in conserved coding regions. *KIF26B* is associated with poor prognosis in breast and colorectal cancers [43,44].

### Deletions frequently occur in combination with inversions and other chromosomal rearrangements

Analysis of WGS using the Brass algorithm identified breakpoints not associated with the copy number change within or close to the extended MRA. Recurrent breakpoints affecting four or more patients were identified in 153 genes (S14 Table). A common feature of many of these genes is that a region of inversion affects them. For example, out of the fifty patients that have the MRA deletion at 6q14.3-q15, in 14 patients there is a region of inversion found covering 15 genes including *GJB7*, *HTR1E*, *SLC35A1*, *ZNF292* which have been found to be affected by breakpoints previously [29]. *ZNF292* was also found to have homozygous deletion in two patients and a mutation in one patient. Taken together, *ZNF292* therefore seems the likely target of this deletion. *PCNXL2*, *TBCE*, and *THSD7B* presented a chromosomal rearrangement in the remaining allele in more than 40% of patients with a SCNA. *THSD7B*, a gene involved in the TGFβ signaling pathway, has been previously found to be the target for mutations [45]. *NCKAP5* is a gene in the large region of inversion at 2q21.3-q22.1 and has been observed to be rearranged in prostate cancer by FISH [46]. Regions of inversions were also observed in GISTIC detected deletions (S15 Table). For example, 15 patients have an inversion at the 2q22.1 deletion that covers *SPOPL*, *NXPH2* and *HNMT*, of which at least four also had a deletion. *SPOPL* is capable of associating with the putative transcriptional regulator of AR [47,48], SPOP and has been previously found to be the target of recurrent mutations [23,29].

### Clinical correlations

**Fig 7:**
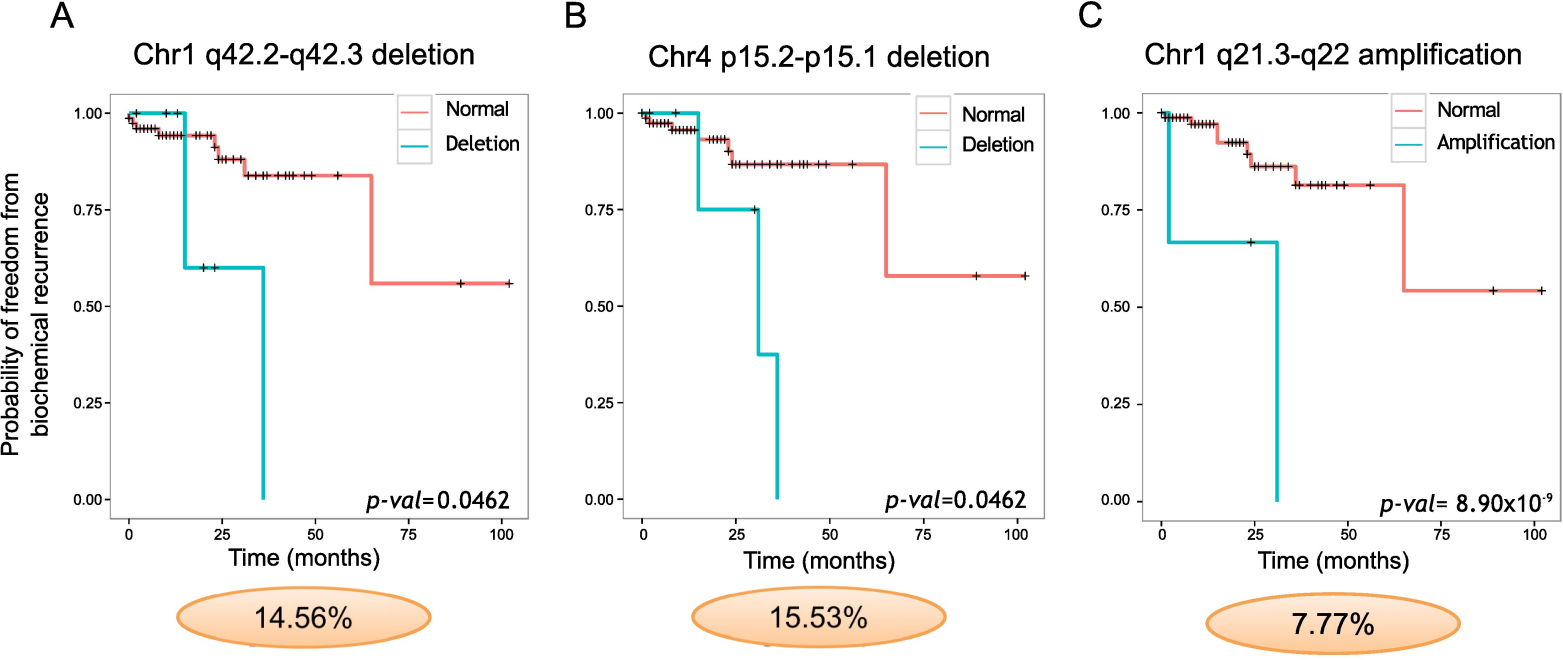
Minimal regions of deletion and amplification significantly associated with treatment failure. (A), 1q42.2-q42.3 deletion, (B) 4p15.2-p15.1 deletion, and (C) 1q21.3-q22 amplification. Kaplan-Meier freedom from biochemical recurrence are displayed. Log-rank test probabilities adjusted for multiple testing by the Benjamini-Hochberg method are indicated. Ovals indicate the frequency of alteration.

Clinical correlations were made using data from prostatectomy patients (S6 Table). No MRAs were significantly associated with Gleason score (*X*^2^ test (FDR); *p* > 0.05) or PSA levels (Mann-Whitney *U* (FDR) *p* > 0.05). Two deletions and seven gains were significantly associated with time to biochemical recurrence (Log-rank test (FDR); *p* < 0.05, Fig 7). This includes a loss at 1q42.2-q42.3 which contains *ARID4B*, a chromatin-remodeling gene that interacts with *RB1*, and reduced expression is associated with the development of breast and other cancers [49,50]. Five of these nine regions were validated as having a significant association with biochemical recurrence in two other datasets, TCGA [23] and Taylor *et al*. [18], with a further two regions validated in one dataset (Log-rank test (FDR); *p* < 0.05, S6 Table). Seven of the nine regions with a significant clinical association were identified as novel recurrent regions of loss and gain. Six of these were validated in at least one other dataset (S6 Table). GISTIC detected one of the regions, 4p15.2-p15.1, exhibiting clinical significance.

### ETS gene fusions and pathway enrichment analysis

49 patients had ETS positive (48%) and 54 patients (52%) had ETS negative cancers (Fig 4B and 4C). Deletions at 3p13 (*RYBP*), 17q21.31, 20p13, 21q22.2, 21q22.3 and 22q13.31 and amplifications at 2q24.3 and 16p13.3 were more commonly present in ETS-positive cancers (Fisher’s exact test, *p* < 0.05, S6 Table). Deletions at 1q42.2-q42.3, 2q21.3-q22.1, 4q22.3, 5q21.1 (*CHD1*), 6q14.3-q15 and 13q14.13 and amplifications at 3q22.1-q21.3, 7p11.2 (*EGFR*), 8q11.1-q11.21, 8q21.11-q22.1, 8q24.21, 9q33.1, 12q23.1 and 14q13.3-q21.1 (*FOXA1*) were more commonly present in ETS-negative cancers (Fisher’s exact test, *p* < 0.05). These observations are in agreement with previous studies associating subtypes of ETS negative cancers with *CHD1* (5q21.1) [23,51,52], *FOXA1* (14q13.3-q21) [51], 2q21.3-q22.1 [23], 6q14.3-q15 [23,53], and 13q14.13 deletions [23] and ETS positive cancers with *PTEN* deletion [18,23,54] (Fisher’s exact test, *p* = 0.08). Several new associations with ETS negative cancers were found, including deletions at 1q42.2-q42.3 & 4q22.3, and amplifications at 3q22.1-q21.3, 7p11.2 (*EGFR*), & 8q (*MYC*). The deletion at 17q21.31 and the amplification at 16p13.3 were associated with ETS positive cancers.

Reactome pathway enrichment analysis of ETS-positive and ETS-negative cancers was performed (S5 Fig, S16 Table). Altered pathways and processes common to ETS-positive and ETS-negative cancers were the cell cycle, cellular senescence, ion channels and WNT signaling. Aberrations in the cell cycle are prognostic of outcome in prostate cancer patients [55,56]. The major pathways altered more prominently in ETS-positive cancers were the PI3K-AKT, EGFR, TGF-beta Receptor Complex, PDGF and FGFR signaling pathways. ETS-negative cancers had more common alterations in the GPCR signaling pathway and DNA replication. An additional analysis of genes altered by mutation in the significant regions of amplification, deletion and homozygous loss showed enrichment of the DNA repair, PI3K/AKT and cell cycle pathways with no difference between ETS-positive and ETS-negative cancers (S16 Table).

## Discussion

Recently, driven by technological improvements and cost reductions, considerable attention has been directed towards the whole genome analysis of cancer genomes. We employed this technology to assess mechanisms of copy number gain and loss. Our study confirmed several previous observations and yielded novel features of the human prostate cancer genome, including highlighting the importance of lincRNAs in prostate cancer development [57–59]. Previously, lincRNAs *PCAT1* and *CCAT1* were identified as targets in the 8q24.21 gain [60,61] and we confirmed this and demonstrated the presence of mutations in patients with gains. *PCAT1* and *CCAT1* have been identified as prognostic markers in prostate and colorectal cancers acting as transcriptional regulators of the genes *BRCA2* and *MYC* respectively [60,62]. We also identified a hotspot of mutations at the *KCCAT42*, *FENDRR*, *CAT1886* and *STCAT2* loci within the 16q23.1-q24.3 loss. Low *FENDRR* expression is associated with poor prognosis in gastric cancer [63]. *KCCAT91*, *CCAT1*, *CAT2185* and *KCCAT199* were also identified as possible targets.

Our observations show that many well-characterised regions of recurrent loss are also the site of clusters of breakpoints. The remaining allele of *NKX3*.*1* was affected by a breakpoint in a high proportion of patients (8/62) suggesting that sometimes both alleles may be inactivated (S14 Table). Supporting this view it has also been reported that 1% of prostate cancers contain point mutations in the *NKX3*.*1* allele [23], and rearrangement of this gene was also reported by Baca *et al*. [29]. These observations are inconsistent with the view that inactivation of *NKX3*.*1* always involves haploinsufficiency, although it is possible that both Knudson and haploinsufficiency, mechanisms are active. We found that the gene *ZNF292* was present in the minimal MRA and was in a region of inversion or mutated in the remaining allele in 15 cases and had homozygous loss in two cases providing strong support for *ZNF292* as a classic two-hit target at this locus. Rearrangements were also detected in the remaining allele at the *CHD1*, *PTEN*, *USP28* and *TP53* loci. For *PTEN* deletion of one allele accompanied by loss of function of the remaining allele caused by a chromosomal rearrangement was consistent with our previous FISH studies of the *PTEN* allele [27].

We confirmed the finding of Taylor *et al*. [18,19] that the burden and number of SCNAs are predictive of biochemical recurrence. We additionally identified nine regions that were significantly associated with relapse. Pathway analysis of genes present in deleted regions had previously identified cell cycle (RB1), PI3K, WNT and RAS/RAF pathways [18,48]. We have confirmed the importance of DNA repair pathways [23,48] and highlighted other processes including ion channel and GPCR signalling pathways.

We also assessed whether our analysis using whole genome DNA sequencing data were consistent with previously proposed mechanisms accounting for gains and losses. The *PTEN* gene provides a classic example of a Knudson two hit model where both alterations involve genetic alterations: deletion of one allele is accompanied by rearrangement or mutation in the remaining allele. However, we could find few other examples of this model (15/40 MRAs) when specifying that alterations must occur exclusively within the extended MRA (Table 2; S2 Appendix). This is confirmed with the regions detected by GISTIC (13/31 regions; S17 Table). One possibility is that regions of loss may be accompanied on the opposite alleles by alterations in mini-drivers genes [64] thus making a small but selectable contribution to cancer development. Recurrent mutations within the regions of copy number change were predominantly (81%) in cases that had not lost or gained an allele. Such alterations (for example *FOXA1*, *KIF26B* and the lincRNAs *CAT1800* at 16p13.3, *KHCAT81* at 16p13.12-p13.11 and *BRCAT9* and *BRCAT3* at 11q14.3) are possible haploinsufficiency targets (S7 Table).

**Table 2:** Summary table of recurrent regions of deletion that follow the Knudson hit model.

Minimal Region of Alteration Band	Candidate Genes/Region	Chromosomal Location	Number of Patients with Deletion	Homozygous Deletion	Number of Patients affected by Deletion and a Point Mutation/Indel on the opposite allele	Number of Patients affected by Deletion and a Breakpoint/Inversion on the opposite allele
**1q31.1**	***AK5*, *GIPC2*, *LPHN2*, *& TTLL7***	1:77747736–84464833	19	0	0	5
**1q42.2-q42.3**	***PCNXL2 & TBCE***	1:233119181–235612283	14	0	0	6
**2q21.3-q22.1**	**12 genes inc. CCNT2, ZRANB3, *CXCR4*, *DARS*, *NCKAP5***	2:133429374–136875735	16	0	0	4
**2q21.3-q22.1**	***THSD7B***	2:137523115–138435287	14	0	0	6
**3p13**	***RYBP***	3:72420976–72496069	34	0	0	5
**3p13**	***SHQ1***	3:72798428–72911065	33	0	0	4
**5q21.1**	***CHD1***	5:98190908–98262240	29	6	0	8
**5q21.1**	***KCCAT91***	5:98150767–100530128	32	4	5	6
**5q21.1**	***ST8SIA4***	5:100142639–100238970	28	0	0	7
**5q31.1-q13.2**	***MAP1B*, *MRPS27*, *ZNF366***	5:71403061–71803554	22	0	0	4
**6q14.3-q15**	***KHCAT370*.*2***	6:87851165–88089974	50	1	3	14
**6q14.3-q15**	***ZNF292***	6:87862551–87973914	50	2	1	14
**8p21.3-p21.2**	***NKX3*.*1***	8:23536206–23540440	62	3	0	8
**8p21.3-p21.2**	***PEBP4*, *RHOBTB2*, *TNFRSF10A*, *TNFRSF10B*, *TNFRSF10C*, *TNFRSF10D***	8:22570769–23082639	62	0	0	9
**11q23.2**	***USP28***	11:113668596–113746292	14	0	5	5
**11q23.2**	***CADM1 & HTR3A***	11:113845603–115375675	15	0	0	4
**12p13.1**	***DUSP16 & LOH12CR1***	12:12510013–12715317	27	0	0	4
**12q24.33**	***TMEM132D***	12:129556270–130388211	14	0	0	4
**13q14.13**	***RB1***	13:48877887–49056122	46	3	1	0
**13q14.13**	**19 genes inc. *GTF2F2*, *HTR2A***	13:45007655–47471169	47	0	0	8
**16q23.1-q24.3**	***KCCAT42*, *CAT1886*.*1*, *FENDRR*, *STCAT2***	16:78475408–90287535	42	0	17	0
**17p13.1**	***TP53***	17:7565097–7590856	37	2	9	5
**22q13.31**	***CELSR1*, *FLJ27365*, *PPARA*, *TRMU***	22:46449749–46933067	14	0	0	4

From these analyses, we conclude that a simple genetic Knudson model does not account for the majority of deletions in prostate cancer. It is possible that genes in the remaining allele may be inactivated by methylation or that haploinsufficiency may account for regions of gene loss.

The criterion for membership of this group was that there had to be at least four mutations (excluding synonymous changes), breakpoints within the gene or inversions affecting the gene in the remaining allele of a gene in the extended MRA. Multiple genes are displayed when an inversion affects multiple genes and there is no additional evidence of which one is the target. *USP28* is located very close to the extended MRA at 12q23.2 and probably represents another example of the Knudson two hit model. *KCCAT42*, *FENDRR*, *CAT1886* and *STCAT2* are a cluster of lincRNAs. A version of this table for GISTIC regions can be found in S17 Table.

## Methods

### Sample cohort

141 malignant samples from 105 patients with prostate cancer within the Cancer Research UK ICGC Prostate Cancer Project were included in this study: 88 cancers collected after radical prostatectomy, including 10 tumor and three blood samples from three men with complex or multifocal disease; five cancers from men diagnosed with prostate cancer in Shanghai; two cancers collected from transurethral resection of the prostate (TURP); 8 lymph node metastatic samples from non-androgen deprived patients; and 36 malignant samples from 7 men with metastatic disease belonging to the PELICAN rapid autopsy program [17]. Samples were collected subject to ICGC standards of ethical consent. Ethical approval for this work was obtained from the respective local ethics committees (NHS South West London REC [10/H0806/113]; NHS East Midlands–Derby REC [01/4/061]; NHS East of England—Cambridge REC [03/018]; John Hopkins IRB [NA_00003925]; Changhai Hospital Ethics committee; Joint Chinese University of Hong Kong-New Territories East Cluster Clinical Research Ethics Committee [CRE-2011.373]) and from The Trent Multicentre Research Ethics Committee [MREC/01/4/061]. Explicit informed written consent was obtained from all participants to the use and storage of their genetic material and tissue samples in research, including whole genome sequencing. Explicit written consent was also obtained for any data derived from these samples, along with coded clinical/health data, to be placed on an international database (ICGC) and made available to ICGC members and other international researchers under open or controlled access. Prostatectomy samples were collected as described previously [65]. Blood samples were used as normal controls except for PELICAN samples where normal tissue was used.

### DNA preparation and DNA sequencing

DNA from whole blood samples and frozen tissue was extracted and quantified using a ds-DNA assay (UK-Quant-iT PicoGreen dsDNA Assay Kit for DNA) following manufacturer’s instructions with a Fluorescence Microplate Reader (Biotek SynergyHT, Biotek). Acceptable DNA had a concentration of at least 50ng/μl in TE (10mM Tris/1mM EDTA), with an OD 260/280 between 1.8–2.0. For aCGH at least 10μl equivalent to 500ng were used for hybridisation to the Affymetrix SNP6.0 array. WGS was performed at Illumina, Inc. (Illumina Sequencing Facility, San Diego, CA USA) or the BGI (Beijing Genome Institute, Hong Kong) as described previously to a target depth of 50X for the tumor samples and 30X for matched controls [66]. The Burrows-Wheeler Aligner (BWA) was used to align the sequencing data to the GRCh37 human genome [66].

### Generation of segmented copy number profiles

Pseudo-SNP6.0 profiles were created for each tumor and matched control from whole genome sequencing data. In a small number of cases data was obtained directly from Affymetrix SNP6.0 arrays (S18 Table) and rescaled and transformed using PennCNV [67]. ASCAT 2.2 was then used to generate segmented copy number profiles and estimate the purity and ploidy of the sample using a penalty of 50 and gamma value of 1 [14,15]. Note that given the target sequencing coverage of 50x/30x, there may be stochastic regions of low coverage where true segments are not detected. Two prostatectomy samples with low estimated tumor content were removed from further analysis. Data from the matched control is used to reduce false positives. A conservative filtering strategy was used to ensure the best quality of segments are obtained and give us the greatest confidence in the minimal regions of alteration that we call. 8876 segments were removed, of which 6692 were removed based on the following filtering criteria:

- Segment was within centromeric or telomeric regions (extended by 1,000,000 bp).
- Segment was within low mapability regions as defined by ENCODE (extended by 1,000,000 bp).
- Segment copy number of major allele (nMajor) >25 and copy number of major allele (nMinor) = = 0, or nMajor> = 10 and length was less than 500,000bp.
- Segment where the minor allele copy number is larger than the major allele copy number.
- Segment had less than 50 supporting SNPs and length was less than 100,000bp

The remaining 2184 segments were filtered out upon visual inspection in a blind fashion. Visual inspection included checking that the change in copy number was in agreement with the overall ploidy of the sample, that there was a change in BAF and total copy number, and that the SNPs within the segment didn’t have high variance. In the majority of cases segments were removed because ASCAT did not get the appropriate fit and there was no obvious copy number change. In this and other ICGC projects we have generally found that visual inspection of any class of alteration is an excellent method for filtering out incorrect calls made by the algorithms. Similar results were obtained from SNP6.0 and pseudo-SNP6.0 profiles (S6 Fig). We also applied the statistical method GISTIC v2.0.16 [22] to define significant regions of gain and loss, using the default parameters. Note that all pre-filtered segments were used when the GISTIC algorithm was applied.

### Conversion of sequencing data to a pseudo-SNP6.0 profile

The number of reads for each nucleotide base at each probe position on the Affymetrix SNP6.0 na32 hg19 annotation was extracted from sequence using deepSNV [68]. The logarithm of the ratio (*LogR*) between allele A and B and the B allele frequency (*BAF*) were calculated as follows:

$$LogR_{i}={\text{log}}_{2}(A_{i}+B_{i})$$

$$BAF=\frac{B_{i}}{\left(A_{i}+B_{i}\right)}$$

where *A*_*i*_ is the read count for allele A for probe *i* and *B*_*i*_ is the read count for allele *B*. For CN probes:

$$LogR_{i}={\text{log}}_{2}(N_{i})$$

where *N*_*i*_ is the total number of reads at the position of probe *i*. The LogR values were normalised:

$$LogR_{i}=LogR_{i}-\text{median}(LogR)$$

SNP probes with no reads at A and B alleles or with a *BAF* of 1 or 0 were removed. Using the SNP6 array probe positions for WGS data with ASCAT is a common approach, used successfully in the ICGC pancancer analysis of whole genomes project (https://dcc.icgc.org/pcawg) [69]. A wrapper program for ease of use is available [70].

### Calculation of the minimal regions of alteration (MRA)

Data from patients with multiple tumor samples is collapsed into one copy number profile per patient using all detected regions. Overlapping regions of SCNAs were identified across all patients by applying the GenomicRanges coverage function [71]; amplifications and deletions were analysed independently. The minimal MRA is defined by identifying the region most frequently altered in each peak region of overlap between five or more patients. This is an arbitrary threshold, but simplifying segment calling to a random process and assuming that a copy number will be called in a region 1% of the time by chance (which we would consider high), then given five patients, it is significantly likely that the true number of segments is greater than 1% (*p* = 0.0039; Exact binomial test). The extended MRA expands the minimal MRA by taking the start position of the overlapping segment with the third largest start position and the end position of the segment with the third smallest end position (S1 Fig).

### Mutational screen

Substitutions, insertions and deletions were detected using the Cancer Genome Project Wellcome Trust Sanger Institute pipeline. An updated version of this pipeline is available as a Docker image (Alignment: https://dockstore.org/containers/quay.io/wtsicgp/dockstore-cgpmap; Variant-calling: https://dockstore.org/containers/quay.io/wtsicgp/dockstore-cgpwgs). The Burrows-Wheeler Aligner’s Smith-Waterman Alignment (BWA-SW) v0.5.9-r16+rugo was used to align the sequencing data from each lane to the GRCh37 reference human genome using parameters -l 32 -t 6 [72]. Lanes that pass quality control are merged into a single well-annotated sample BAM file with PCR duplicate reads removed. Substitutions were detected using CaVEMan v1.3, an in-house bespoke algorithm developed at the Sanger Institute (http://cancerit.github.io/CaVEMan/), with a cut-off ‘somatic’ probability of 95%. CaVEMan utilises a Bayesian expectation maximization (EM) algorithm: Given the reference base, copy number status and fraction of aberrant tumor cells present in each cancer sample, CaVEMan generates a probability score for potential genotypes at each genomic position. Further post-processing filters were applied to eliminate false positive calls arising from genomic features that generate mapping errors and systematic sequencing artifacts. In comparisons with other mutation callers it has been found to be amongst the top performers in terms of sensitivity and specificity [73]. Only substitutions that were a missense, nonsense, start-lost, or occurred in a non-coding transcript were considered. Insertions and deletions were called using a lightly modified version of pindel v4.2 [74] (http://cancerit.github.io/cgpPindel/). Only indels that were inframe, frameshift, or occurred in a non-coding transcript were considered. Structural variants were detected using Brass (Breakpoints via assembly) v1.0.3, an in-house bespoke algorithm developed at the Sanger Institute (https://github.com/cancerit/BRASS). In brief, the first step is to combine discordant read pairs into potential regions where a breakpoint might occur. Next, reads around each potential region, including half-unmapped reads, are gathered and a local *de novo* assembly using Velvet is performed [75]. By analysing the De Bruijn graph pattern the breakpoint can be identified down to base pair resolution. Any breakpoints where an exact location could not be determined were removed. A positive ETS status was assigned if a breakpoint between *ERG*, *ETV1* or *ETV4* and previously reported partner DNA sequences [76] were detected. Genes in each MRA were identified using good quality transcripts annotated in the EnsEMBL Genome Reference Consortium Human Build 37.p13 (GRCh37.p13).

The mutational screen of homozygous loss was performed in any region that had a homozygous loss in at least one sample. For mutations that occur within promoters, EPDNew human version 003 [77] was used to define promoter regions. Conserved regions of DNA were determined from UCSC phastCons scores for multiple alignments of 45 vertebrate genomes to the hg19 human genome using a threshold *p*-value of 0.95. We used 7,942 long noncoding RNAs (lincRNAs) from the MiTranscriptome project that were detected in 7,256 RNA sequencing libraries from tumors, normal tissues and cell lines and defined as being either cancer-associated or containing conserved regions [20].

### Statistical tests and survival analyses

All statistical tests were performed in R [78]. For comparisons between groups the non-parametric Mann-Whitney *U* test was used for continuous variables and the *X*^2^ test with Yates' continuity correction or Fisher’s exact test was used for categorical variables. For all statistical tests used there is the assumption of independence between data and we have ensured this is the case by only using one copy number profile per patient in all comparisons. The sample used for the copy number profile was chosen at random (see S2 Table for selection). In all cases where the *X*^2^ test was applied no cells in the contingency table had an expected value less than 5.

Clinical associations were determined using standard statistical tests with Benjamin-Hochberg multiple testing correction applied. The log-rank test was used to perform survival analyses with biochemical recurrence after prostatectomy as the end point for survival analyses. Reasonable assumptions were made i.e. censoring is unrelated to progress, survival probabilities are the same for subjects recruited early and late in the study, and events happen at the times specified. 84 out of 86 prostatectomy patients were used; two patients had incomplete clinical data (0040 and 0052). For the patients where data was available from multiple samples, the patient was classified as having the minimal region of alteration if it occurred in any of the samples.

### Pathway analysis

The enrichment analysis was performed by exploring the Reactome canonical pathways using the Reactome plugin in Cytoscape [79] using the genes contained within the extended MRAs. Integration of Reactome and Kyoto Encyclopaedia of Genes and Genomes (KEGG) canonical pathways pathway analysis [80] was used to construct a map of the major pathways altered in prostate cancer within our cohort.

### Identifying genes affected by rearrangements

Rearrangement breakpoints within the extended MRA and within the significant GISTIC regions (*q*-value < = 0.05) were selected and used to define associated gene allele-specific events. Deletions that had common regions detected by ASCAT2.2 and Brass or by ASCAT2.2 only were included in the analysis. A gene was defined as being affected by a rearrangement, and hence a bi-allelic event, if an insertion, inversion or intra-chromosomal breakpoint occurred within the gene region or the gene occurred within an inversion. If a gene was inverted twice and the breakpoints of the rearrangement were outside the gene coordinates it was assumed the gene was still functional; these even inversions were removed from the analysis.

### Data access

Sequencing data has been deposited at the European Genome-phenome Archive (EGAS00001000262, S1 Table).

## Supporting information

S1 Fig Diagrammatic explanation on minimal and extended MRA. (PDF)

S2 Fig Hierarchical clustering of 103 prostate cancer samples with multiscale bootstrap resampling. The data are binary values corresponding to the presence/absence (1/0) of regions of copy number gain and loss in each of the tumour samples. *p*-values were calculated via hierarchical cluster analysis with multiscale bootstrap resampling of 1000 using Ward’s method and the Manhattan distance. The analysis was performed using the *pvclust* package in R. Values at branches are AU (Approximately Unbiased) *p*-values (left, red), and BP (Bootstrap Probability) values (right, green). Clusters significantly supported by the data (AU ≥ 95) are indicated by the red rectangles.
(PDF)

S3 Fig Copy number alteration segments detected by ASCAT that overlap with deletion MRAs. Each deletion is represented as a distinct colour as shown in the key. Deletions are as follows: neutral LOH (loss of one allele with duplication of the remaining allele); hemizygous deletion LOH (loss of one allele); homozygous loss (loss of the two alleles); and other loss (loss of one allele copy following whole genome duplication). Cases for which more than one sample was available are all indicated, however contribution to the frequency of the SCNA was defined on a per patient basis. The regions of SCNA are ordered by length: top-smallest, bottom-largest. Each block has been labelled with sample ID. (a) chr1 p31.1, (b) chr1 q42.2-q42.3, (c) chr2 q21.3-q22.1, (d) chr3 p13, (e) chr4 p15.2-p15.1, (f) chr4 q22.3, (g) chr4 q27-q28.1, (h) chr4 q34.3, (i) chr5 q13.1-q13.2, (j) chr7 q31.32-q31.33, (k) chr9 p22.3, (l) chr11 p13, (m) chr11 q23.2, (n) chr12 p13.1, (o) chr12 q24.33, (p) chr14 q24.1, (q) chr14 q32.13, (r) chr15 q21.3, (s) chr16 q23.1-q24.3, (t) chr17 p13.1, (u) chr18 p11.32-p11.31, (v) chr18 q12.3, (w) chr18 q23-q22.3, (x) chr19 p12, (y) chr19 q13.31, (z) chr20 p13, (a2) chr20 p12.1, (b2) chr20 q13.33, (c2) chr21 q22.2, (d2) chr21 q22.3, (e2) chr22 q12.1-q12.2, (f2) chr22 q13.31, (g2) Chr6q14.3-q15, (h2) Chr8p21.3-p21.2, (i2) Chr10q23.31, (j2) Chr13q14.13, (k 2) Chr17q21.3, (l2) Chr5q21.1, (m2) Chr6p25.1-p24.3, (n2) Chr6q27.
(PDF)

S4 Fig Copy number alteration segments detected by ASCAT that overlap with amplification MRAs. The chromosome gain events are represented with distinct colour blocks depending on the type of SCNA: gain (any gain in the number of normal allele copies) and amplification LOH (loss of one allele with any gain of the remaining allele). The regions of SCNA are ordered by length: top-smallest, bottom-largest. Each block has been labelled with sample ID. Cases for which more than one sample was available are all indicated, however contribution to the frequency of the SCNA was defined on a per patient basis. (a) chr1 q21.3-q22, (b) chr1 q25.3, (c) chr1 q43-q44, (d) chr2 q24.3, (e) chr5 p15.31, (f) chr5 q33.3-q35.2, (g) chr9 q33.1, (h) chr10 q21.1-q21.3, (i) chr11 q13.4-q13.5, (j) chr11 q14.3, (k) chr12 q23.1, (l) chr13 q11-q12.11, (m) chr13 q33.3, (n) chr13 q33.3-q34, (o) chr14 q13.3-q21.1, (p) chr16 p13.3, (q) chr16 p13.3, (r) chr16 p13.12-p13.11, (s) chr17 q22-q23.1, (t) Chr8q11.1-q11.21, (u) Chr7p11.2, (v) Chr8q21.1-q12.1, (w) Chr8q24.21, (x) Chr3q22.1-q21.3.
(PDF)

S5 Fig Commonly altered pathways in ETS positive and negative cancers. Blue and red blocks indicate genes contained in regions of deletion and amplification respectively. Grey blocks indicate genes with no alteration that were required for representation of the pathway. Purple and blue squares indicate the percentage of samples with a copy number alteration in that gene in ETS negative and positive samples.
(PDF)

S6 Fig A platform comparison of ASCAT profiles on a single sample. (a) one profile from SNP6.0 and (b) one from NGS data.
(PDF)

S1 Table Classification of somatic copy number alterations. (DOCX)

S2 Table Copy number profiling of 103 patients. Including ploidy, degree of contamination and number of SCNAs. Clinico-pathological characteristics of the study cohort consisting of 105 patients. Information on patients undergoing radical prostatectomy (88) and TURP (2) is displayed.
(XLSX)

S3 Table ETS gene status. (XLSX)

S4 Table Summary characteristics of the genomes and somatic copy number alterations (SCNAs). Left side: Samples classified by disease status i.e. samples from prostatectomies from patients free of metastatic disease (PT) or samples from patients with metastatic disease (M). Right side: Prostatectomy samples where there was at least six months follow up (n = 69) classified by whether there was rapid biochemical recurrence within six months of prostatectomy (PG) or not (RF) (right side). The two TURP samples are not summarized in this table. Results from statistical tests are shown that test whether there are significant differences between either metastatic and prostatectomy patients or prostatectomy patients that have biochemical recurrence within six months or not.
(DOCX)

S5 Table Summary of clinico-pathological characteristics of the patients in the defined sets of clusters. (DOCX)

S6 Table Minimal regions of deletions and amplifications with linked genes. This table includes: Comparison of MRAs, extended MRAs and GISTIC-detected regions; amplifications and deletions found in previous prostate cancer studies.; percentage of the minimal regions of alterations that are conserved regions; and clinical correlations and ETS associations of minimal regions of somatic copy number alteration.
(XLSX)

S7 Table Genes with mutations in extended MRA, GISTIC, and homozygous loss regions. Possible haploinsufficiency targets are identified as genes where there are at least three mutations and a normal allele retained.
(XLSX)

S8 Table Mutations in lncRNAs and conserved RNAs located within the minimal regions of somatic copy number alteration. lncRNAs were examined that were defined by the MiTranscriptome project as being either cancer-associated or containing conserved regions.
(XLSX)

S9 Table Mutations in promoter regions of genes within the minimal regions of alteration. (DOCX)

S10 Table Mutations in DNA High-occupancy target (HOT) regions. (XLSX)

S11 Table GISTIC regions of deletion and amplification. q-values: The q-value of the peak region. Residual q-values: The q-value of the peak region after removing (“peeling off”) amplifications or deletions that overlap other, more significant peak regions in the same chromosome. Wide Peak Limits: The “wide peak” boundaries most likely to contain the targeted genes. These are listed in genomic coordinates and marker (or probe) indices.
(XLSX)

S12 Table List of regions of homozygous loss that occur in greater than two patients. (DOCX)

S13 Table Mutations in conserved DNA sequences within the minimal regions of somatic copy number alteration. (XLSX)

S14 Table Structural rearrangements in regions of deletion involving genes found to be in or close to minimal regions of alteration. (XLSX)

S15 Table Structural rearrangements in regions of deletion involving genes found to be in or close to GISTIC detected regions. (XLSX)

S16 Table Pathway enrichment analysis of ETS positive and negative cancers using mutated genes in significant regions of amplification and deletion. The analysis was performed using the Reactome plugin for analysis of canonical pathways in Cytoscape. Only significant (FDR calculated using the Benjamini-Hochberg procedure *p* < 0.05) enriched pathways with potential involvement in cancer are listed.
(XLSX)

S17 Table Summary table of the GISTIC detected deletions that follow the Knudson hit model. (DOCX)

S18 Table Summary of cases and copy number platforms. (DOCX)

S1 Appendix Example copy number plots (BAF and logR) for four tumour samples (and associated controls) for each of the 24 novel MCRs that we have identified. The black lines indicate the segment detected by ASCAT and the blue lines indicate the MCR region.
(PDF)

S2 Appendix An example statistical consideration of the Knudson 2-hit model. (PDF)

S3 Appendix Membership of the CRUK-ICGC prostate group. (PDF)
