# Supplementary figures and images for: Appraising the relevance of DNA copy number loss and gain in prostate cancer using whole genome DNA sequence data
Source: PLoS Genet. 2017 Sep 25;13(9):e1007001. doi: 10.1371/journal.pgen.1007001 (PMC5628936; doi:10.1371/journal.pgen.1007001)

**S1 Fig**

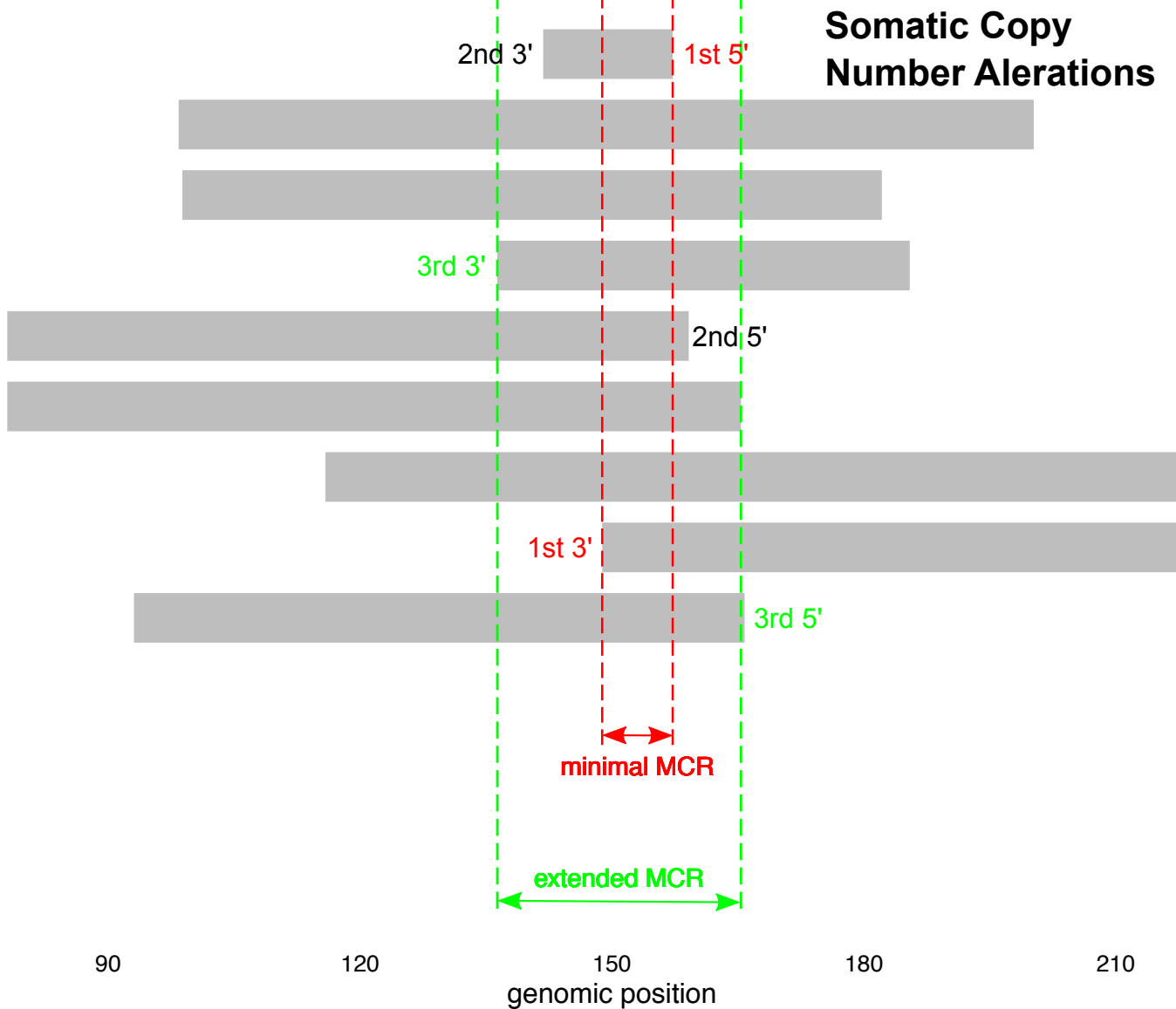

Supplement: S1 Fig — (PDF) [file pgen.1007001.s001.pdf]

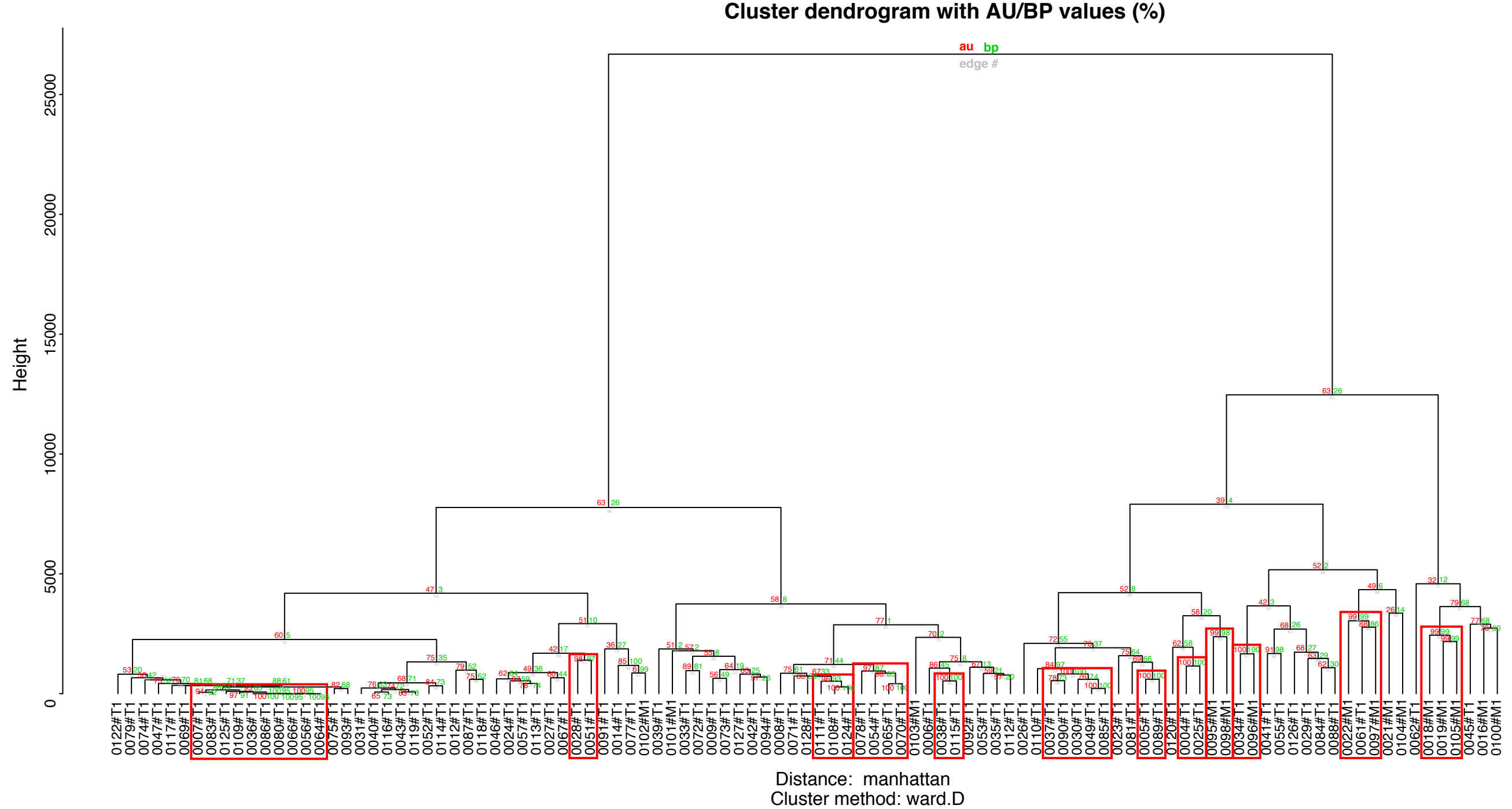

Supplement: S2 Fig — The data are binary values corresponding to the presence/absence (1/0) of regions of copy number gain and loss in each of the tumour samples. p-values were calculated via hierarchical cluster analysis with multiscale bootstrap resampling of 1000 using Ward’s method and the Manhattan distance. The analysis was performed using the pvclust package in R. Values at branches are AU (Approximately Unbiased) p-values (left, red), and BP (Bootstrap Probability) values (right, green). Clusters significantly supported by the data (AU ≥ 95) are indicated by the red rectangles. (PDF) [file pgen.1007001.s002.pdf]

S4 Fig

a

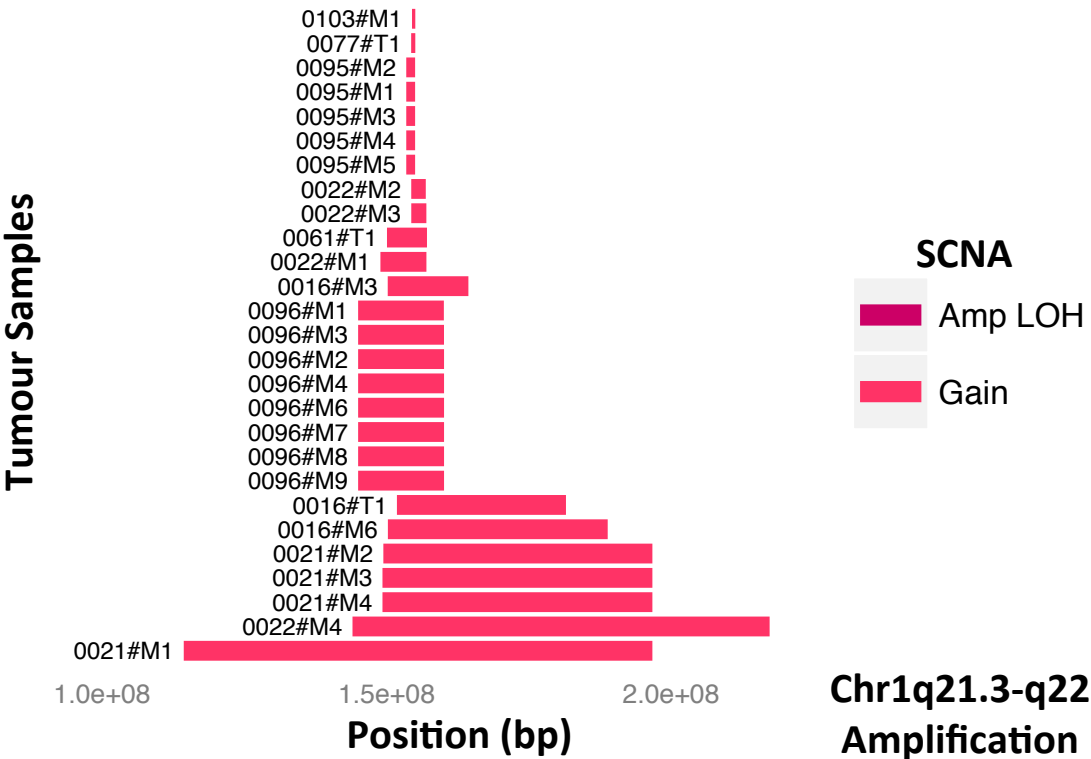

b

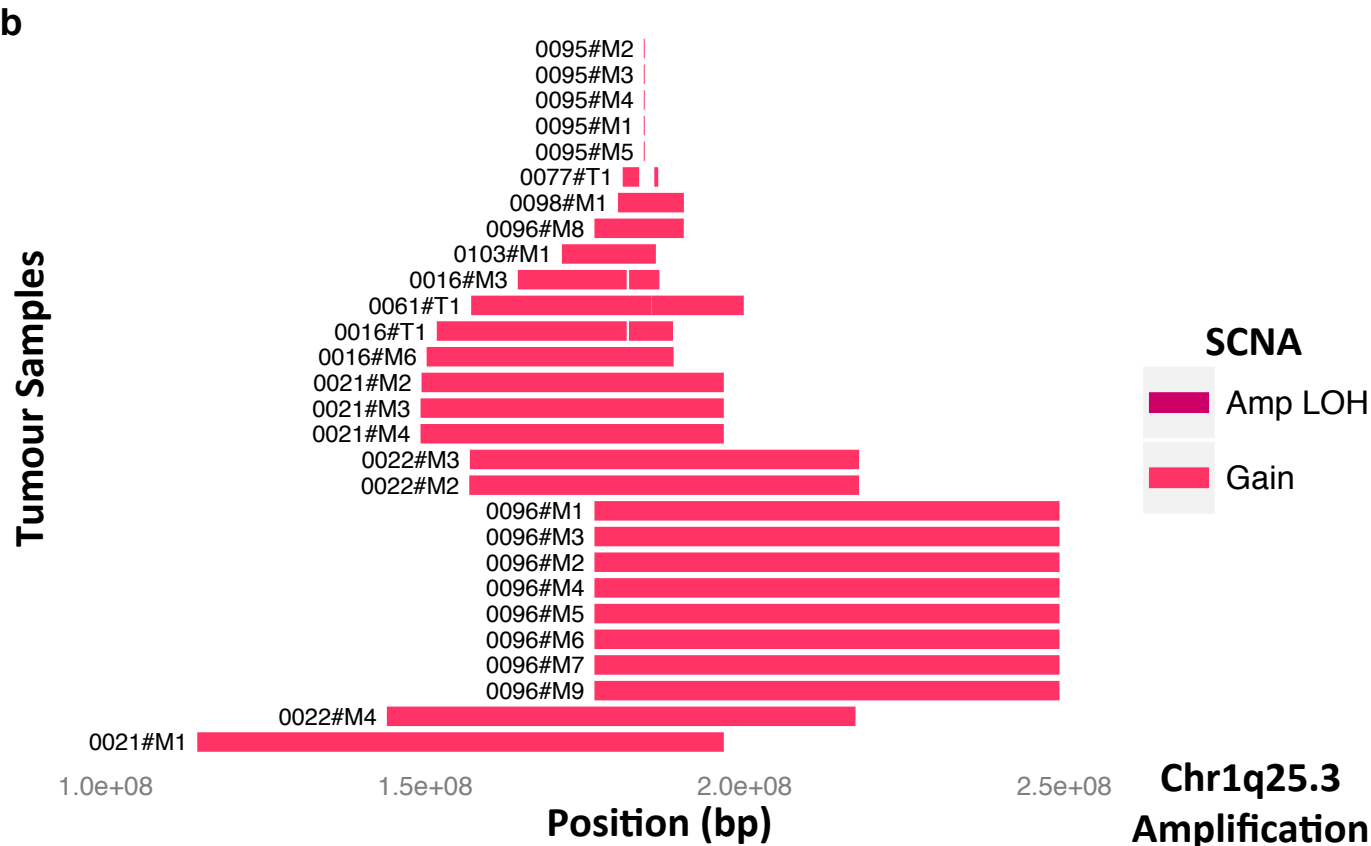

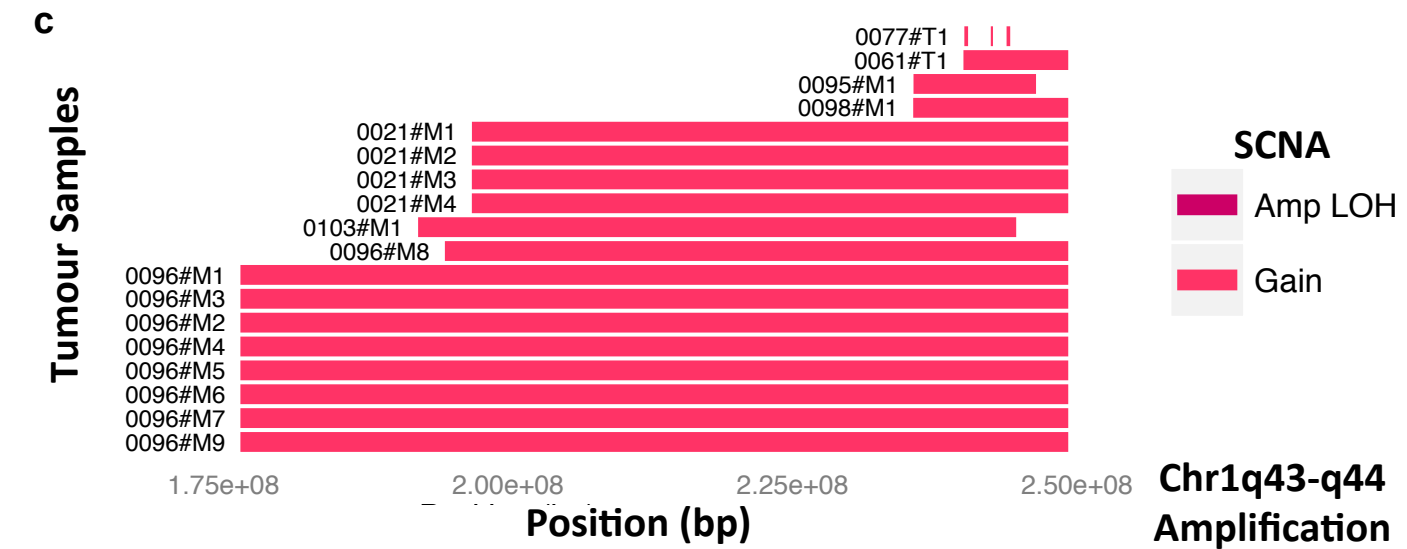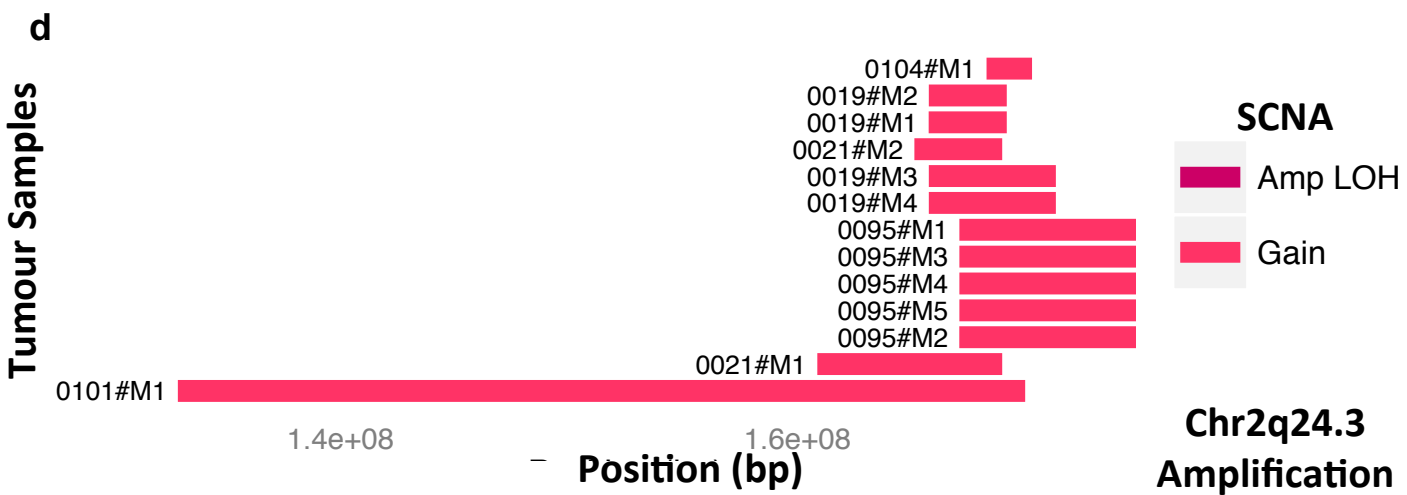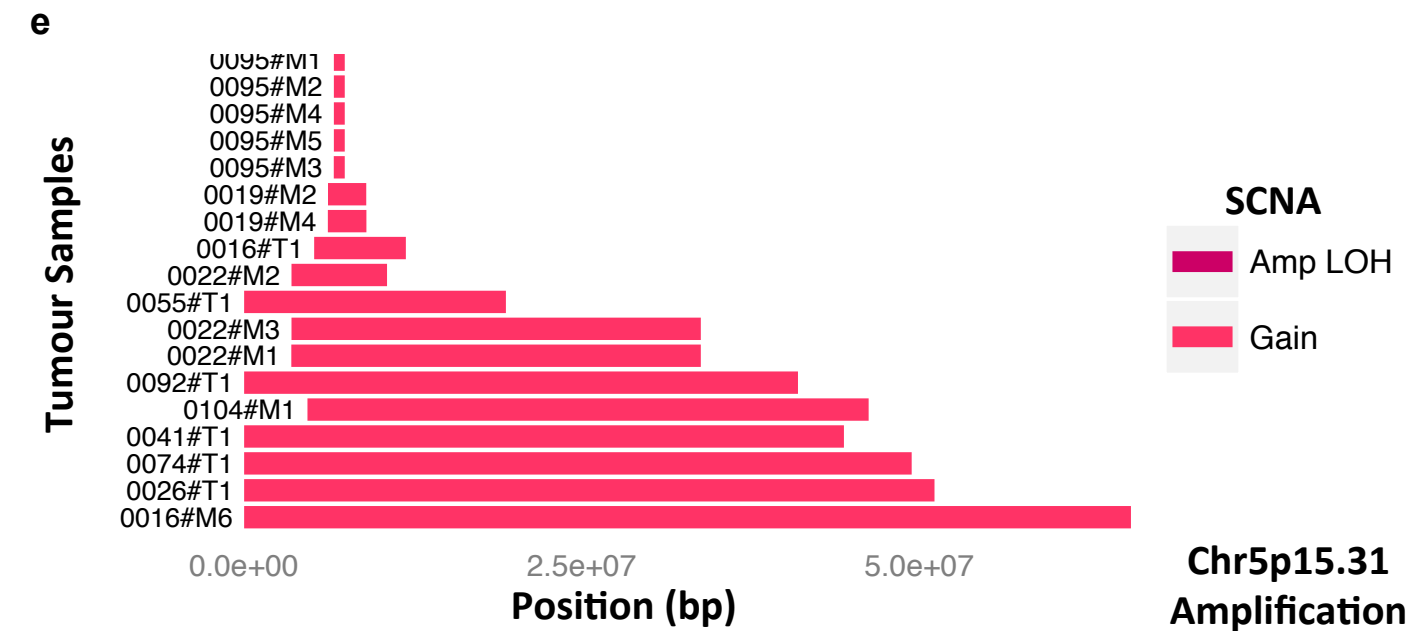

f

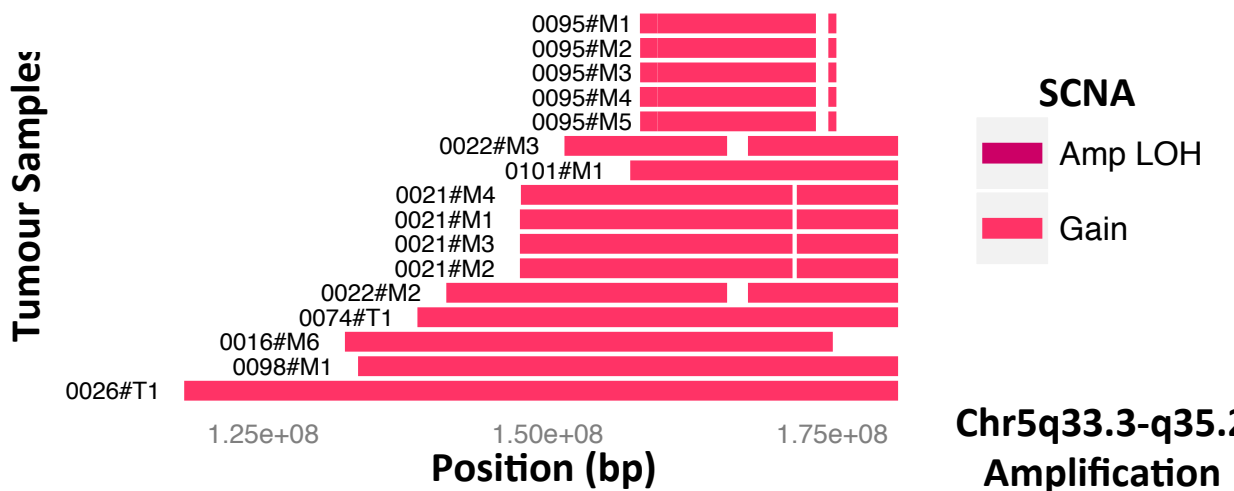

g

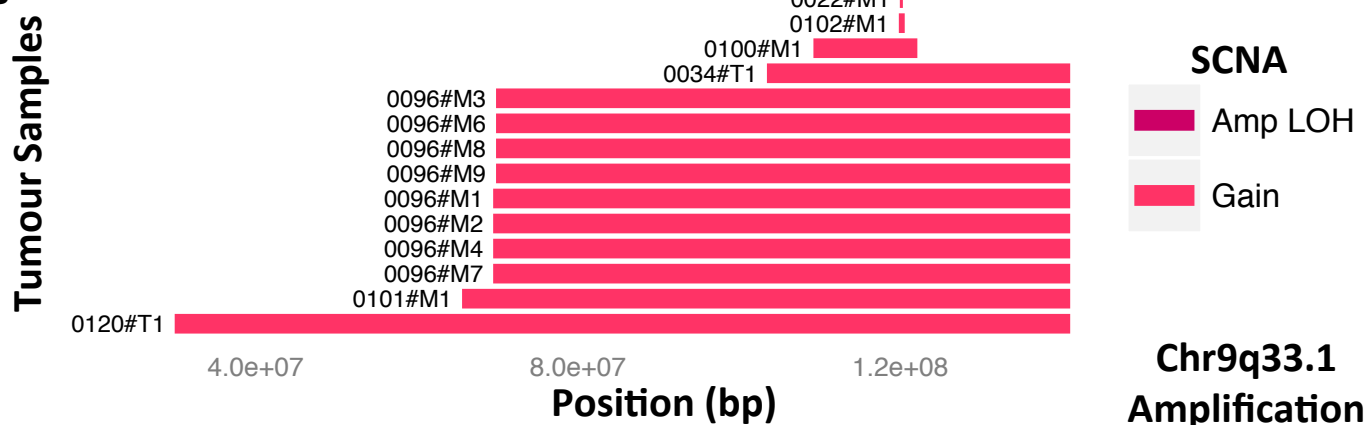

h

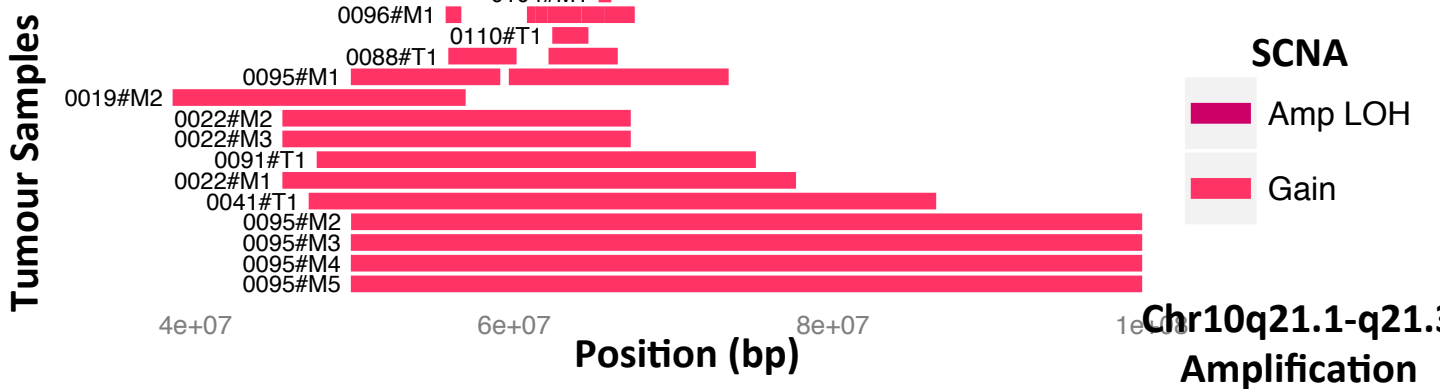

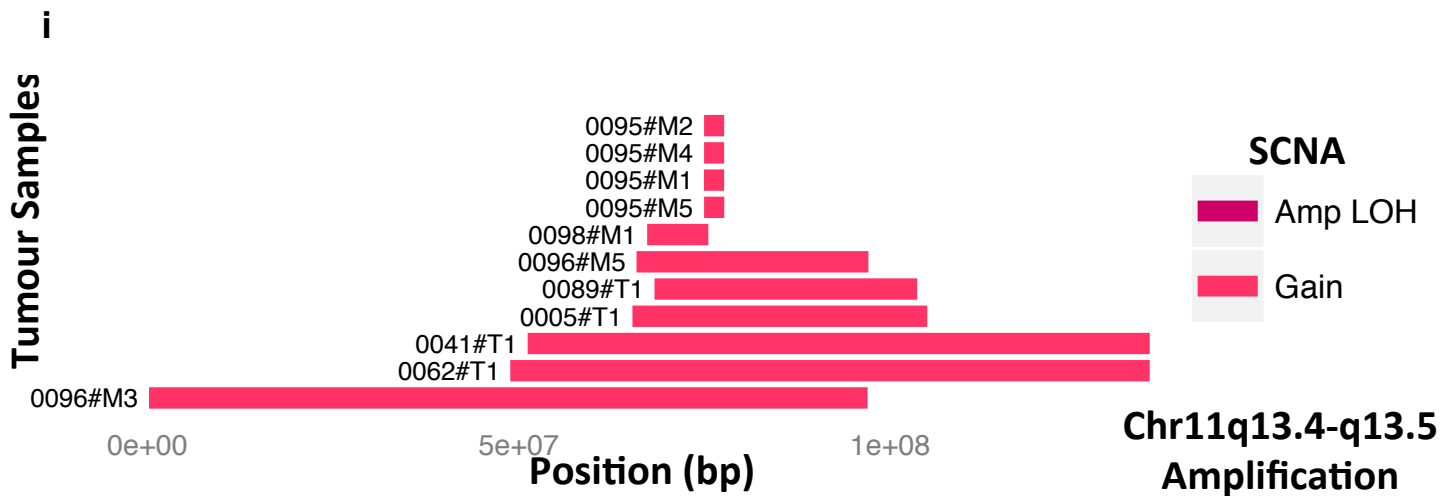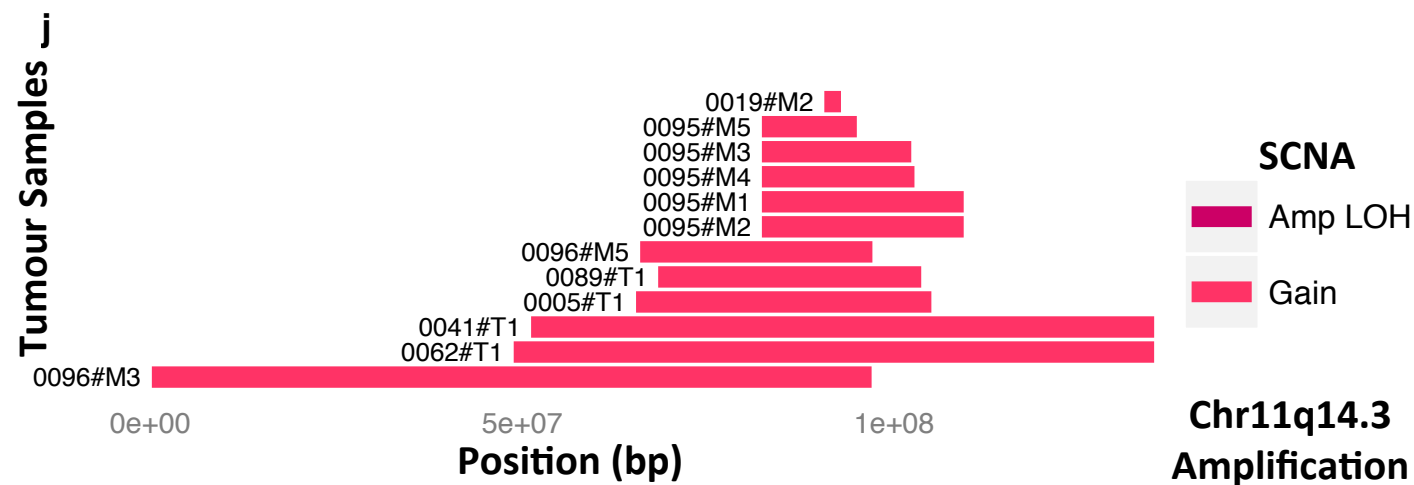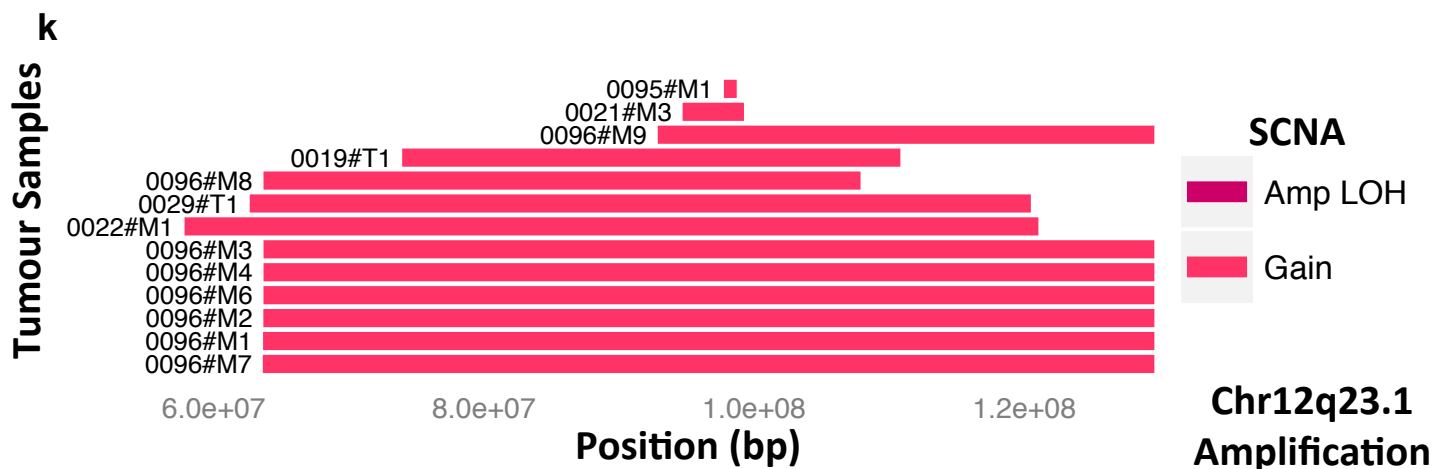

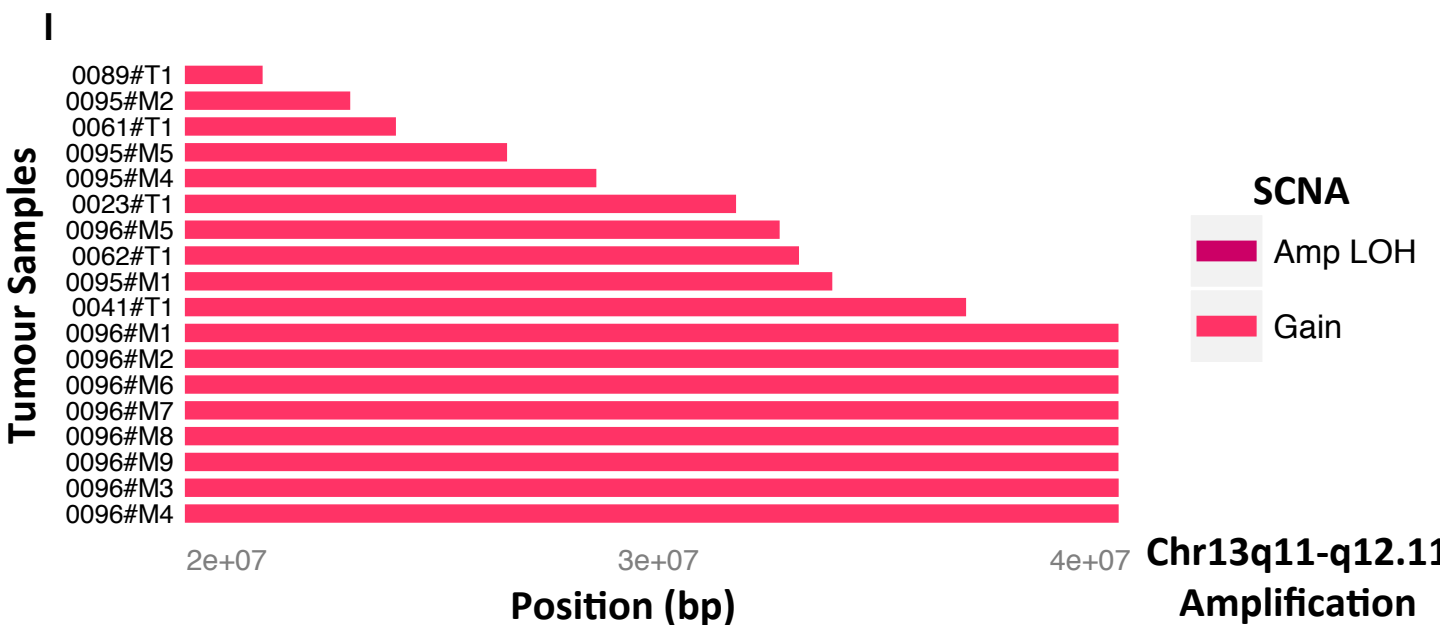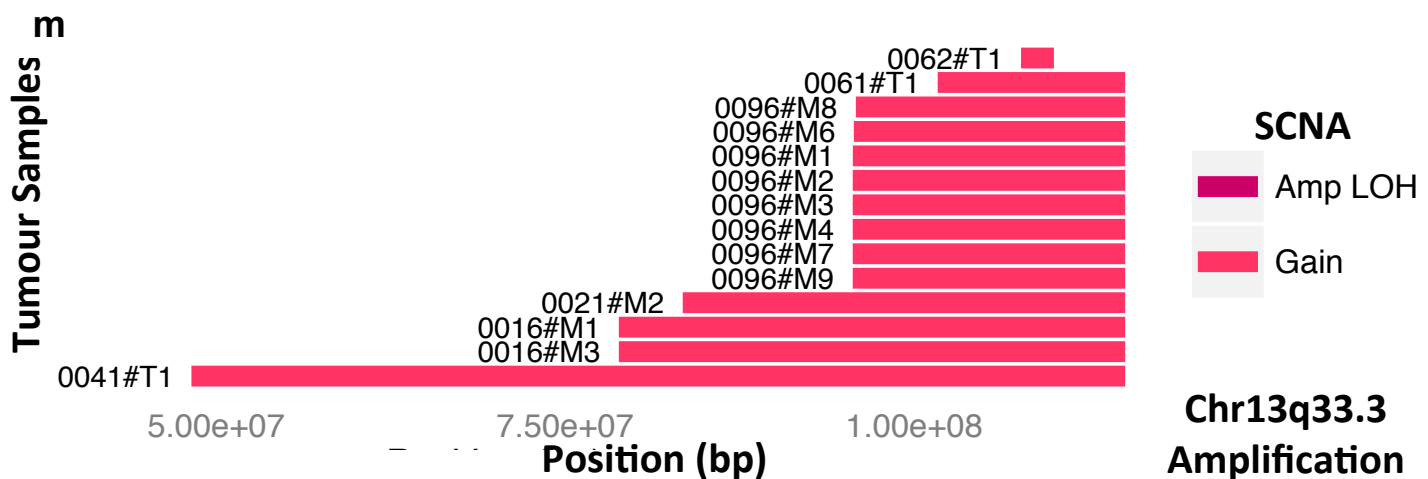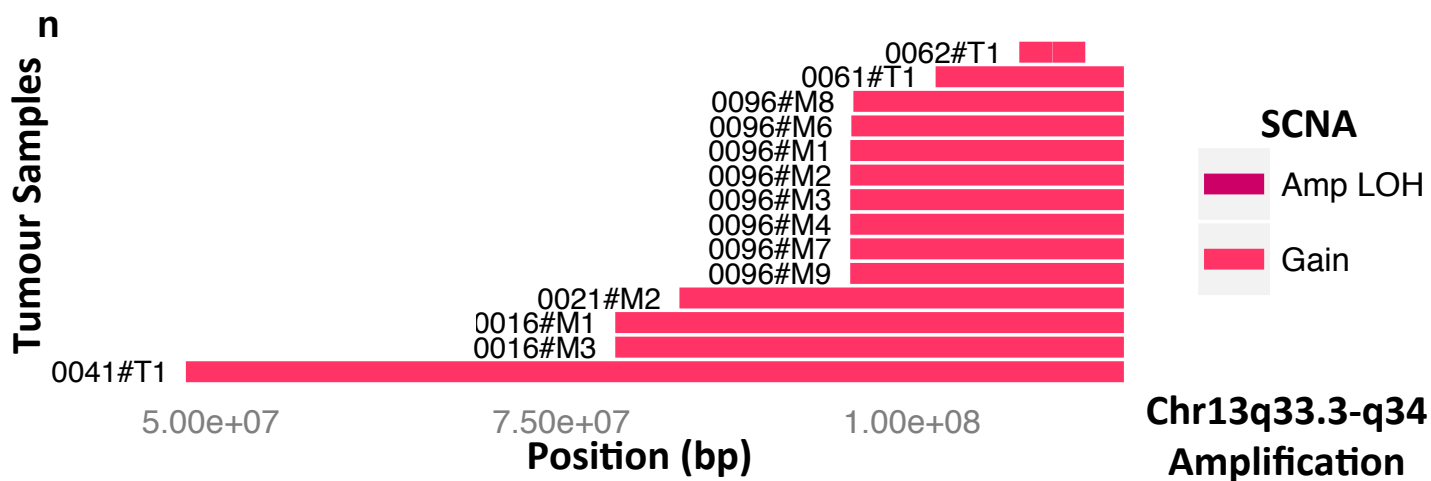

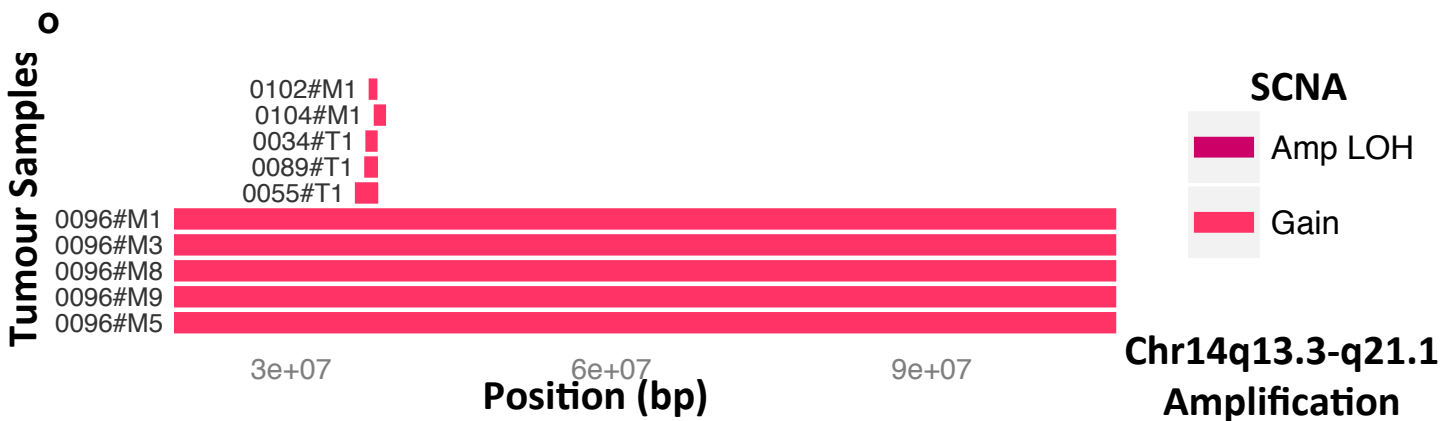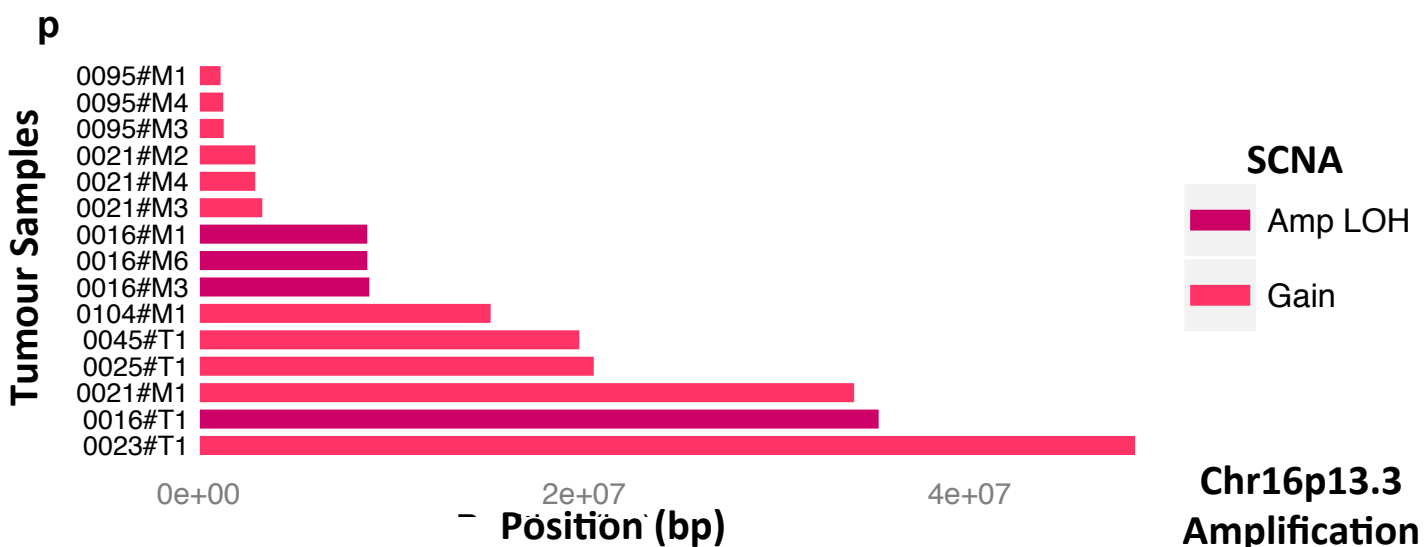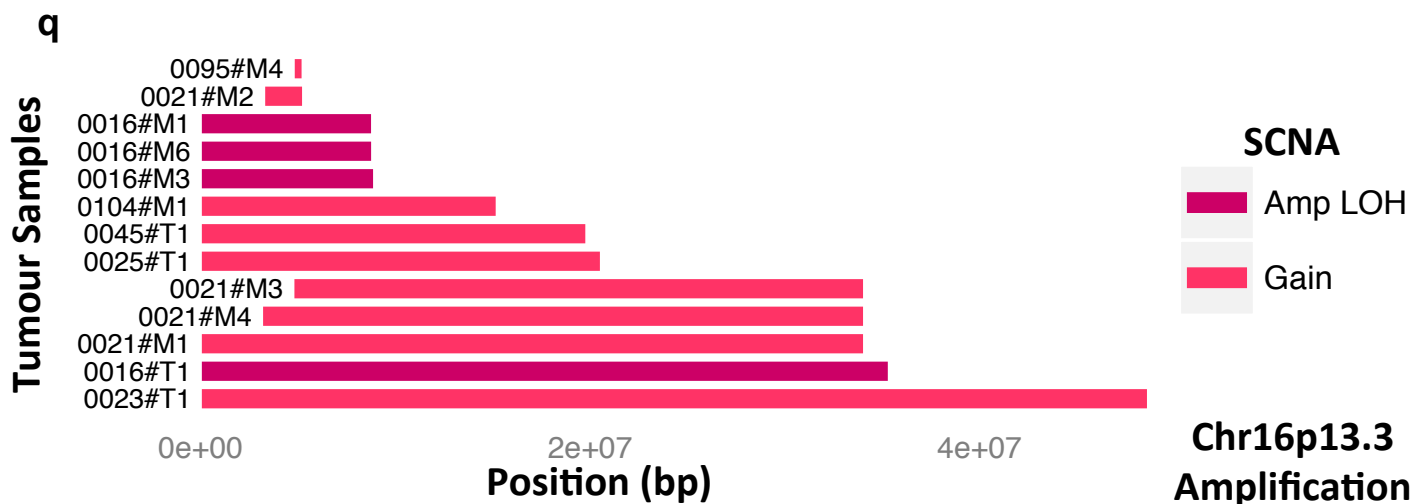

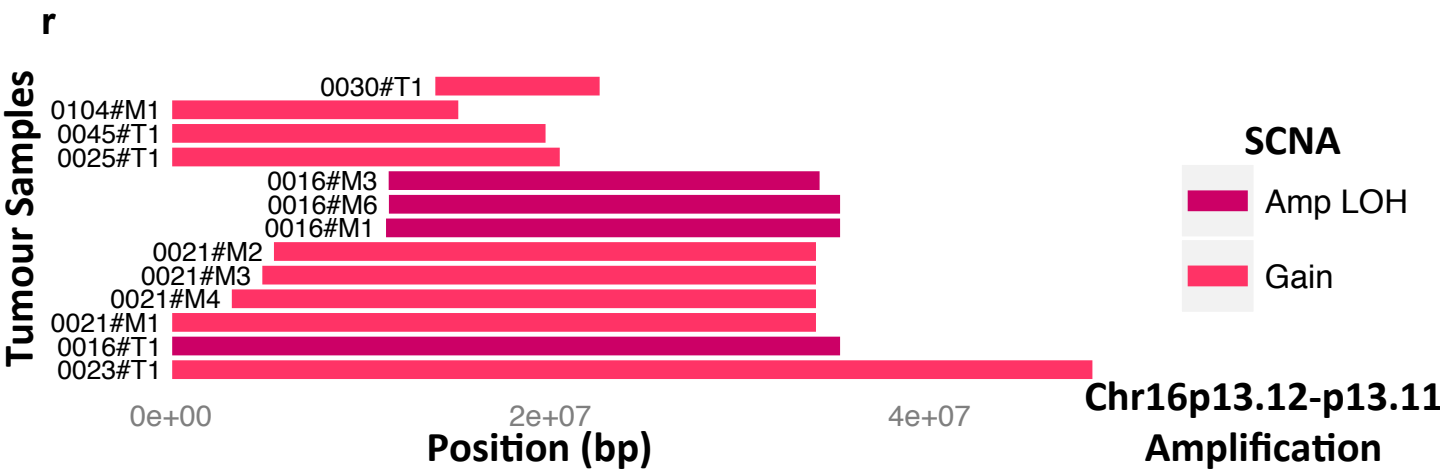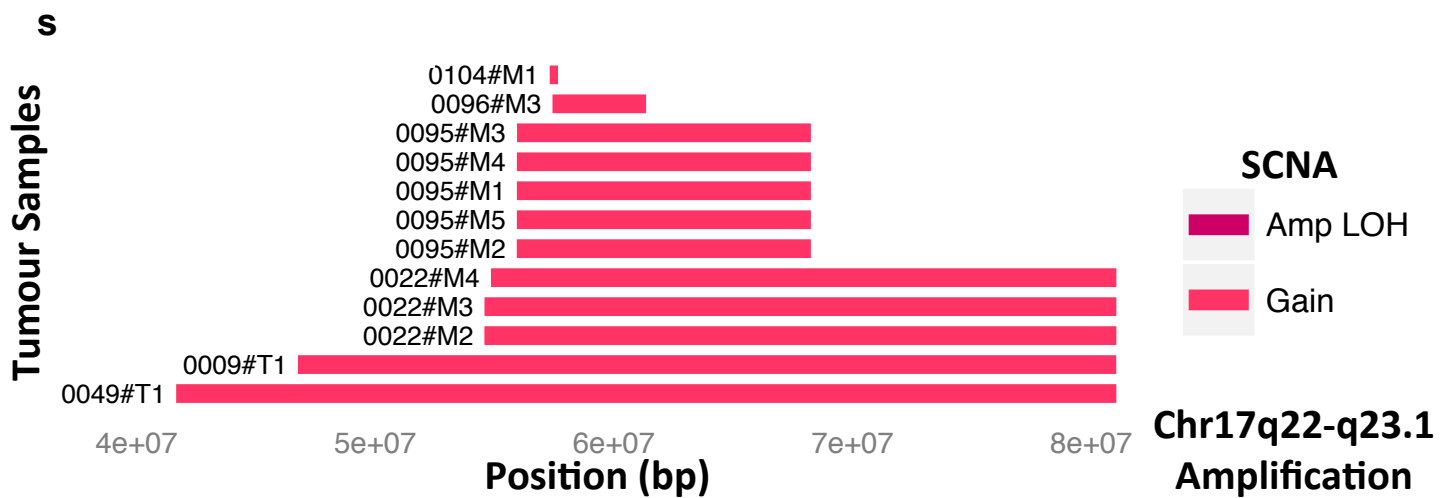

t

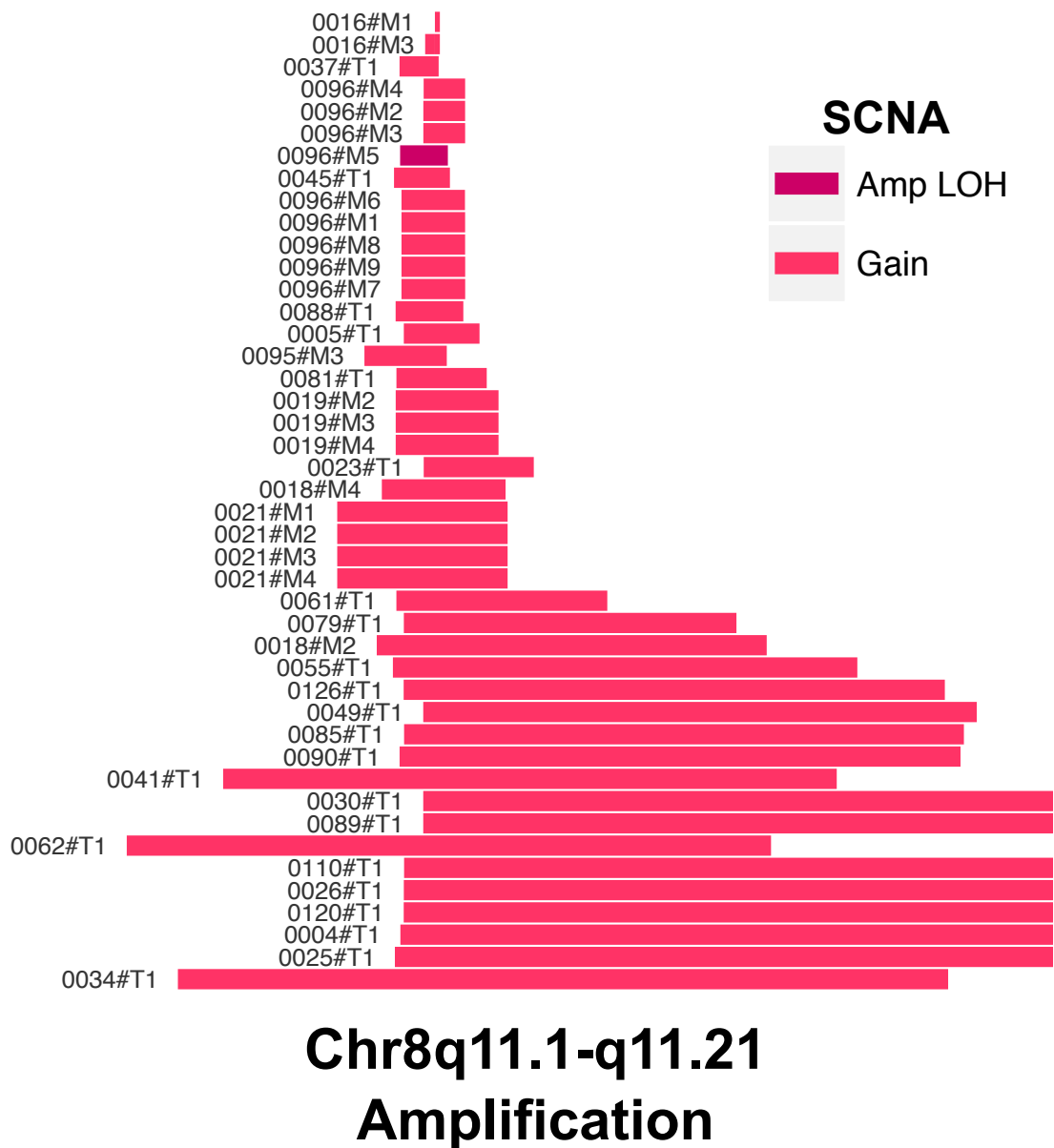

u

Tumour Samples

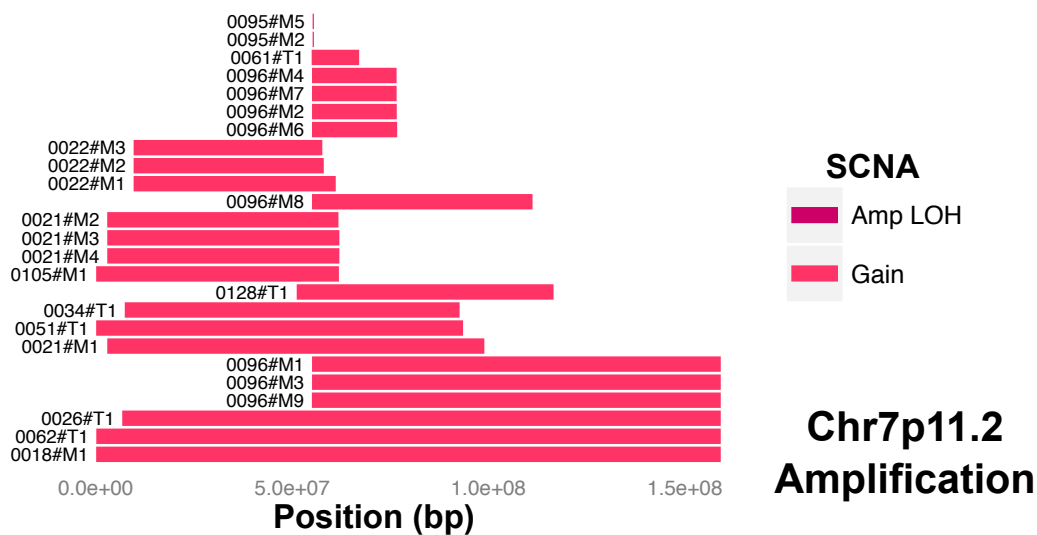

v

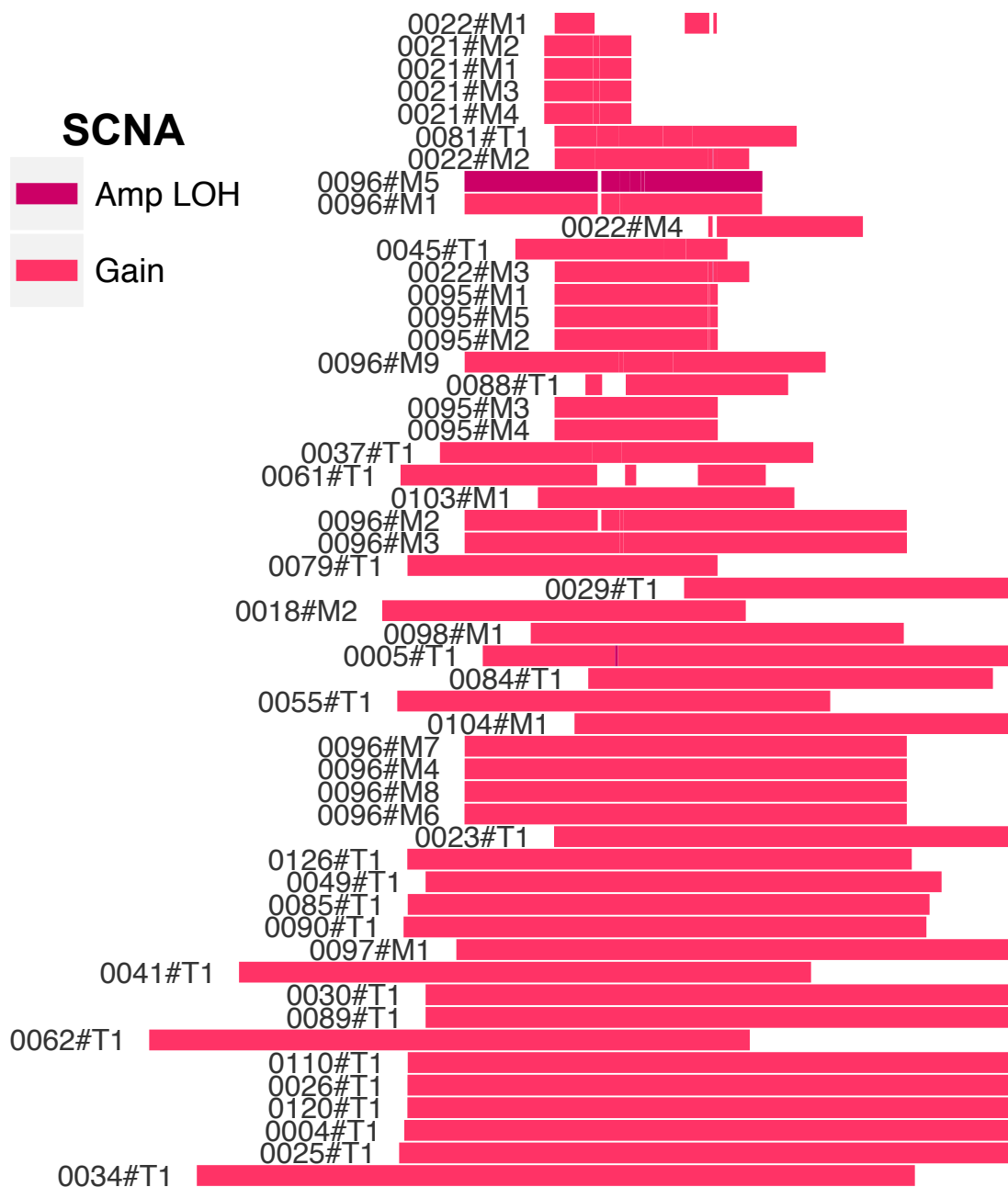

**Chr8q21.1-q12.1  
Amplification**

W

Chr8q24.21  
Amplification

SCNA

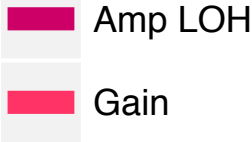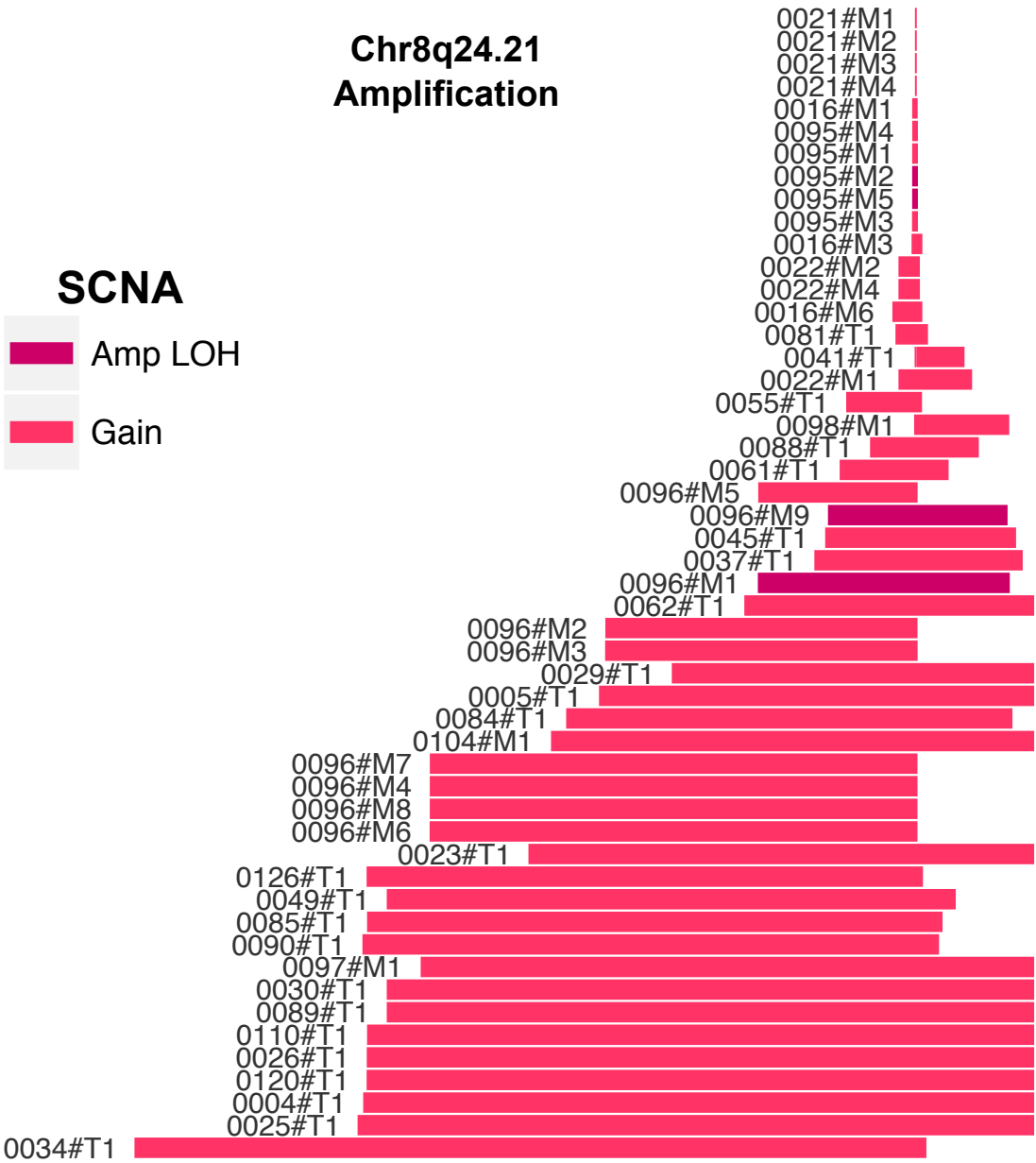

X

Tumour Samples

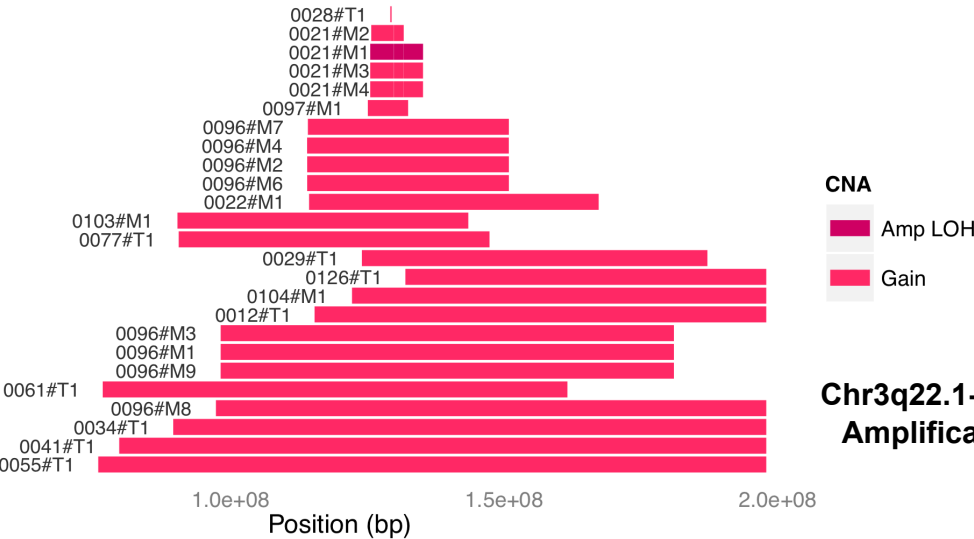

Supplement: S4 Fig — The chromosome gain events are represented with distinct colour blocks depending on the type of SCNA: gain (any gain in the number of normal allele copies) and amplification LOH (loss of one allele with any gain of the remaining allele). The regions of SCNA are ordered by length: top-smallest, bottom-largest. Each block has been labelled with sample ID. Cases for which more than one sample was available are all indicated, however contribution to the frequency of the SCNA was defined on a per patient basis. (a) chr1 q21.3-q22, (b) chr1 q25.3, (c) chr1 q43-q44, (d) chr2 q24.3, (e) chr5 p15.31, (f) chr5 q33.3-q35.2, (g) chr9 q33.1, (h) chr10 q21.1-q21.3, (i) chr11 q13.4-q13.5, (j) chr11 q14.3, (k) chr12 q23.1, (l) chr13 q11-q12.11, (m) chr13 q33.3, (n) chr13 q33.3-q34, (o) chr14 q13.3-q21.1, (p) chr16 p13.3, (q) chr16 p13.3, (r) chr16 p13.12-p13.11, (s) chr17 q22-q23.1, (t) Chr8q11.1-q11.21, (u) Chr7p11.2, (v) Chr8q21.1-q12.1, (w) Chr8q24.21, (x) Chr3q22.1-q21.3. (PDF) [file pgen.1007001.s004.pdf]

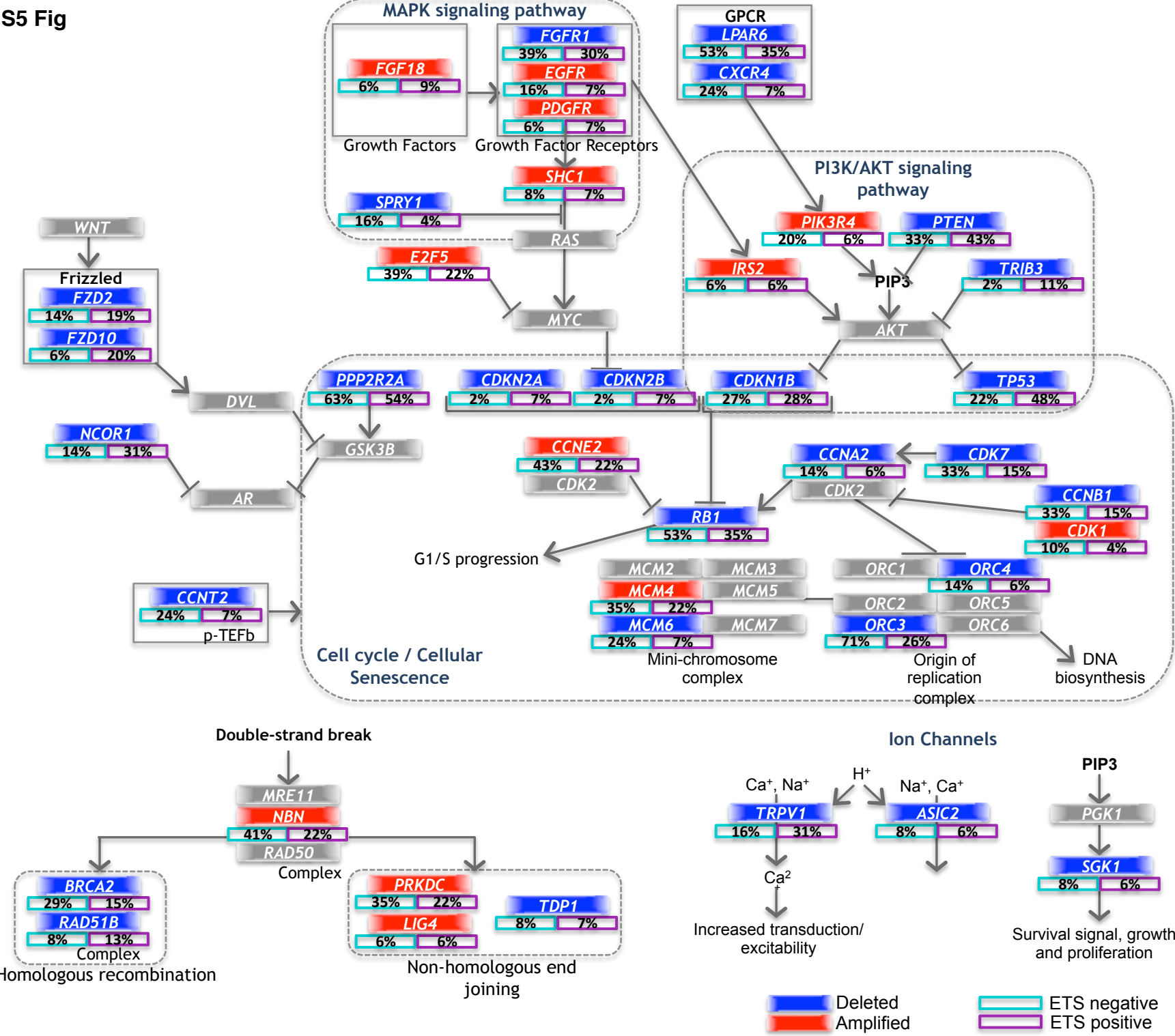

Supplement: S5 Fig — Blue and red blocks indicate genes contained in regions of deletion and amplification respectively. Grey blocks indicate genes with no alteration that were required for representation of the pathway. Purple and blue squares indicate the percentage of samples with a copy number alteration in that gene in ETS negative and positive samples. (PDF) [file pgen.1007001.s005.pdf]

S6 Fig  
a

ASCAT 2.2 on Affymetrix SNP6.0 data

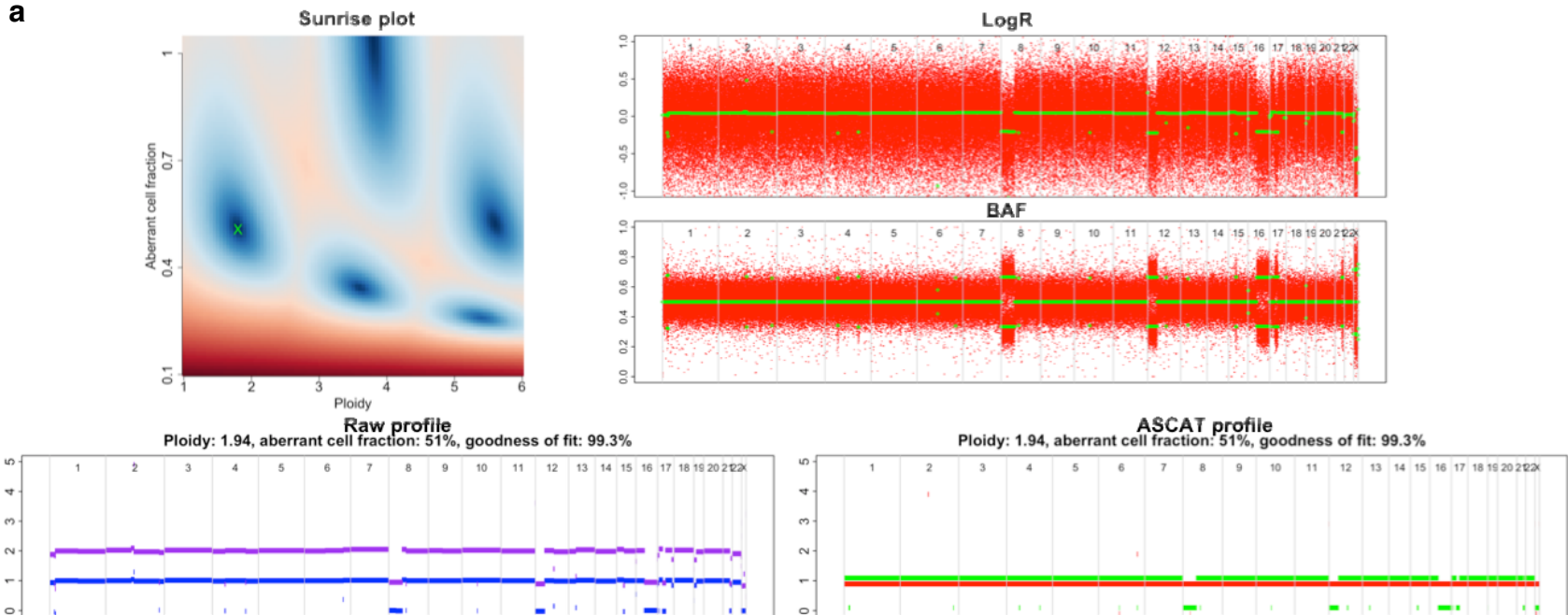

b

ASCAT 2.2 on NGS-SNP6.0 data

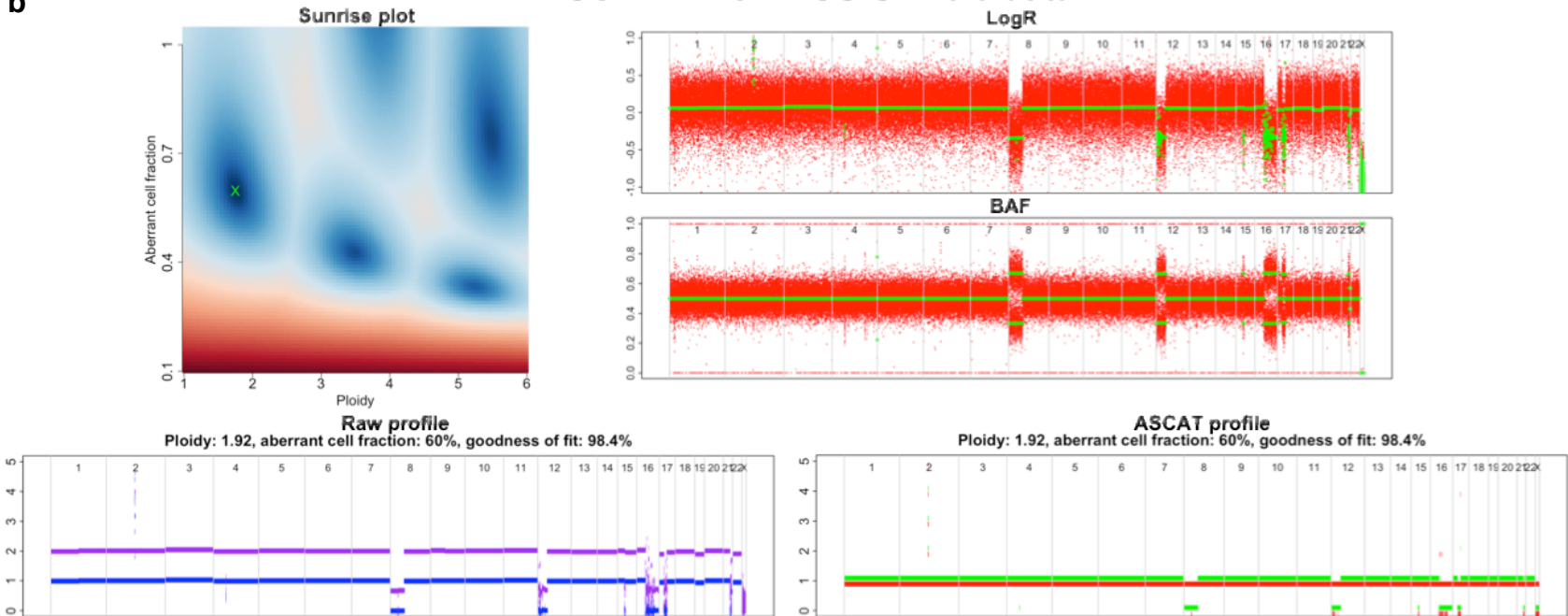

Supplement: S6 Fig — (a) one profile from SNP6.0 and (b) one from NGS data. (PDF) [file pgen.1007001.s006.pdf]
