# Supplementary material for: Appraising the relevance of DNA copy number loss and gain in prostate cancer using whole genome DNA sequence data
Source: PLoS Genet. 2017 Sep 25;13(9):e1007001. doi: 10.1371/journal.pgen.1007001 (PMC5628936; doi:10.1371/journal.pgen.1007001)
Supplement: S3 Fig — Each deletion is represented as a distinct colour as shown in the key. Deletions are as follows: neutral LOH (loss of one allele with duplication of the remaining allele); hemizygous deletion LOH (loss of one allele); homozygous loss (loss of the two alleles); and other loss (loss of one allele copy following whole genome duplication). Cases for which more than one sample was available are all indicated, however contribution to the frequency of the SCNA was defined on a per patient basis. The regions of SCNA are ordered by length: top-smallest, bottom-largest. Each block has been labelled with sample ID. (a) chr1 p31.1, (b) chr1 q42.2-q42.3, (c) chr2 q21.3-q22.1, (d) chr3 p13, (e) chr4 p15.2-p15.1, (f) chr4 q22.3, (g) chr4 q27-q28.1, (h) chr4 q34.3, (i) chr5 q13.1-q13.2, (j) chr7 q31.32-q31.33, (k) chr9 p22.3, (l) chr11 p13, (m) chr11 q23.2, (n) chr12 p13.1, (o) chr12 q24.33, (p) chr14 q24.1, (q) chr14 q32.13, (r) chr15 q21.3, (s) chr16 q23.1-q24.3, (t) chr17 p13.1, (u) chr18 p11.32-p11.31, (v) chr18 q12.3, (w) chr18 q23-q22.3, (x) chr19 p12, (y) chr19 q13.31, (z) chr20 p13, (a2) chr20 p12.1, (b2) chr20 q13.33, (c2) chr21 q22.2, (d2) chr21 q22.3, (e2) chr22 q12.1-q12.2, (f2) chr22 q13.31, (g2) Chr6q14.3-q15, (h2) Chr8p21.3-p21.2, (i2) Chr10q23.31, (j2) Chr13q14.13, (k 2) Chr17q21.3, (l2) Chr5q21.1, (m2) Chr6p25.1-p24.3, (n2) Chr6q27. (PDF) [file pgen.1007001.s003.pdf]

S3 Fig

a

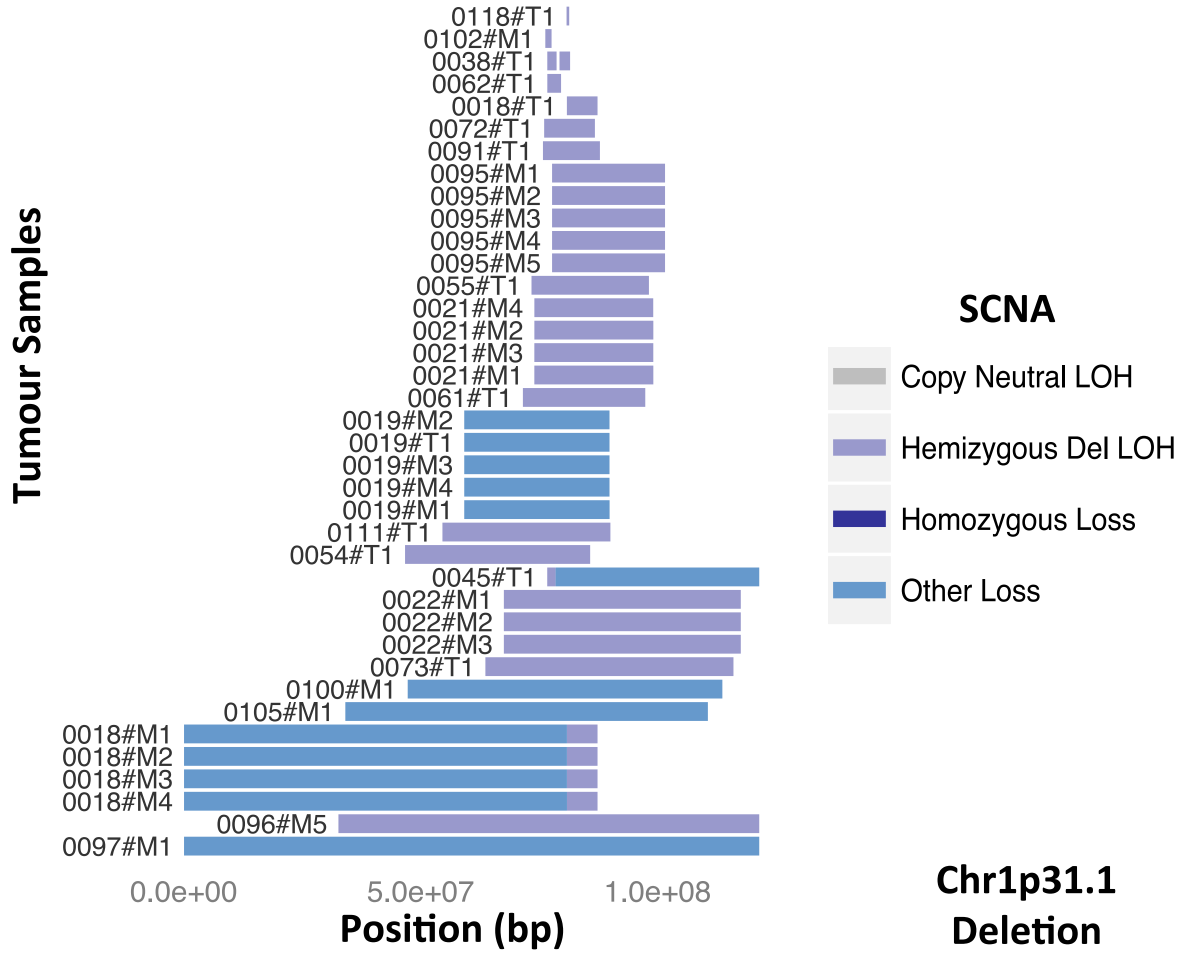
0118#T1
0102#M1
0038#T1
0062#T1
0018#T1
0072#T1
0091#T1
0095#M1
0095#M2
0095#M3
0095#M4
0095#M5
0055#T1
0021#M4
0021#M2
0021#M3
0021#M1
0061#T1
0019#M2
0019#T1
0019#M3
0019#M4
0019#M1
0111#T1
0054#T1
0045#T1
0022#M1
0022#M2
0022#M3
0073#T1
0100#M1
0105#M1
0018#M1
0018#M2
0018#M3
0018#M4
0096#M5
0097#M1
Tumour Samples
0.0e+00
5.0e+07
1.0e+08
Position (bp)
SCNA
Copy Neutral LOH
Hemizygous Del LOH
Homozygous Loss
Other Loss
Chr1p31.1
Deletion

b

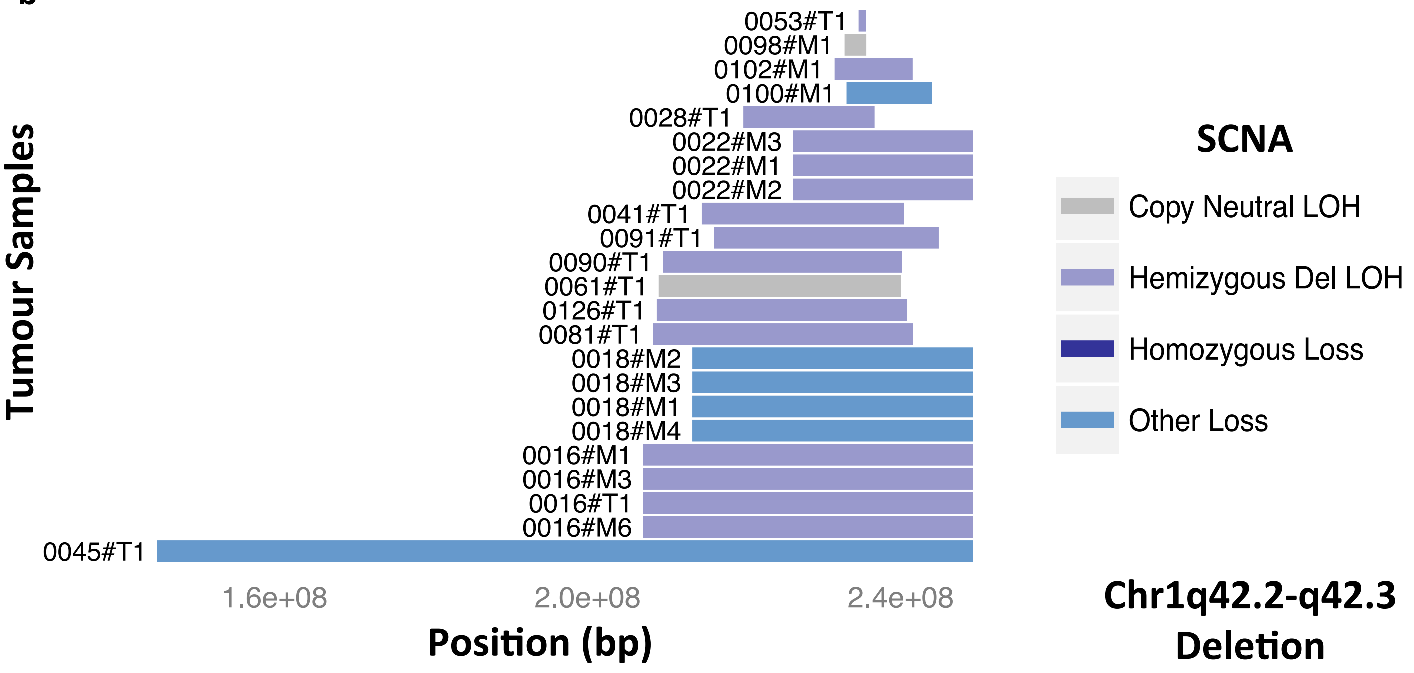
0053#T1
0098#M1
0102#M1
0100#M1
0028#T1
0022#M3
0022#M1
0022#M2
0041#T1
0091#T1
0090#T1
0061#T1
0126#T1
0081#T1
0018#M2
0018#M3
0018#M1
0018#M4
0016#M1
0016#M3
0016#T1
0016#M6
0045#T1
Tumour Samples
1.6e+08
2.0e+08
2.4e+08
Position (bp)
SCNA
Copy Neutral LOH
Hemizygous Del LOH
Homozygous Loss
Other Loss
Chr1q42.2-q42.3
Deletion

c

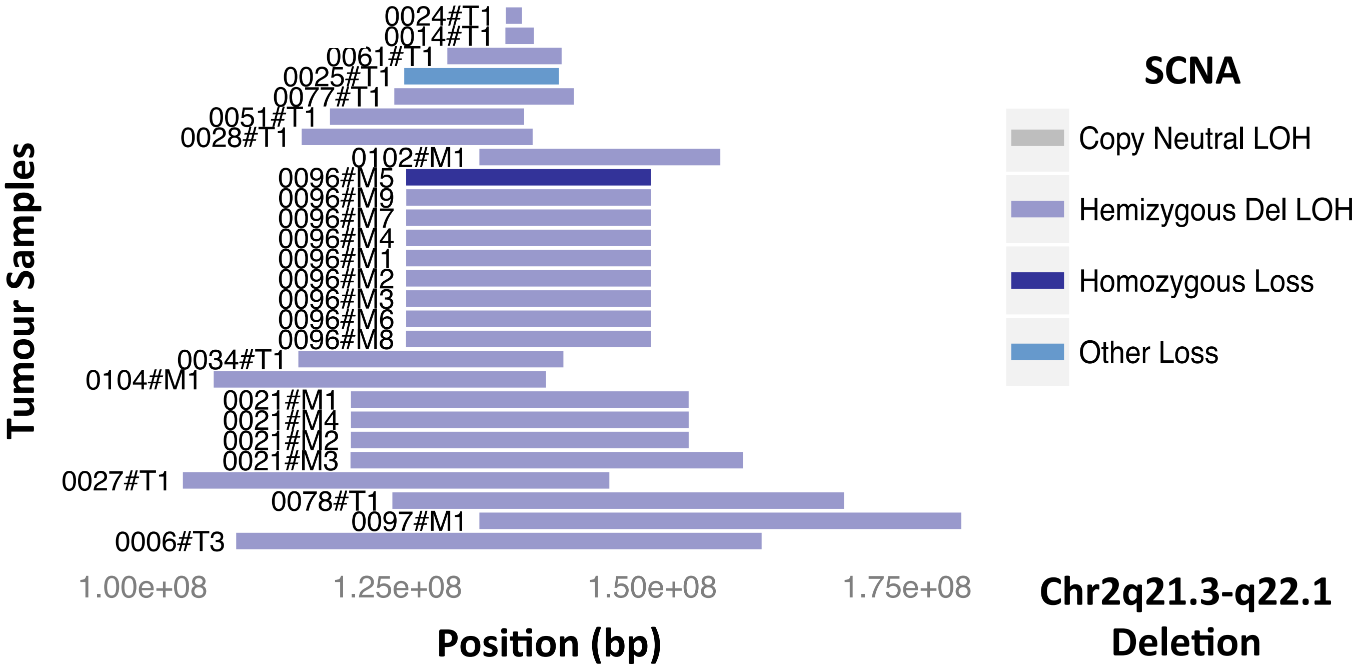

0024#T1
0014#T1
0061#T1
0025#T1
0077#T1
0051#T1
0028#T1
0102#M1
0096#M5
0096#M9
0096#M7
0096#M4
0096#M1
0096#M2
0096#M3
0096#M6
0096#M8
0034#T1
0104#M1
0021#M1
0021#M4
0021#M2
0021#M3
0027#T1
0078#T1
0097#M1
0006#T3
Tumour Samples
1.00e+08
1.25e+08
1.50e+08
1.75e+08
Position (bp)
SCNA
Copy Neutral LOH
Hemizygous Del LOH
Homozygous Loss
Other Loss
Chr2q21.3-q22.1
Deletion

d

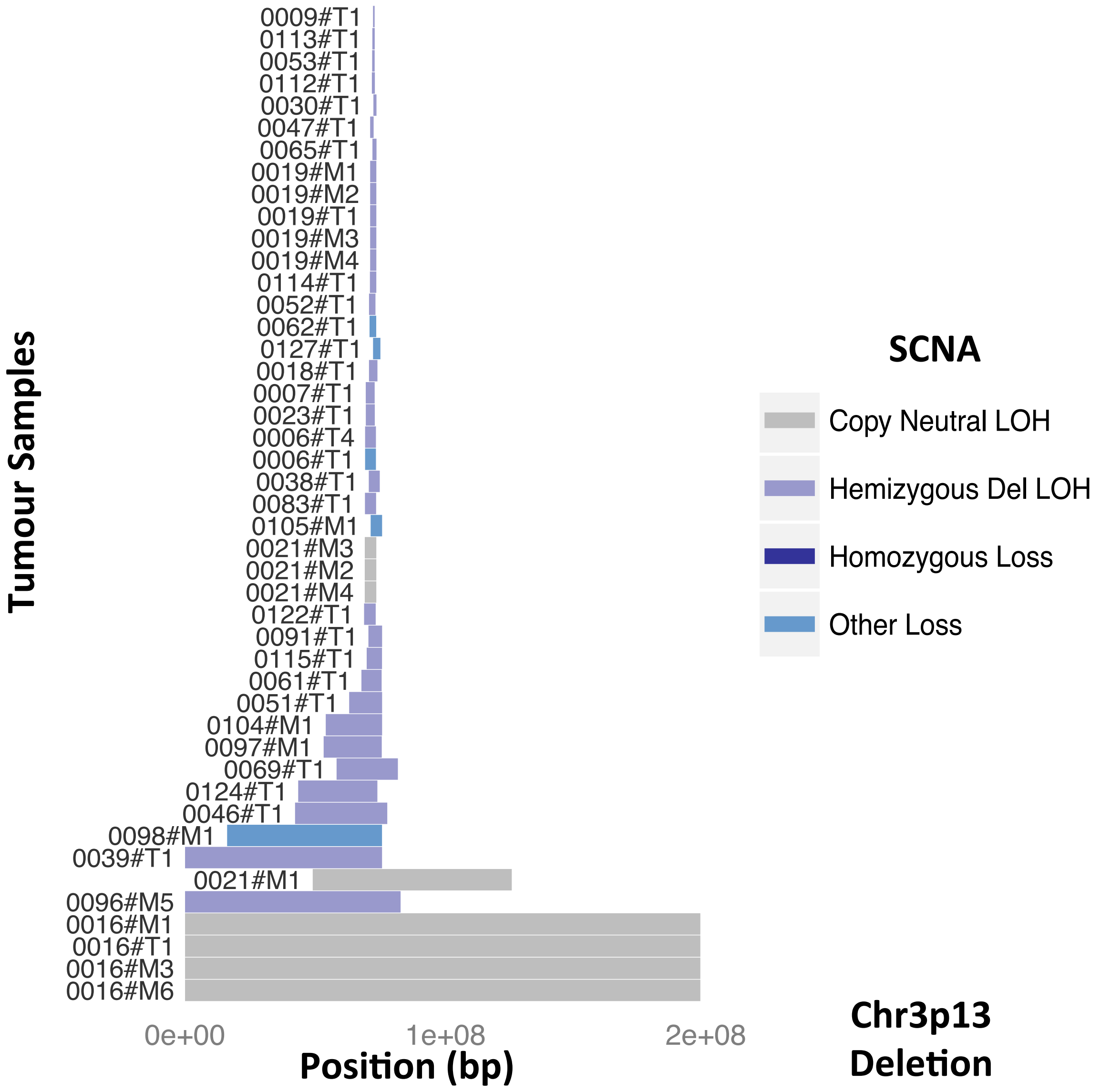

0009#T1
0113#T1
0053#T1
0112#T1
0030#T1
0047#T1
0065#T1
0019#M1
0019#M2
0019#T1
0019#M3
0019#M4
0114#T1
0052#T1
0062#T1
0127#T1
0018#T1
0007#T1
0023#T1
0006#T4
0006#T1
0038#T1
0083#T1
0105#M1
0021#M3
0021#M2
0021#M4
0122#T1
0091#T1
0115#T1
0061#T1
0051#T1
0104#M1
0097#M1
0069#T1
0124#T1
0046#T1
0098#M1
0039#T1
0021#M1
0096#M5
0016#M1
0016#T1
0016#M3
0016#M6
Tumour Samples
0e+00
1e+08
2e+08
Position (bp)
SCNA
Copy Neutral LOH
Hemizygous Del LOH
Homozygous Loss
Other Loss
Chr3p13
Deletion

e

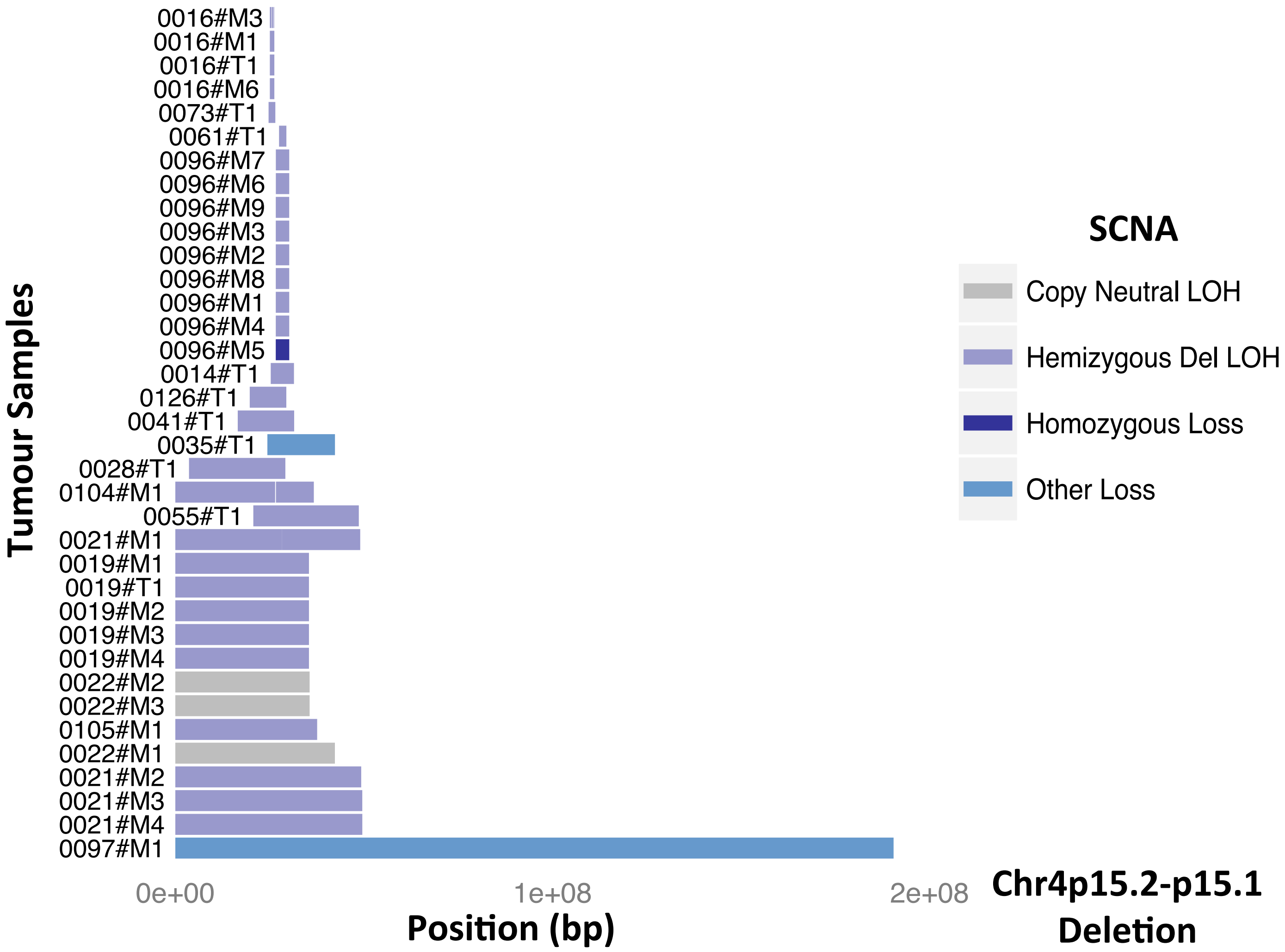

0016#M3
0016#M1
0016#T1
0016#M6
0073#T1
0061#T1
0096#M7
0096#M6
0096#M9
0096#M3
0096#M2
0096#M8
0096#M1
0096#M4
0096#M5
0014#T1
0126#T1
0041#T1
0035#T1
0028#T1
0104#M1
0055#T1
0021#M1
0019#M1
0019#T1
0019#M2
0019#M3
0019#M4
0022#M2
0022#M3
0105#M1
0022#M1
0021#M2
0021#M3
0021#M4
0097#M1
Tumour Samples
0e+00
1e+08
2e+08
Position (bp)
SCNA
Copy Neutral LOH
Hemizygous Del LOH
Homozygous Loss
Other Loss
Chr4p15.2-p15.1
Deletion

f

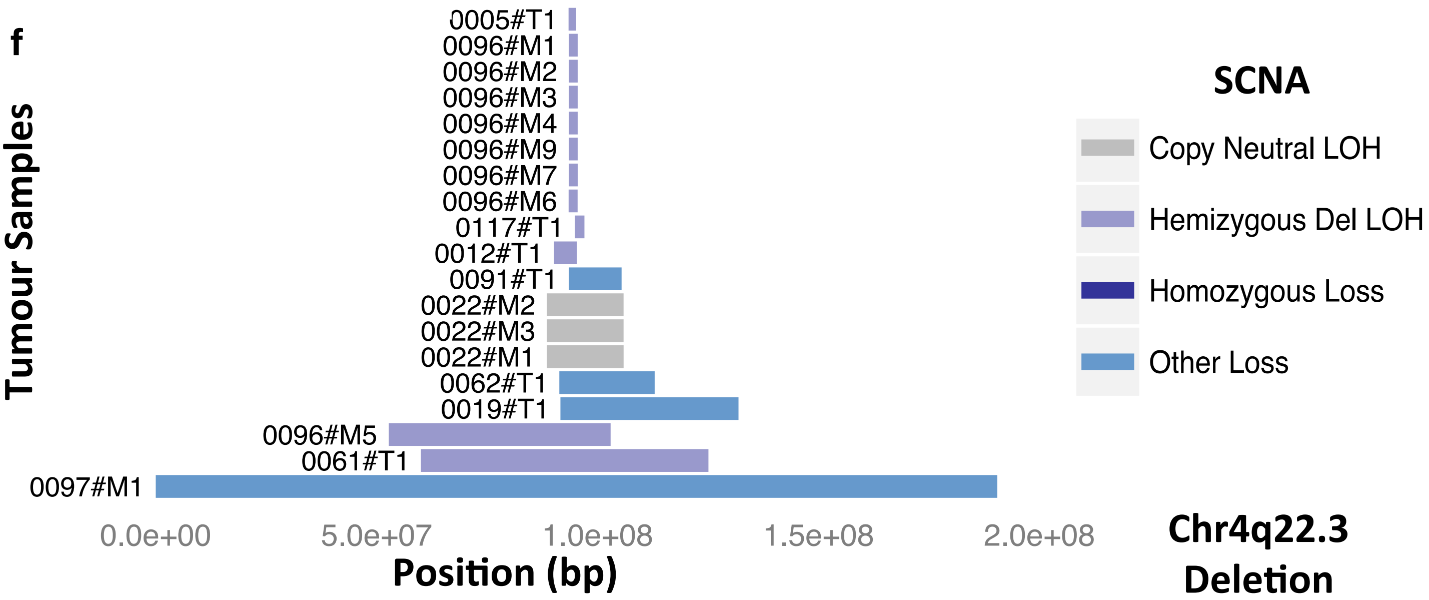

0005#T1
0096#M1
0096#M2
0096#M3
0096#M4
0096#M9
0096#M7
0096#M6
0117#T1
0012#T1
0091#T1
0022#M2
0022#M3
0022#M1
0062#T1
0019#T1
0096#M5
0061#T1
0097#M1
Tumour Samples
0.0e+00
5.0e+07
1.0e+08
1.5e+08
2.0e+08
Position (bp)
SCNA
Copy Neutral LOH
Hemizygous Del LOH
Homozygous Loss
Other Loss
Chr4q22.3
Deletion

g

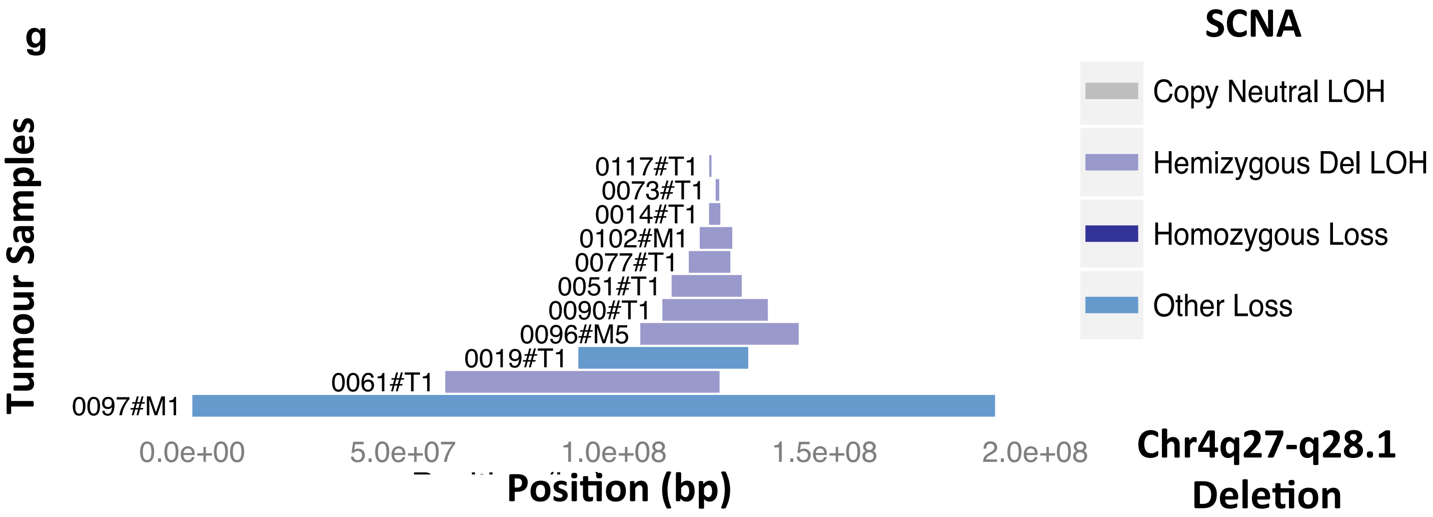
SCNA
Copy Neutral LOH
Hemizygous Del LOH
Homozygous Loss
Other Loss
Tumour Samples
0117#T1
0073#T1
0014#T1
0102#M1
0077#T1
0051#T1
0090#T1
0096#M5
0019#T1
0061#T1
0097#M1
0.0e+00
5.0e+07
1.0e+08
1.5e+08
2.0e+08
Position (bp)
Chr4q27-q28.1
Deletion

h

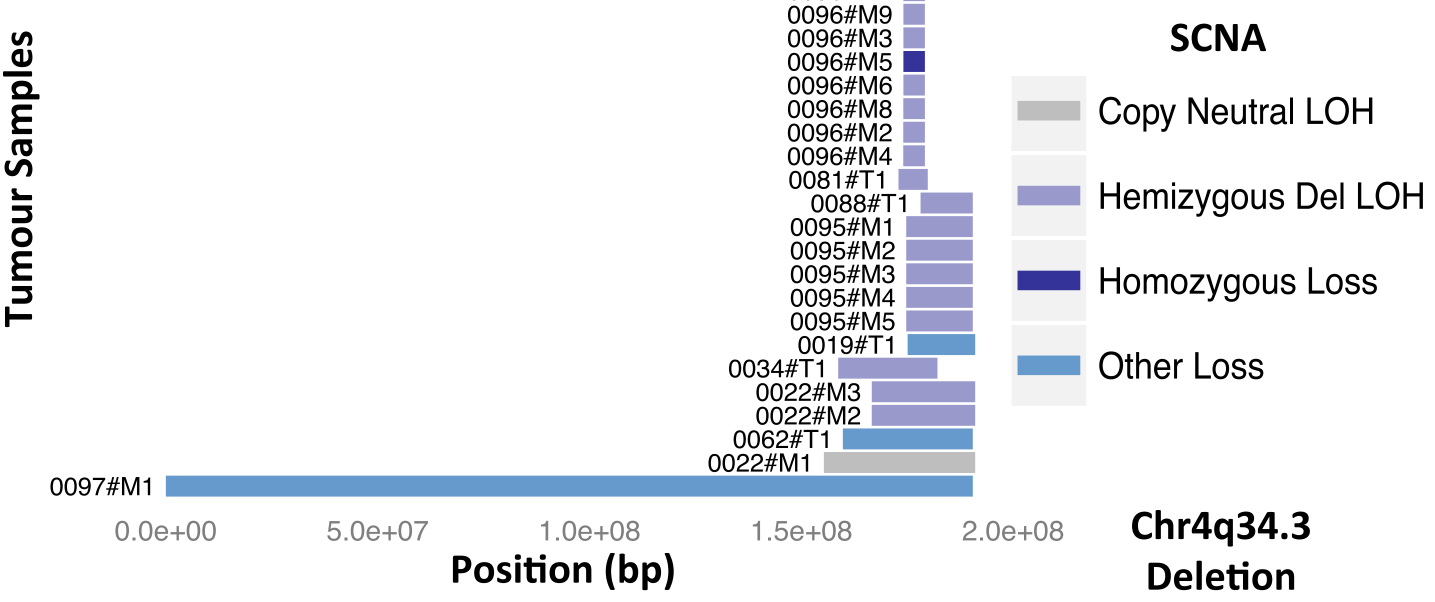
SCNA
Copy Neutral LOH
Hemizygous Del LOH
Homozygous Loss
Other Loss
Tumour Samples
0028#T1
0026#T1
0105#M1
0096#M7
0096#M1
0096#M9
0096#M3
0096#M5
0096#M6
0096#M8
0096#M2
0096#M4
0081#T1
0088#T1
0095#M1
0095#M2
0095#M3
0095#M4
0095#M5
0019#T1
0034#T1
0022#M3
0022#M2
0062#T1
0022#M1
0097#M1
0.0e+00
5.0e+07
1.0e+08
1.5e+08
2.0e+08
Position (bp)
Chr4q34.3
Deletion

i

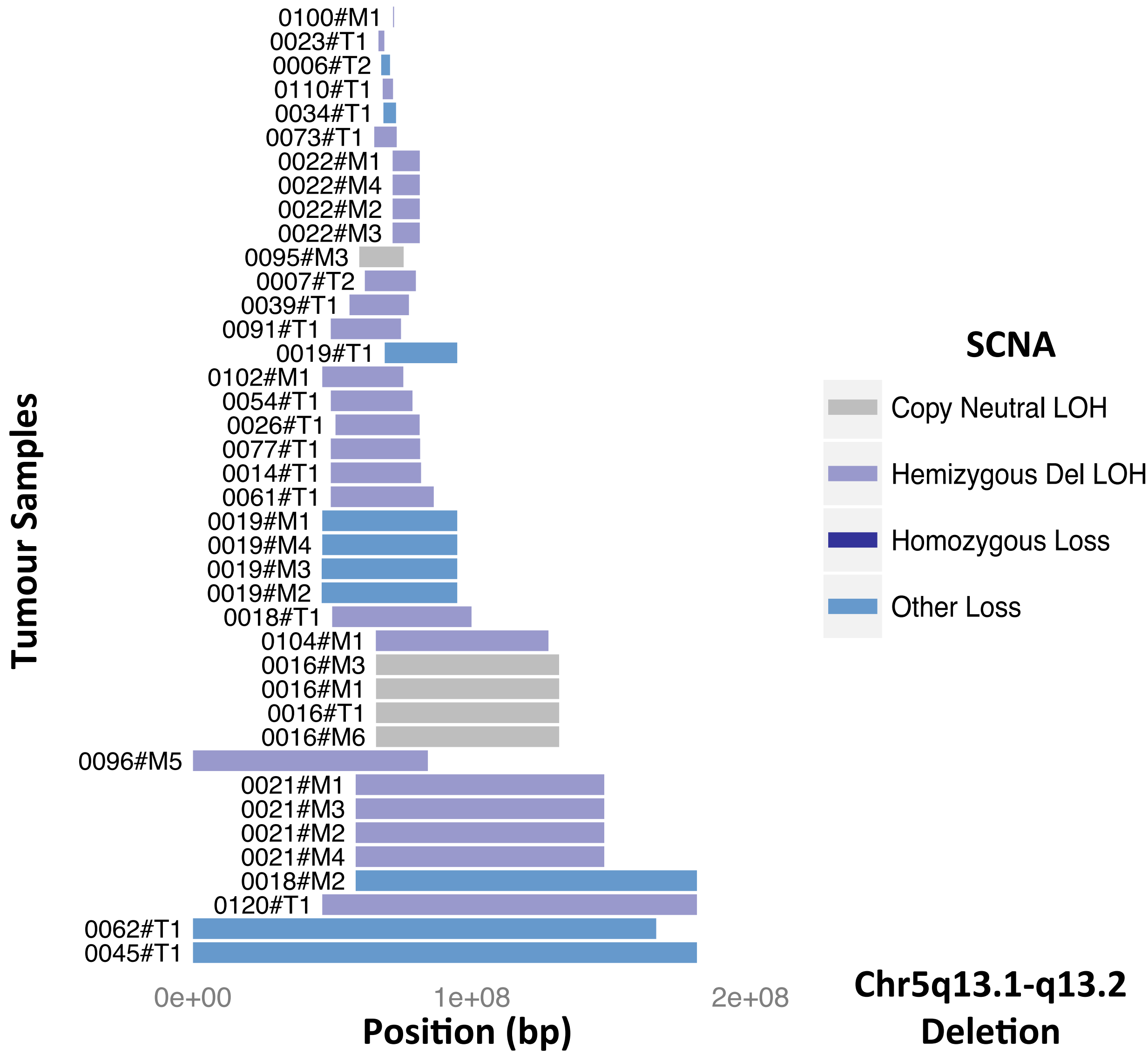

0100#M1
0023#T1
0006#T2
0110#T1
0034#T1
0073#T1
0022#M1
0022#M4
0022#M2
0022#M3
0095#M3
0007#T2
0039#T1
0091#T1
0019#T1
0102#M1
0054#T1
0026#T1
0077#T1
0014#T1
0061#T1
0019#M1
0019#M4
0019#M3
0019#M2
0018#T1
0104#M1
0016#M3
0016#M1
0016#T1
0016#M6
0096#M5
0021#M1
0021#M3
0021#M2
0021#M4
0018#M2
0120#T1
0062#T1
0045#T1
Tumour Samples
0e+00
1e+08
2e+08
Position (bp)
SCNA
Copy Neutral LOH
Hemizygous Del LOH
Homozygous Loss
Other Loss
Chr5q13.1-q13.2
Deletion

j

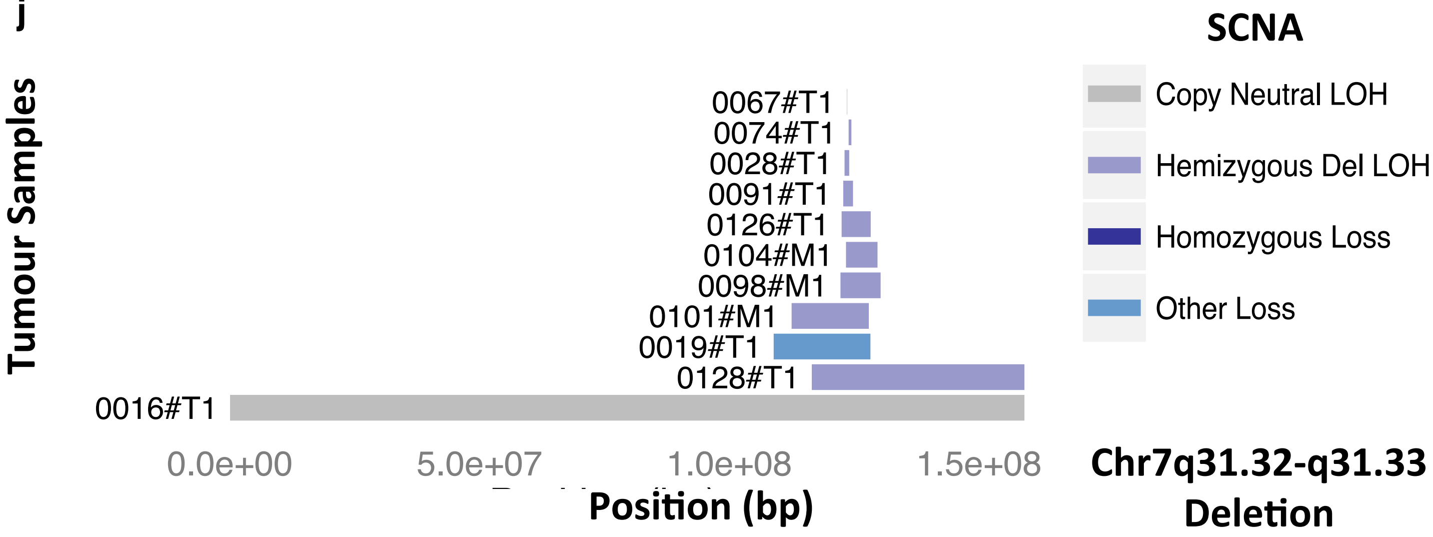

SCNA
0067#T1
0074#T1
0028#T1
0091#T1
0126#T1
0104#M1
0098#M1
0101#M1
0019#T1
0128#T1
0016#T1
Tumour Samples
0.0e+00
5.0e+07
1.0e+08
1.5e+08
Position (bp)
Copy Neutral LOH
Hemizygous Del LOH
Homozygous Loss
Other Loss
Chr7q31.32-q31.33
Deletion

k

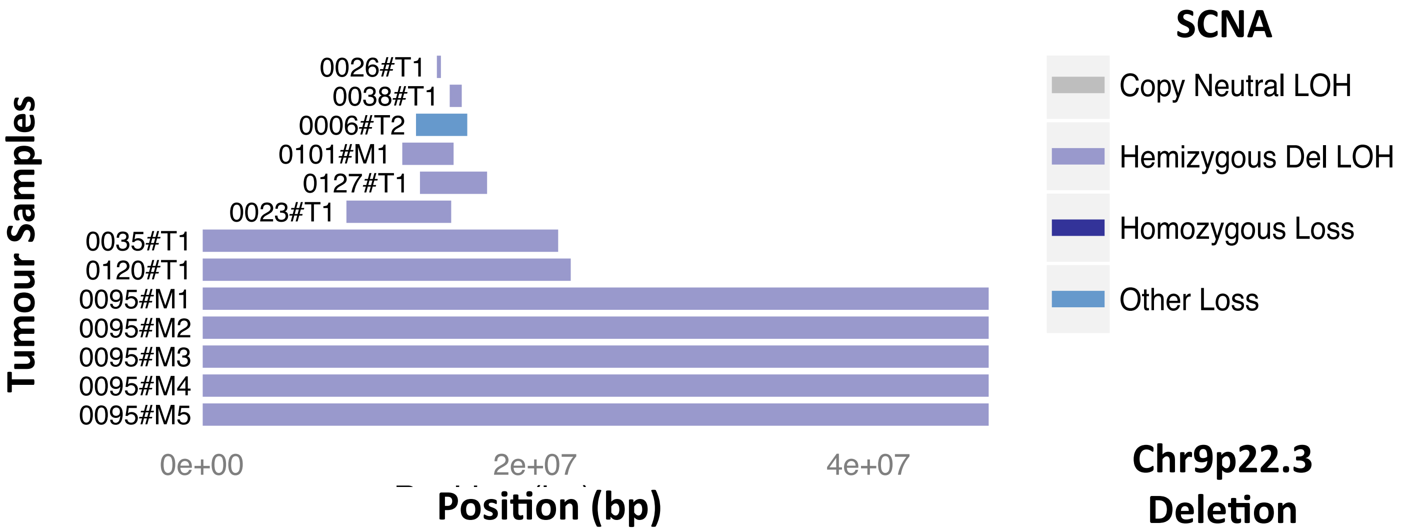
SCNA
Copy Neutral LOH
Hemizygous Del LOH
Homozygous Loss
Other Loss
Tumour Samples
0026#T1
0038#T1
0006#T2
0101#M1
0127#T1
0023#T1
0035#T1
0120#T1
0095#M1
0095#M2
0095#M3
0095#M4
0095#M5
0e+00
2e+07
4e+07
Position (bp)
Chr9p22.3
Deletion

l

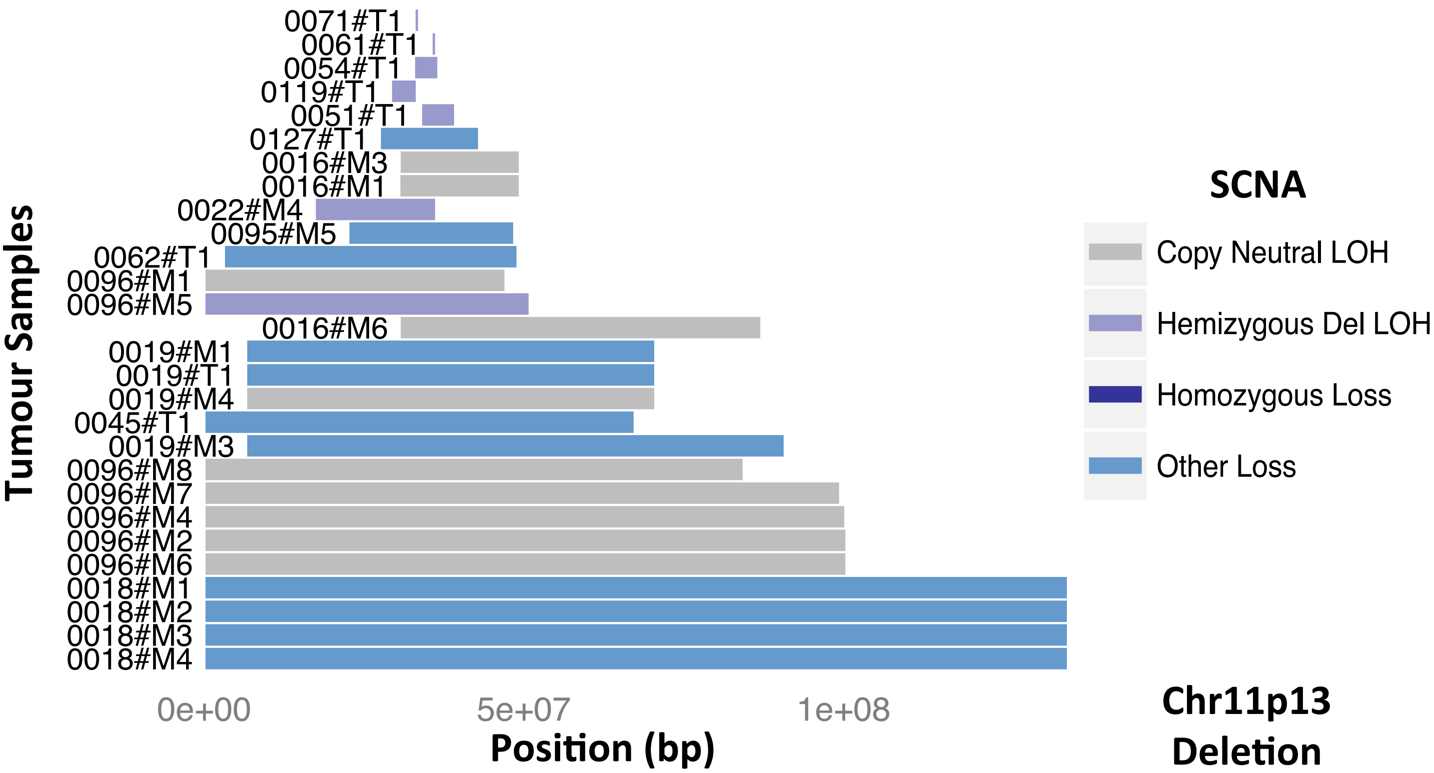
SCNA
Copy Neutral LOH
Hemizygous Del LOH
Homozygous Loss
Other Loss
Tumour Samples
0071#T1
0061#T1
0054#T1
0119#T1
0051#T1
0127#T1
0016#M3
0016#M1
0022#M4
0095#M5
0062#T1
0096#M1
0096#M5
0016#M6
0019#M1
0019#T1
0019#M4
0045#T1
0019#M3
0096#M8
0096#M7
0096#M4
0096#M2
0096#M6
0018#M1
0018#M2
0018#M3
0018#M4
0e+00
5e+07
1e+08
Position (bp)
Chr11p13
Deletion

m

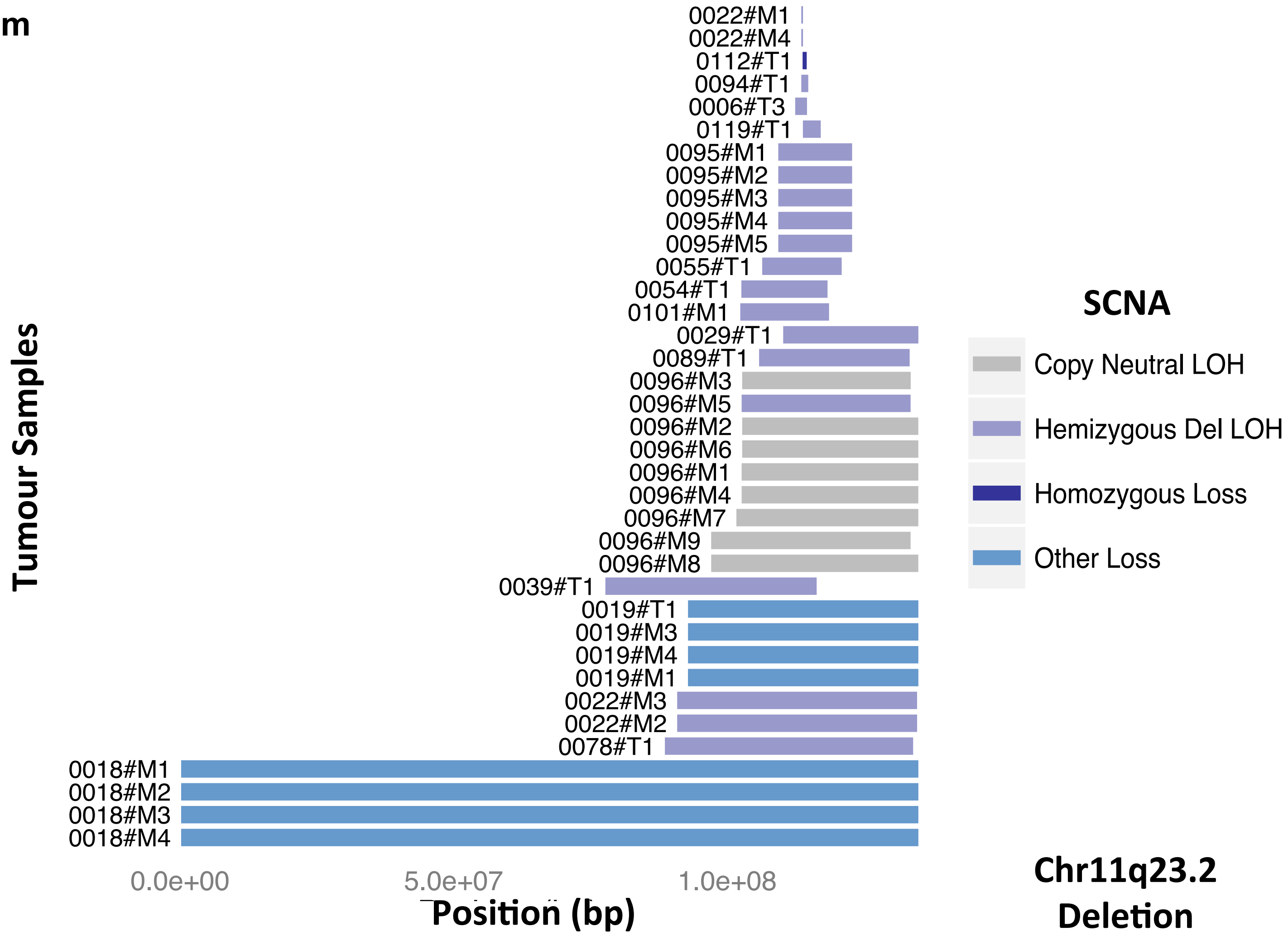

0022#M1
0022#M4
0112#T1
0094#T1
0006#T3
0119#T1
0095#M1
0095#M2
0095#M3
0095#M4
0095#M5
0055#T1
0054#T1
0101#M1
0029#T1
0089#T1
0096#M3
0096#M5
0096#M2
0096#M6
0096#M1
0096#M4
0096#M7
0096#M9
0096#M8
0039#T1
0019#T1
0019#M3
0019#M4
0019#M1
0022#M3
0022#M2
0078#T1
0018#M1
0018#M2
0018#M3
0018#M4
Tumour Samples
0.0e+00
5.0e+07
1.0e+08
Position (bp)
SCNA
Copy Neutral LOH
Hemizygous Del LOH
Homozygous Loss
Other Loss
Chr11q23.2
Deletion

n

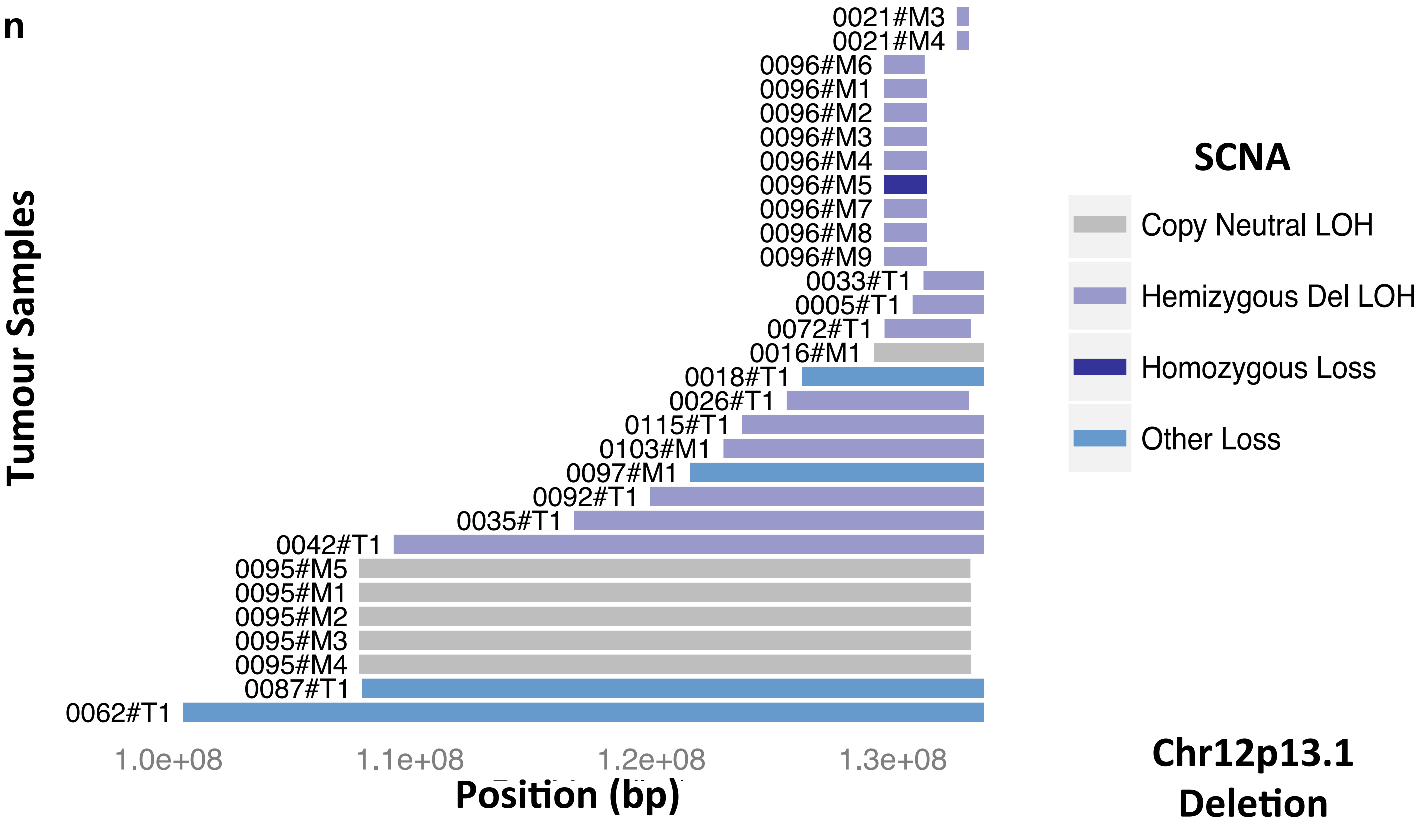

0021#M3
0021#M4
0096#M6
0096#M1
0096#M2
0096#M3
0096#M4
0096#M5
0096#M7
0096#M8
0096#M9
0033#T1
0005#T1
0072#T1
0016#M1
0018#T1
0026#T1
0115#T1
0103#M1
0097#M1
0092#T1
0035#T1
0042#T1
0095#M5
0095#M1
0095#M2
0095#M3
0095#M4
0087#T1
0062#T1
Tumour Samples
1.0e+08
1.1e+08
1.2e+08
1.3e+08
Position (bp)
SCNA
Copy Neutral LOH
Hemizygous Del LOH
Homozygous Loss
Other Loss
Chr12p13.1
Deletion

o

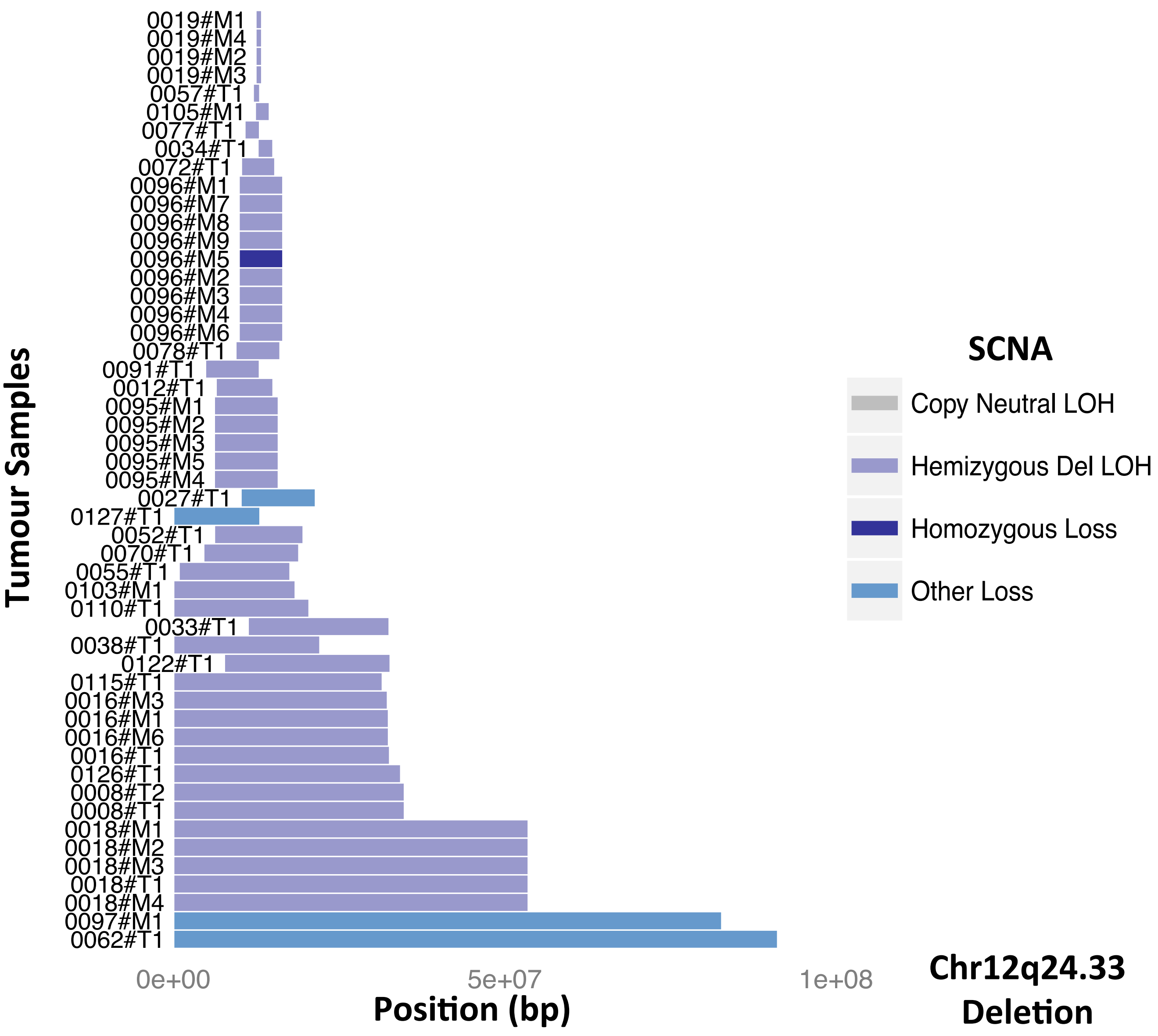

0019#M1
0019#M4
0019#M2
0019#M3
0057#T1
0105#M1
0077#T1
0034#T1
0072#T1
0096#M1
0096#M7
0096#M8
0096#M9
0096#M5
0096#M2
0096#M3
0096#M4
0096#M6
0078#T1
0091#T1
0012#T1
0095#M1
0095#M2
0095#M3
0095#M5
0095#M4
0027#T1
0127#T1
0052#T1
0070#T1
0055#T1
0103#M1
0110#T1
0033#T1
0038#T1
0122#T1
0115#T1
0016#M3
0016#M1
0016#M6
0016#T1
0126#T1
0008#T2
0008#T1
0018#M1
0018#M2
0018#M3
0018#T1
0018#M4
0097#M1
0062#T1
Tumour Samples
0e+00
5e+07
1e+08
Position (bp)
SCNA
Copy Neutral LOH
Hemizygous Del LOH
Homozygous Loss
Other Loss
Chr12q24.33
Deletion

p

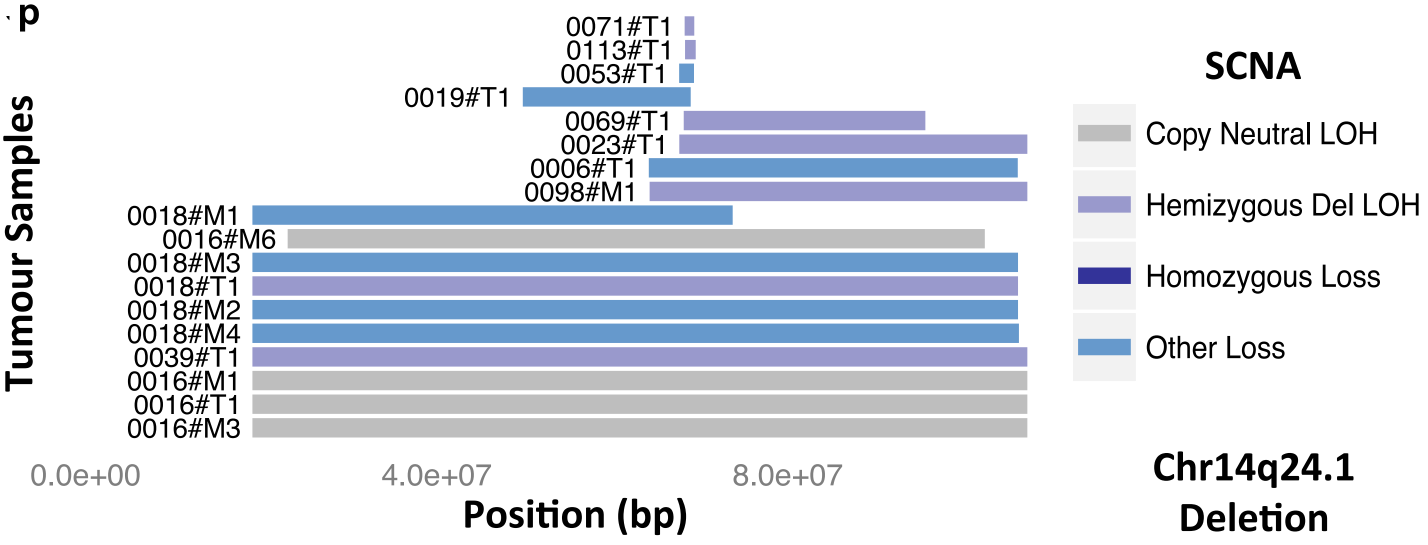

0071#T1
0113#T1
0053#T1
0019#T1
0069#T1
0023#T1
0006#T1
0098#M1
0018#M1
0016#M6
0018#M3
0018#T1
0018#M2
0018#M4
0039#T1
0016#M1
0016#T1
0016#M3
Tumour Samples
0.0e+00
4.0e+07
8.0e+07
Position (bp)
SCNA
Copy Neutral LOH
Hemizygous Del LOH
Homozygous Loss
Other Loss
Chr14q24.1
Deletion

q

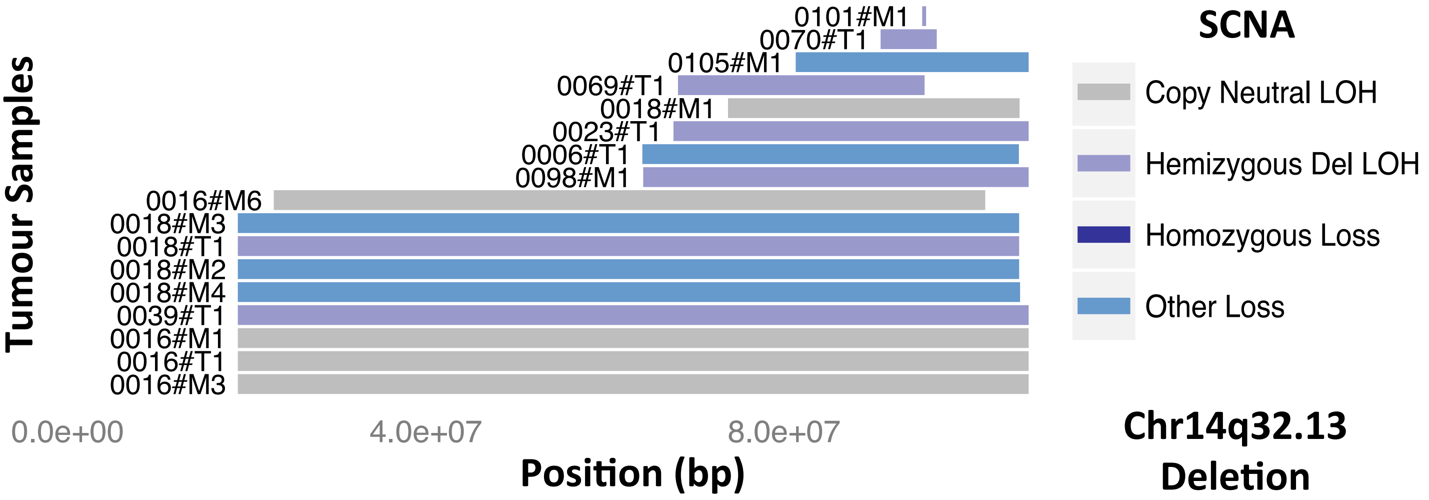

SCNA
Copy Neutral LOH
Hemizygous Del LOH
Homozygous Loss
Other Loss
Tumour Samples
0101#M1
0070#T1
0105#M1
0069#T1
0018#M1
0023#T1
0006#T1
0098#M1
0016#M6
0018#M3
0018#T1
0018#M2
0018#M4
0039#T1
0016#M1
0016#T1
0016#M3
0.0e+00
4.0e+07
8.0e+07
Position (bp)
Chr14q32.13
Deletion

r

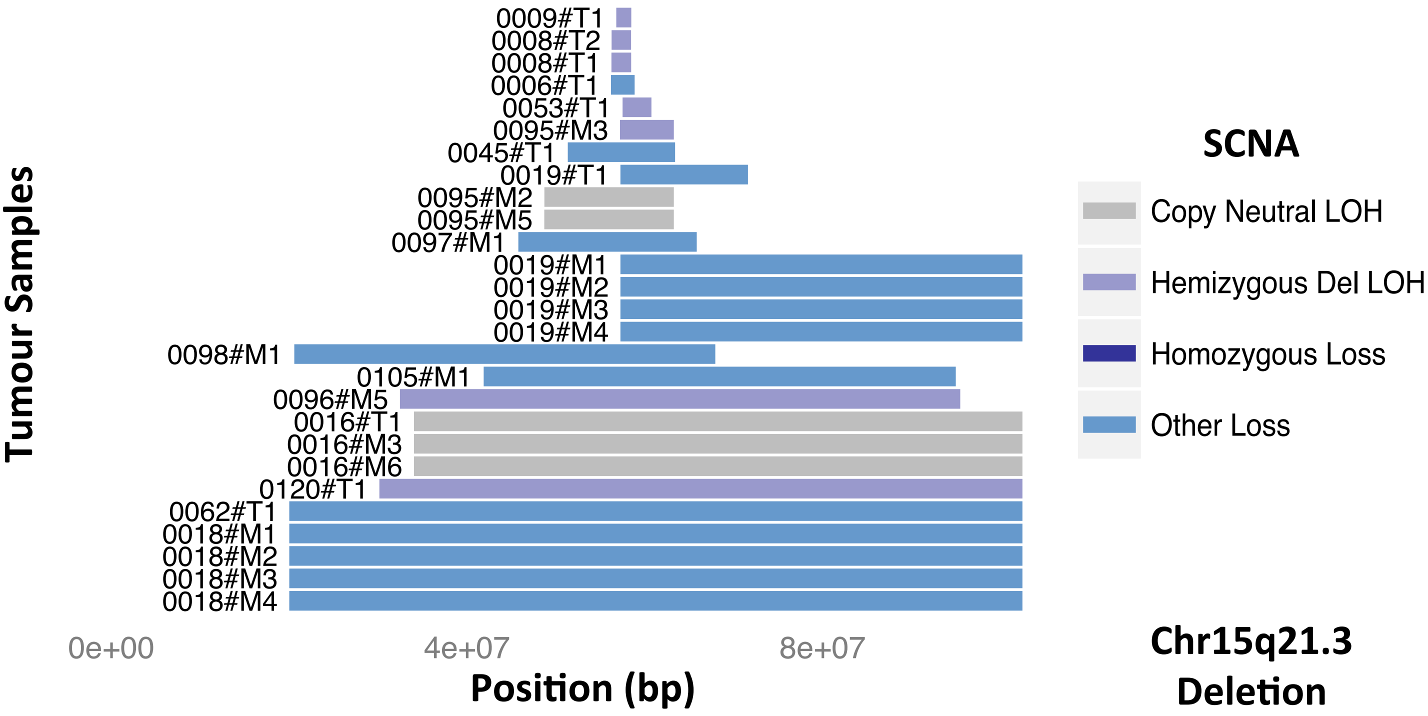

SCNA
Copy Neutral LOH
Hemizygous Del LOH
Homozygous Loss
Other Loss
Tumour Samples
0009#T1
0008#T2
0008#T1
0006#T1
0053#T1
0095#M3
0045#T1
0019#T1
0095#M2
0095#M5
0097#M1
0019#M1
0019#M2
0019#M3
0019#M4
0098#M1
0105#M1
0096#M5
0016#T1
0016#M3
0016#M6
0120#T1
0062#T1
0018#M1
0018#M2
0018#M3
0018#M4
0e+00
4e+07
8e+07
Position (bp)
Chr15q21.3
Deletion

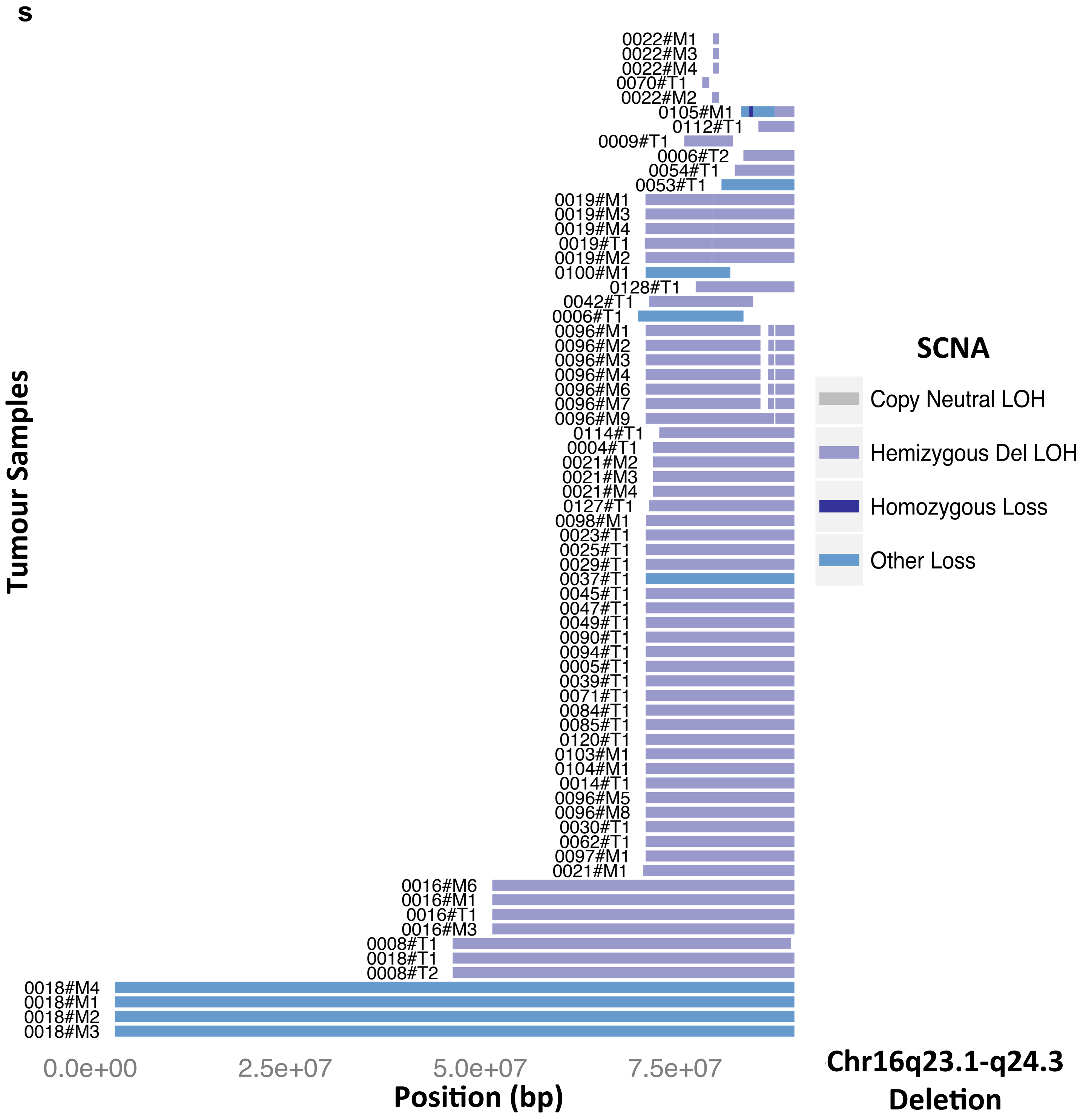
s
0022#M1
0022#M3
0022#M4
0070#T1
0022#M2
0105#M1
0112#T1
0009#T1
0006#T2
0054#T1
0053#T1
0019#M1
0019#M3
0019#M4
0019#T1
0019#M2
0100#M1
0128#T1
0042#T1
0006#T1
0096#M1
0096#M2
0096#M3
0096#M4
0096#M6
0096#M7
0096#M9
0114#T1
0004#T1
0021#M2
0021#M3
0021#M4
0127#T1
0098#M1
0023#T1
0025#T1
0029#T1
0037#T1
0045#T1
0047#T1
0049#T1
0090#T1
0094#T1
0005#T1
0039#T1
0071#T1
0084#T1
0085#T1
0120#T1
0103#M1
0104#M1
0014#T1
0096#M5
0096#M8
0030#T1
0062#T1
0097#M1
0021#M1
0016#M6
0016#M1
0016#T1
0016#M3
0008#T1
0018#T1
0008#T2
0018#M4
0018#M1
0018#M2
0018#M3
Tumour Samples
0.0e+00
2.5e+07
5.0e+07
7.5e+07
Position (bp)
SCNA
Copy Neutral LOH
Hemizygous Del LOH
Homozygous Loss
Other Loss
Chr16q23.1-q24.3
Deletion

t

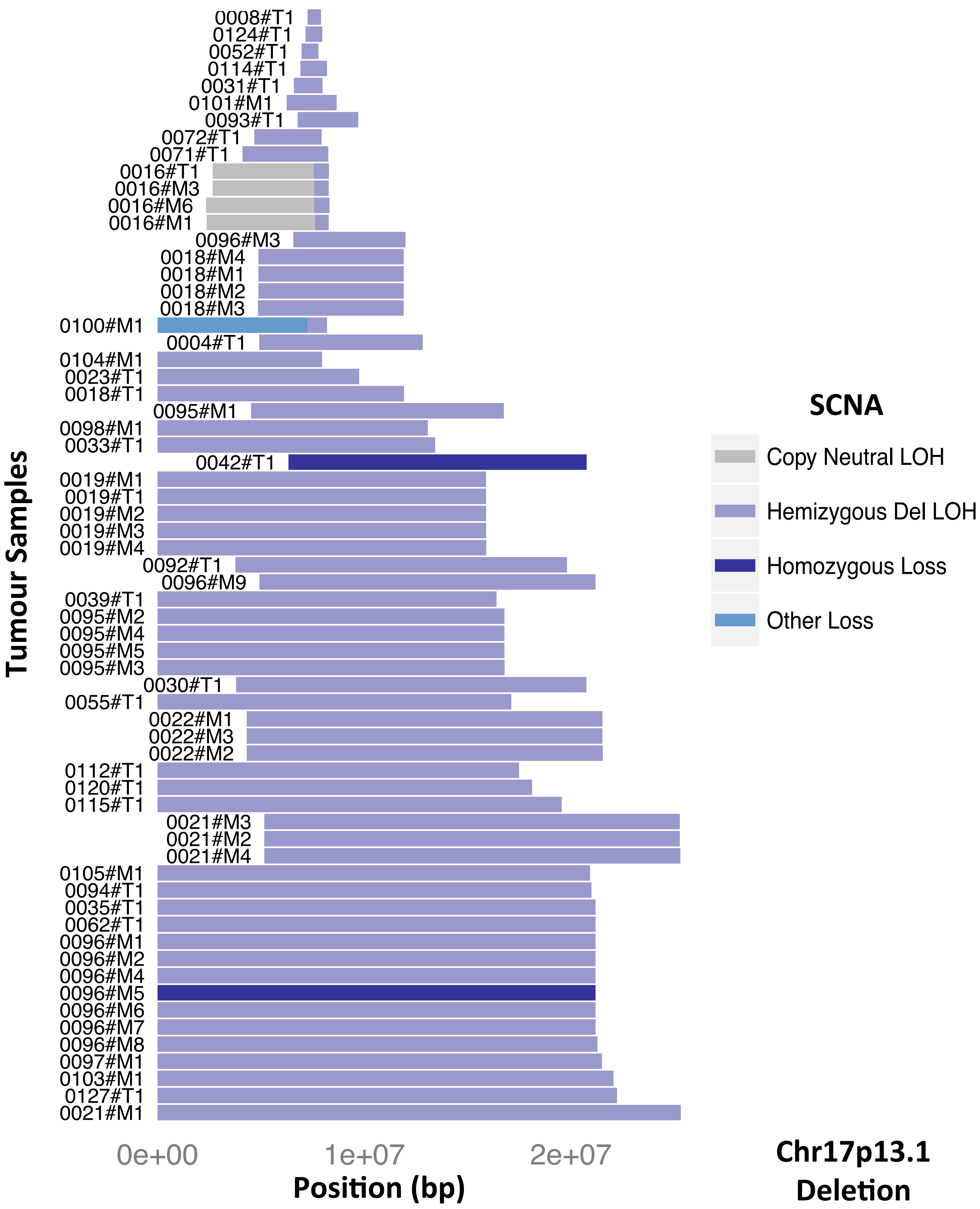

0008#T1
0124#T1
0052#T1
0114#T1
0031#T1
0101#M1
0093#T1
0072#T1
0071#T1
0016#T1
0016#M3
0016#M6
0016#M1
0096#M3
0018#M4
0018#M1
0018#M2
0018#M3
0100#M1
0004#T1
0104#M1
0023#T1
0018#T1
0095#M1
0098#M1
0033#T1
0042#T1
0019#M1
0019#T1
0019#M2
0019#M3
0019#M4
0092#T1
0096#M9
0039#T1
0095#M2
0095#M4
0095#M5
0095#M3
0030#T1
0055#T1
0022#M1
0022#M3
0022#M2
0112#T1
0120#T1
0115#T1
0021#M3
0021#M2
0021#M4
0105#M1
0094#T1
0035#T1
0062#T1
0096#M1
0096#M2
0096#M4
0096#M5
0096#M6
0096#M7
0096#M8
0097#M1
0103#M1
0127#T1
0021#M1
Tumour Samples
0e+00
1e+07
2e+07
Position (bp)
SCNA
Copy Neutral LOH
Hemizygous Del LOH
Homozygous Loss
Other Loss
Chr17p13.1
Deletion

u

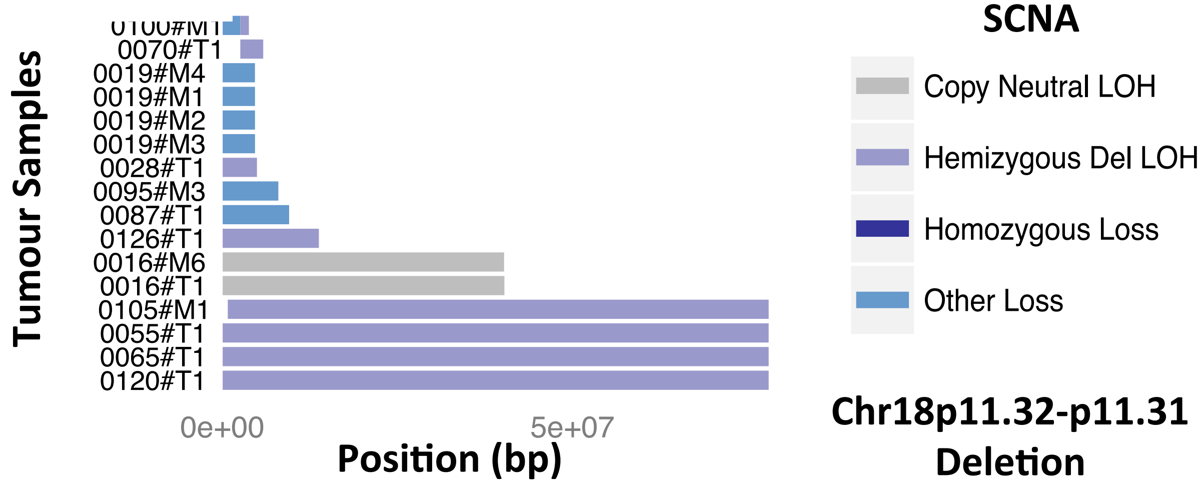
0100#M1
0070#T1
0019#M4
0019#M1
0019#M2
0019#M3
0028#T1
0095#M3
0087#T1
0126#T1
0016#M6
0016#T1
0105#M1
0055#T1
0065#T1
0120#T1
Tumour Samples
0e+00
5e+07
Position (bp)
SCNA
Copy Neutral LOH
Hemizygous Del LOH
Homozygous Loss
Other Loss
Chr18p11.32-p11.31
Deletion

v

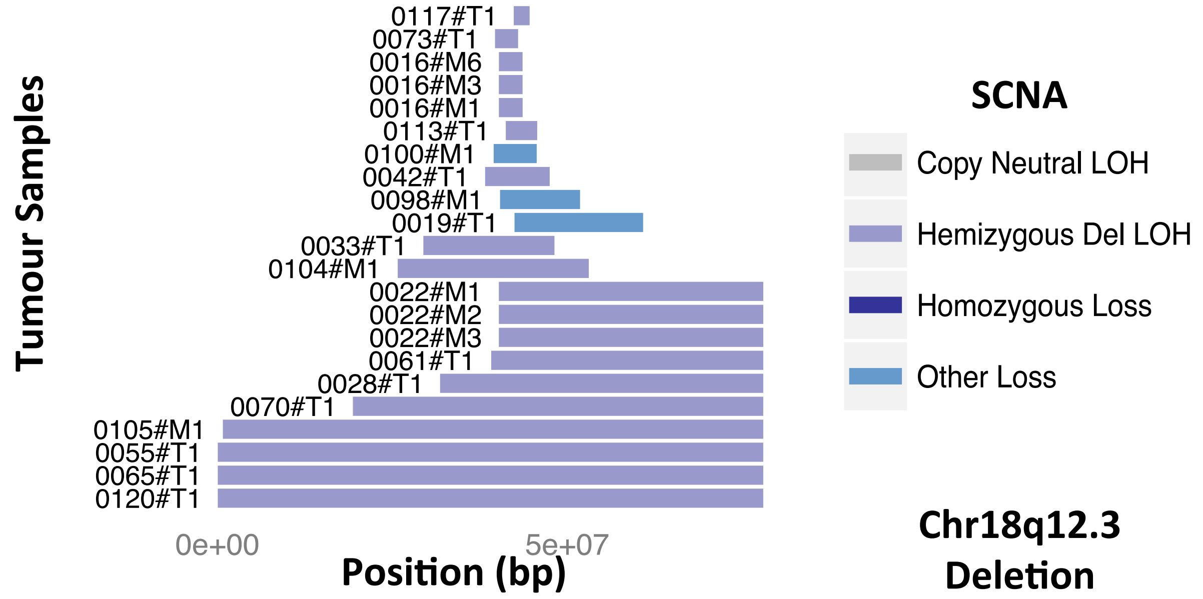
0117#T1
0073#T1
0016#M6
0016#M3
0016#M1
0113#T1
0100#M1
0042#T1
0098#M1
0019#T1
0033#T1
0104#M1
0022#M1
0022#M2
0022#M3
0061#T1
0028#T1
0070#T1
0105#M1
0055#T1
0065#T1
0120#T1
Tumour Samples
0e+00
5e+07
Position (bp)
SCNA
Copy Neutral LOH
Hemizygous Del LOH
Homozygous Loss
Other Loss
Chr18q12.3
Deletion

w

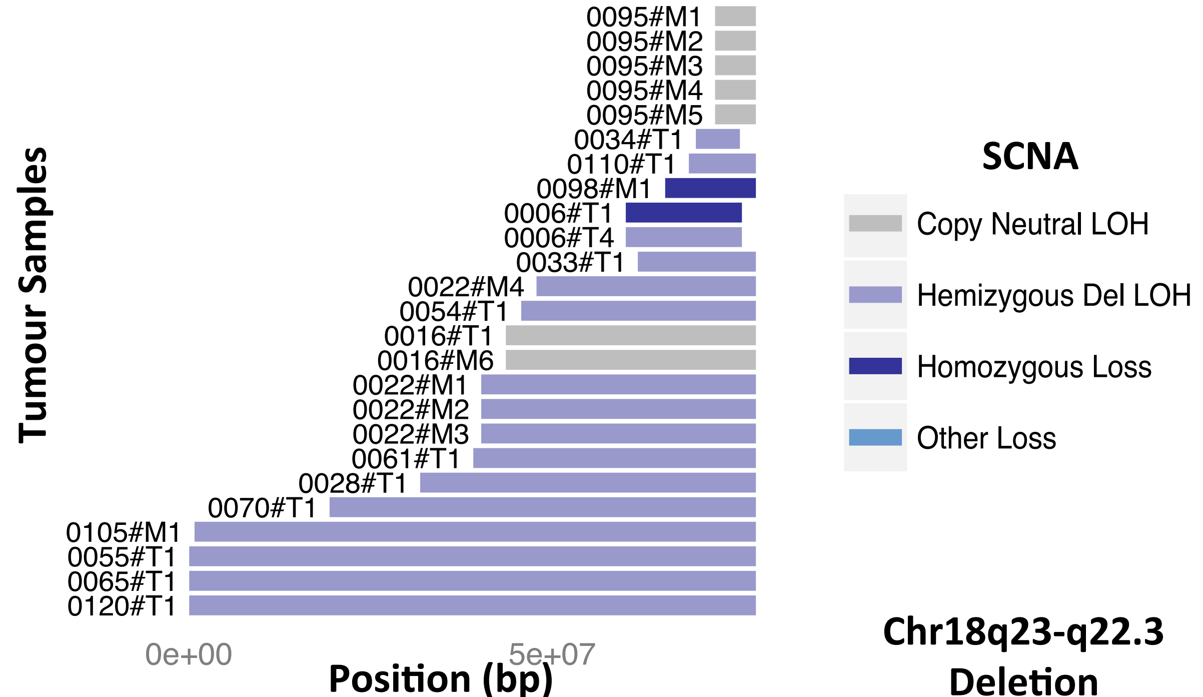
0095#M1
0095#M2
0095#M3
0095#M4
0095#M5
0034#T1
0110#T1
0098#M1
0006#T1
0006#T4
0033#T1
0022#M4
0054#T1
0016#T1
0016#M6
0022#M1
0022#M2
0022#M3
0061#T1
0028#T1
0070#T1
0105#M1
0055#T1
0065#T1
0120#T1
Tumour Samples
0e+00
5e+07
Position (bp)
SCNA
Copy Neutral LOH
Hemizygous Del LOH
Homozygous Loss
Other Loss
Chr18q23-q22.3
Deletion

x

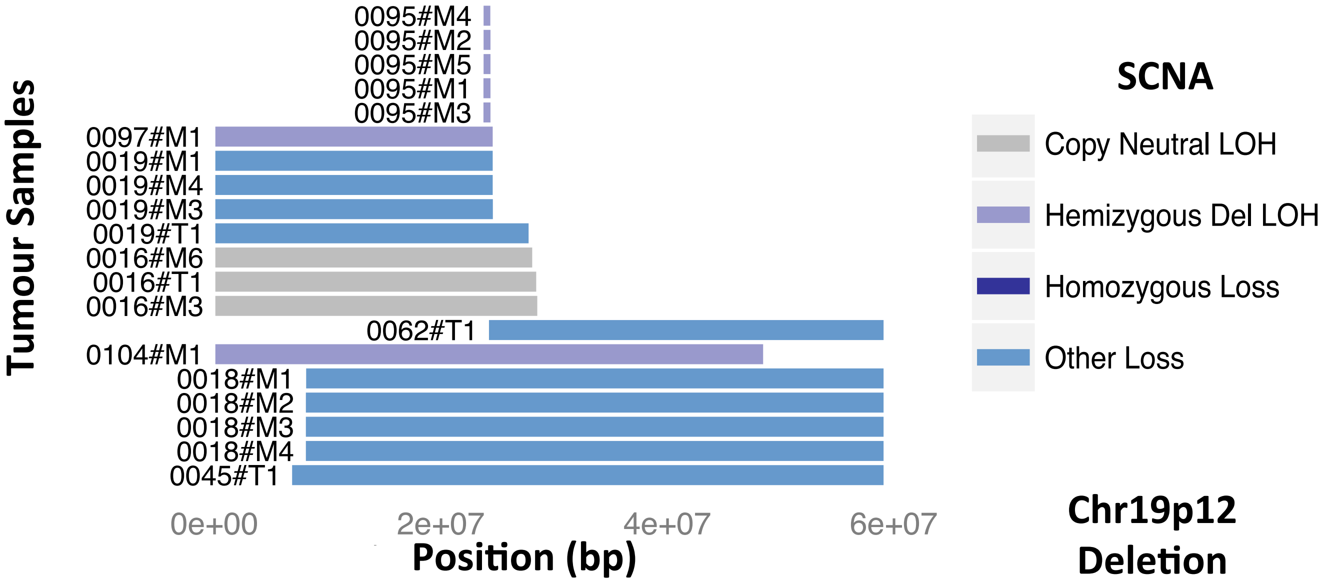

0095#M4
0095#M2
0095#M5
0095#M1
0095#M3
0097#M1
0019#M1
0019#M4
0019#M3
0019#T1
0016#M6
0016#T1
0016#M3
0062#T1
0104#M1
0018#M1
0018#M2
0018#M3
0018#M4
0045#T1
Tumour Samples
0e+00
2e+07
4e+07
6e+07
Position (bp)
SCNA
Copy Neutral LOH
Hemizygous Del LOH
Homozygous Loss
Other Loss
Chr19p12
Deletion

y

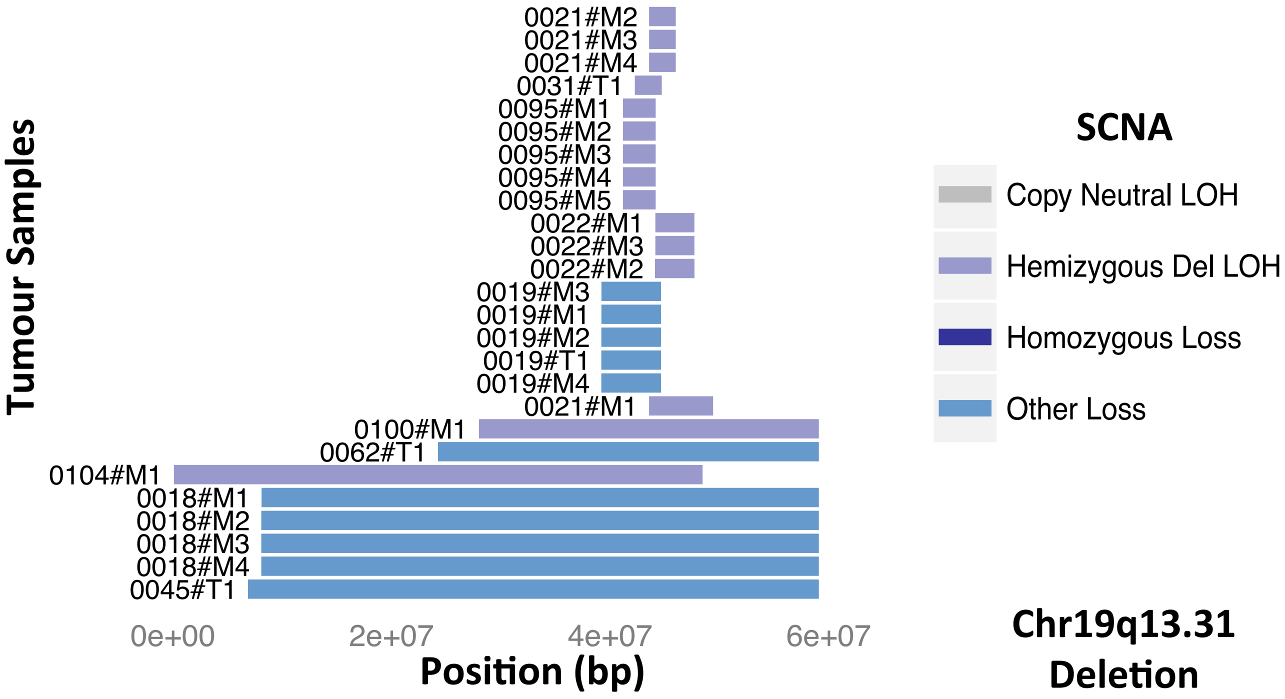

0021#M2
0021#M3
0021#M4
0031#T1
0095#M1
0095#M2
0095#M3
0095#M4
0095#M5
0022#M1
0022#M3
0022#M2
0019#M3
0019#M1
0019#M2
0019#T1
0019#M4
0021#M1
0100#M1
0062#T1
0104#M1
0018#M1
0018#M2
0018#M3
0018#M4
0045#T1
Tumour Samples
0e+00
2e+07
4e+07
6e+07
Position (bp)
SCNA
Copy Neutral LOH
Hemizygous Del LOH
Homozygous Loss
Other Loss
Chr19q13.31
Deletion

z

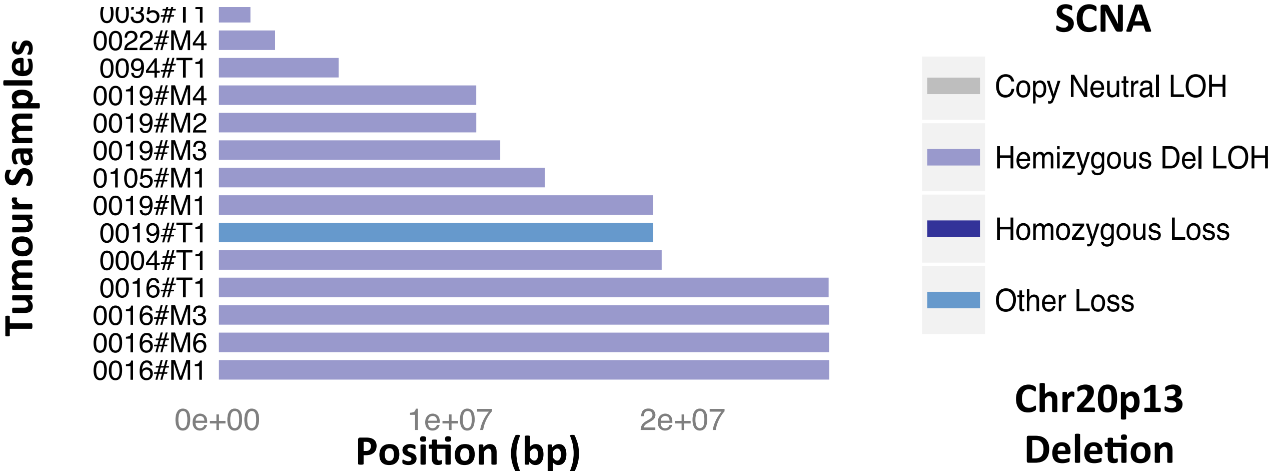

0022#M4
0094#T1
0019#M4
0019#M2
0019#M3
0105#M1
0019#M1
0019#T1
0004#T1
0016#T1
0016#M3
0016#M6
0016#M1
Tumour Samples
0e+00
1e+07
2e+07
Position (bp)
SCNA
Copy Neutral LOH
Hemizygous Del LOH
Homozygous Loss
Other Loss
Chr20p13
Deletion

a2

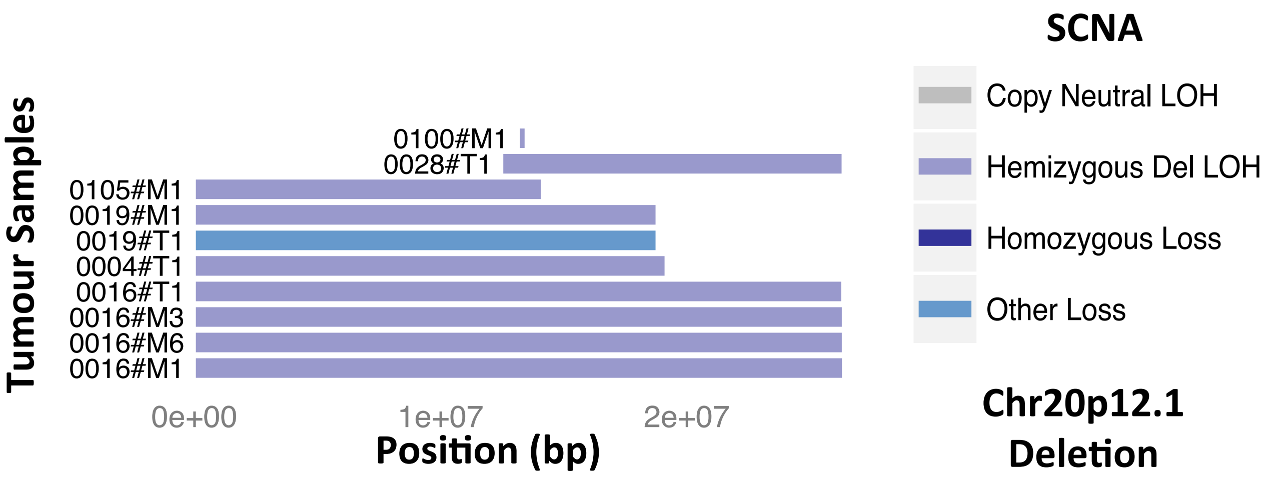

Tumour Samples
0100#M1
0028#T1
0105#M1
0019#M1
0019#T1
0004#T1
0016#T1
0016#M3
0016#M6
0016#M1
0e+00
1e+07
2e+07
Position (bp)
SCNA
Copy Neutral LOH
Hemizygous Del LOH
Homozygous Loss
Other Loss
Chr20p12.1
Deletion

b2

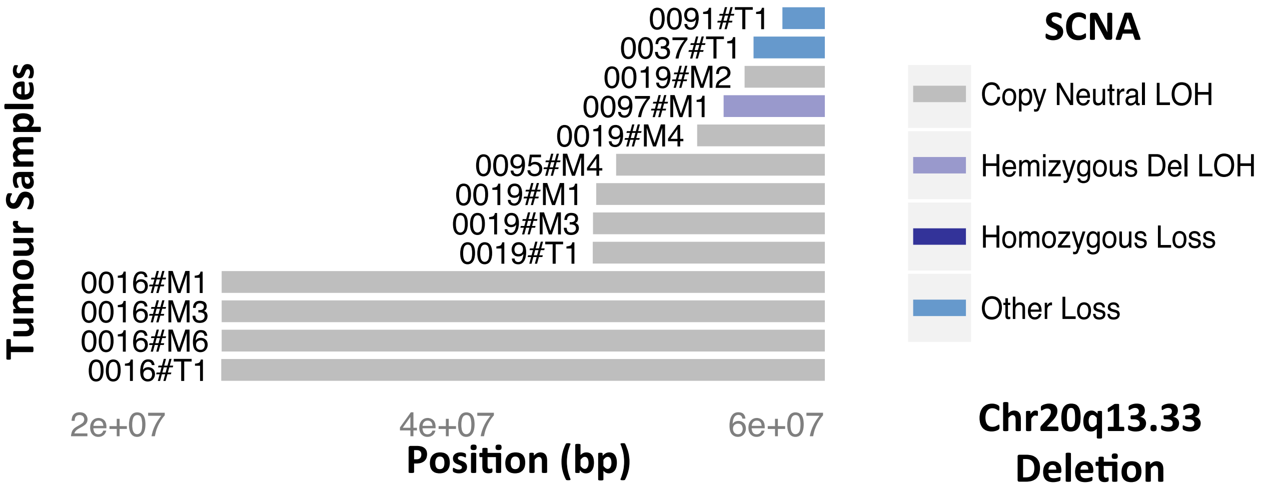

Tumour Samples
0091#T1
0037#T1
0019#M2
0097#M1
0019#M4
0095#M4
0019#M1
0019#M3
0019#T1
0016#M1
0016#M3
0016#M6
0016#T1
2e+07
4e+07
6e+07
Position (bp)
SCNA
Copy Neutral LOH
Hemizygous Del LOH
Homozygous Loss
Other Loss
Chr20q13.33
Deletion

c2

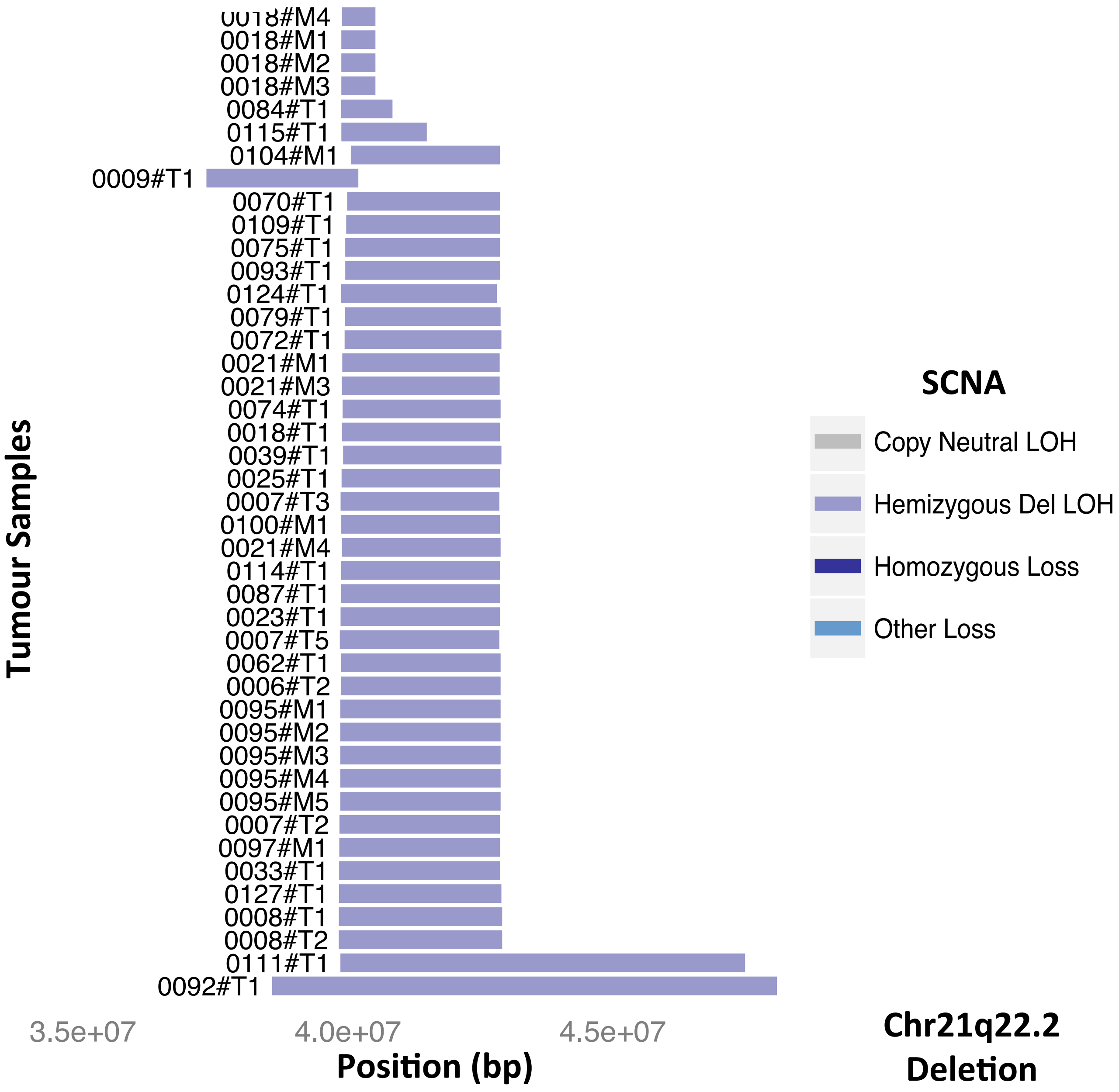

0018#M4
0018#M1
0018#M2
0018#M3
0084#T1
0115#T1
0104#M1
0009#T1
0070#T1
0109#T1
0075#T1
0093#T1
0124#T1
0079#T1
0072#T1
0021#M1
0021#M3
0074#T1
0018#T1
0039#T1
0025#T1
0007#T3
0100#M1
0021#M4
0114#T1
0087#T1
0023#T1
0007#T5
0062#T1
0006#T2
0095#M1
0095#M2
0095#M3
0095#M4
0095#M5
0007#T2
0097#M1
0033#T1
0127#T1
0008#T1
0008#T2
0111#T1
0092#T1
Tumour Samples
3.5e+07
4.0e+07
4.5e+07
Position (bp)
SCNA
Copy Neutral LOH
Hemizygous Del LOH
Homozygous Loss
Other Loss
Chr21q22.2
Deletion

d2

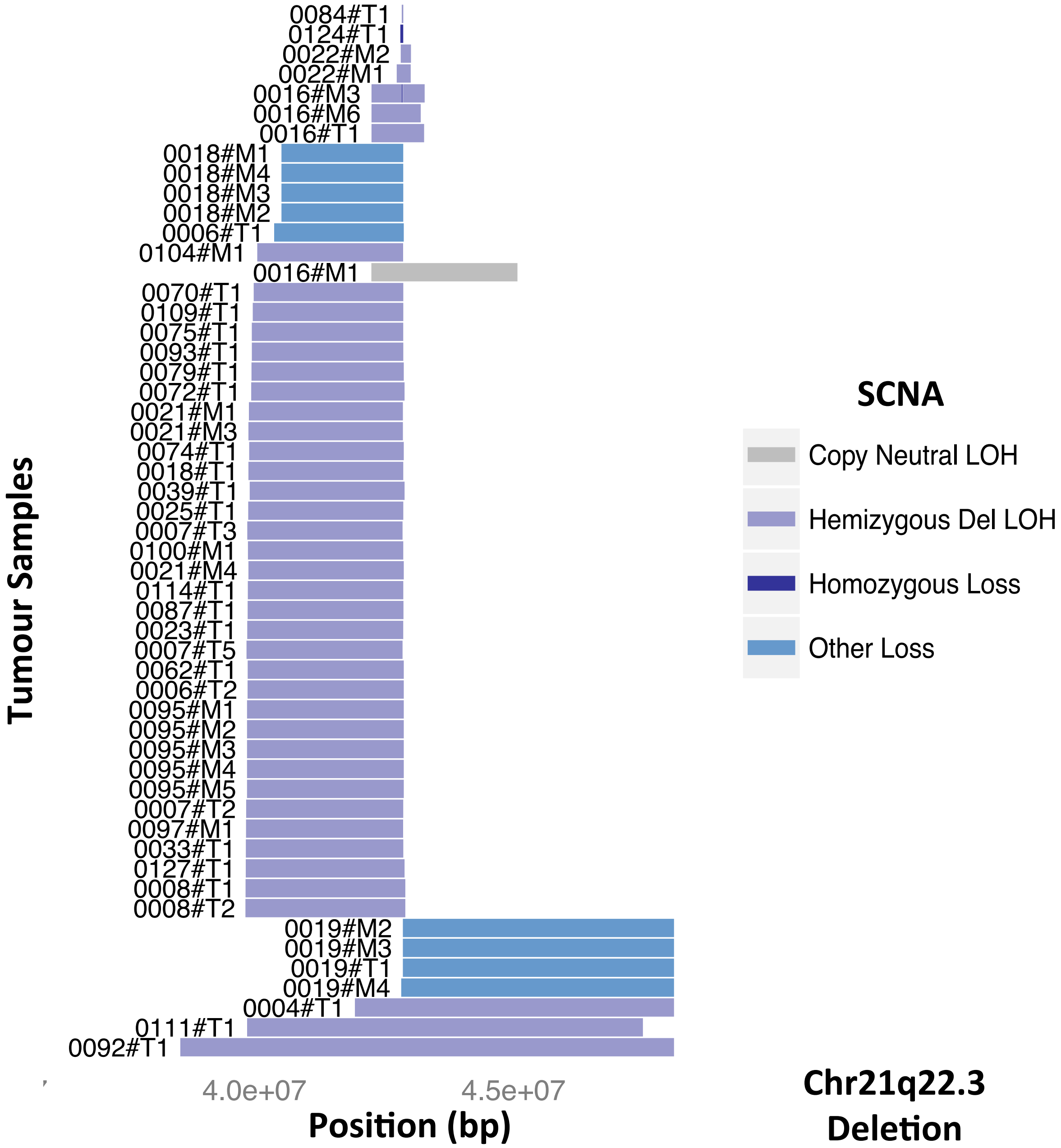

Chr21q22.3
Deletion

e2

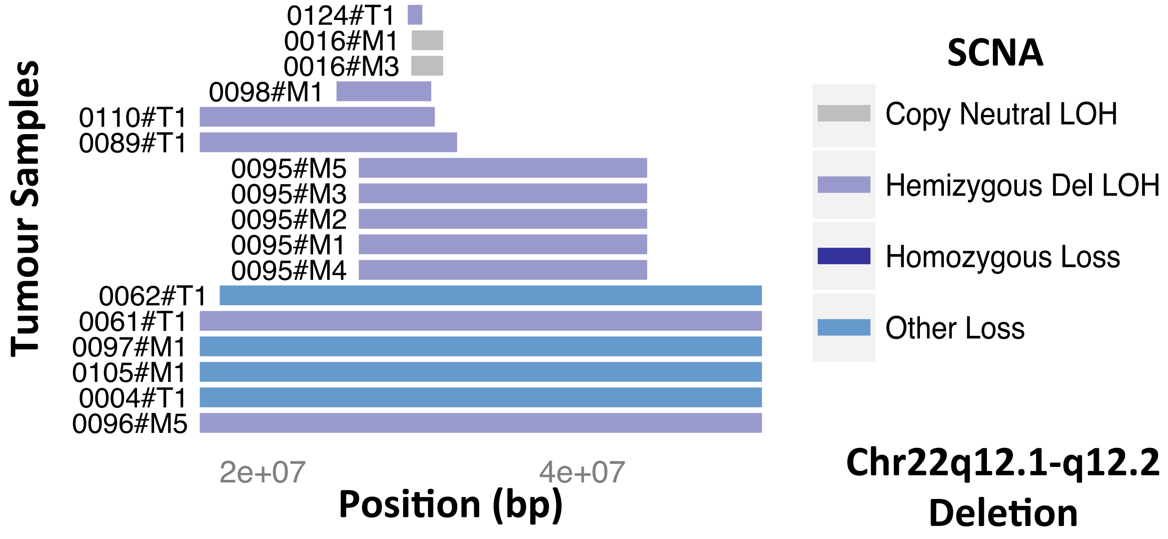
0124#T1
0016#M1
0016#M3
0098#M1
0110#T1
0089#T1
0095#M5
0095#M3
0095#M2
0095#M1
0095#M4
0062#T1
0061#T1
0097#M1
0105#M1
0004#T1
0096#M5
Tumour Samples
2e+07
4e+07
Position (bp)
SCNA
Copy Neutral LOH
Hemizygous Del LOH
Homozygous Loss
Other Loss
Chr22q12.1-q12.2
Deletion

f2

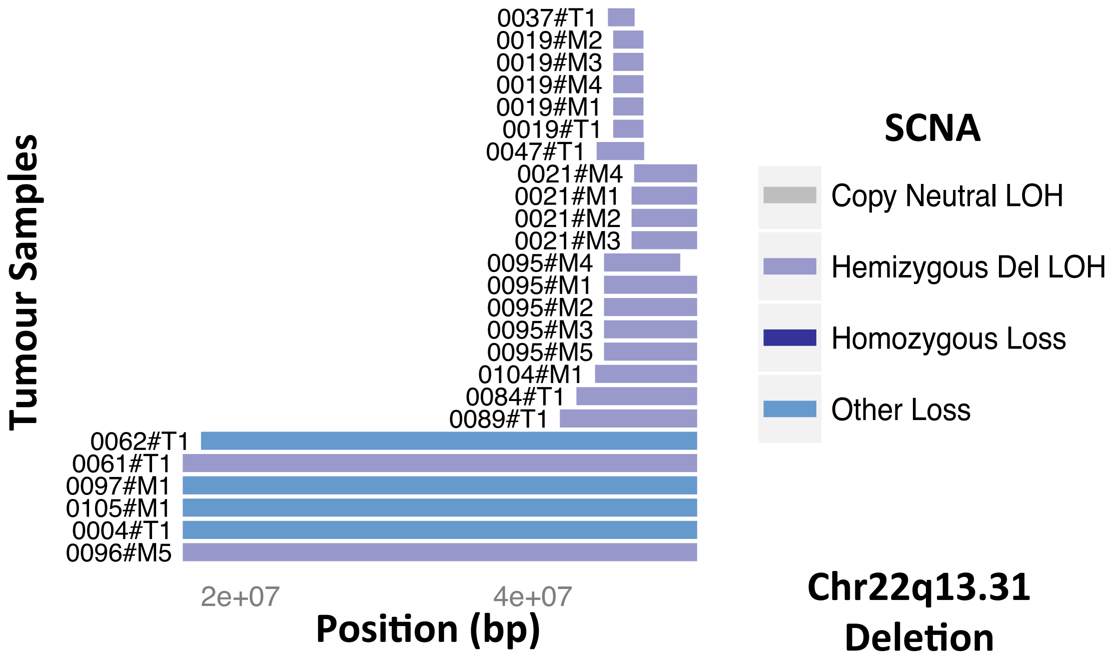
0037#T1
0019#M2
0019#M3
0019#M4
0019#M1
0019#T1
0047#T1
0021#M4
0021#M1
0021#M2
0021#M3
0095#M4
0095#M1
0095#M2
0095#M3
0095#M5
0104#M1
0084#T1
0089#T1
0062#T1
0061#T1
0097#M1
0105#M1
0004#T1
0096#M5
Tumour Samples
2e+07
4e+07
Position (bp)
SCNA
Copy Neutral LOH
Hemizygous Del LOH
Homozygous Loss
Other Loss
Chr22q13.31
Deletion

g2

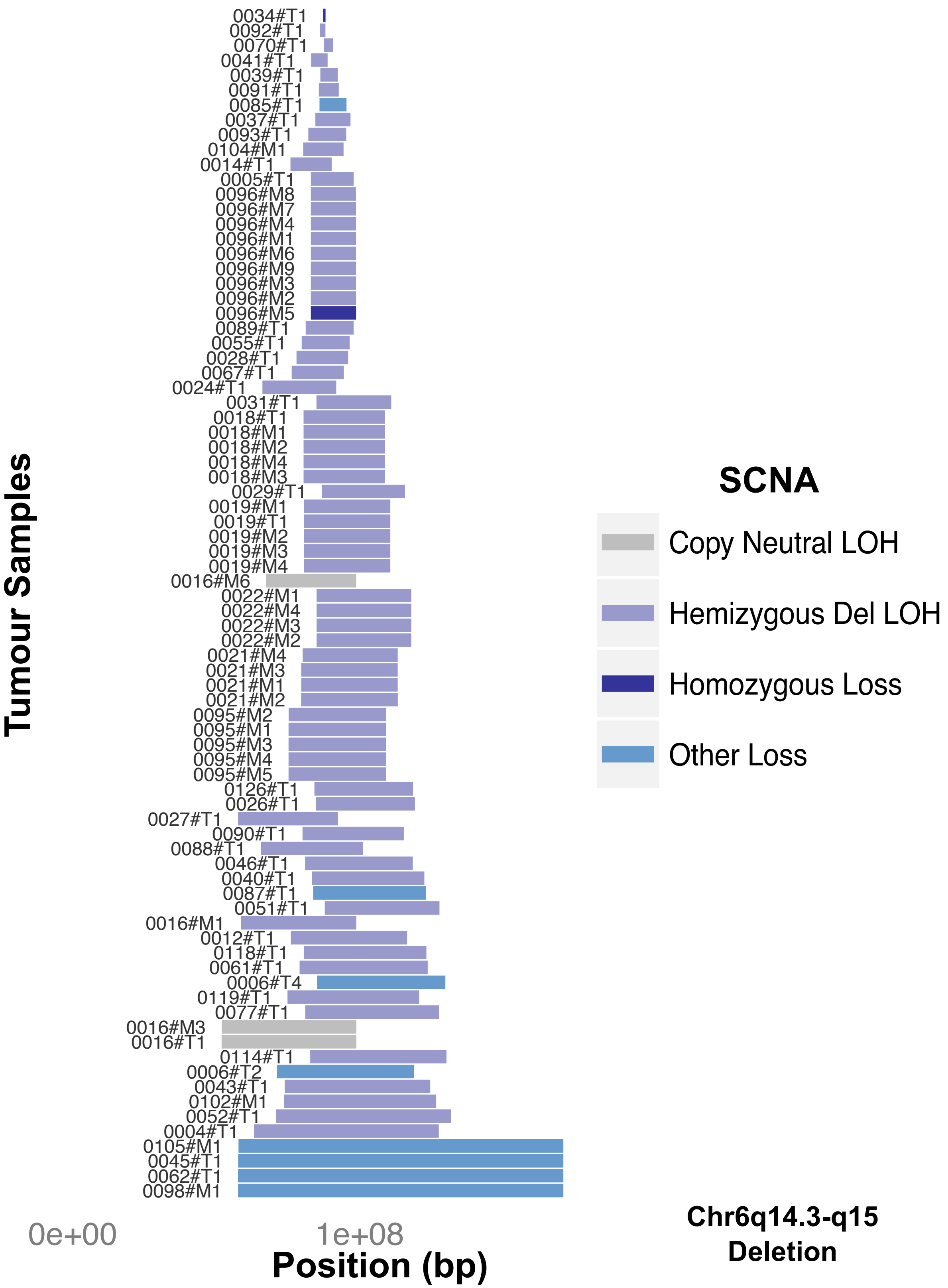

Chr6q14.3-q15 Deletion

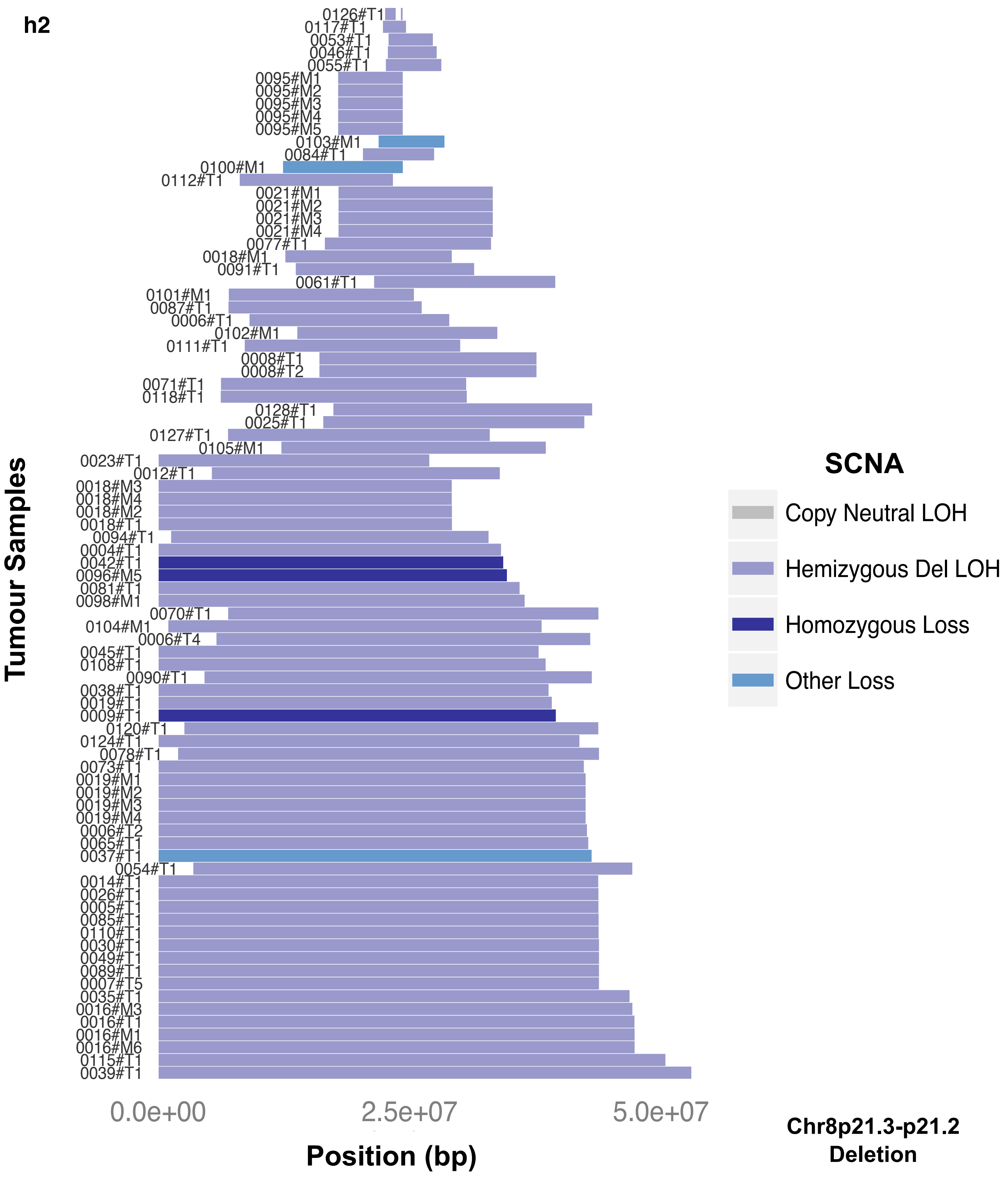
h2
Tumour Samples
Position (bp)
0.0e+00
2.5e+07
5.0e+07
SCNA
Copy Neutral LOH
Hemizygous Del LOH
Homozygous Loss
Other Loss
0126#T1
0117#T1
0053#T1
0046#T1
0055#T1
0095#M1
0095#M2
0095#M3
0095#M4
0095#M5
0103#M1
0084#T1
0100#M1
0112#T1
0021#M1
0021#M2
0021#M3
0021#M4
0077#T1
0018#M1
0091#T1
0061#T1
0101#M1
0087#T1
0006#T1
0102#M1
0111#T1
0008#T1
0008#T2
0071#T1
0118#T1
0128#T1
0025#T1
0127#T1
0105#M1
0023#T1
0012#T1
0018#M3
0018#M4
0018#M2
0018#T1
0094#T1
0004#T1
0042#T1
0096#M5
0081#T1
0098#M1
0070#T1
0104#M1
0006#T4
0045#T1
0108#T1
0090#T1
0038#T1
0019#T1
0009#T1
0120#T1
0124#T1
0078#T1
0073#T1
0019#M1
0019#M2
0019#M3
0019#M4
0006#T2
0065#T1
0037#T1
0054#T1
0014#T1
0026#T1
0005#T1
0085#T1
0110#T1
0030#T1
0049#T1
0089#T1
0007#T5
0035#T1
0016#M3
0016#T1
0016#M1
0016#M6
0115#T1
0039#T1
Chr8p21.3-p21.2
Deletion

i2

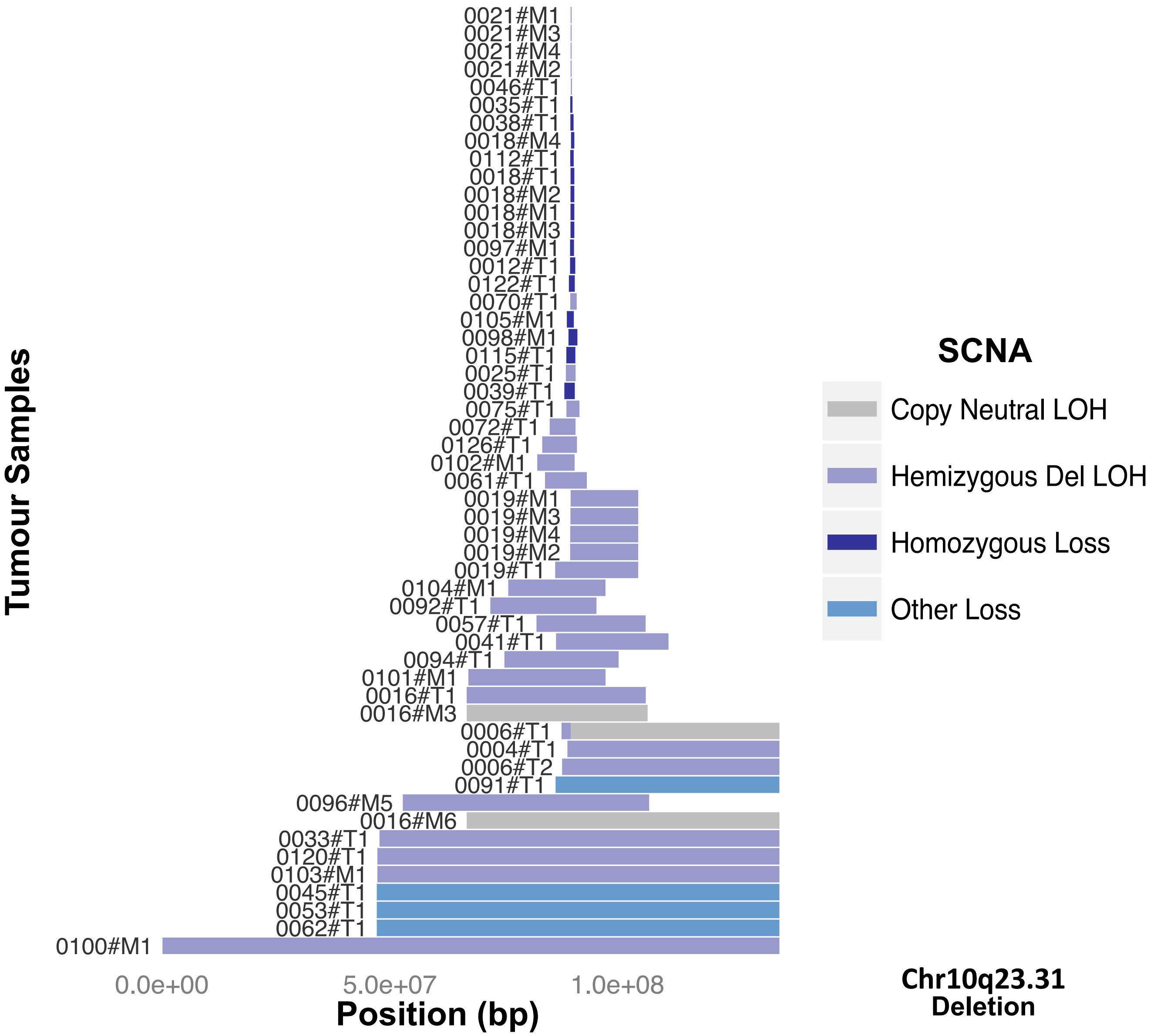
Tumour Samples
0021#M1
0021#M3
0021#M4
0021#M2
0046#T1
0035#T1
0038#T1
0018#M4
0112#T1
0018#T1
0018#M2
0018#M1
0018#M3
0097#M1
0012#T1
0122#T1
0070#T1
0105#M1
0098#M1
0115#T1
0025#T1
0039#T1
0075#T1
0072#T1
0126#T1
0102#M1
0061#T1
0019#M1
0019#M3
0019#M4
0019#M2
0019#T1
0104#M1
0092#T1
0057#T1
0041#T1
0094#T1
0101#M1
0016#T1
0016#M3
0006#T1
0004#T1
0006#T2
0091#T1
0096#M5
0016#M6
0033#T1
0120#T1
0103#M1
0045#T1
0053#T1
0062#T1
0100#M1
0.0e+00
5.0e+07
1.0e+08
Position (bp)
SCNA
Copy Neutral LOH
Hemizygous Del LOH
Homozygous Loss
Other Loss
Chr10q23.31
Deletion

j2

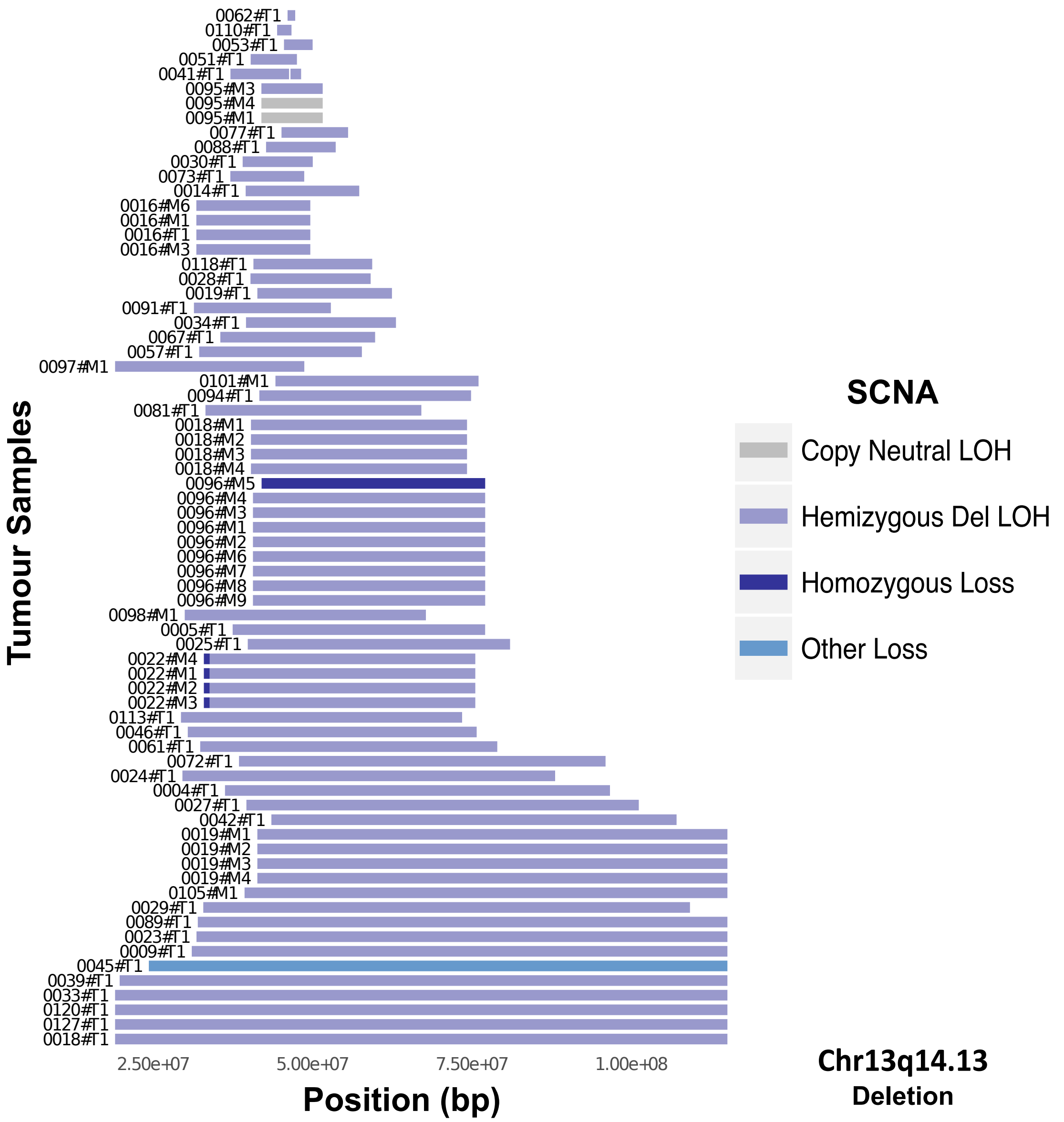
0062#T1
0110#T1
0053#T1
0051#T1
0041#T1
0095#M3
0095#M4
0095#M1
0077#T1
0088#T1
0030#T1
0073#T1
0014#T1
0016#M6
0016#M1
0016#T1
0016#M3
0118#T1
0028#T1
0019#T1
0091#T1
0034#T1
0067#T1
0057#T1
0097#M1
0101#M1
0094#T1
0081#T1
0018#M1
0018#M2
0018#M3
0018#M4
0096#M5
0096#M4
0096#M3
0096#M1
0096#M2
0096#M6
0096#M7
0096#M8
0096#M9
0098#M1
0005#T1
0025#T1
0022#M4
0022#M1
0022#M2
0022#M3
0113#T1
0046#T1
0061#T1
0072#T1
0024#T1
0004#T1
0027#T1
0042#T1
0019#M1
0019#M2
0019#M3
0019#M4
0105#M1
0029#T1
0089#T1
0023#T1
0009#T1
0045#T1
0039#T1
0033#T1
0120#T1
0127#T1
0018#T1
Tumour Samples
2.50e+07
5.00e+07
7.50e+07
1.00e+08
Position (bp)
SCNA
Copy Neutral LOH
Hemizygous Del LOH
Homozygous Loss
Other Loss
Chr13q14.13
Deletion

k2

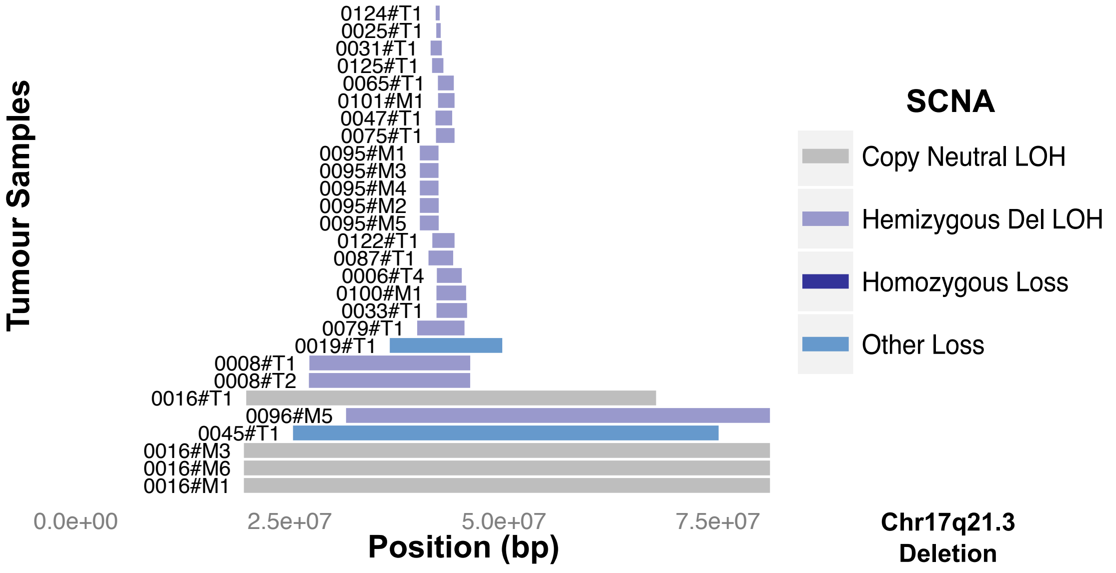
0124#T1
0025#T1
0031#T1
0125#T1
0065#T1
0101#M1
0047#T1
0075#T1
0095#M1
0095#M3
0095#M4
0095#M2
0095#M5
0122#T1
0087#T1
0006#T4
0100#M1
0033#T1
0079#T1
0019#T1
0008#T1
0008#T2
0016#T1
0096#M5
0045#T1
0016#M3
0016#M6
0016#M1
Tumour Samples
0.0e+00
2.5e+07
5.0e+07
7.5e+07
Position (bp)
SCNA
Copy Neutral LOH
Hemizygous Del LOH
Homozygous Loss
Other Loss
Chr17q21.3
Deletion

I2

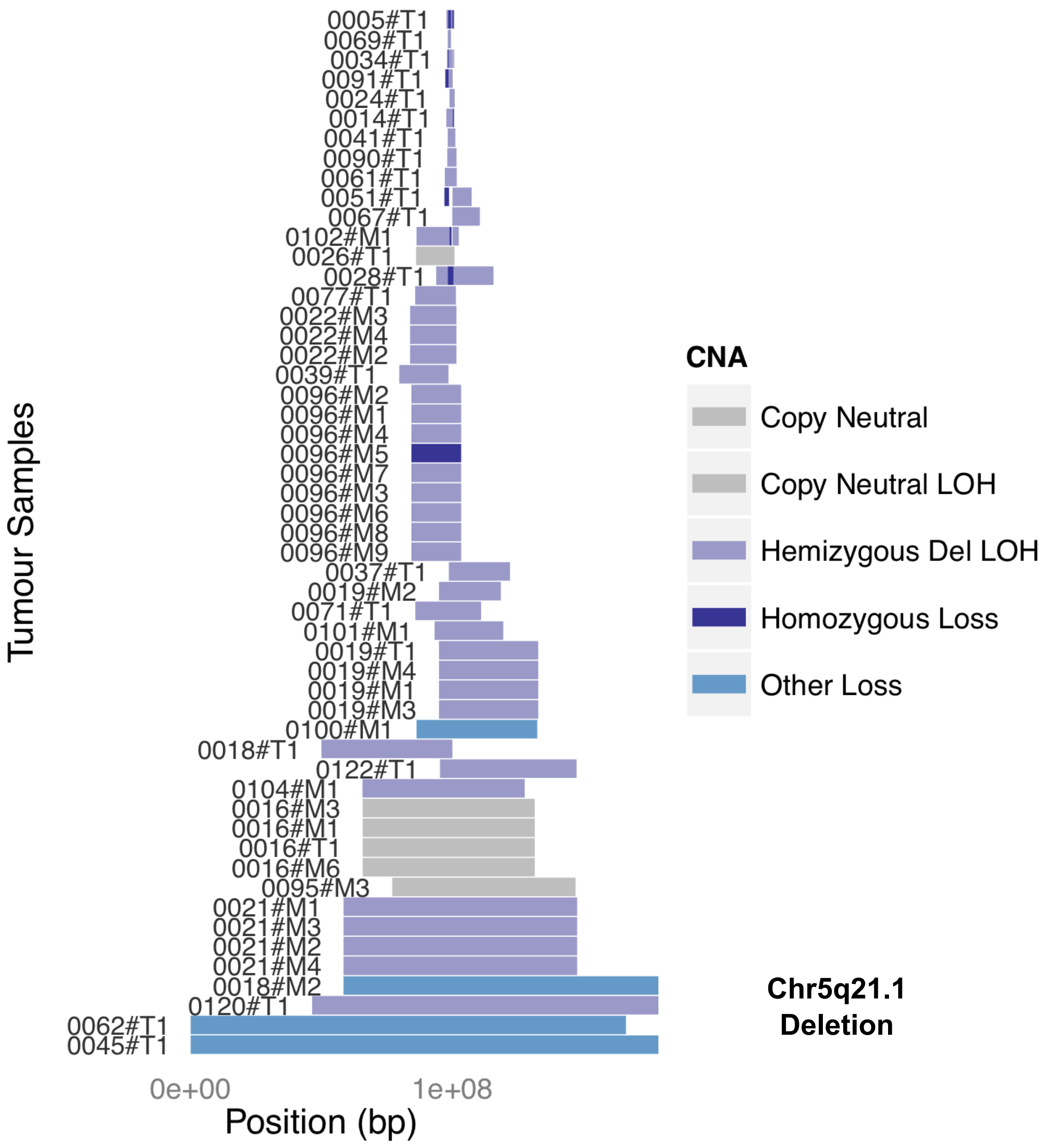

Chr5q21.1 Deletion

m2

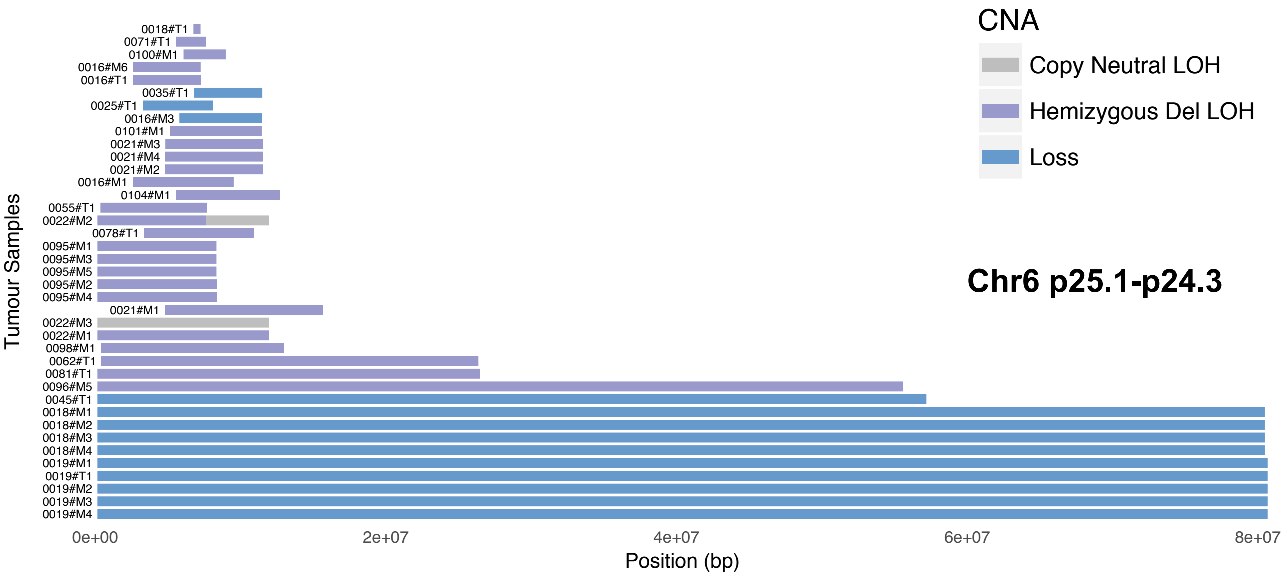
CNA
Copy Neutral LOH
Hemizygous Del LOH
Loss
Chr6 p25.1-p24.3
Tumour Samples
0018#T1
0071#T1
0100#M1
0016#M6
0016#T1
0035#T1
0025#T1
0016#M3
0101#M1
0021#M3
0021#M4
0021#M2
0016#M1
0104#M1
0055#T1
0022#M2
0078#T1
0095#M1
0095#M3
0095#M5
0095#M2
0095#M4
0021#M1
0022#M3
0022#M1
0098#M1
0062#T1
0081#T1
0096#M5
0045#T1
0018#M1
0018#M2
0018#M3
0018#M4
0019#M1
0019#T1
0019#M2
0019#M3
0019#M4
0e+00
2e+07
4e+07
6e+07
8e+07
Position (bp)

n2

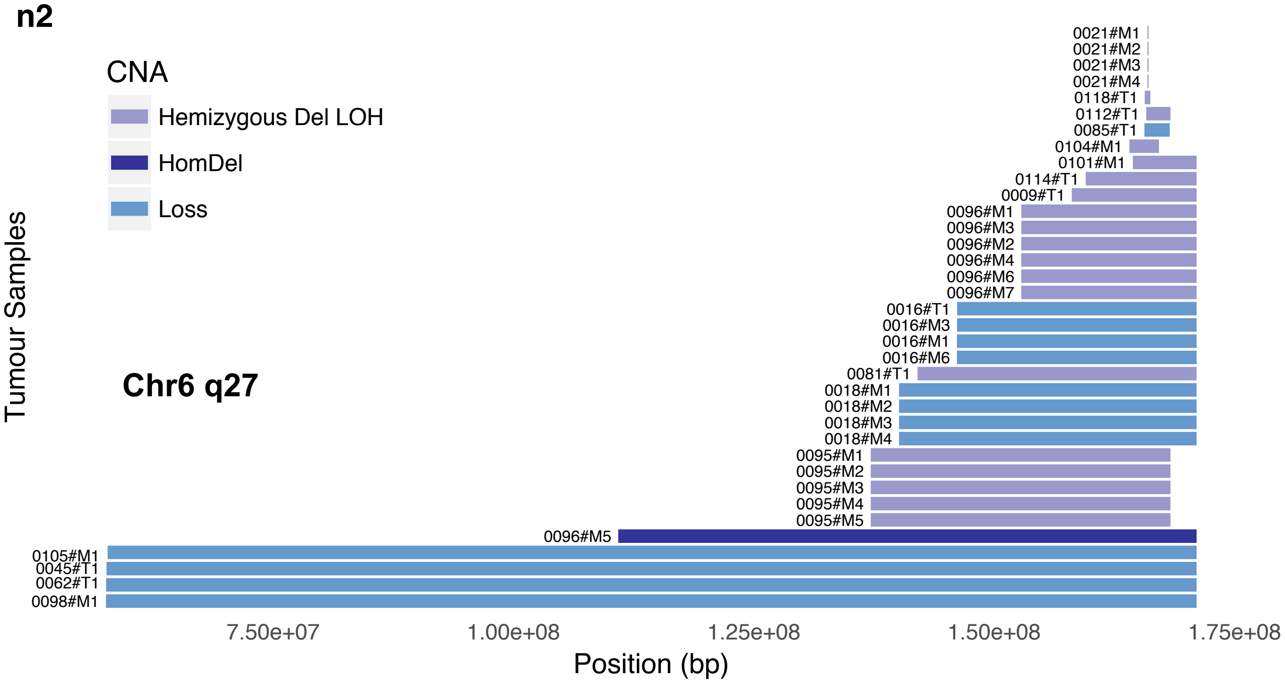
CNA
Hemizygous Del LOH
HomDel
Loss
Chr6 q27
Tumour Samples
0021#M1
0021#M2
0021#M3
0021#M4
0118#T1
0112#T1
0085#T1
0104#M1
0101#M1
0114#T1
0009#T1
0096#M1
0096#M3
0096#M2
0096#M4
0096#M6
0096#M7
0016#T1
0016#M3
0016#M1
0016#M6
0081#T1
0018#M1
0018#M2
0018#M3
0018#M4
0095#M1
0095#M2
0095#M3
0095#M4
0095#M5
0096#M5
0105#M1
0045#T1
0062#T1
0098#M1
7.50e+07
1.00e+08
1.25e+08
1.50e+08
1.75e+08
Position (bp)
