# Supplementary material for: Appraising the relevance of DNA copy number loss and gain in prostate cancer using whole genome DNA sequence data
Source: PLoS Genet. 2017 Sep 25;13(9):e1007001. doi: 10.1371/journal.pgen.1007001 (PMC5628936; doi:10.1371/journal.pgen.1007001)
Supplement: S1 Table — (DOCX) [file pgen.1007001.s007.docx]

**S1 Table.** Classification of Somatic Copy Number Alterations.

| **Chr1:22** |  |  |
| --- | --- | --- |
| **Category** | **Subcategory** | **Copy number type assignment criteria** |
| Normal |  | (nMinor = nMajor) & ((nMinor + nMajor) = ploidy) |
| Amplification | Amp LOH | (nMinor = 0) & (nMajor > ploidy) |
| Amplification | Gain | (nMinor + nMajor) > ploidy |
| Deletion | Copy neutral | (nMinor != nMajor) & ((nMinor + nMajor) = ploidy) |
| Deletion | Copy neutral LOH | (nMinor = 0) & (nMajor = ploidy) |
| Deletion | Hemizygous deletion LOH | (nMinor = 0) & (nMajor < ploidy) & (nMajor > 0) |
| Deletion | Other Loss | (nMinor + nMajor) < ploidy |
| Deletion | Homozygous loss | (nMinor = 0) & (nMajor = 0) |
|  |  |  |
| **ChrX** |  |  |
| **Category** | **Subcategory** | **Copy number type assignment criteria** |
| Normal |  | (nMinor = 0) & (nMajor = ploidy) |
| Amplification |  | (nMinor = 0) & (nMajor > ploidy) |
| Deletion |  | (nMinor = 0) & (nMajor < ploidy) |

**Key**

| != | Does not equal |
| --- | --- |
| nMinor | copy number of minor allele |
| nMajor | copy number of major allele |
| ploidy | copy number of genome overall |
