# Supplementary material for: Appraising the relevance of DNA copy number loss and gain in prostate cancer using whole genome DNA sequence data
Source: PLoS Genet. 2017 Sep 25;13(9):e1007001. doi: 10.1371/journal.pgen.1007001 (PMC5628936; doi:10.1371/journal.pgen.1007001)
Supplement: S4 Table — Left side: Samples classified by disease status i.e. samples from prostatectomies from patients free of metastatic disease (PT) or samples from patients with metastatic disease (M). Right side: Prostatectomy samples where there was at least six months follow up (n = 69) classified by whether there was rapid biochemical recurrence within six months of prostatectomy (PG) or not (RF) (right side). The two TURP samples are not summarized in this table. Results from statistical tests are shown that test whether there are significant differences between either metastatic and prostatectomy patients or prostatectomy patients that have biochemical recurrence within six months or not. (DOCX) [file pgen.1007001.s010.docx]

**S4 Table.** Summary characteristics of the genomes and somatic copy number alterations (SCNAs). Left side: Samples classified by disease status i.e. samples from prostatectomies from patients free of metastatic disease (PT) or samples from patients with metastatic disease (M). Right side: Prostatectomy samples where there was at least six months follow up (n = 69) classified by whether there was rapid biochemical recurrence within six months of prostatectomy (PG) or not (RF) (right side). The two TURP samples are not summarized in this table. Results from statistical tests are shown that test whether there are significant differences between either metastatic and prostatectomy patients or prostatectomy patients that have biochemical recurrence within six months or not.

|  | **Classification based on metastasis** | | **Classification based on biochemical recurrence at 6 months** | |
| --- | --- | --- | --- | --- |
|  | **Prostatectomy Patients (PT) (86)** | **Metastatic Patients (M) (15)** | **Relapse Free Patients (RF) (59)** | **Progressed Patients (PG) (10)** |
| **Ploidy** |  |  |  |  |
| 1.5-2.5 (diploid) | 70 (81%) | 6 (40%) | 49 (83%) | 6 (60%) |
| 2.5-3.5 (triploid) | 0 (0%) | 1 (7%) | 0 (0%) | 0 (0%) |
| 3.5-4.5 (tetraploid) | 16 (19%) | 8 (53%) | 10 (17%) | 4 (40%) |
|  | Fisher’s exact test, PT vs M, *p* = 0.0042 | | Fisher’s exact test, RF vs PG, *p* = 0.1091 | |
| **Number of SCNAs** |  |  |  |  |
| Min | 0 | 42 | 0 | 13 |
| Median | 16 | 84 | 14 | 31 |
| Mean | 21 | 83 | 19 | 44 |
| Max | 109 | 186 | 71 | 109 |
|  | Mann-Whitney *U*, PT vs M, *p =* 2.165x10^-08^ | | Mann-Whitney *U*, RF vs PG, *p* = 0.0133 | |
| **Length SCNAs (Mb)** |  |  |  |  |
| Min | 0.014 | 0.013 | 0.014 | 0.023 |
| Median | 1.998 | 2.775 | 2.744 | 1.063 |
| Mean | 10.140 | 11.210 | 11.530 | 7.966 |
| Max | 180.800 | 197.800 | 180.800 | 118.300 |
|  | Mann-Whitney *U*, PT vs M, *p* = 4.371x10^-04^ | | Mann-Whitney *U*, RF vs PG, *p* = 2.05x10^-11^ | |
| **Type of SCNAs**  **(mean per patient)** |  |  |  |  |
| Amplification | 6 | 26 | 6 | 9 |
| Deletion | 19 | 57 | 18 | 36 |
|  | Fisher’s exact test, PT vs M, *p* = 0.6194 | | Fisher’s exact test, PT vs M, *p* = 0.76 | |
