# Supplementary material for: Appraising the relevance of DNA copy number loss and gain in prostate cancer using whole genome DNA sequence data
Source: PLoS Genet. 2017 Sep 25;13(9):e1007001. doi: 10.1371/journal.pgen.1007001 (PMC5628936; doi:10.1371/journal.pgen.1007001)
Supplement: S5 Table — (DOCX) [file pgen.1007001.s011.docx]

**S5 Table.** Summary of clinico-pathological characteristics of the patients in the defined sets of clusters.

|  | **Clusters** | | | | |
| --- | --- | --- | --- | --- | --- |
|  | **C1** | **C2** | **C3** | **C4** | **C5** |
| **Number of Patients** | 28 | 39 | 16 | 13 | 7 |
| **Progression** |  |  |  |  |  |
| **(6 months follow up)** |  |  |  |  |  |
| Progressed (10) | 0 | 6 | 2 | 2 | 0 |
| Relapse free patients (59) | 20 | 23 | 11 | 4 | 1 |
| Not available | 8 | 7 | 1 | 1 | 0 |
| **Metastatic Disease** |  |  |  |  |  |
| Metastatic (16) | 0 | 3 | 2 | 6 | 5 |
| Non-Metastatic (87) | 28 | 36 | 14 | 7 | 2 |
| **PSA at diagnosis (ng/ml)** |  |  |  |  |  |
| <4 | 0 | 2 | 0 | 0 | 0 |
| 4–10 | 20 | 20 | 10 | 6 | 0 |
| >10 | 8 | 14 | 4 | 2 | 2 |
| Not available | 0 | 3 | 2 | 5 | 5 |
| **Gleason score** |  |  |  |  |  |
| 3+3 (6) | 4 | 3 | 0 | 0 | 0 |
| 3+4 (7) | 19 | 25 | 8 | 3 | 1 |
| 4+3 (7) | 4 | 7 | 5 | 4 | 0 |
| 3+5 (8) | 1 | 1 | 0 | 0 | 0 |
| 4+5 (9) | 0 | 0 | 1 | 0 | 0 |
| 5+4 (9) | 0 | 0 | 0 | 1 | 1 |
| Not available | 0 | 3 | 2 | 5 | 5 |
| **Clinical stage** |  |  |  |  |  |
| T1 | 16 | 20 | 3 | 3 | 0 |
| T2 | 4 | 6 | 4 | 2 | 0 |
| T3 | 0 | 1 | 0 | 0 | 0 |
| T4 | 0 | 0 | 0 | 0 | 0 |
| Tx | 8 | 9 | 0 | 3 | 2 |
| Not available | 0 | 3 | 2 | 5 | 5 |
