# Supplementary material for: Appraising the relevance of DNA copy number loss and gain in prostate cancer using whole genome DNA sequence data
Source: PLoS Genet. 2017 Sep 25;13(9):e1007001. doi: 10.1371/journal.pgen.1007001 (PMC5628936; doi:10.1371/journal.pgen.1007001)
Supplement: S9 Table — (DOCX) [file pgen.1007001.s015.docx]

**S9 Table.** Mutations in promoter regions of genes within the minimal regions of alteration.

| **Sample** | **Chr** | **Position** | **Type** | **Promoter** | **Promoter region with mutation and SCNA in the same patient** |
| --- | --- | --- | --- | --- | --- |
| PD11331c | 18 | 72917467 | Sub | ZADH2_2 | FALSE |
| PD11335a | 20 | 62205592 | Del | HELZ2_1 | FALSE |
| PD13395a | 20 | 60758100 | Sub | MTG2_1 | FALSE |
| PD13412d | 8 | 23564071 | Sub | NKX2-6_1 | TRUE |
| PD14711a | 12 | 106696594 | Ins | TCP11L2_1 | FALSE |
| PD14717a | 17 | 7387694 | Sub | POLR2A_1 | FALSE |
| PD14728a | 11 | 72929335 | Sub | P2RY2_1 | FALSE |
| PD7445e | 5 | 179247907 | Sub | SQSTM1_3 | FALSE |
| PD9169a | 11 | 74951786 | Sub | TPBGL_1 | TRUE |
