# Supplementary material for: Appraising the relevance of DNA copy number loss and gain in prostate cancer using whole genome DNA sequence data
Source: PLoS Genet. 2017 Sep 25;13(9):e1007001. doi: 10.1371/journal.pgen.1007001 (PMC5628936; doi:10.1371/journal.pgen.1007001)
Supplement: S12 Table — (DOCX) [file pgen.1007001.s018.docx]

**S12 Table.** List of regions of homozygous loss that occur in greater than two patients.

| **Chromosomal location** | **Number of patients** | **Genes in Region** |
| --- | --- | --- |
| Chr10:89643014-90075877 | 11 | *PTEN, RNLS* |
| Chr5:98246652-98522541 | 6 | *CHD1* |
| Chr8:25417422-26386565 | 5 | *EBF2, PPP2R2A, BNIP3L, PNMA2, DPYSL2* |
| Chr5:100108038-100331392 | 4 | *ST8SIA4* |
| Chr3:176710694-176912497 | 3 | *TBL1XR1* |
| Chr5:90620914-91834830 | 3 | *ARRDC3* |
| Chr5:92191841-93058810 | 3 | *NR2F1, FAM172A* |
| Chr5:108723417-109385888 | 3 | *PJA2, MAN2A1, AC011366.3* |
| Chr8:31254-34302399 | 3 | *NKX3.1 and greater 50 others.* |
| Chr13:48718746-49924527 | 3 | *ITM2B, RB1, LPAR6, RCBTB2, CYSLTR2, FNDC3A, MLNR, CDADC1, CAB39L* |
| Chr2:25675712-26076426 | 2 | *ASXL2, DTNB* |
| Chr2:138289875-138564480 | 2 | *THSD7B* |
| Chr2:140727573-141421683 | 2 | *LRP1B* |
| Chr2:141882095-142498384 | 2 | *LRP1B* |
| Chr3:177422141-178044879 | 2 | *KCNMB2* |
| Chr4:178215123-179157773 | 2 | *NEIL3, AGA, LINC01098* |
| Chr5:55099427-56260339 | 2 | *DDX4, IL31RA, AC008914.1, IL6ST, ANKRD55, CTC-236F12.4, AC022431.2, MAP3K1, SETD9, MIER3* |
| Chr5:56380357-58217472 | 2 | *GPBP1, ACTBL2, PLK2, GAPT, CTD-2117L12.1, RAB3C* |
| Chr5:58290945-58569764 | 2 | *PDE4D* |
| Chr5:58714964-59169335 | 2 | *PDE4D* |
| Chr5:102372607-103277876 | 2 | *GIN1, PPIP5K2, C5orf30, NUDT12* |
| Chr6:87340866-88128509 | 2 | *HTR1E, CGA, ZNF292, GJB7, SMIM8, RP1-102H19.8, C6orf163, C6orf164, C6ORF165, C6orf165* |
| Chr6:95988486-96199591 | 2 | *MANEA* |
| Chr6:139258567-140092629 | 2 | *REPS1, ABRACL, HECA, TXLNB, CITED2* |
| Chr13:48445251-48578558 | 2 | *SUCLA2* |
| Chr13:71253907-73265917 | 2 | *DACH1* |
| Chr13:74426332-75903401 | 2 | *KLF12, AL355390.1, TBC1D4* |
| Chr17:6333920-20759553 | 2 | *TP53 and greater than 50 others* |
| Chr17:44394414-45255529 | 2 | *ARL17B, LRRC37A, LRRC37A2, ARL17A, NSF, WNT3, WNT9B, GOSR2, RP11-156P1.2, RPRML, CDC27* |
| Chr21:42842666-42858015 | 2 | *TMPRSS2* |
