# Supplementary material for: Appraising the relevance of DNA copy number loss and gain in prostate cancer using whole genome DNA sequence data
Source: PLoS Genet. 2017 Sep 25;13(9):e1007001. doi: 10.1371/journal.pgen.1007001 (PMC5628936; doi:10.1371/journal.pgen.1007001)
Supplement: S17 Table — (DOCX) [file pgen.1007001.s023.docx]

**S17 Table.** Summary table of the GISTIC detected deletions that follow the Knudson Hit Model.

| **GISTIC Alteration Band** | **Gene/Region** | **Chromosomal Location** | **Number of Patients with Deletion** | **Homozygous Deletion** | **Number of Patients affected by Deletion and a Point Mutation/Indel on opposite allele** | **Number of Patients affected by Deletion and another Breakpoint** |
| --- | --- | --- | --- | --- | --- | --- |
| 1q42.12 | *TP53* | Chr1:208416154-241297899 | 37 | 2 | 9 | 5 |
| 2q22.1 | *NXPH2 & SPOPL* | Chr2:139259371-139537918 | 14 | 0 | 0 | 5 |
| 2q22.1 | *HNMT* | Chr2:138721590-138773930 | 13 | 0 | 0 | 4 |
| 3p13 | *RYBP* | Chr3:72420976-72496069 | 34 | 0 | 0 | 5 |
| 3p13 | *SHQ1* | Chr3:72798428-72911065 | 33 | 0 | 0 | 4 |
| 3p24.3 | *UBE2E2* | Chr3:23244511-23633284 | 13 | 0 | 0 | 5 |
| 3p24.3 | *ZNF385D* | Chr3:21459915-22414812 | 14 | 0 | 0 | 4 |
| 5q13.2 | 5 genes inc. *MAP1B, SV2C* | Chr5:71403061-75649764 | 22 | 0 | 0 | 4 |
| 5q21.1 | *CHD1* | Chr5:98190908-98262240 | 29 | 0 | 0 | 8 |
| 5q21.1 | *SLCO4C1, SLCO6A1, ST8SIA4, AC008948.1* | Chr5:100142639-101834720 | 26 | 0 | 0 | 7 |
| 5q21.1 | *NUDT12 & PAM* | Chr5:102089685-102898494 | 24 | 0 | 0 | 5 |
| 6q14.3 | 35 genes inc. *ANKRD6, BACH2, CNR1, GJB7, HTR1E, MAP3K7, NT5E, PM20D2, RNGTT, RRAGD, SLC35A1, UBE2J1, ZNF292* | Chr6:86159809-91296764 | 49 | 0 | 0 | 14 |
| 6q14.3 | *EPHA7* | Chr6:93949738-94129265 | 45 | 0 | 0 | 13 |
| 6q14.3 | *TBX18* | Chr6:85397069-85474237 | 43 | 0 | 0 | 11 |
| 6q14.3 | *KIAA1009* | Chr6:84833960-84937353 | 41 | 0 | 0 | 10 |
| 8p21.3 | 7 genes | Chr8:22298332-22526661 | 60 | 0 | 0 | 9 |
| 10q23.31 | *PTEN* | chr10:89617158-90034038 | 39 | 11 | 7 | 9 |
| 10q23.31 | *RNLS* | Chr10:90033621-90344287 | 37 | 0 | 0 | 7 |
| 10q23.31 | *KLLN* | Chr10:89618918-89623194 | 37 | 0 | 0 | 4 |
| 11q23.2 | *ZBTB16* | Chr11:113930315-114121398 | 16 | 0 | 0 | 5 |
| 11q23.2 | *HTR3A* | Chr11:113845603-113861035 | 16 | 0 | 0 | 4 |
| 12p13.2 | *DUSP16, LOH12CR1, LOH12CR2* | Chr12:12508342-12715317 | 27 | 0 | 0 | 4 |
| 13q14.11 | 11 genes inc. *CAB39L, ENOX1, LCP1, LECT1, PCDH8, RB1, TSC22D1* | Chr13:43597339-53422775 | 44 | 0 | 0 | 9 |
| 13q14.11 | 5 genes inc. *PRR20B, PRR20C* | Chr13:57715052-57744352 | 37 | 0 | 0 | 6 |
