# Supplementary material for: Appraising the relevance of DNA copy number loss and gain in prostate cancer using whole genome DNA sequence data
Source: PLoS Genet. 2017 Sep 25;13(9):e1007001. doi: 10.1371/journal.pgen.1007001 (PMC5628936; doi:10.1371/journal.pgen.1007001)
Supplement: S18 Table — (DOCX) [file pgen.1007001.s024.docx]

**S18 Table.** Summary of cases and copy number platforms.

| **Case Type** | **aCGH SNP6.0 array (Affymetrix)** | **Whole Genome DNA Sequencing (BGI, Illumina)** |
| --- | --- | --- |
| **Radical Prostatectomy (88)** | 3 (10 tumour and 3 matched blood samples) | 85 (83 patients with one tumoru and matched blood samples each and two patients with 5 and 9 rumour and matched normal samples) |
| **Metastatic - Rapid Autopsy (15)** | 5 (22 tumour and 5 matched normal samples) | 10 (10 tumour and matched normal samples) |
| **Transurethral Resection of the Prostate (2)** | 0 | 2 (2 tumour and matched normal samples) |
