# Supplementary material for: Appraising the relevance of DNA copy number loss and gain in prostate cancer using whole genome DNA sequence data
Source: PLoS Genet. 2017 Sep 25;13(9):e1007001. doi: 10.1371/journal.pgen.1007001 (PMC5628936; doi:10.1371/journal.pgen.1007001)

S1 Appendix

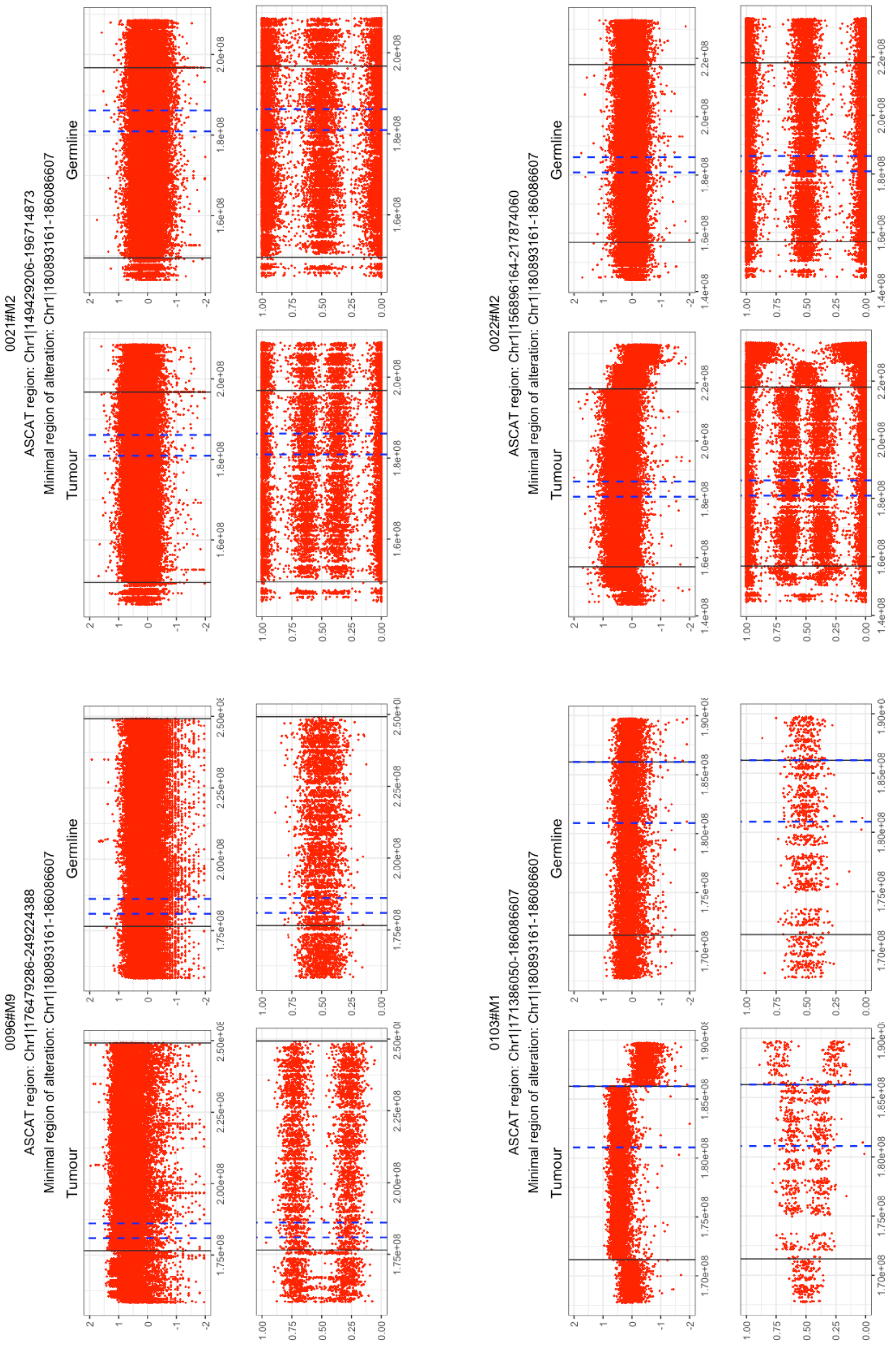
0021#M2
ASCAT region: Chr1|149429206-196714873
Minimal region of alteration: Chr1|180893161-186086607
Tumour
Germline
0022#M2
ASCAT region: Chr1|156896164-217874060
Minimal region of alteration: Chr1|180893161-186086607
Tumour
Germline
0096#M9
ASCAT region: Chr1|176479286-249224388
Minimal region of alteration: Chr1|180893161-186086607
Tumour
Germline
0103#M1
ASCAT region: Chr1|171386050-186086607
Minimal region of alteration: Chr1|180893161-186086607
Tumour
Germline

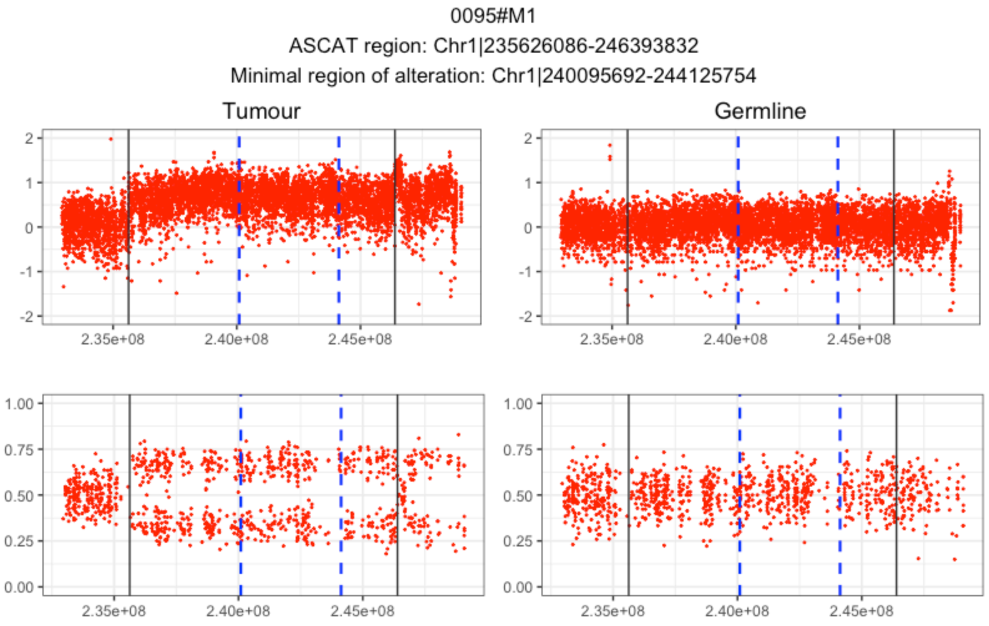
0095#M1
ASCAT region: Chr1|235626086-246393832
Minimal region of alteration: Chr1|240095692-244125754
Tumour
Germline

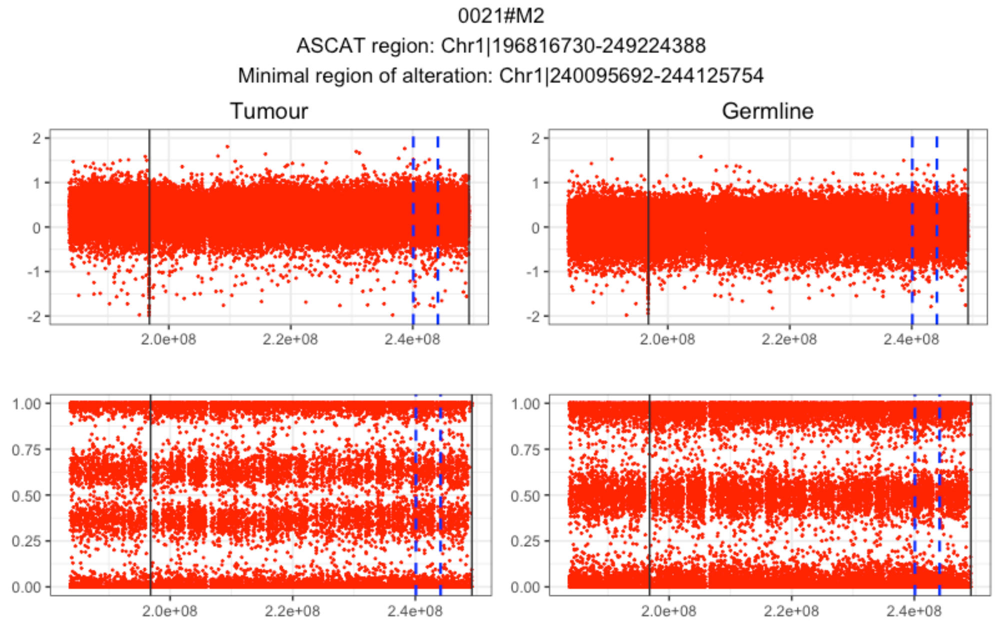
0021#M2
ASCAT region: Chr1|196816730-249224388
Minimal region of alteration: Chr1|240095692-244125754
Tumour
Germline

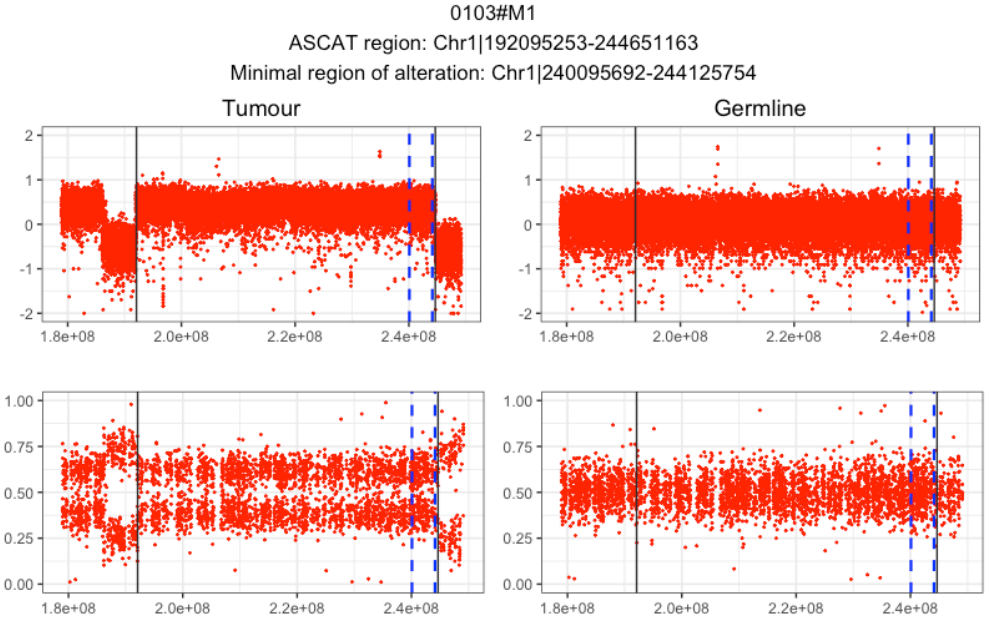
0103#M1
ASCAT region: Chr1|192095253-244651163
Minimal region of alteration: Chr1|240095692-244125754
Tumour
Germline

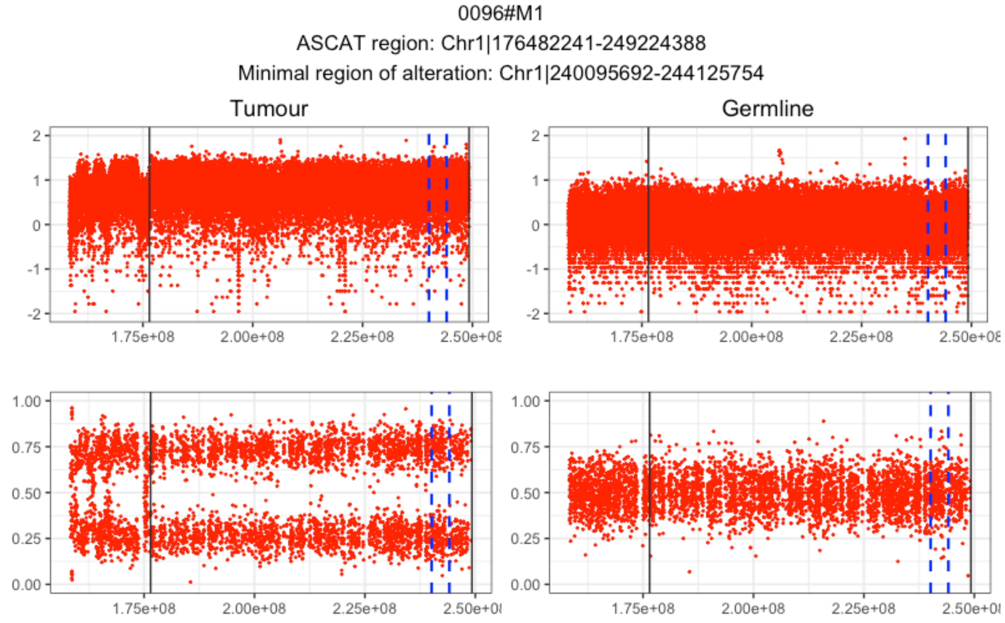
0096#M1
ASCAT region: Chr1|176482241-249224388
Minimal region of alteration: Chr1|240095692-244125754
Tumour
Germline

0101#M1
ASCAT region: Chr2|132881416-169963011
Minimal region of alteration: Chr2|168271595-168957571

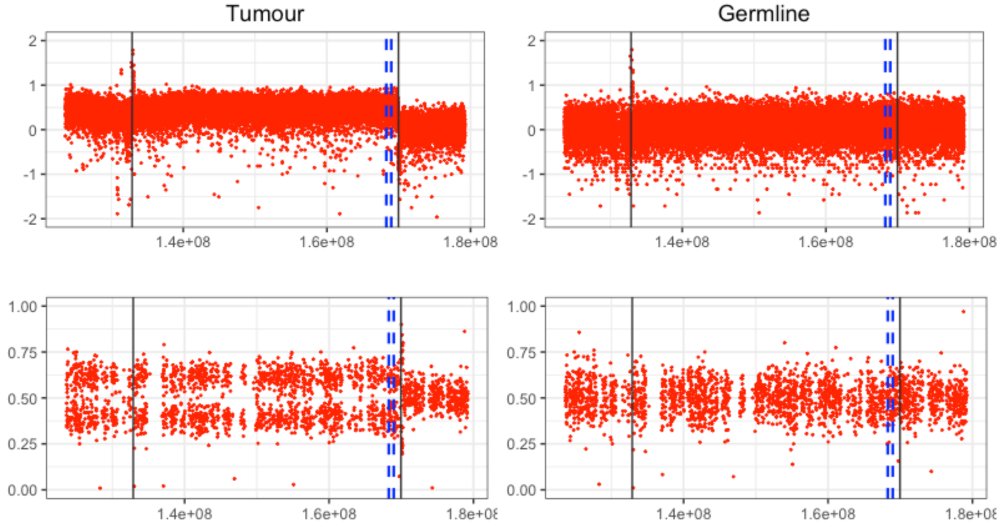

0019#M2
ASCAT region: Chr2|165745814-169156907
Minimal region of alteration: Chr2|168271595-168957571

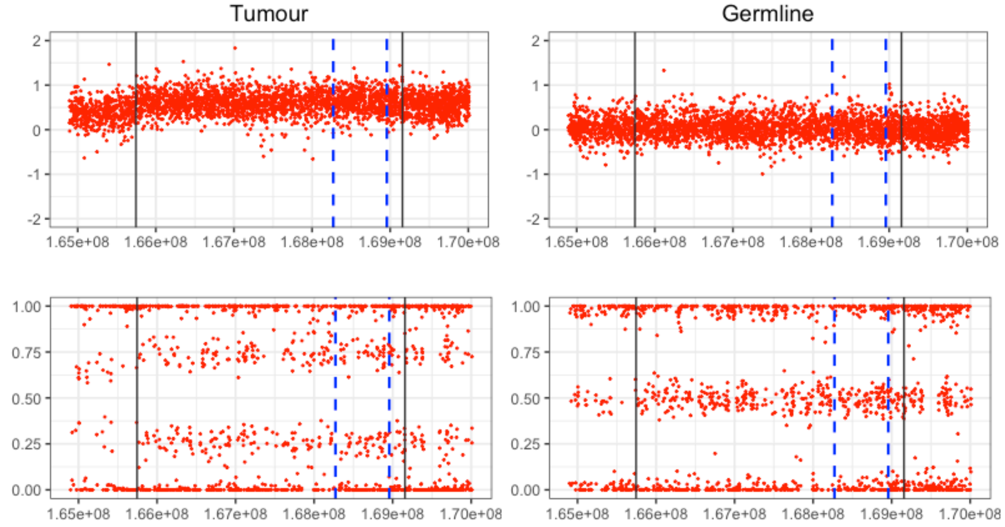

0104#M1
ASCAT region: Chr2|168271595-170262735
Minimal region of alteration: Chr2|168271595-168957571

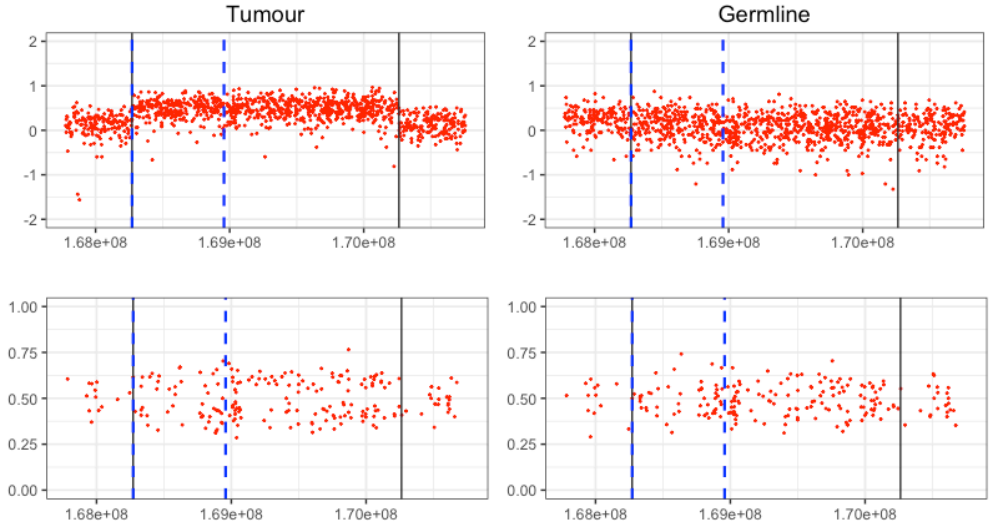

0095#M3
ASCAT region: Chr2|167083311-174812716
Minimal region of alteration: Chr2|168271595-168957571

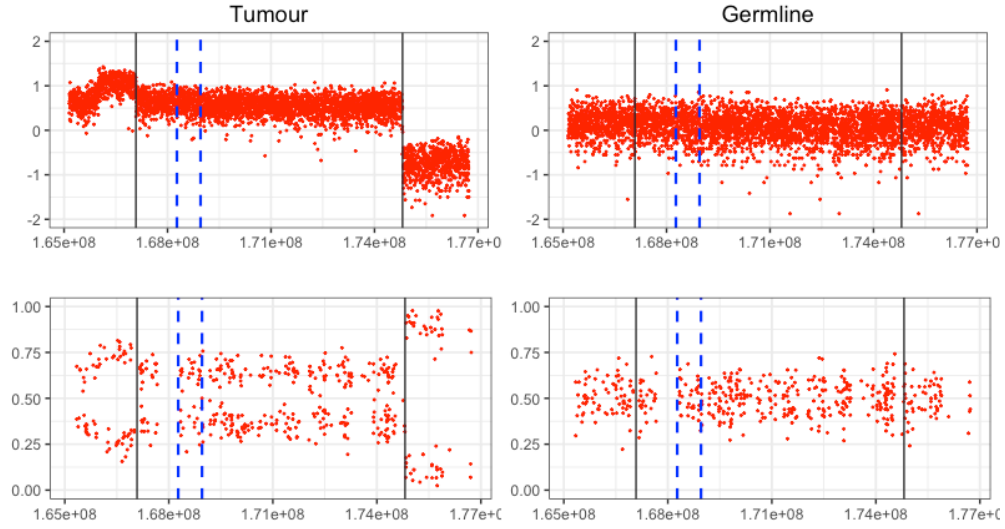

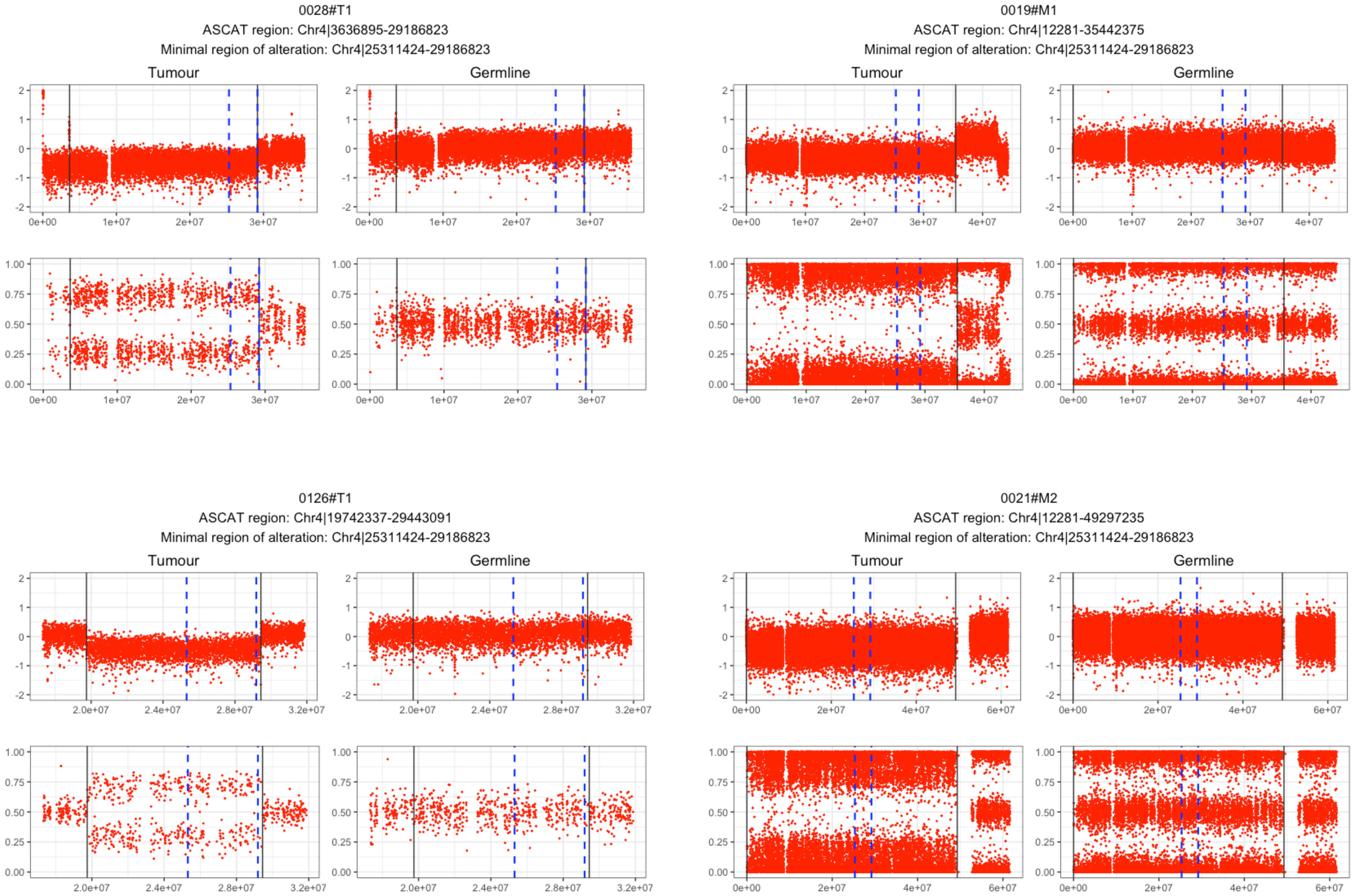
0028#T1
ASCAT region: Chr4|3636895-29186823
Minimal region of alteration: Chr4|25311424-29186823
Tumour
Germline
0019#M1
ASCAT region: Chr4|12281-35442375
Minimal region of alteration: Chr4|25311424-29186823
Tumour
Germline
0126#T1
ASCAT region: Chr4|19742337-29443091
Minimal region of alteration: Chr4|25311424-29186823
Tumour
Germline
0021#M2
ASCAT region: Chr4|12281-49297235
Minimal region of alteration: Chr4|25311424-29186823
Tumour
Germline

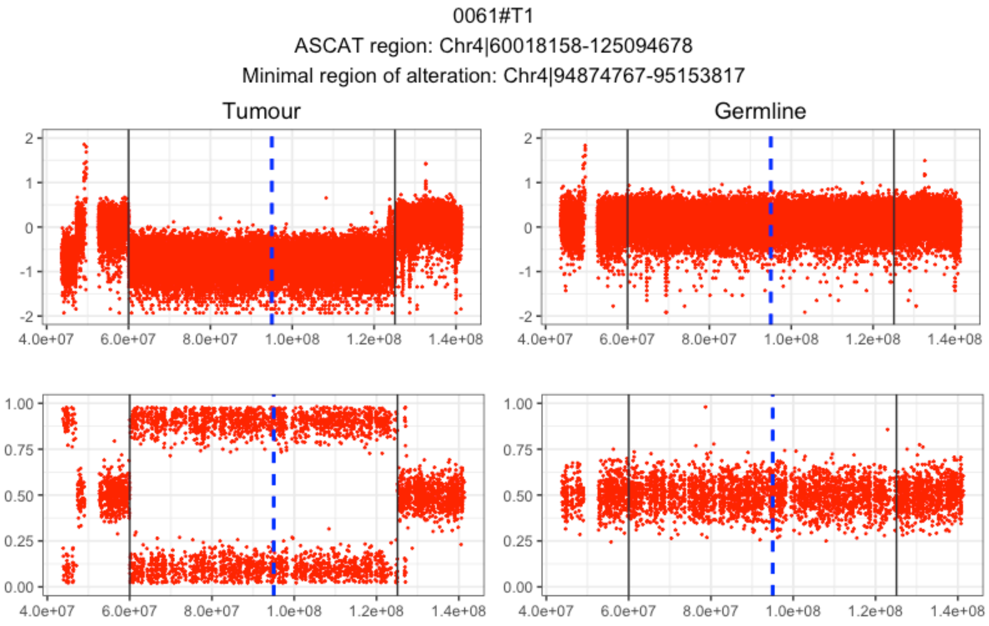
0061#T1
ASCAT region: Chr4|60018158-125094678
Minimal region of alteration: Chr4|94874767-95153817
Tumour
Germline

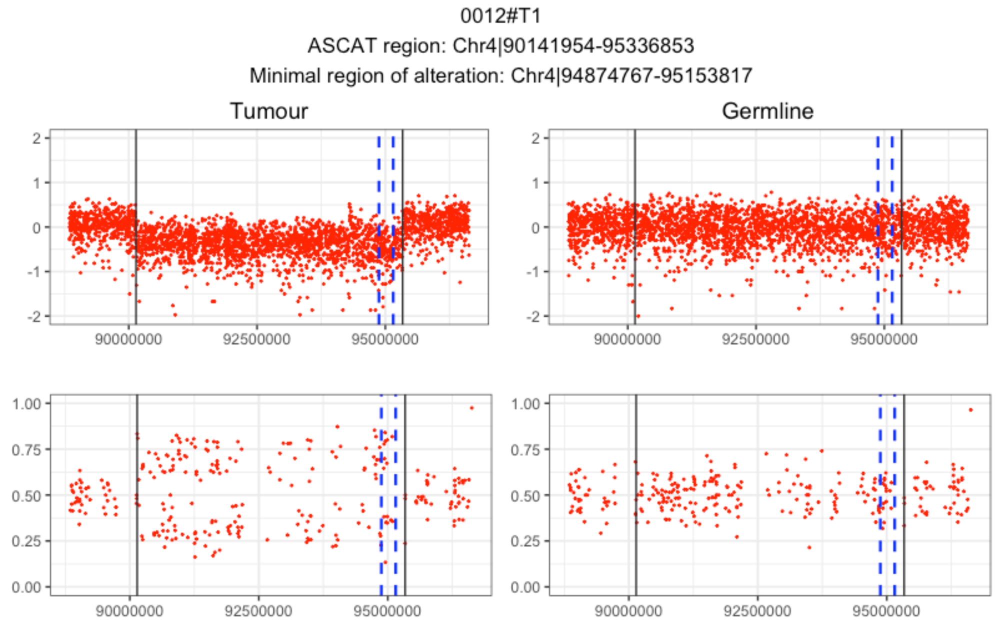
0012#T1
ASCAT region: Chr4|90141954-95336853
Minimal region of alteration: Chr4|94874767-95153817
Tumour
Germline

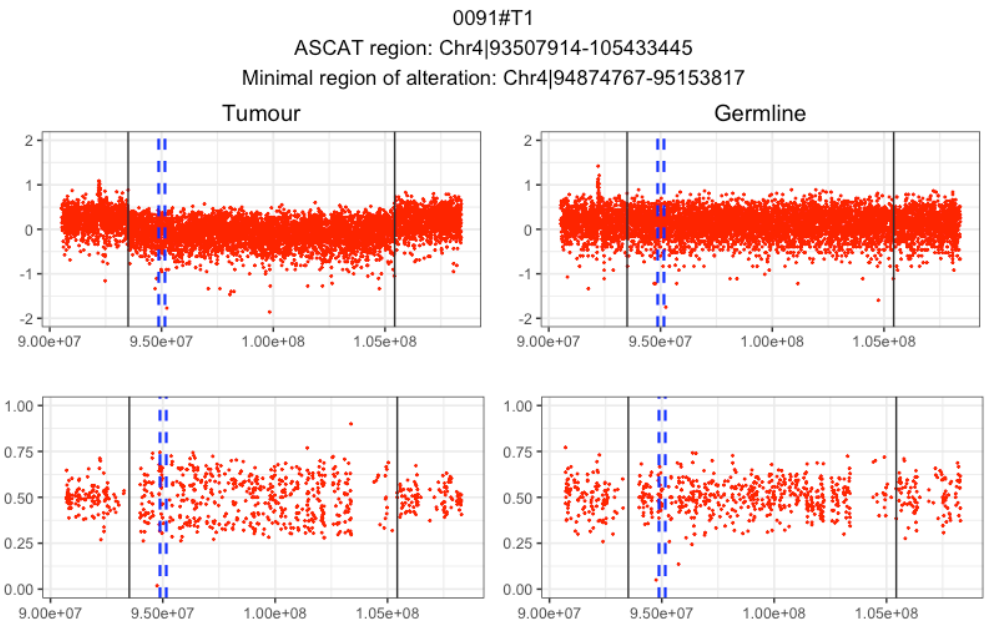
0091#T1
ASCAT region: Chr4|93507914-105433445
Minimal region of alteration: Chr4|94874767-95153817
Tumour
Germline

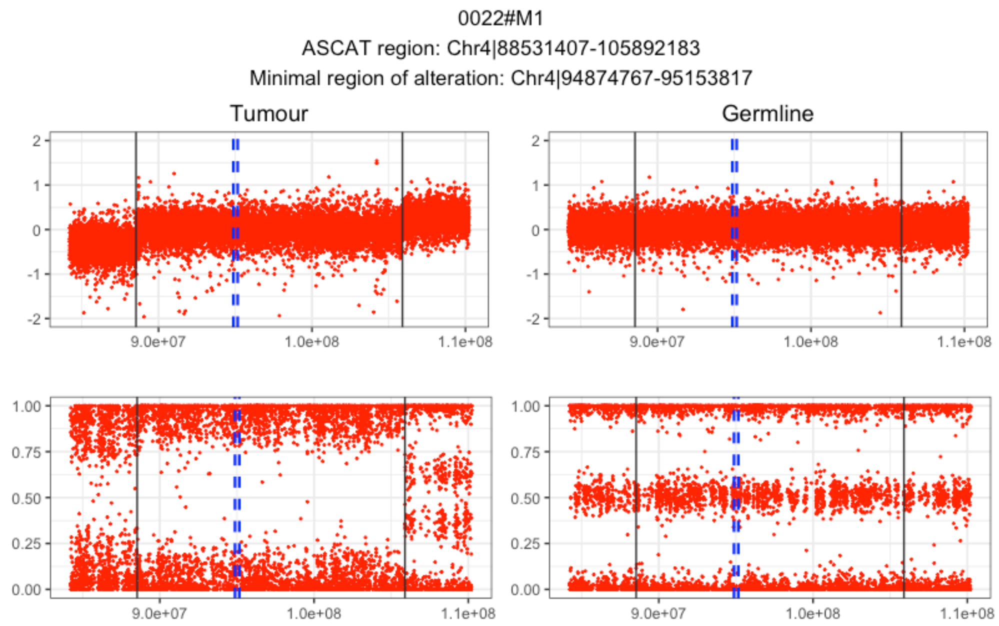
0022#M1
ASCAT region: Chr4|88531407-105892183
Minimal region of alteration: Chr4|94874767-95153817
Tumour
Germline

0034#T1
ASCAT region: Chr4|158694762-182128100
Minimal region of alteration: Chr4|177366483-179157773

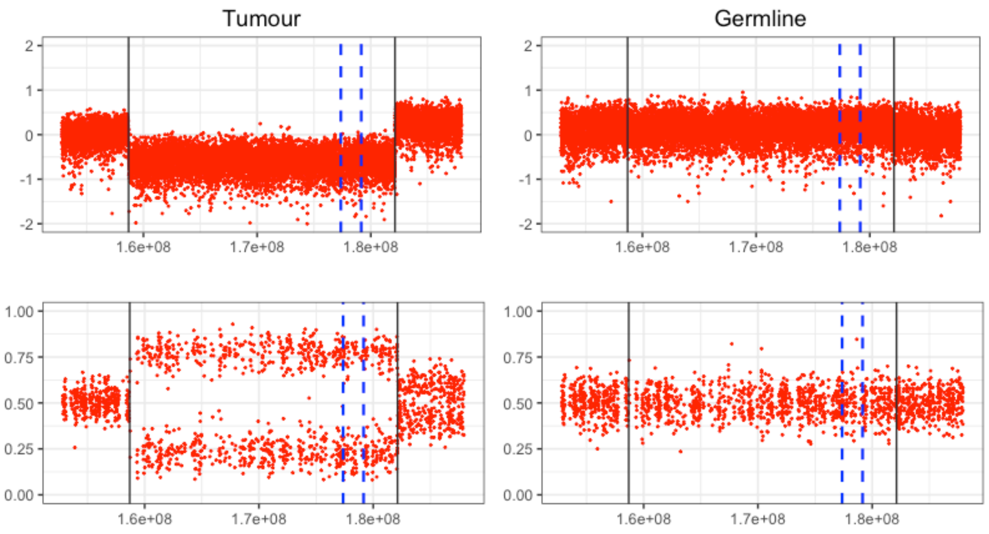

0022#M1
ASCAT region: Chr4|155314793-191027923
Minimal region of alteration: Chr4|177366483-179157773

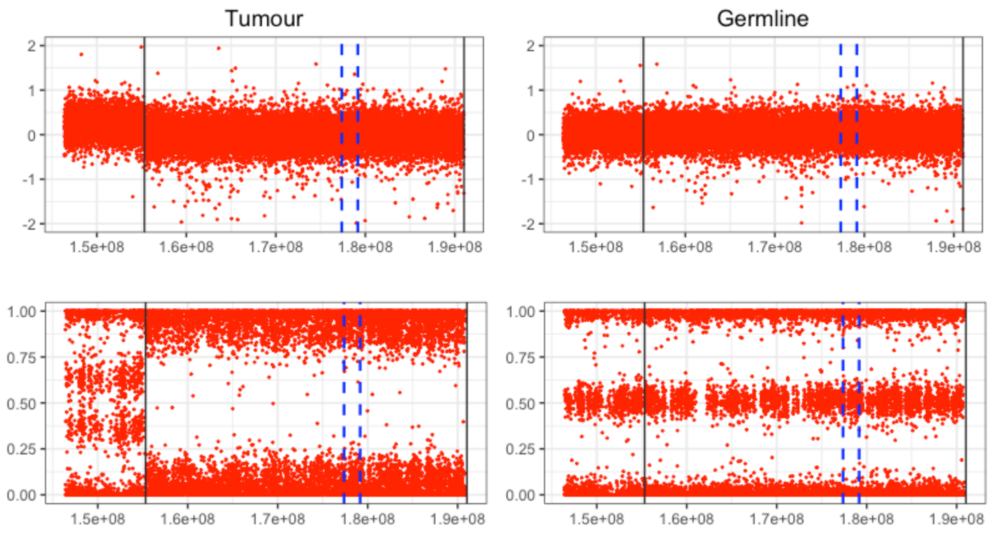

0095#M1
ASCAT region: Chr4|174748352-190464257
Minimal region of alteration: Chr4|177366483-179157773

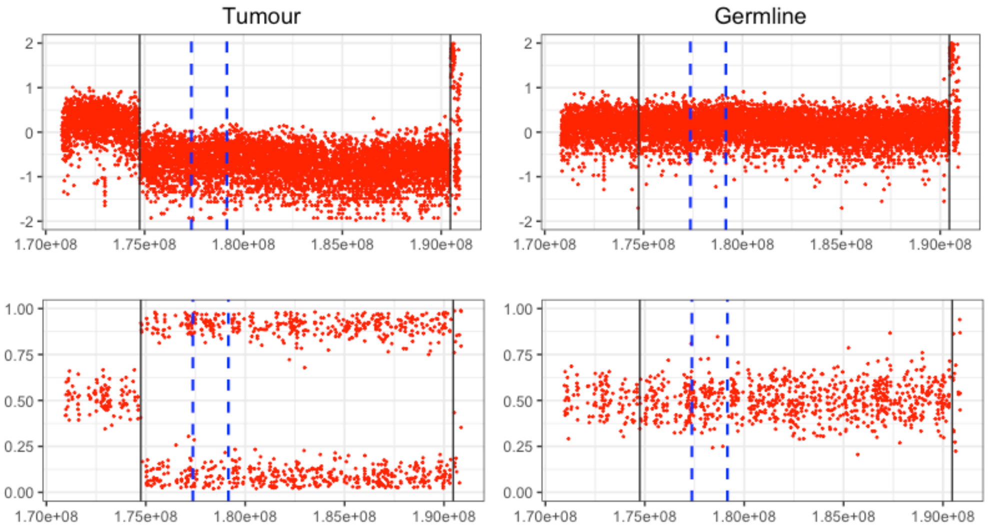

0096#M1
ASCAT region: Chr4|174086282-179155798
Minimal region of alteration: Chr4|177366483-179157773

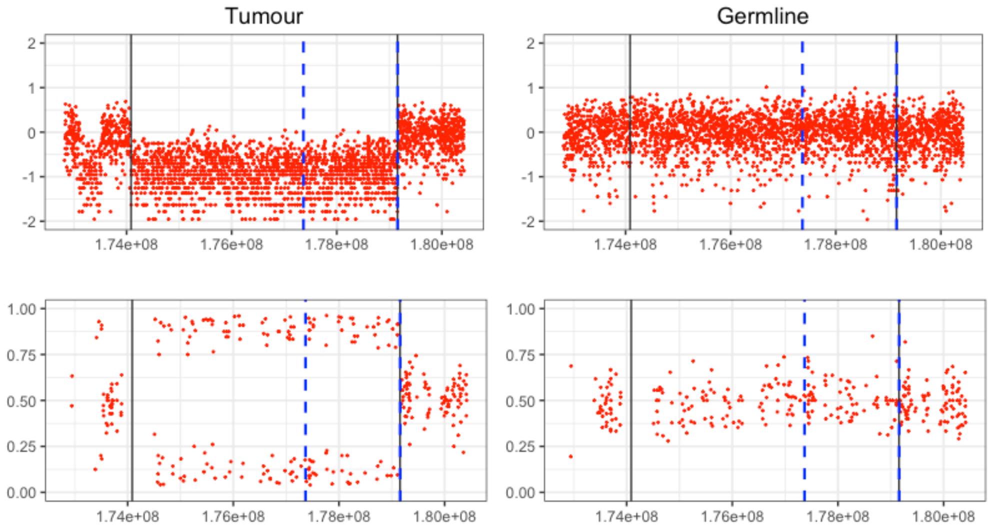

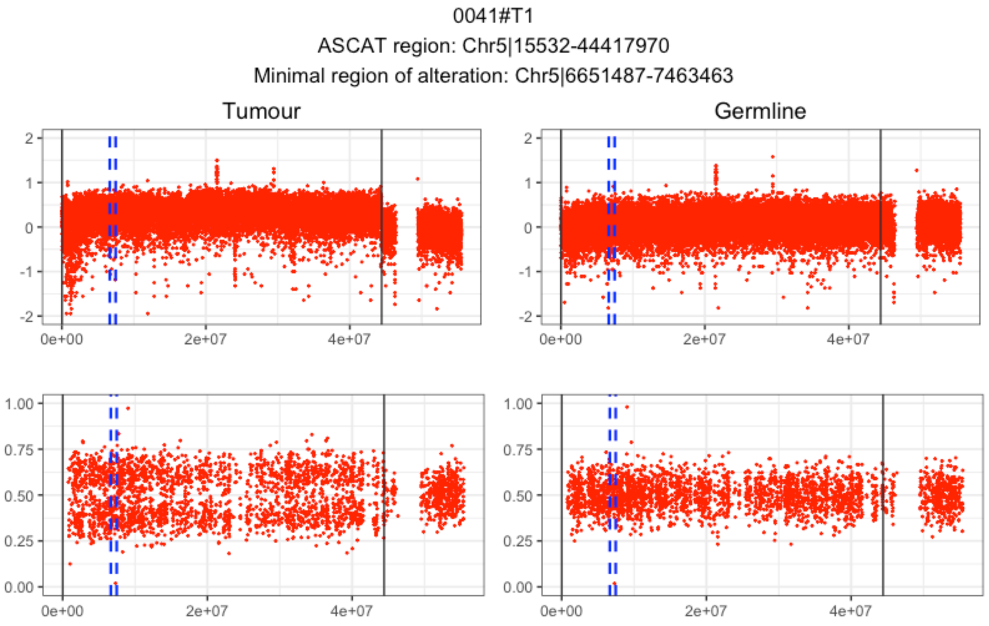
0041#T1
ASCAT region: Chr5|15532-44417970
Minimal region of alteration: Chr5|6651487-7463463
Tumour
Germline

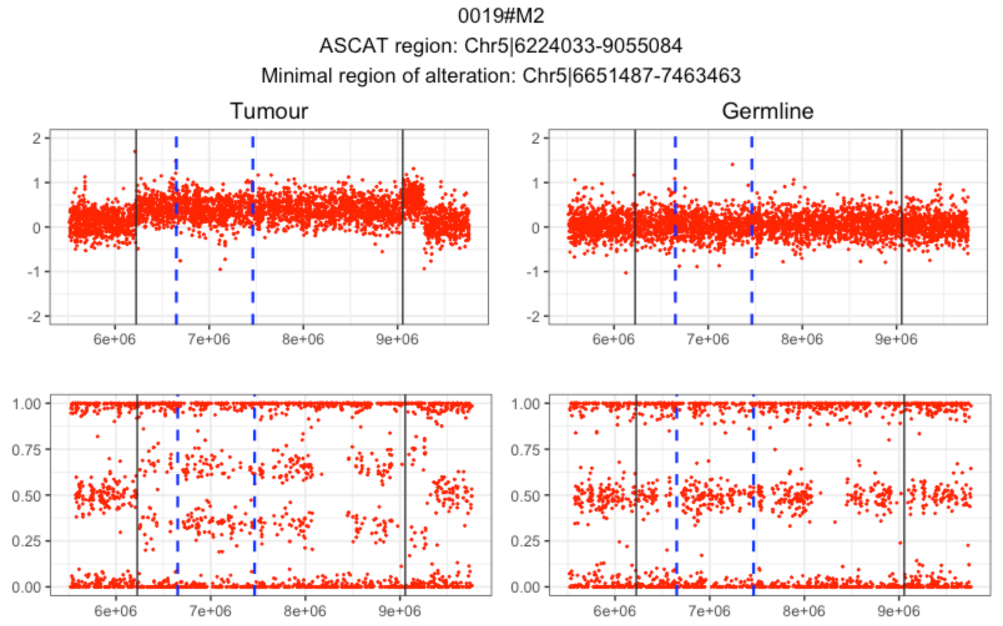
0019#M2
ASCAT region: Chr5|6224033-9055084
Minimal region of alteration: Chr5|6651487-7463463
Tumour
Germline

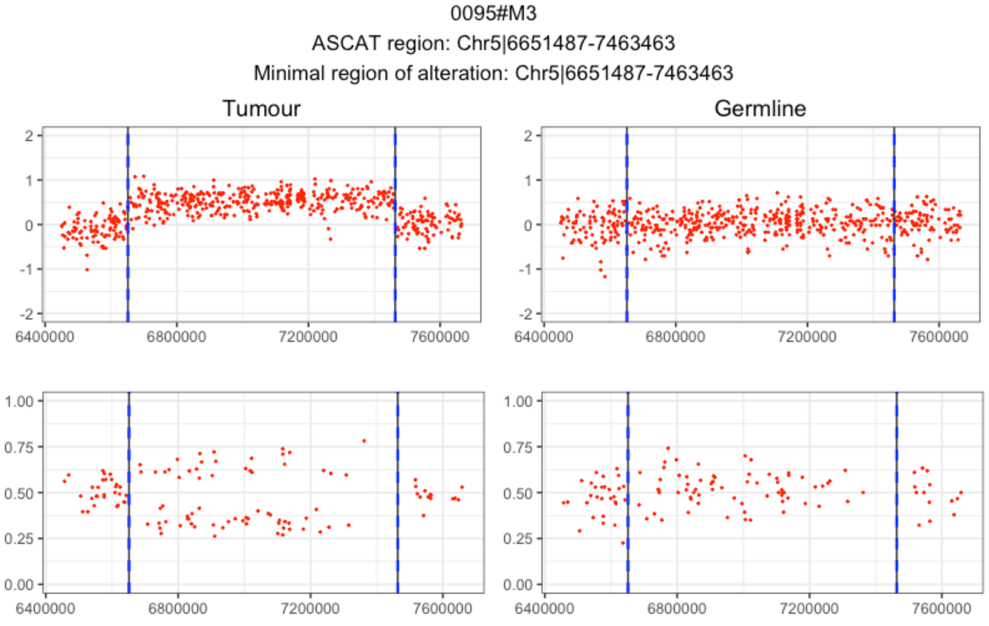
0095#M3
ASCAT region: Chr5|6651487-7463463
Minimal region of alteration: Chr5|6651487-7463463
Tumour
Germline

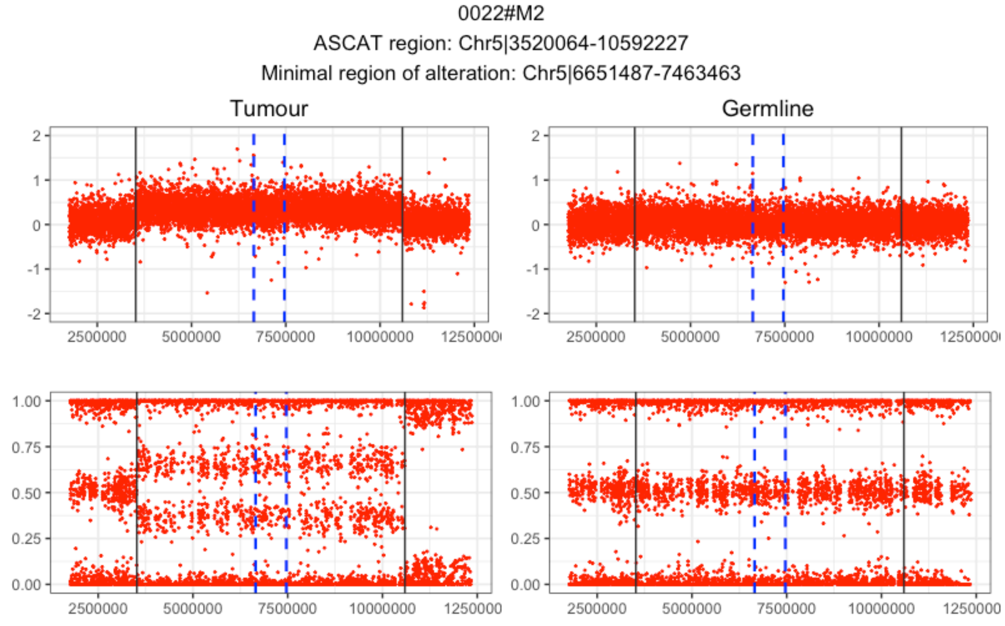
0022#M2
ASCAT region: Chr5|3520064-10592227
Minimal region of alteration: Chr5|6651487-7463463
Tumour
Germline

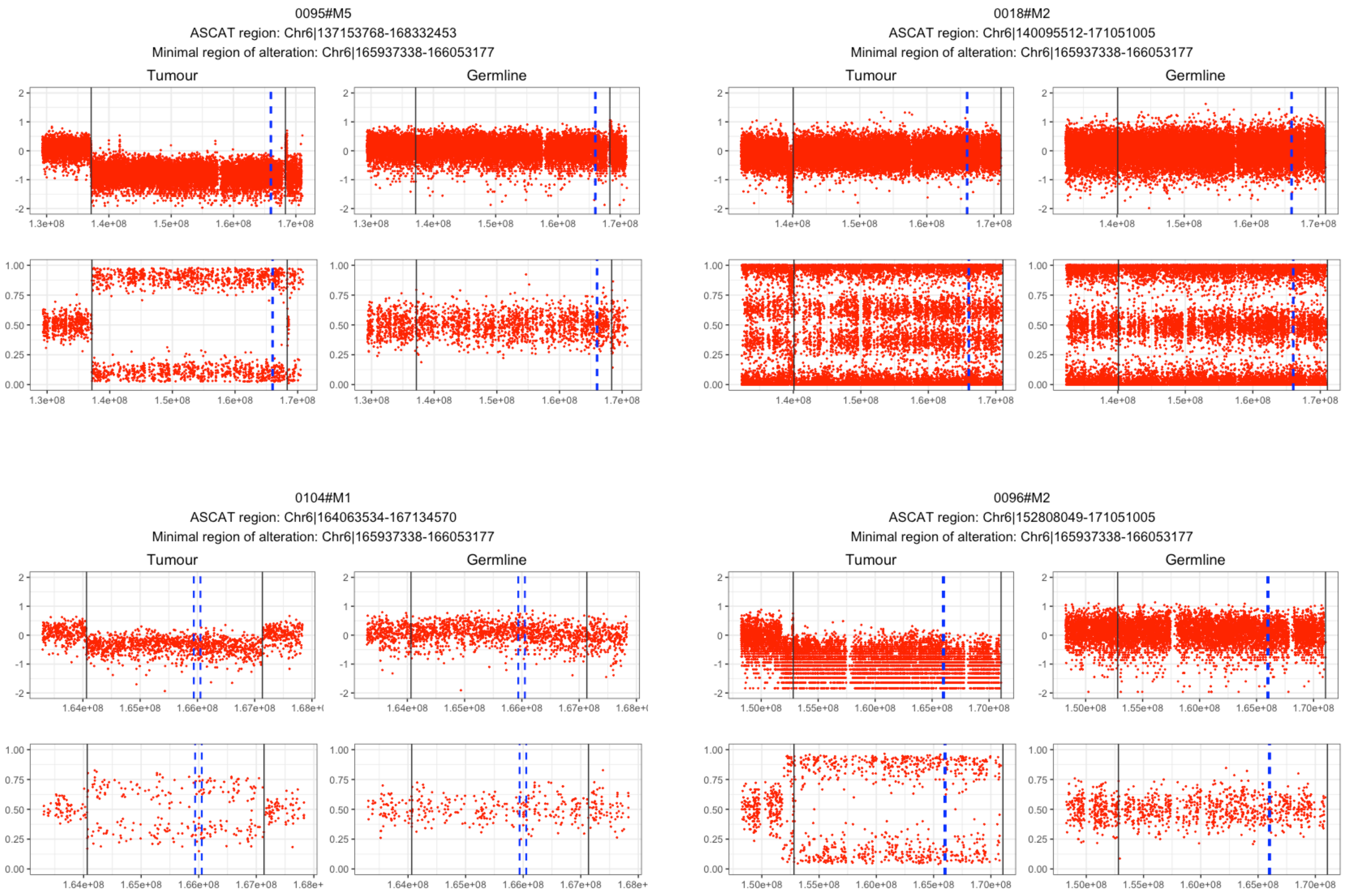
0095#M5
ASCAT region: Chr6|137153768-168332453
Minimal region of alteration: Chr6|165937338-166053177
Tumour
Germline
0018#M2
ASCAT region: Chr6|140095512-171051005
Minimal region of alteration: Chr6|165937338-166053177
Tumour
Germline
0104#M1
ASCAT region: Chr6|164063534-167134570
Minimal region of alteration: Chr6|165937338-166053177
Tumour
Germline
0096#M2
ASCAT region: Chr6|152808049-171051005
Minimal region of alteration: Chr6|165937338-166053177
Tumour
Germline

0095#M1
ASCAT region: Chr9|47463-47217176
Minimal region of alteration: Chr9|14100316-14960575

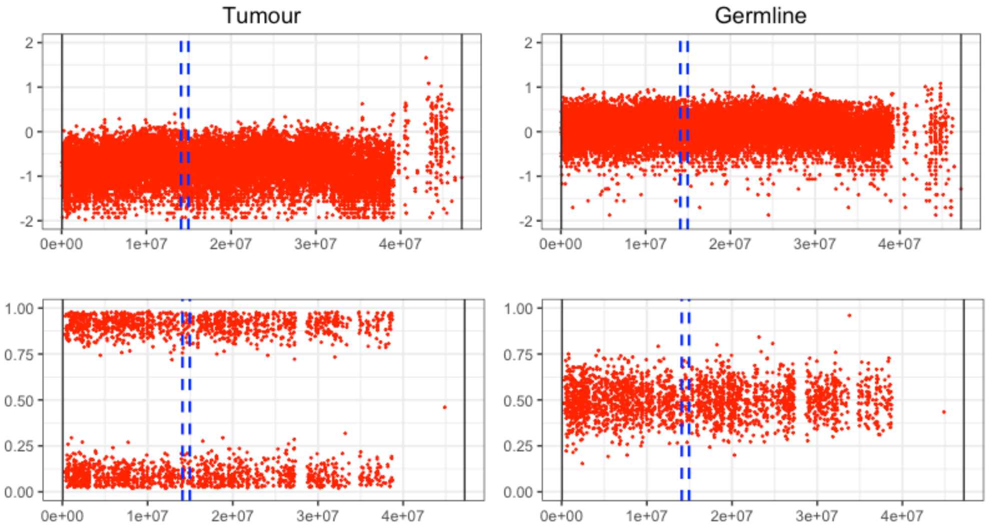

0023#T1
ASCAT region: Chr9|8672220-14960575
Minimal region of alteration: Chr9|14100316-14960575

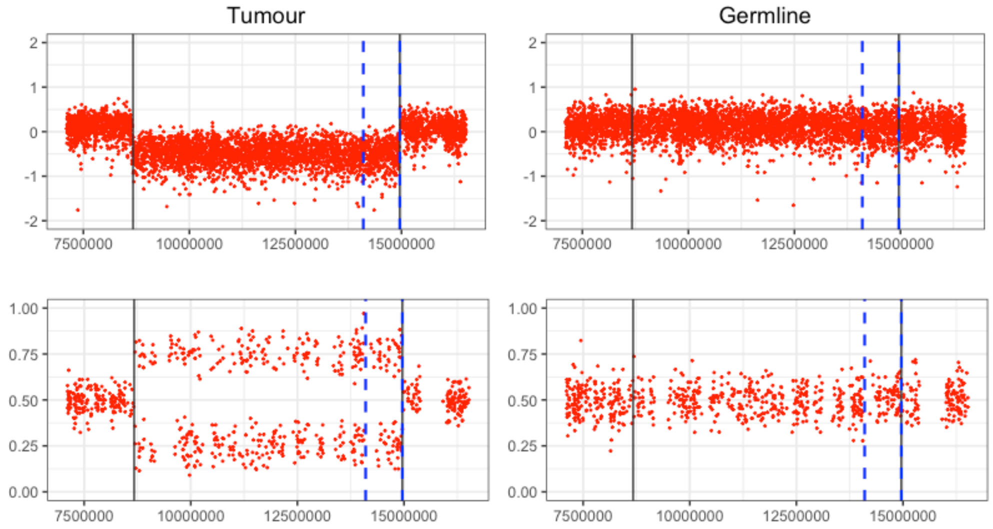

0120#T1
ASCAT region: Chr9|47463-22135210
Minimal region of alteration: Chr9|14100316-14960575

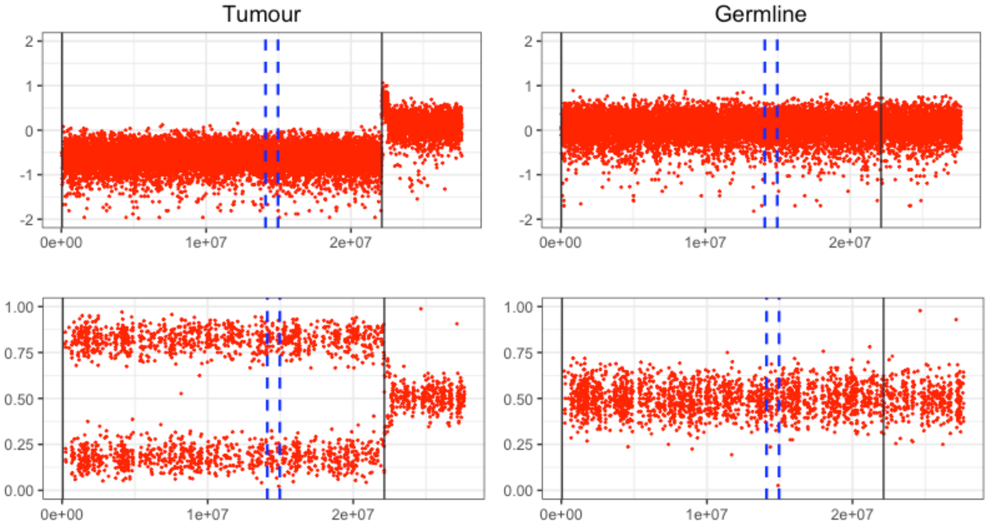

0035#T1
ASCAT region: Chr9|47463-21379549
Minimal region of alteration: Chr9|14100316-14960575

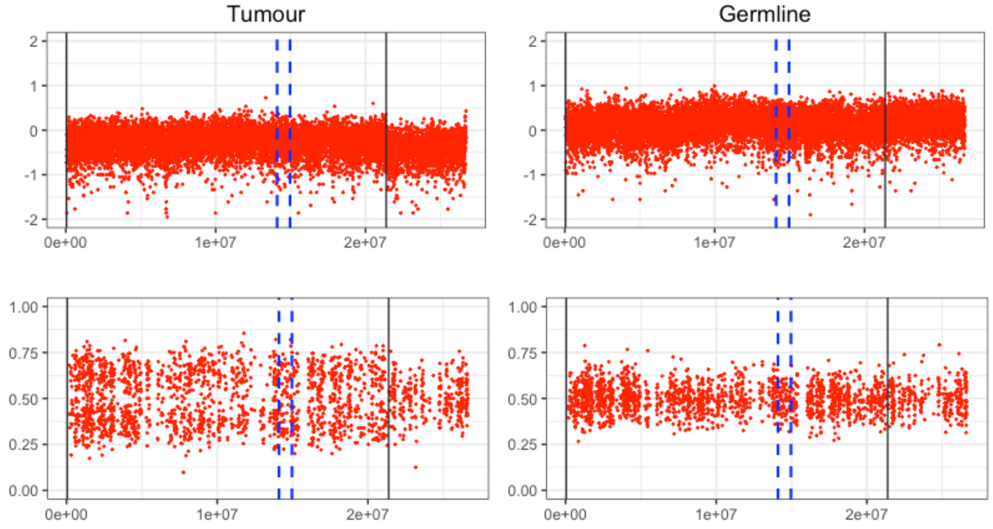

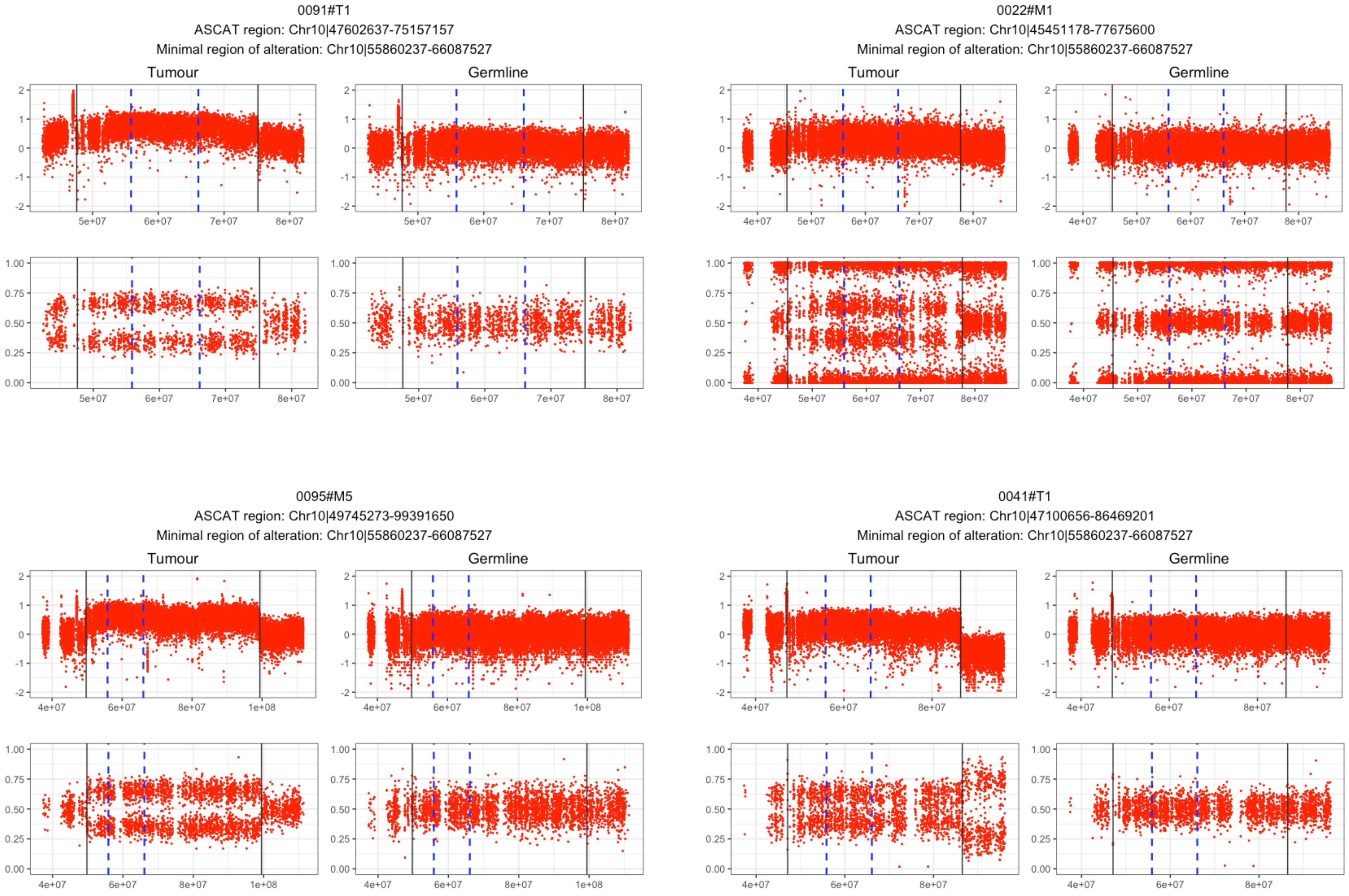
0091#T1
ASCAT region: Chr10|47602637-75157157
Minimal region of alteration: Chr10|55860237-66087527
Tumour
Germline
0022#M1
ASCAT region: Chr10|45451178-77675600
Minimal region of alteration: Chr10|55860237-66087527
Tumour
Germline
0095#M5
ASCAT region: Chr10|49745273-99391650
Minimal region of alteration: Chr10|55860237-66087527
Tumour
Germline
0041#T1
ASCAT region: Chr10|47100656-86469201
Minimal region of alteration: Chr10|55860237-66087527
Tumour
Germline

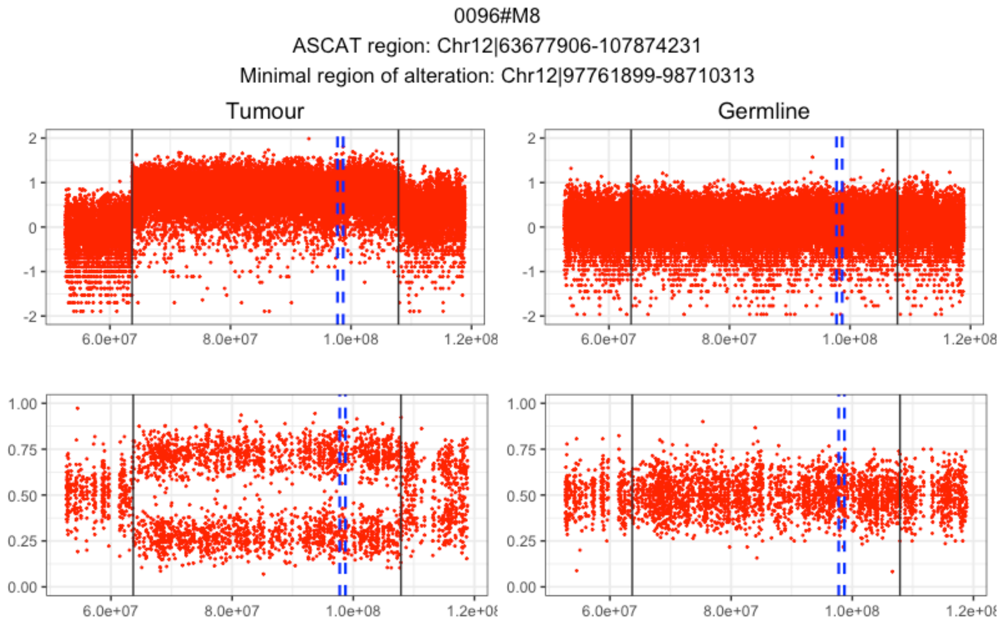
0096#M8
ASCAT region: Chr12|63677906-107874231
Minimal region of alteration: Chr12|97761899-98710313
Tumour
Germline

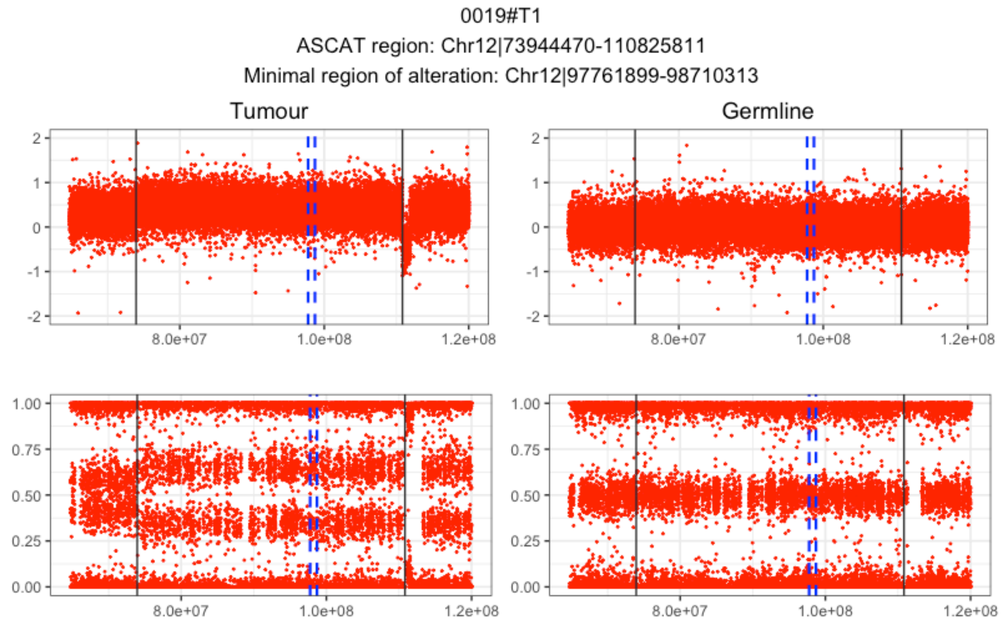
0019#T1
ASCAT region: Chr12|73944470-110825811
Minimal region of alteration: Chr12|97761899-98710313
Tumour
Germline

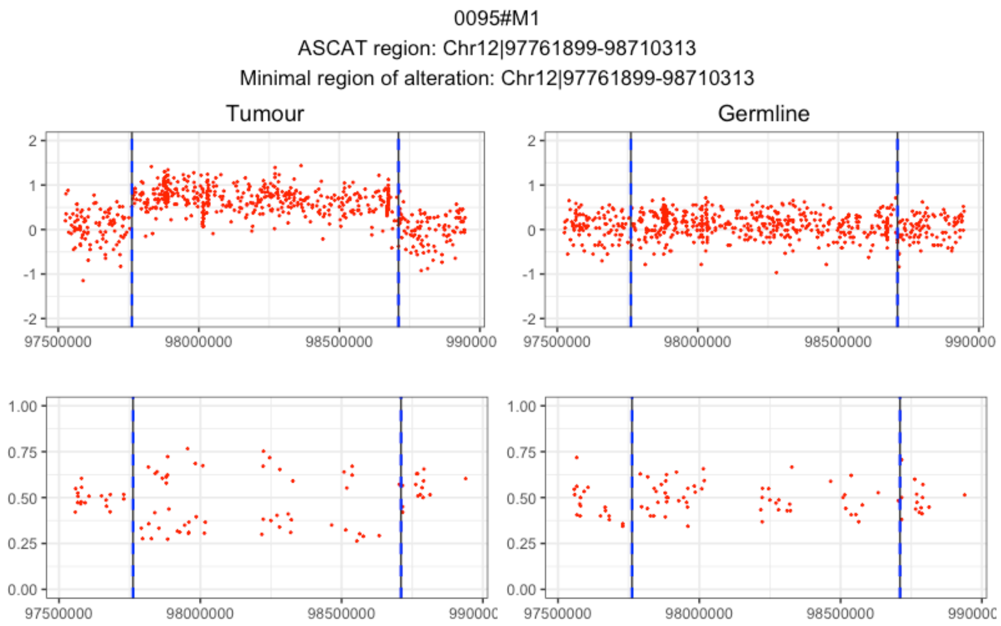
0095#M1
ASCAT region: Chr12|97761899-98710313
Minimal region of alteration: Chr12|97761899-98710313
Tumour
Germline

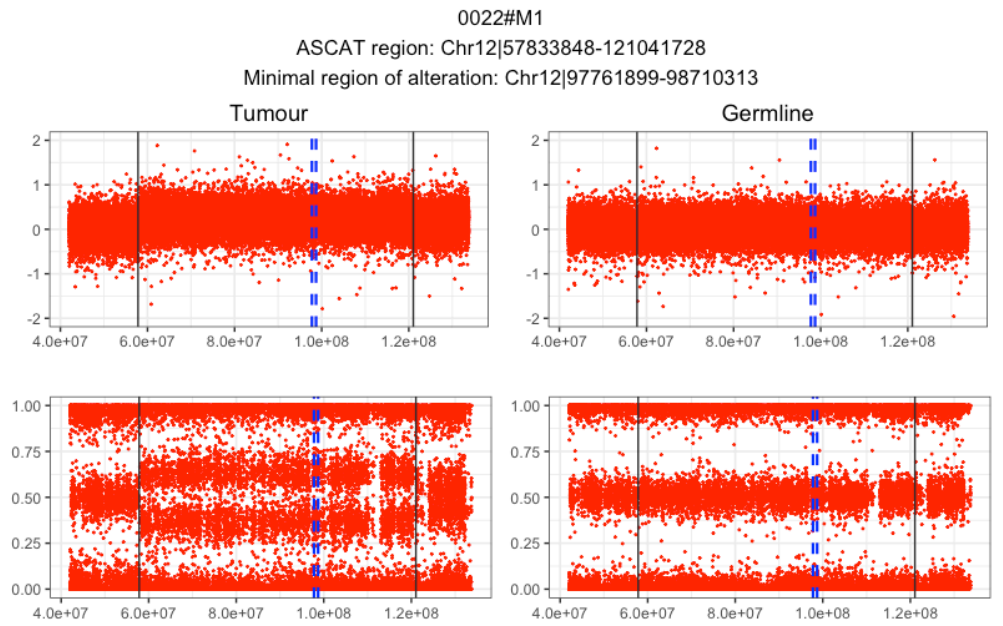
0022#M1
ASCAT region: Chr12|57833848-121041728
Minimal region of alteration: Chr12|97761899-98710313
Tumour
Germline

0061#T1
ASCAT region: Chr13|101680631-115108397
Minimal region of alteration: Chr13|107649943-110004041

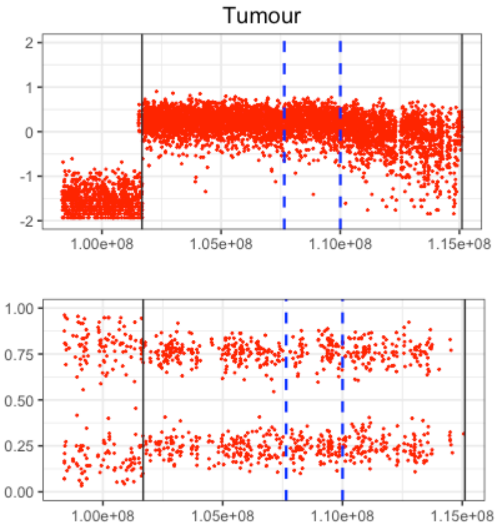

0096#M1
ASCAT region: Chr13|95598308-115108397
Minimal region of alteration: Chr13|107649943-110004041

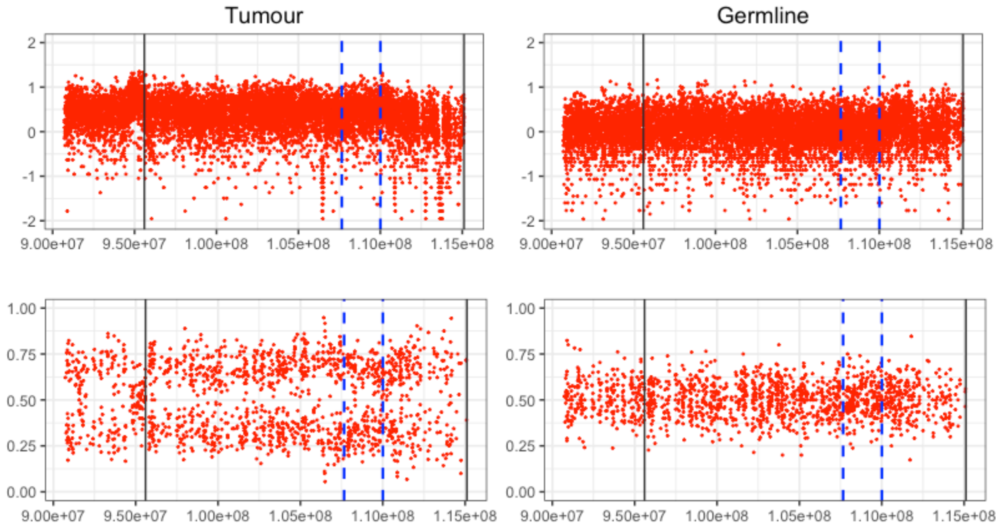

0062#T1
ASCAT region: Chr13|107649943-110004041
Minimal region of alteration: Chr13|107649943-110004041

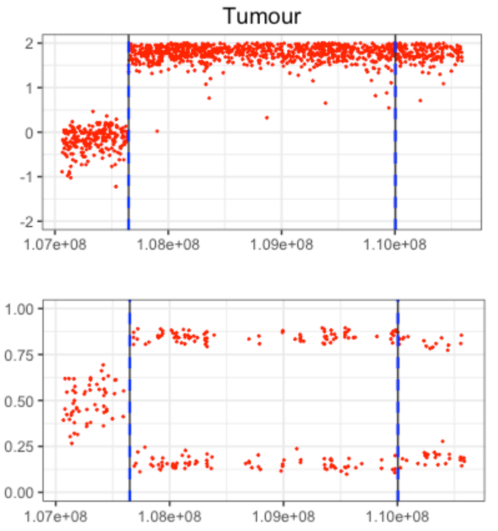

0041#T1
ASCAT region: Chr13|48218889-115108397
Minimal region of alteration: Chr13|107649943-110004041

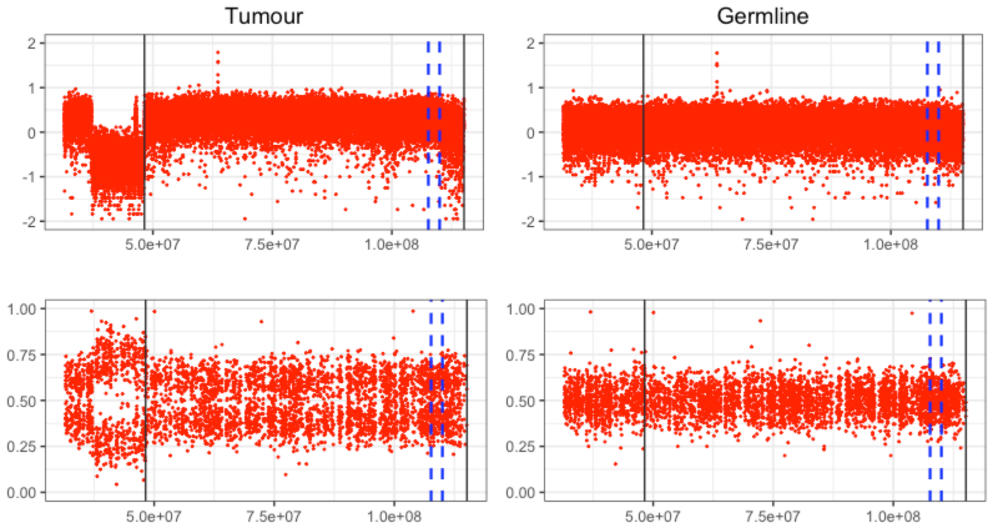

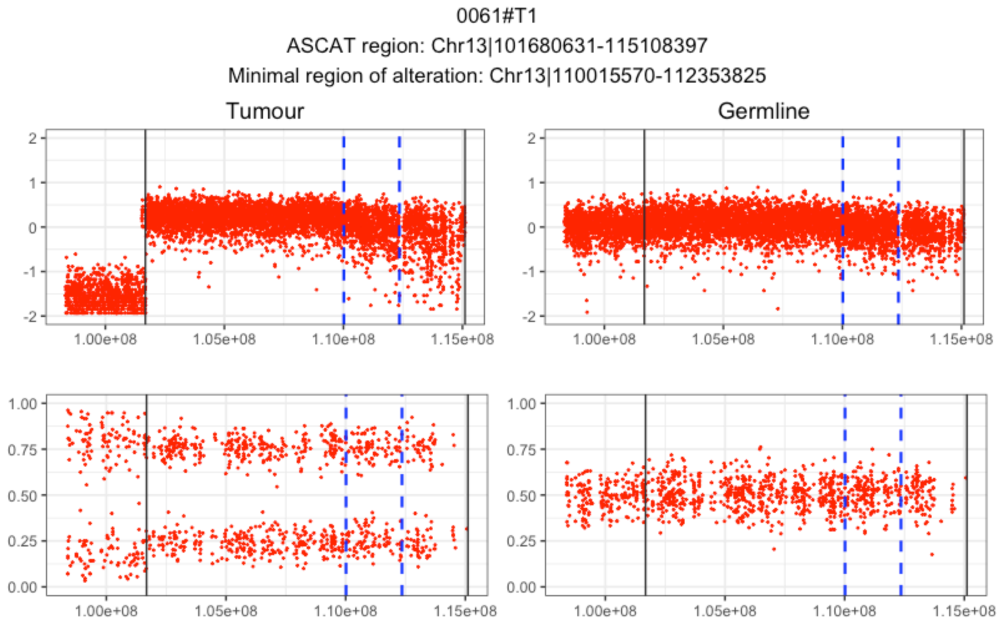
0061#T1
ASCAT region: Chr13|101680631-115108397
Minimal region of alteration: Chr13|110015570-112353825
Tumour
Germline

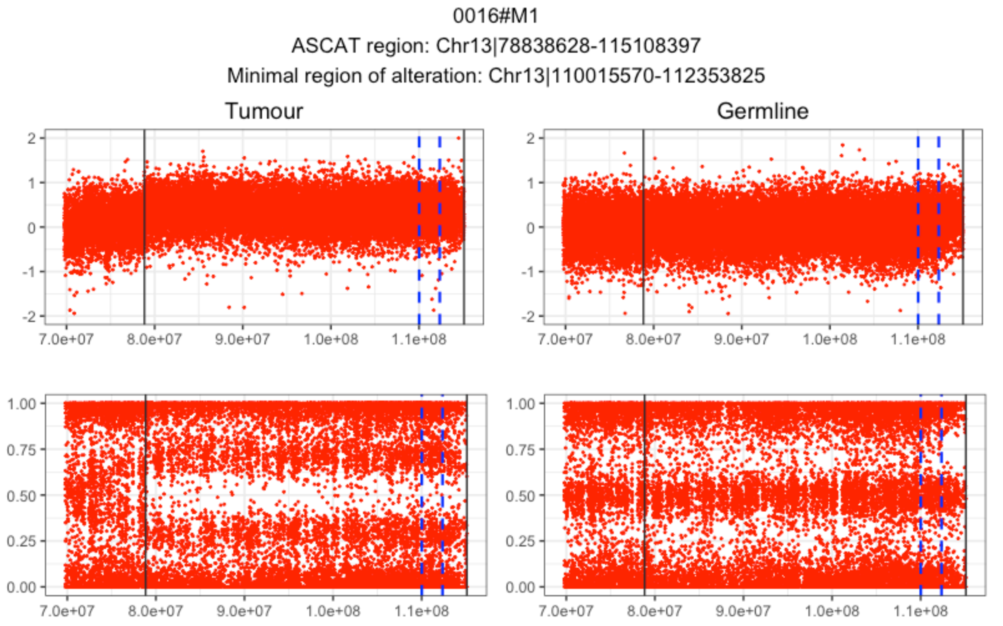
0016#M1
ASCAT region: Chr13|78838628-115108397
Minimal region of alteration: Chr13|110015570-112353825
Tumour
Germline

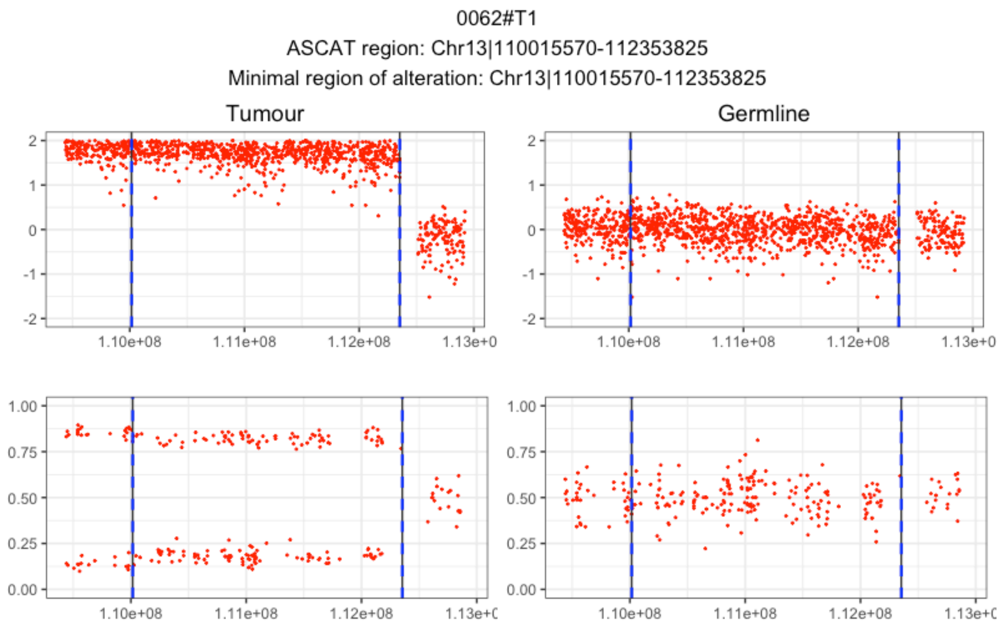
0062#T1
ASCAT region: Chr13|110015570-112353825
Minimal region of alteration: Chr13|110015570-112353825
Tumour
Germline

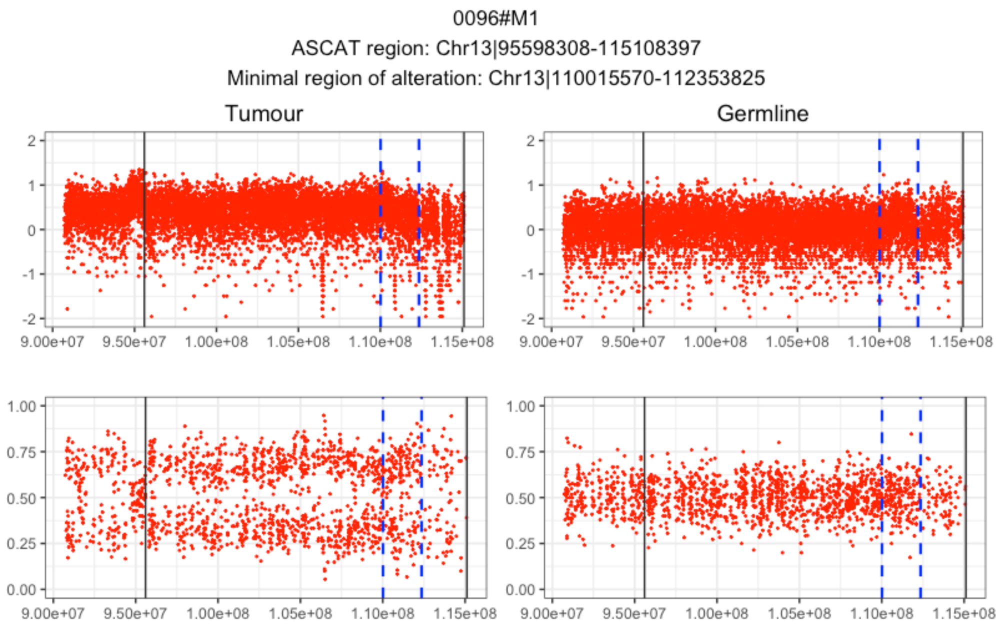
0096#M1
ASCAT region: Chr13|95598308-115108397
Minimal region of alteration: Chr13|110015570-112353825
Tumour
Germline

0069#T1
ASCAT region: Chr14|68138824-95667778
Minimal region of alteration: Chr14|68288580-68934690

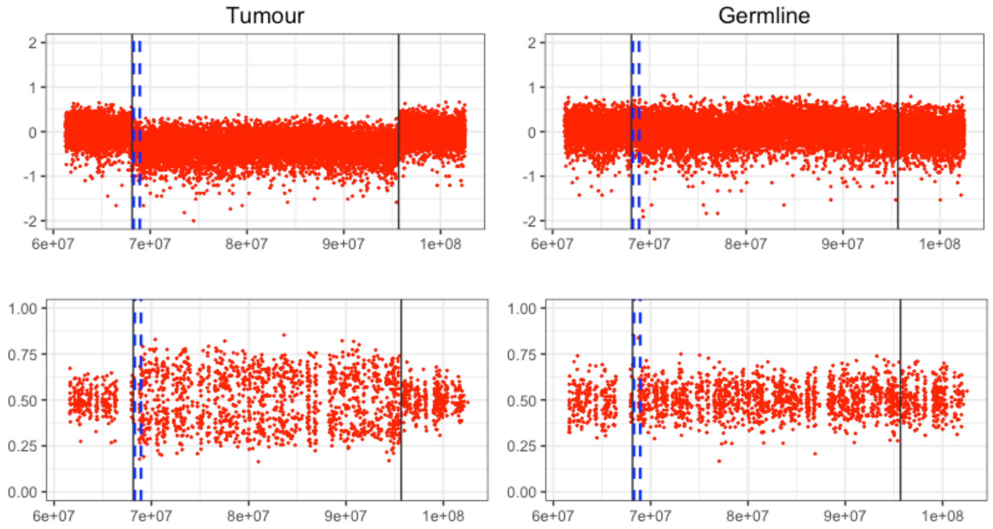

0006#T1
ASCAT region: Chr14|64162344-106197988
Minimal region of alteration: Chr14|68288580-68934690

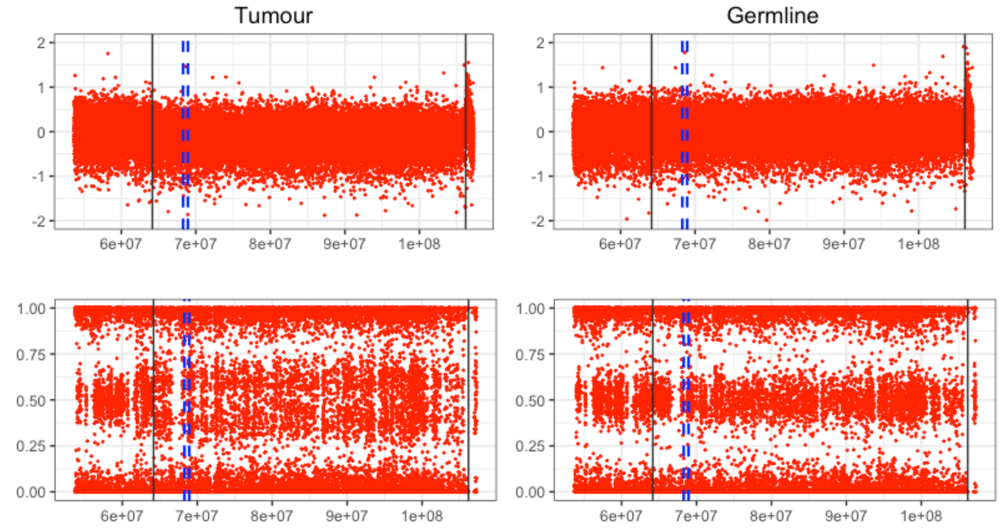

0113#T1
ASCAT region: Chr14|68288580-69508603
Minimal region of alteration: Chr14|68288580-68934690

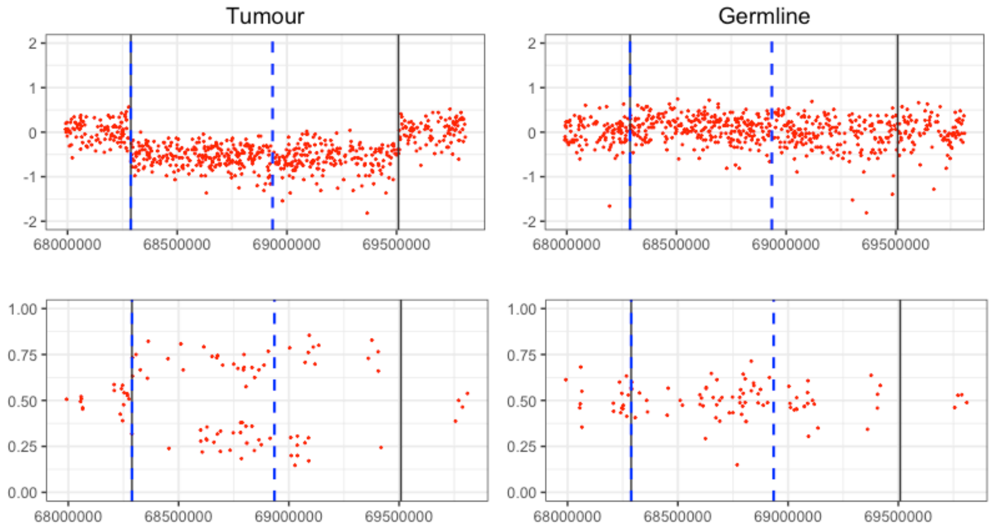

0023#T1
ASCAT region: Chr14|67633758-107285437
Minimal region of alteration: Chr14|68288580-68934690

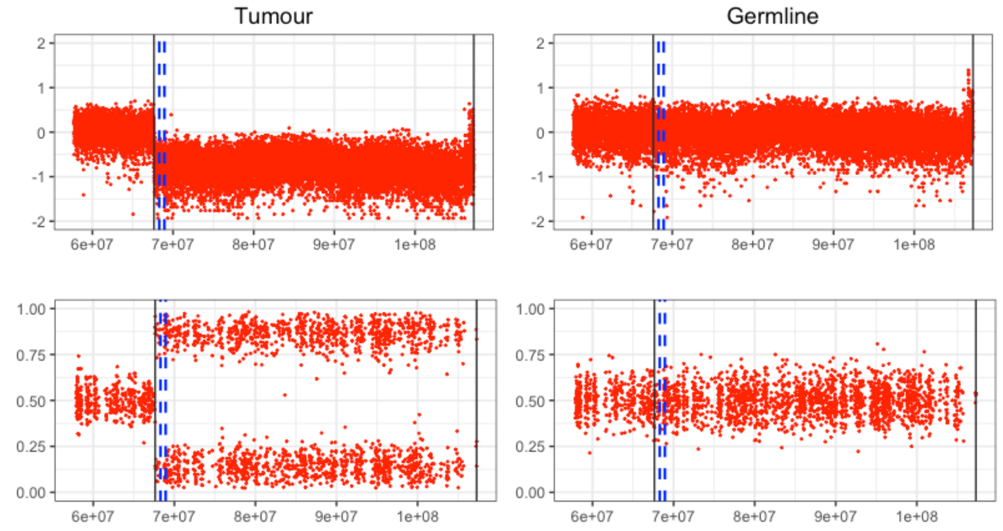

0070#T1
ASCAT region: Chr14|90757133-97027759
Minimal region of alteration: Chr14|95403326-95667778

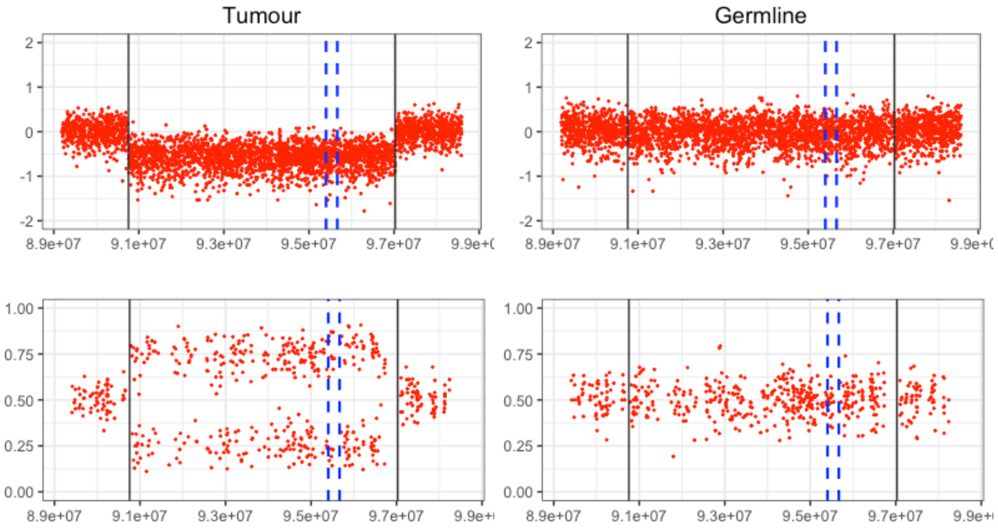

0023#T1
ASCAT region: Chr14|67633758-107285437
Minimal region of alteration: Chr14|95403326-95667778

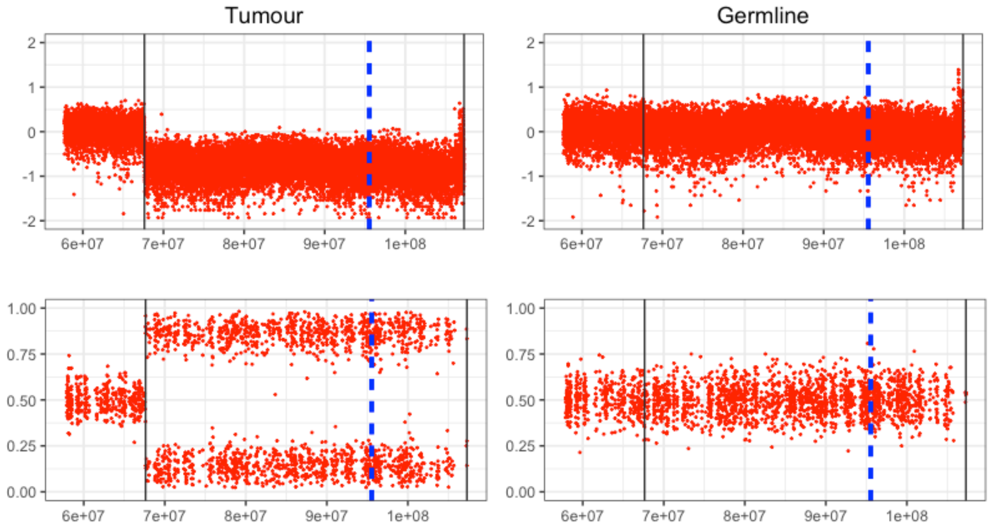

0098#M1
ASCAT region: Chr14|64254037-107285437
Minimal region of alteration: Chr14|95403326-95667778

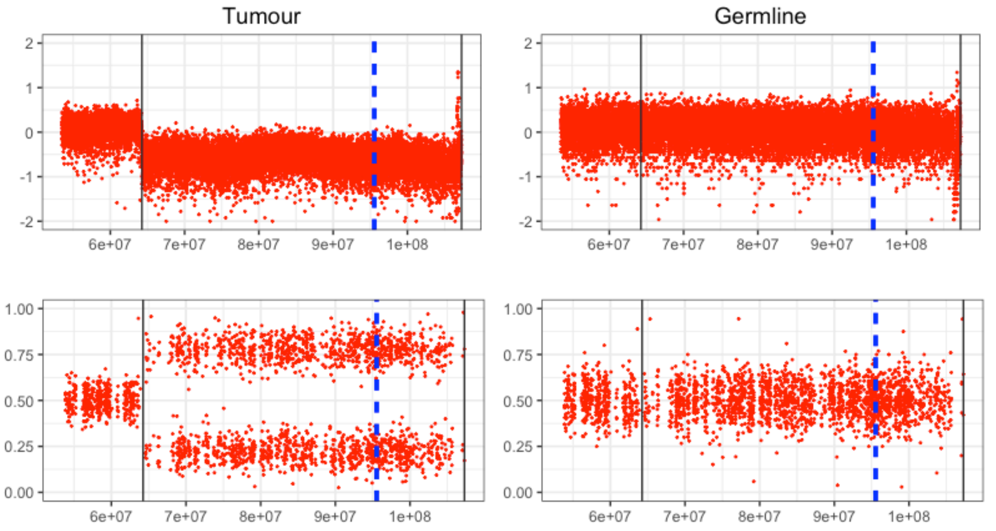

0069#T1
ASCAT region: Chr14|68138824-95667778
Minimal region of alteration: Chr14|95403326-95667778

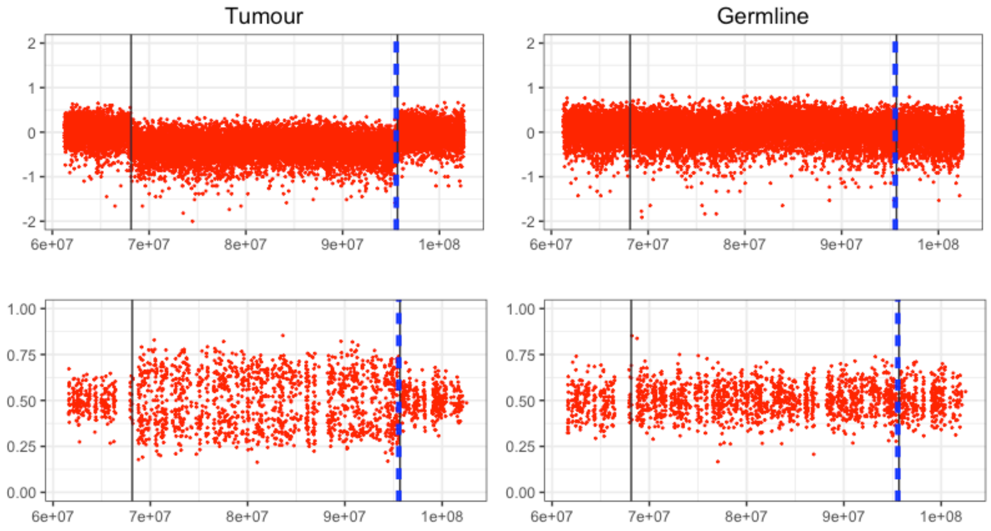

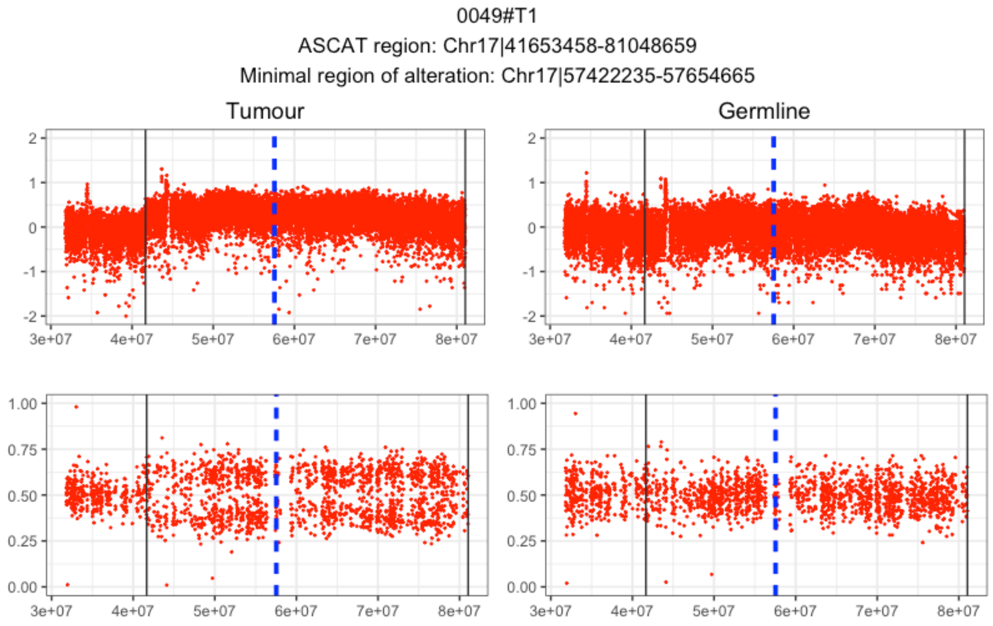
0049#T1
ASCAT region: Chr17|41653458-81048659
Minimal region of alteration: Chr17|57422235-57654665
Tumour
Germline
3e+07 4e+07 5e+07 6e+07 7e+07 8e+07
2 1 0 -1 -2
1.00 0.75 0.50 0.25 0.00

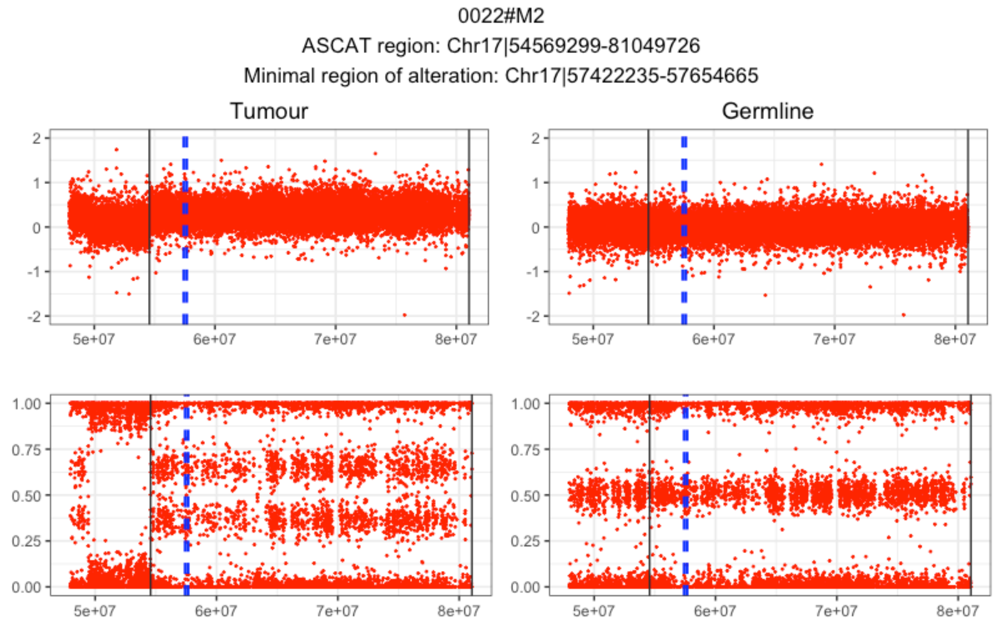
0022#M2
ASCAT region: Chr17|54569299-81049726
Minimal region of alteration: Chr17|57422235-57654665
Tumour
Germline
5e+07 6e+07 7e+07 8e+07
2 1 0 -1 -2
1.00 0.75 0.50 0.25 0.00

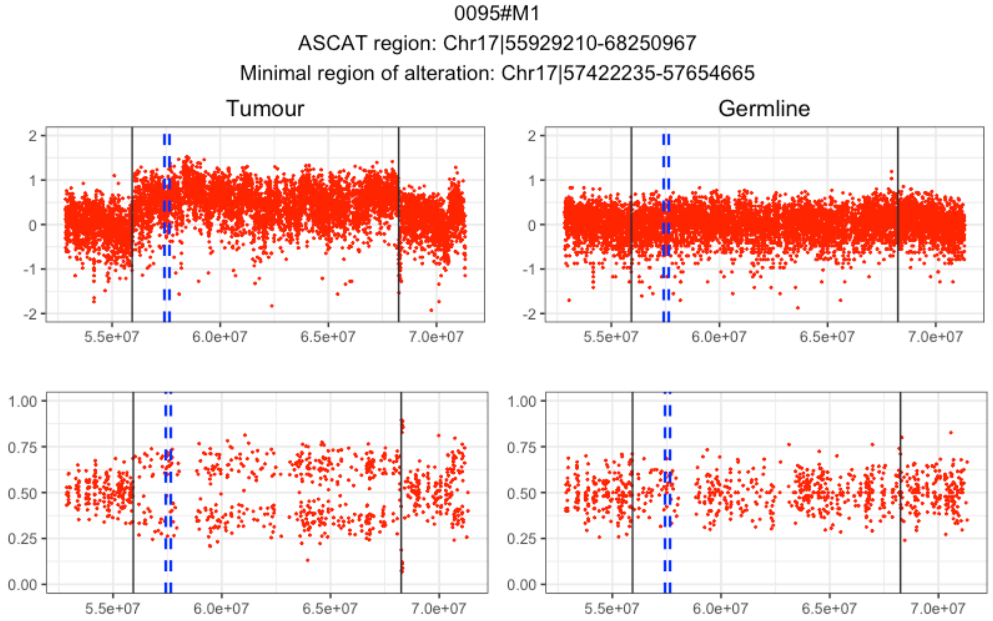
0095#M1
ASCAT region: Chr17|55929210-68250967
Minimal region of alteration: Chr17|57422235-57654665
Tumour
Germline
5.5e+07 6.0e+07 6.5e+07 7.0e+07
2 1 0 -1 -2
1.00 0.75 0.50 0.25 0.00

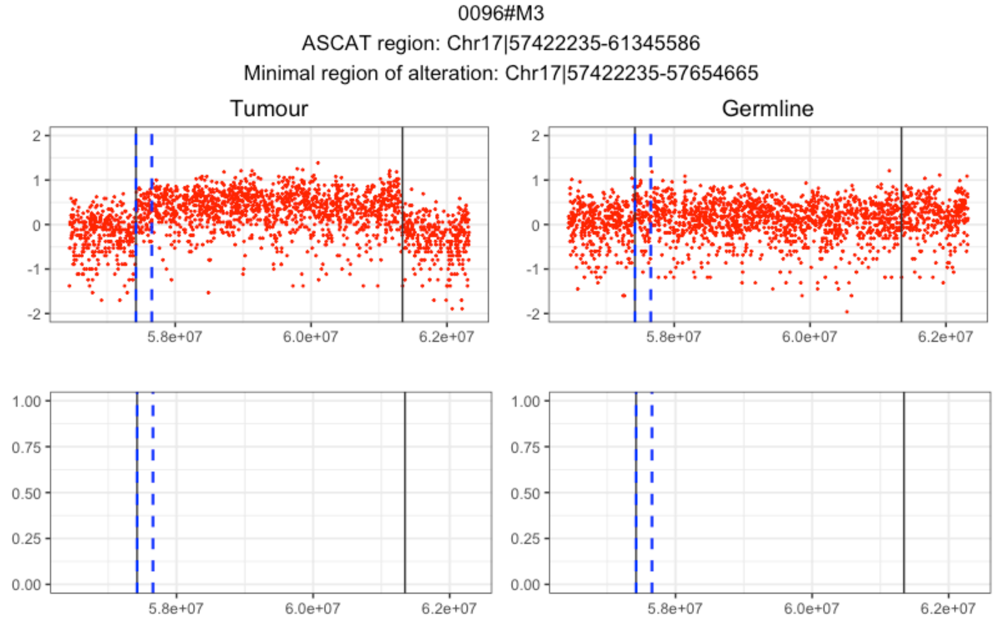
0096#M3
ASCAT region: Chr17|57422235-61345586
Minimal region of alteration: Chr17|57422235-57654665
Tumour
Germline
5.8e+07 6.0e+07 6.2e+07
2 1 0 -1 -2
1.00 0.75 0.50 0.25 0.00

0070#T1
ASCAT region: Chr18|2565103-5836359
Minimal region of alteration: Chr18|2565103-3788627

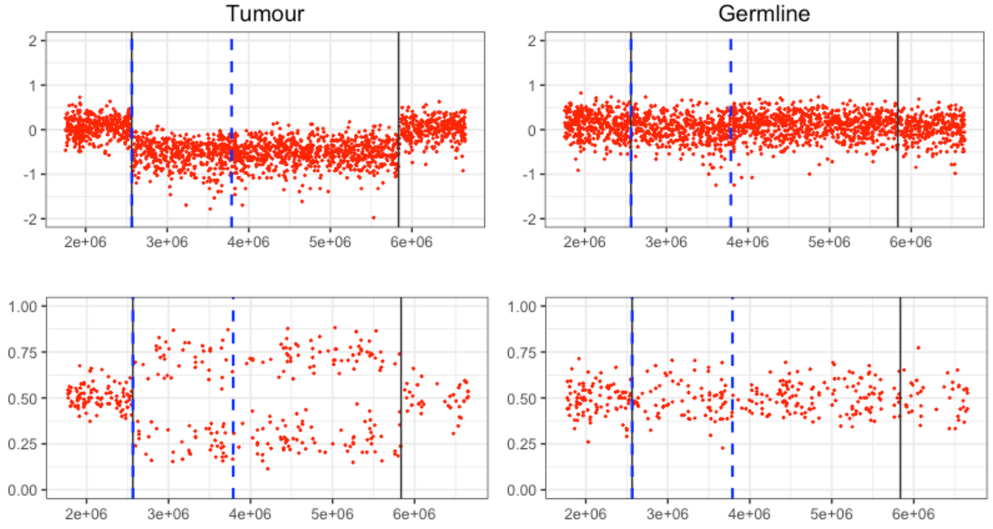

0028#T1
ASCAT region: Chr18|48133-4943870
Minimal region of alteration: Chr18|2565103-3788627

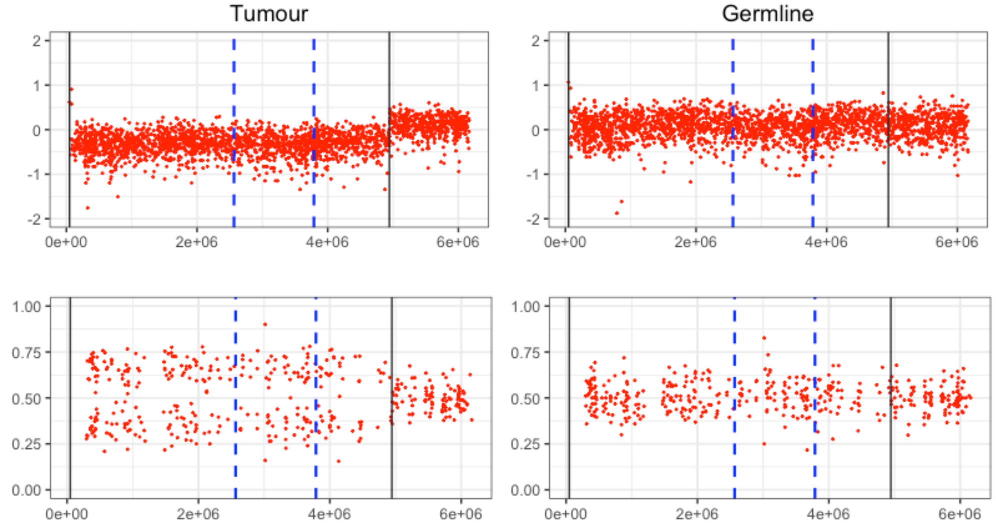

0120#T1
ASCAT region: Chr18|48133-78014136
Minimal region of alteration: Chr18|2565103-3788627

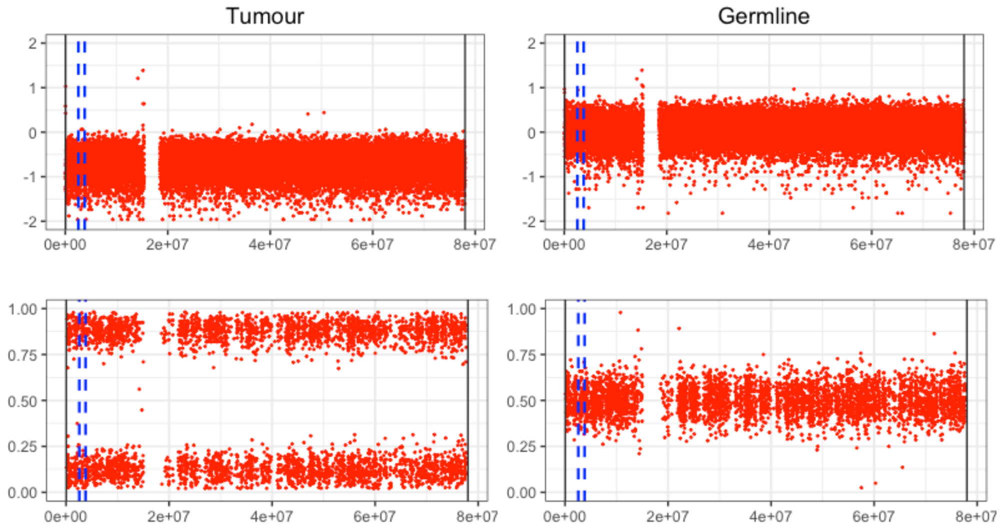

0065#T1
ASCAT region: Chr18|48133-78014136
Minimal region of alteration: Chr18|2565103-3788627

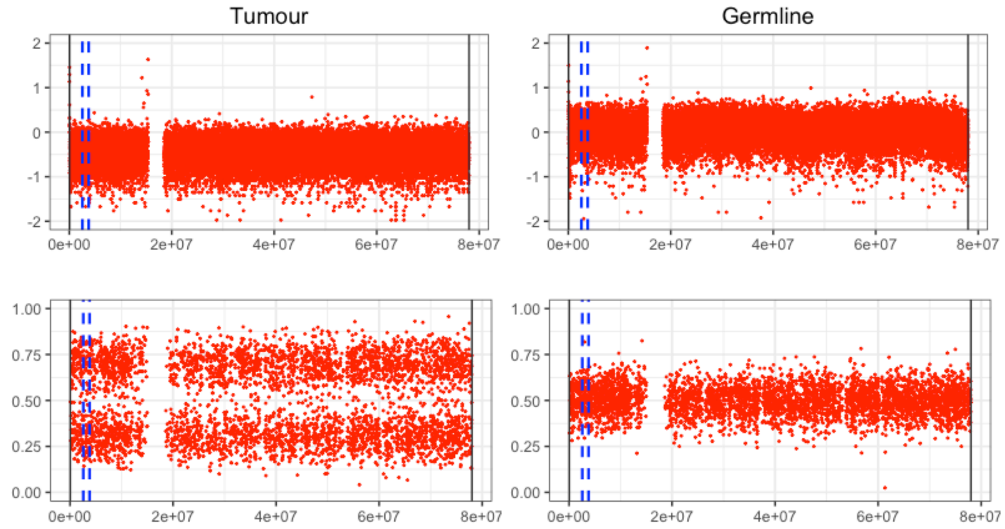

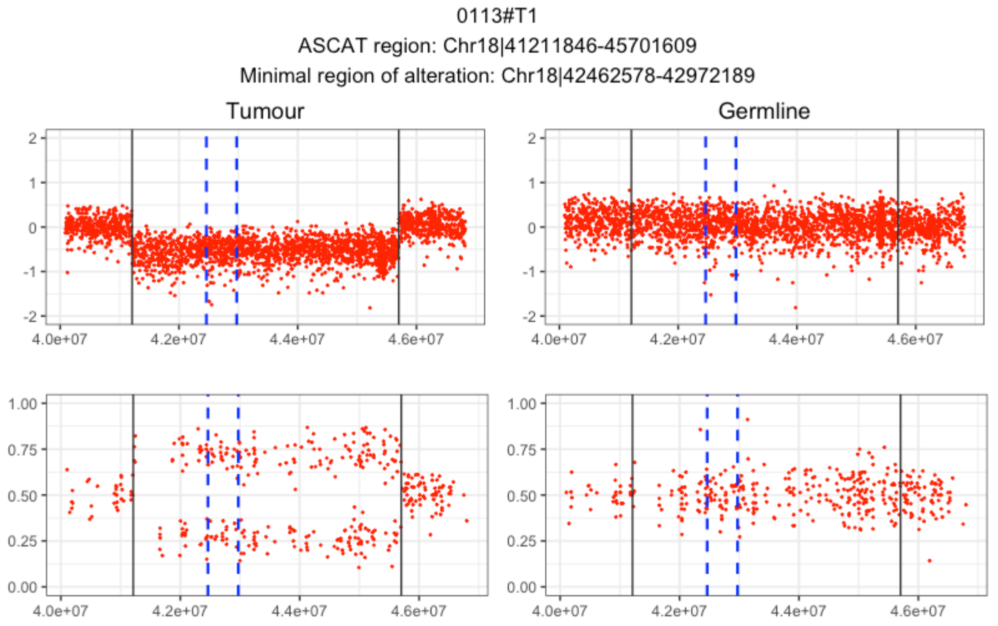
0113#T1
ASCAT region: Chr18|41211846-45701609
Minimal region of alteration: Chr18|42462578-42972189
Tumour
Germline

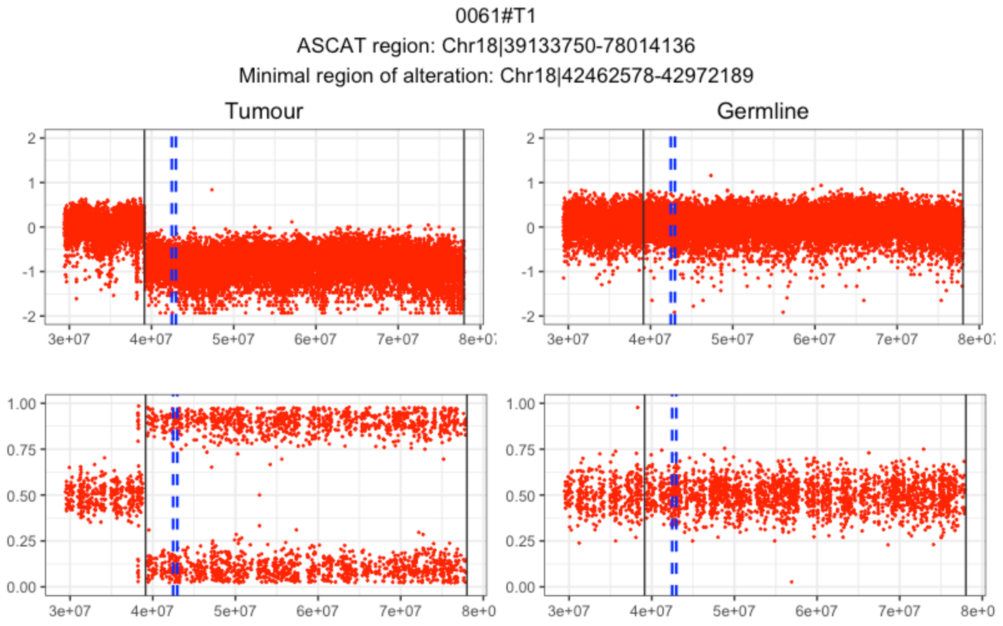
0061#T1
ASCAT region: Chr18|39133750-78014136
Minimal region of alteration: Chr18|42462578-42972189
Tumour
Germline

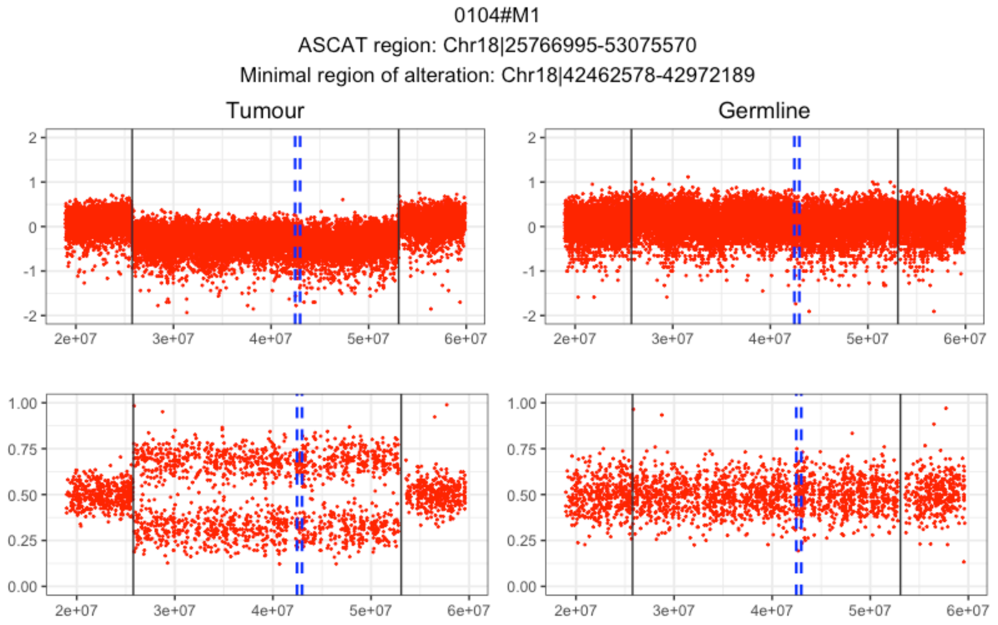
0104#M1
ASCAT region: Chr18|25766995-53075570
Minimal region of alteration: Chr18|42462578-42972189
Tumour
Germline

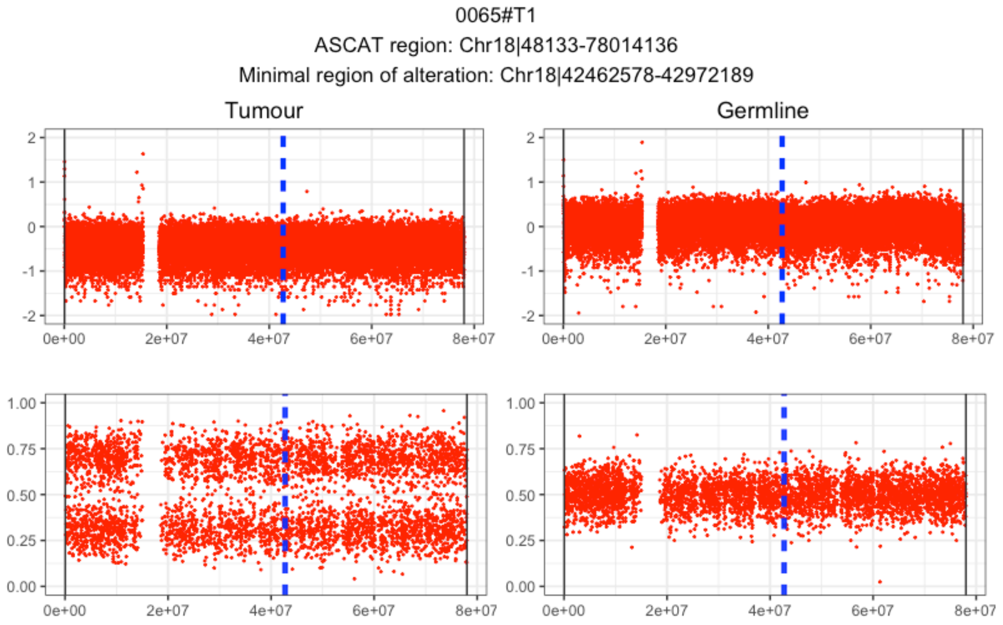
0065#T1
ASCAT region: Chr18|48133-78014136
Minimal region of alteration: Chr18|42462578-42972189
Tumour
Germline

0097#M1
ASCAT region: Chr19|90910-24582577
Minimal region of alteration: Chr19|24253251-24385424

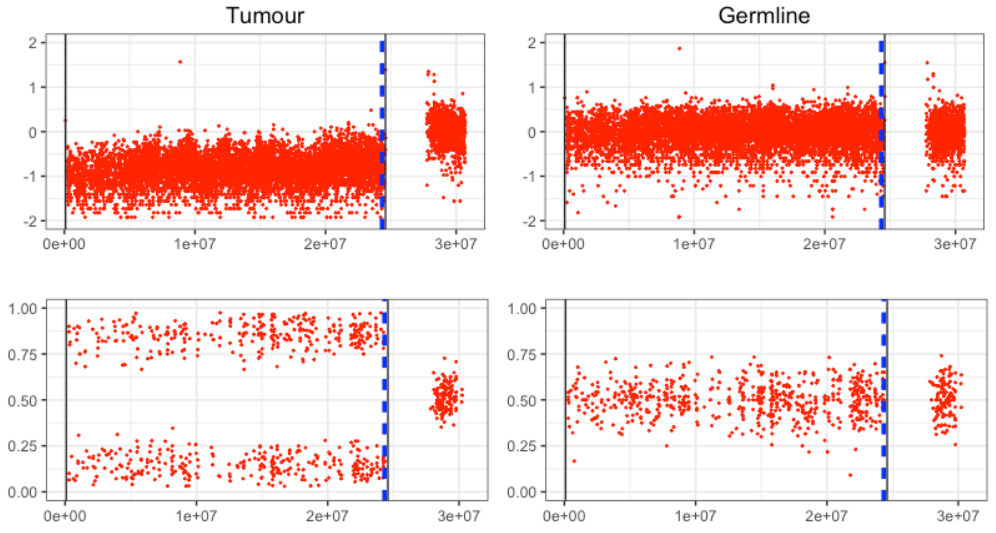

0019#M1
ASCAT region: Chr19|90910-24583312
Minimal region of alteration: Chr19|24253251-24385424

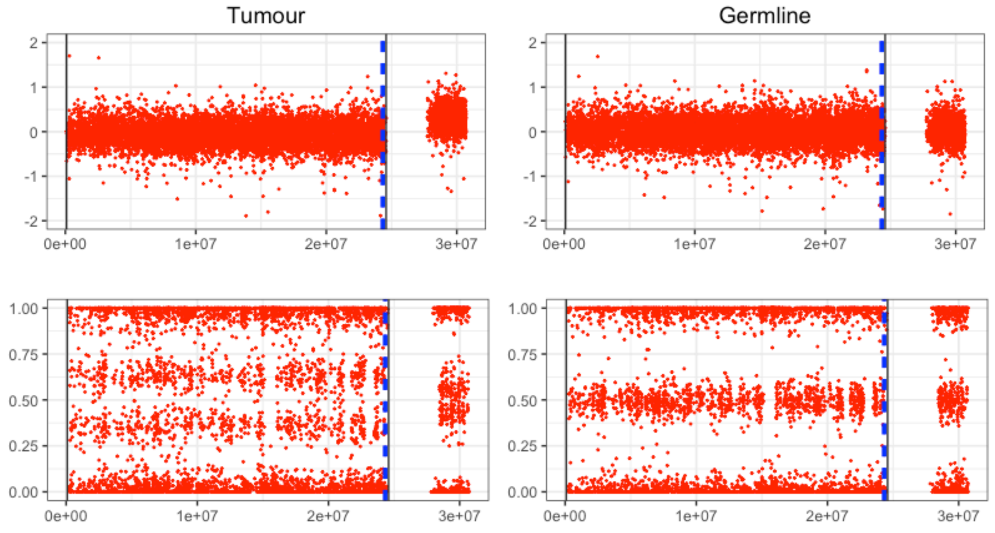

0104#M1
ASCAT region: Chr19|90910-48462630
Minimal region of alteration: Chr19|24253251-24385424

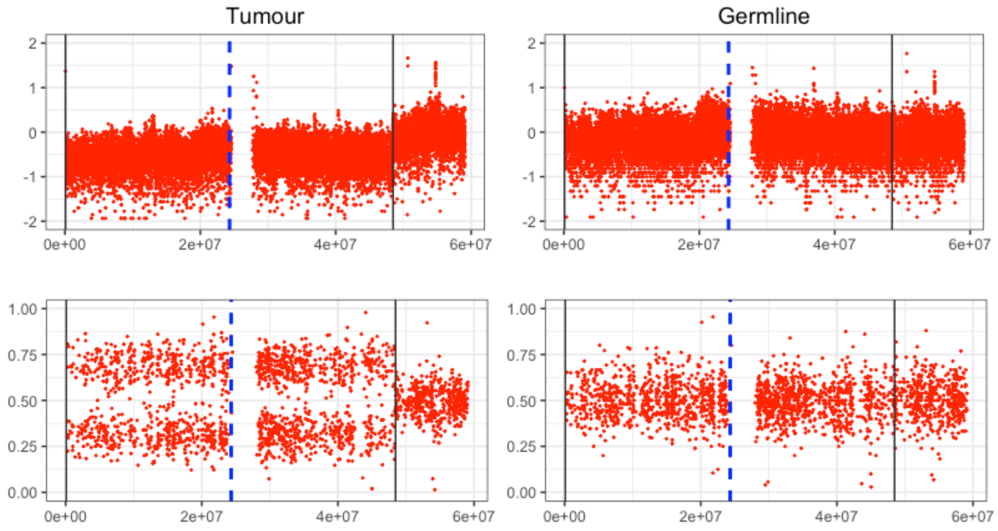

0095#M1
ASCAT region: Chr19|23765492-24385424
Minimal region of alteration: Chr19|24253251-24385424

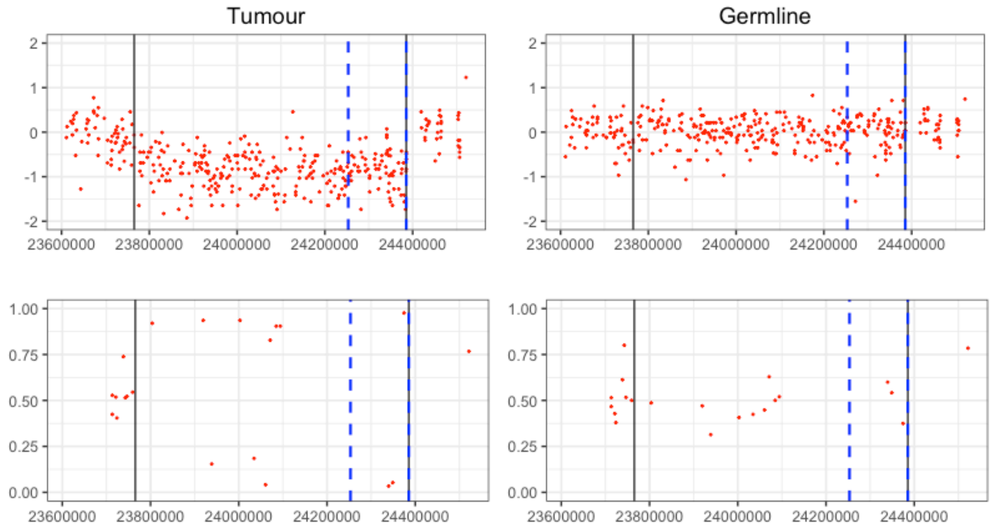

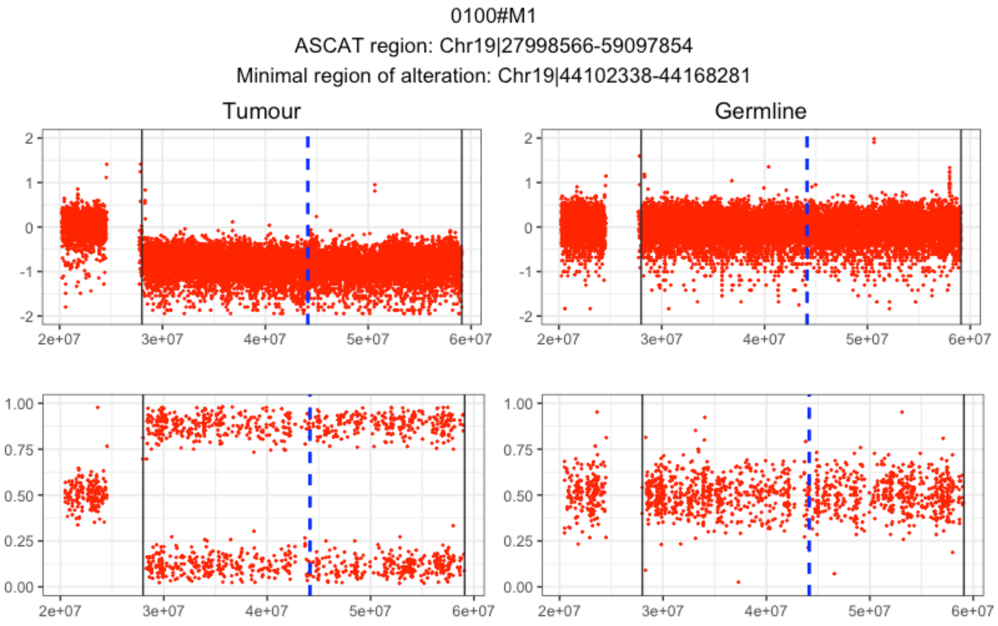
0100#M1
ASCAT region: Chr19|27998566-59097854
Minimal region of alteration: Chr19|44102338-44168281
Tumour
Germline

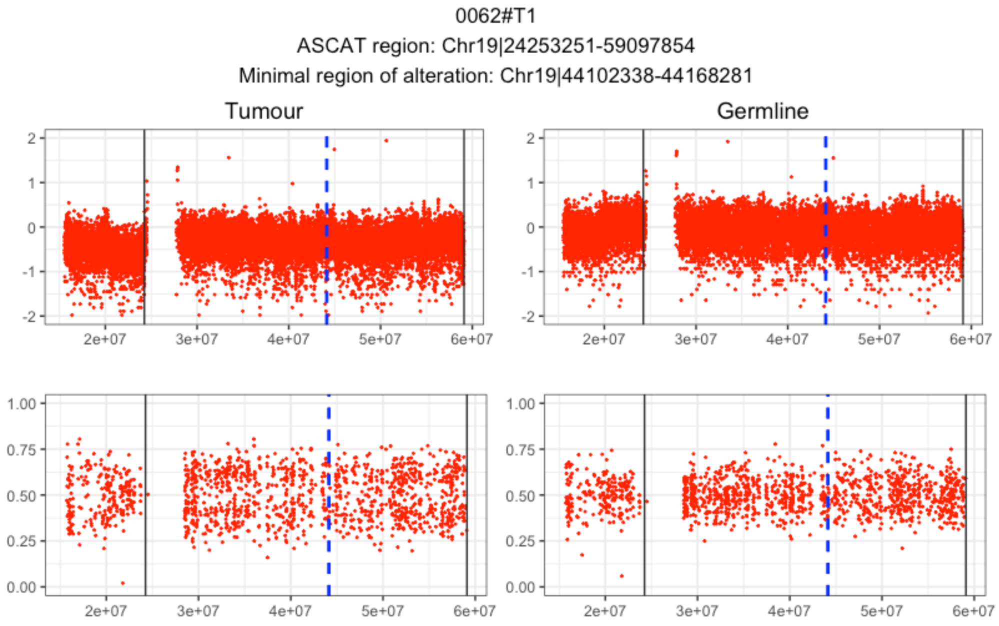
0062#T1
ASCAT region: Chr19|24253251-59097854
Minimal region of alteration: Chr19|44102338-44168281
Tumour
Germline

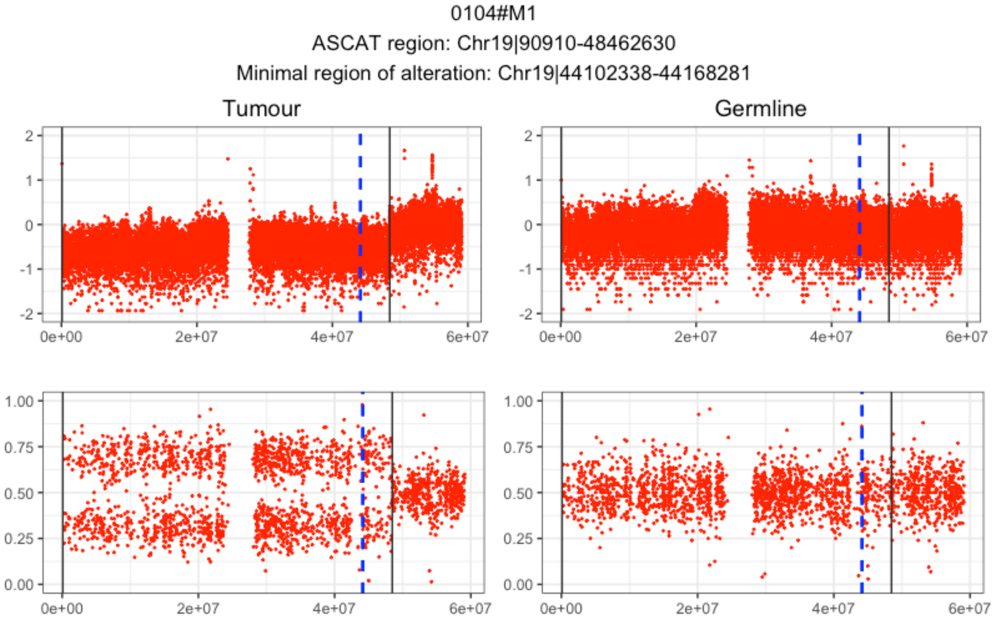
0104#M1
ASCAT region: Chr19|90910-48462630
Minimal region of alteration: Chr19|44102338-44168281
Tumour
Germline

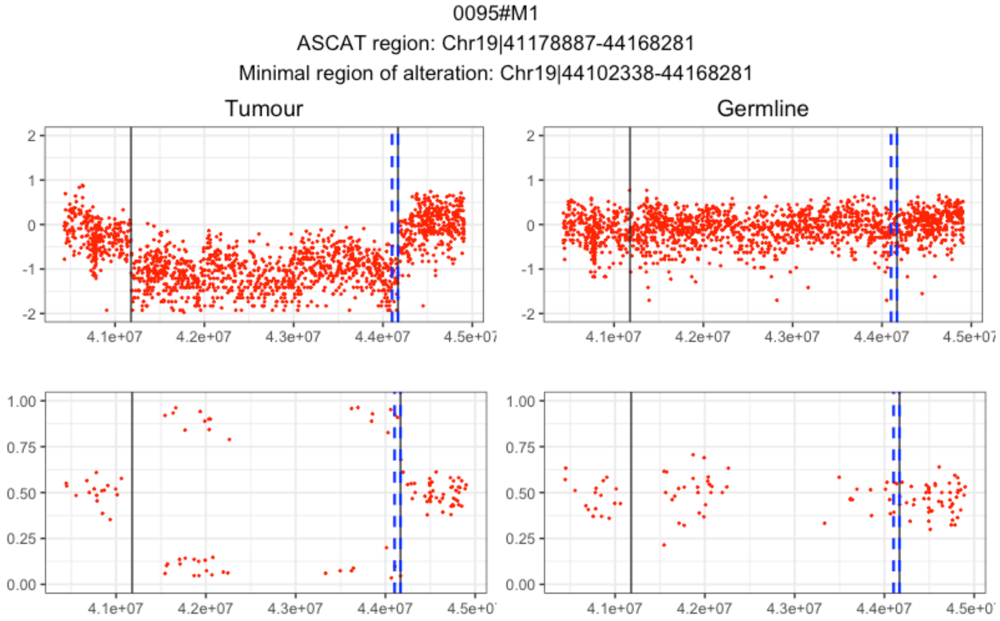
0095#M1
ASCAT region: Chr19|41178887-44168281
Minimal region of alteration: Chr19|44102338-44168281
Tumour
Germline

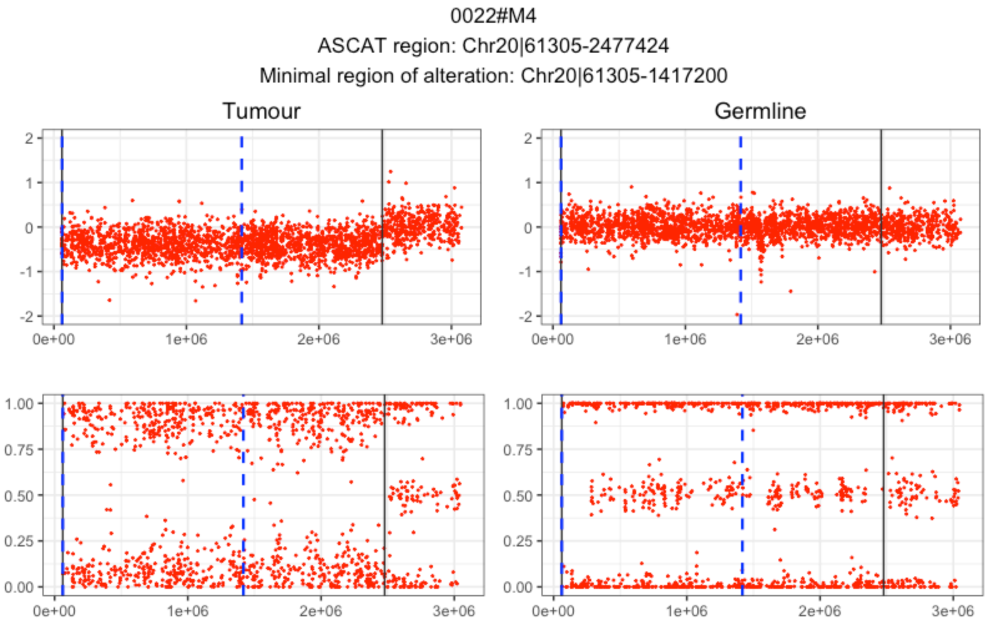
0022#M4
ASCAT region: Chr20|61305-2477424
Minimal region of alteration: Chr20|61305-1417200
Tumour
Germline

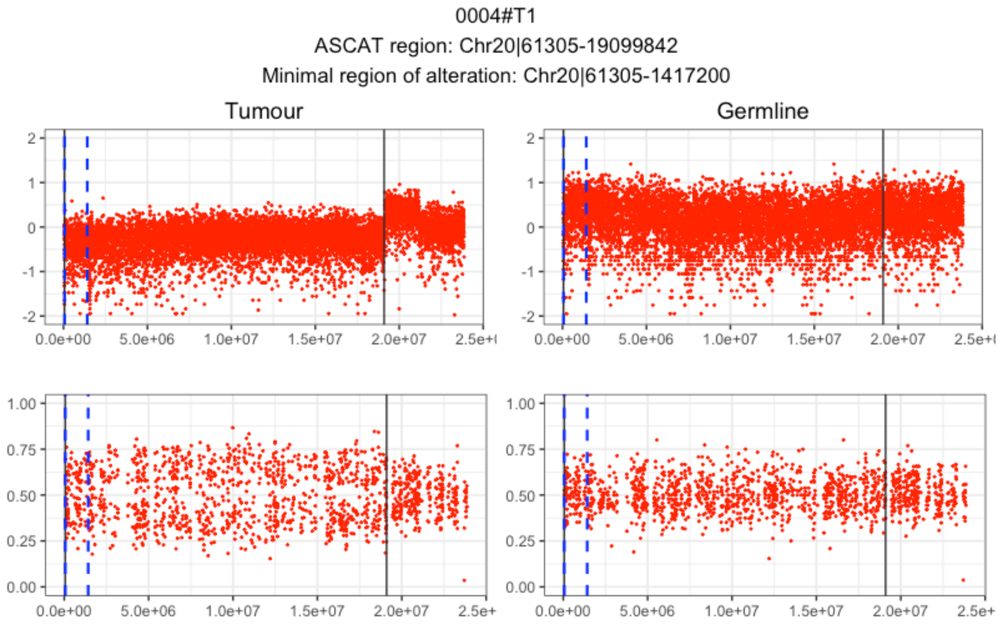
0004#T1
ASCAT region: Chr20|61305-19099842
Minimal region of alteration: Chr20|61305-1417200
Tumour
Germline

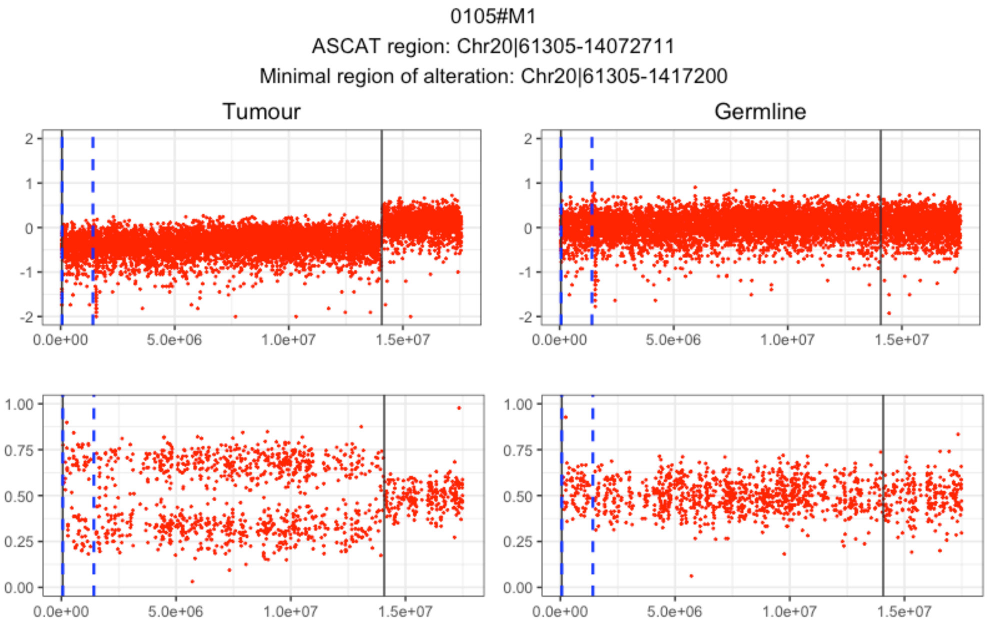
0105#M1
ASCAT region: Chr20|61305-14072711
Minimal region of alteration: Chr20|61305-1417200
Tumour
Germline

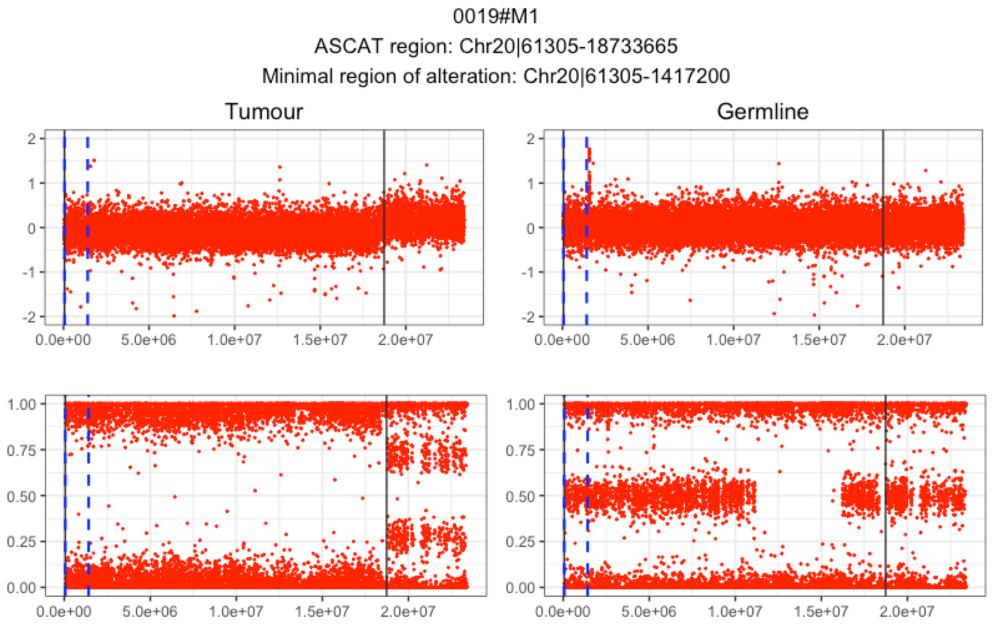
0019#M1
ASCAT region: Chr20|61305-18733665
Minimal region of alteration: Chr20|61305-1417200
Tumour
Germline

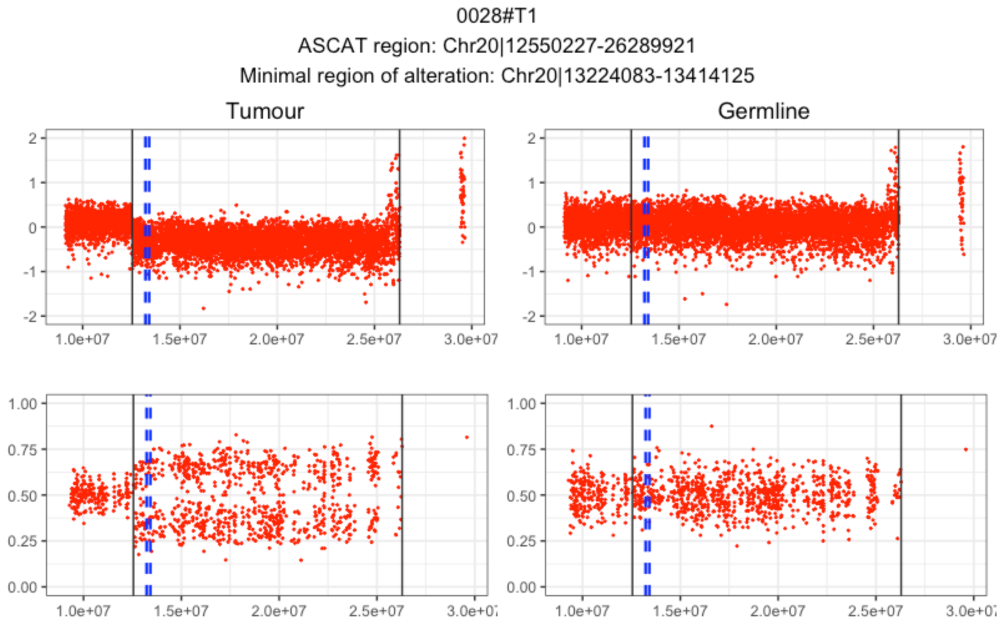
0028#T1
ASCAT region: Chr20|12550227-26289921
Minimal region of alteration: Chr20|13224083-13414125
Tumour
Germline

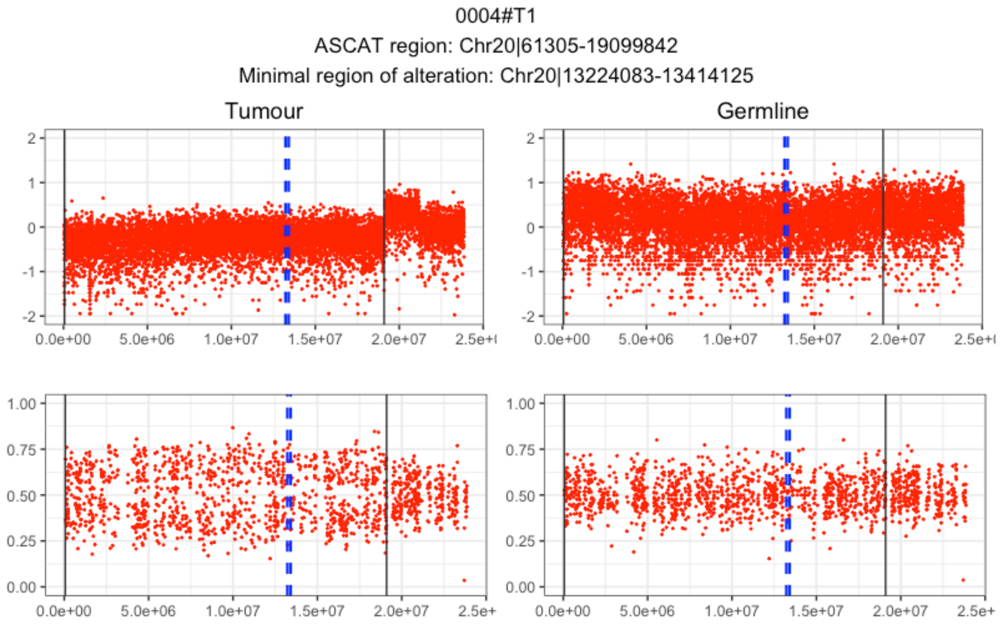
0004#T1
ASCAT region: Chr20|61305-19099842
Minimal region of alteration: Chr20|13224083-13414125
Tumour
Germline

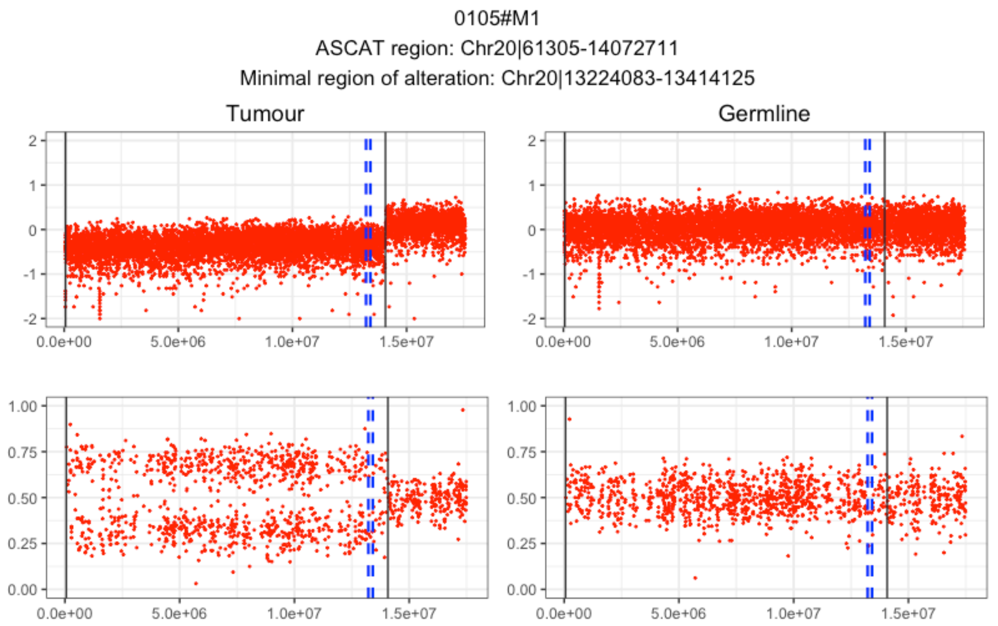
0105#M1
ASCAT region: Chr20|61305-14072711
Minimal region of alteration: Chr20|13224083-13414125
Tumour
Germline

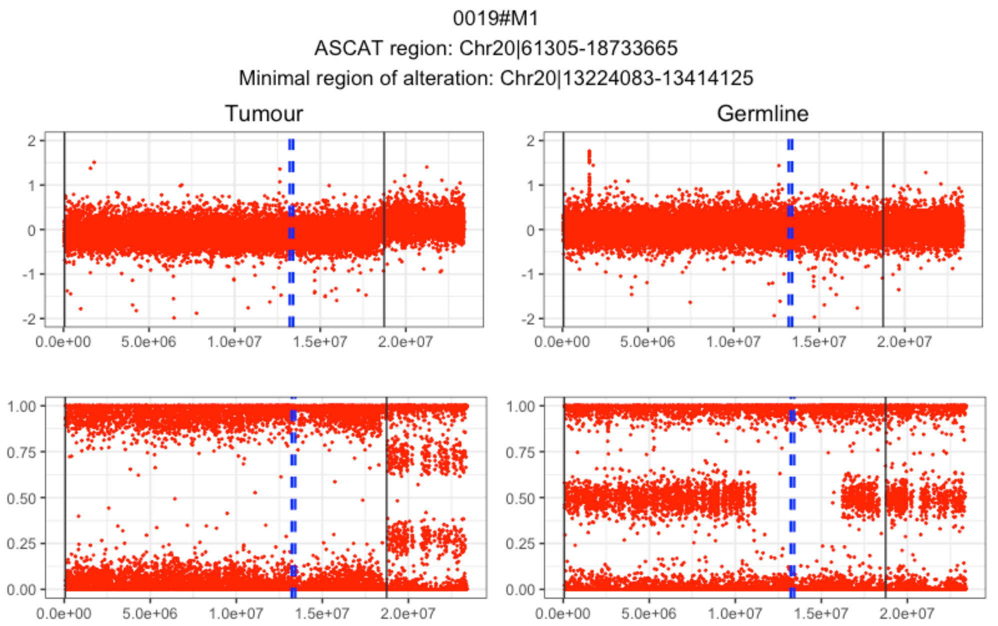
0019#M1
ASCAT region: Chr20|61305-18733665
Minimal region of alteration: Chr20|13224083-13414125
Tumour
Germline

0095#M4
ASCAT region: Chr20|50274179-62956153
Minimal region of alteration: Chr20|60377988-62956153

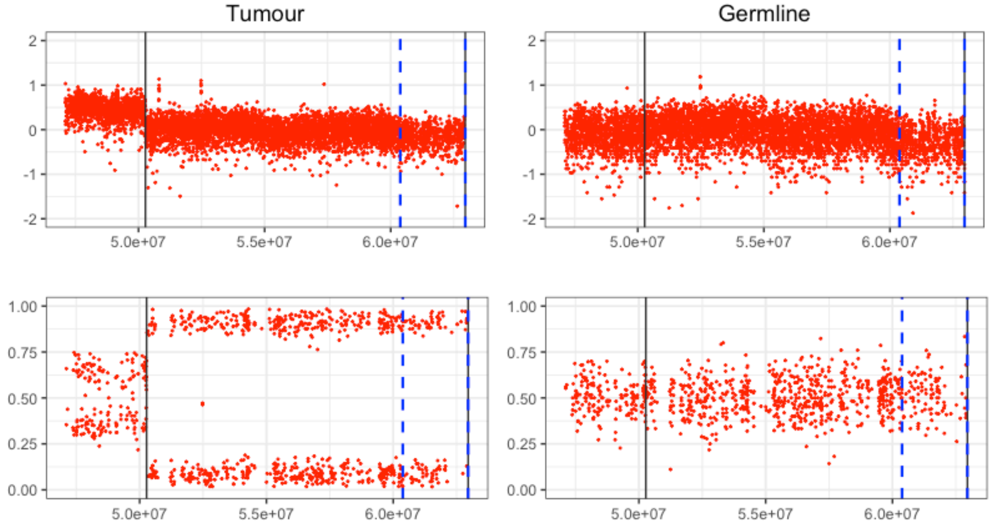

0016#M1
ASCAT region: Chr20|26305579-62956153
Minimal region of alteration: Chr20|60377988-62956153

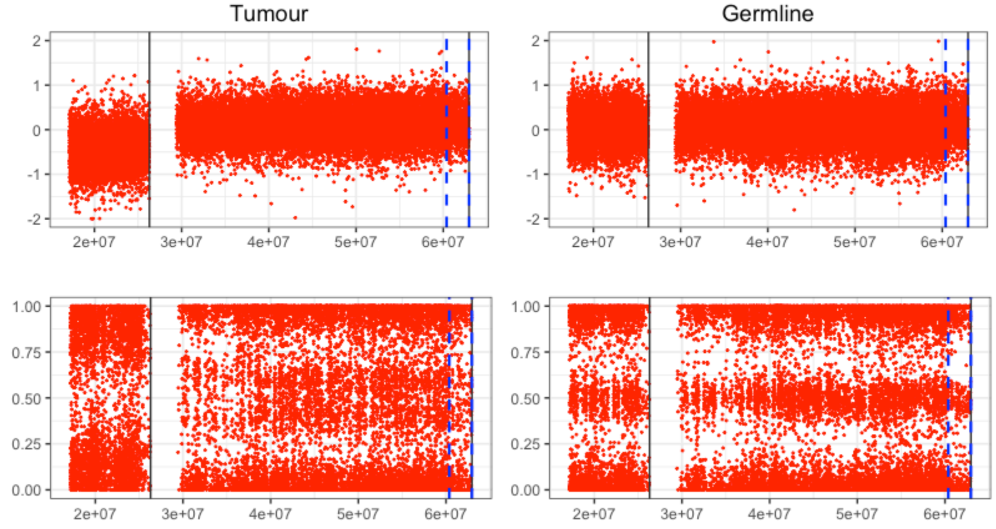

0097#M1
ASCAT region: Chr20|56804694-62956153
Minimal region of alteration: Chr20|60377988-62956153

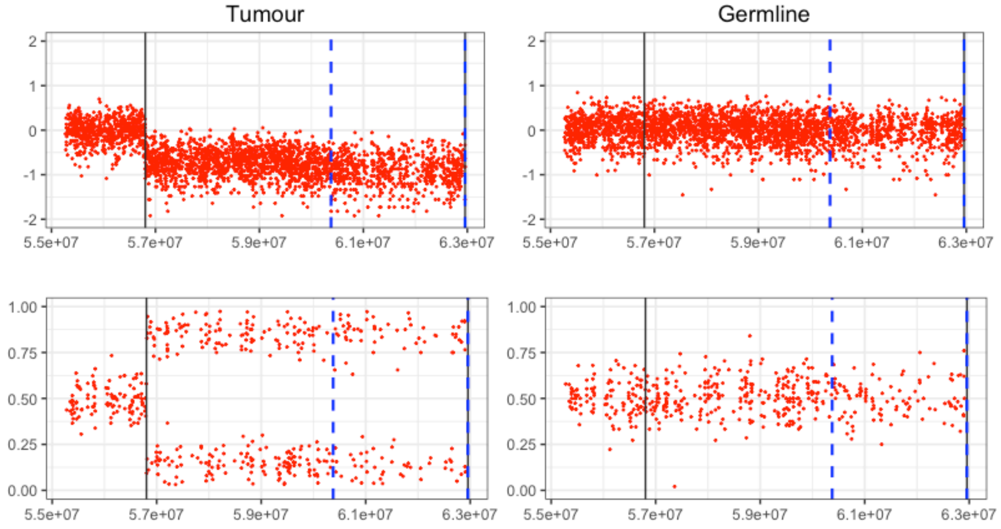

0019#M2
ASCAT region: Chr20|58079512-62956153
Minimal region of alteration: Chr20|60377988-62956153

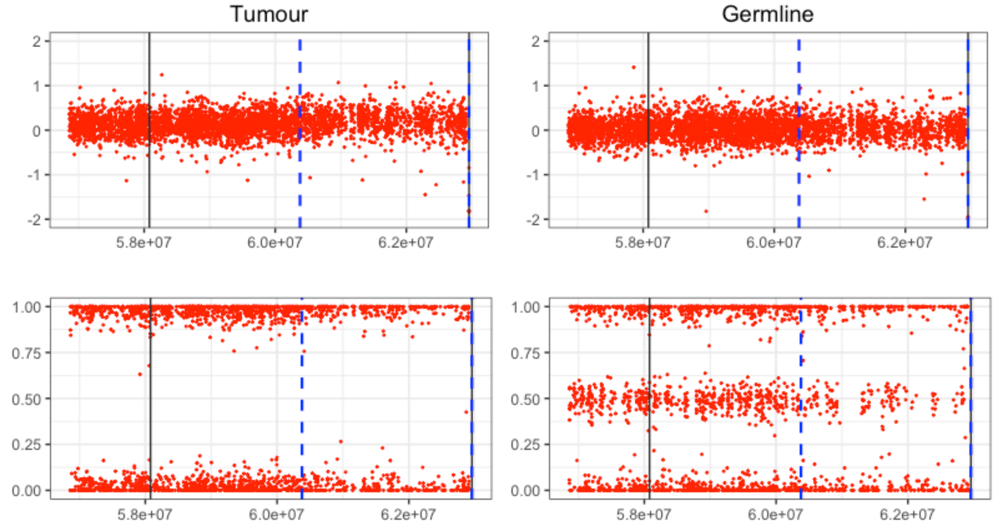

0098#M1
ASCAT region: Chr22|24608924-30537885
Minimal region of alteration: Chr22|29287451-29979121

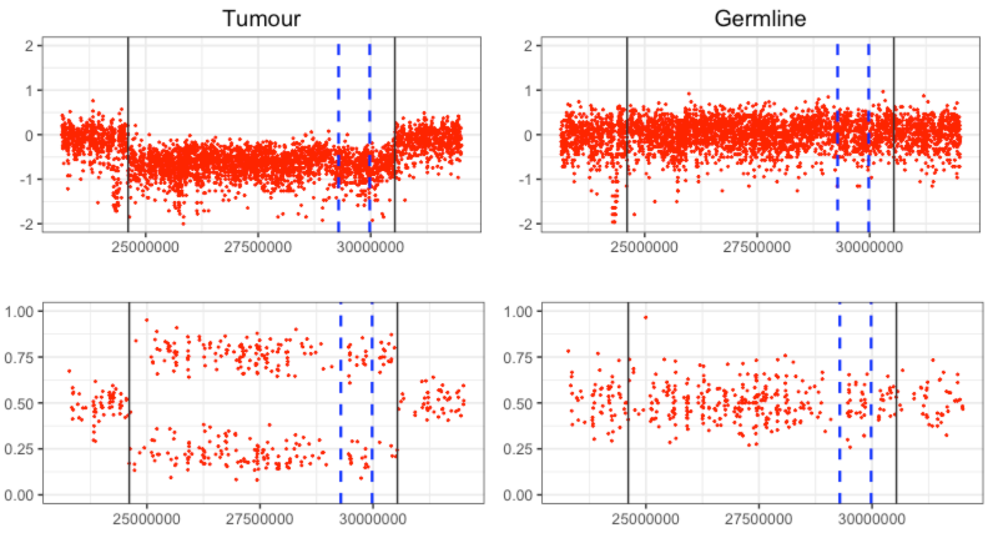

0095#M1
ASCAT region: Chr22|26004052-44051755
Minimal region of alteration: Chr22|29287451-29979121

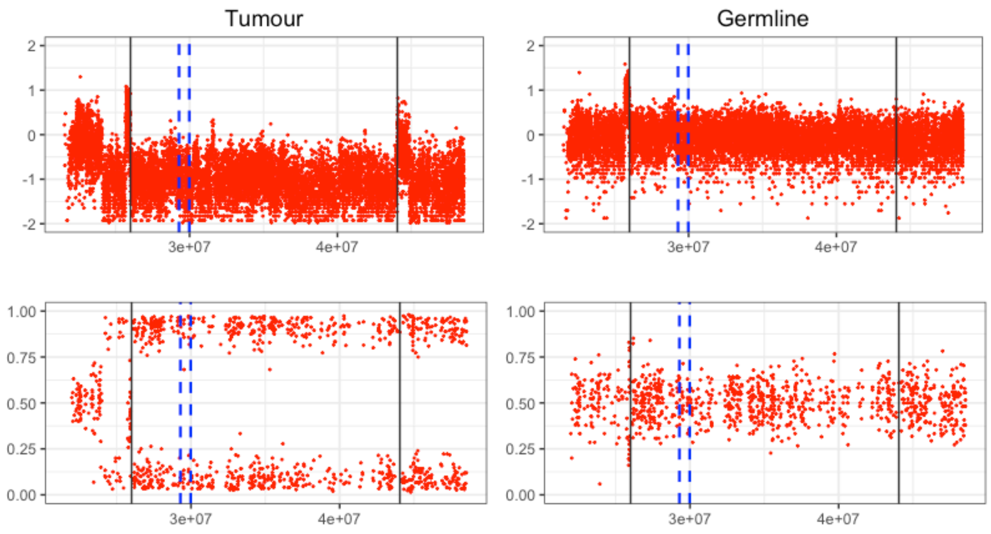

0110#T1
ASCAT region: Chr22|16052528-30763030
Minimal region of alteration: Chr22|29287451-29979121

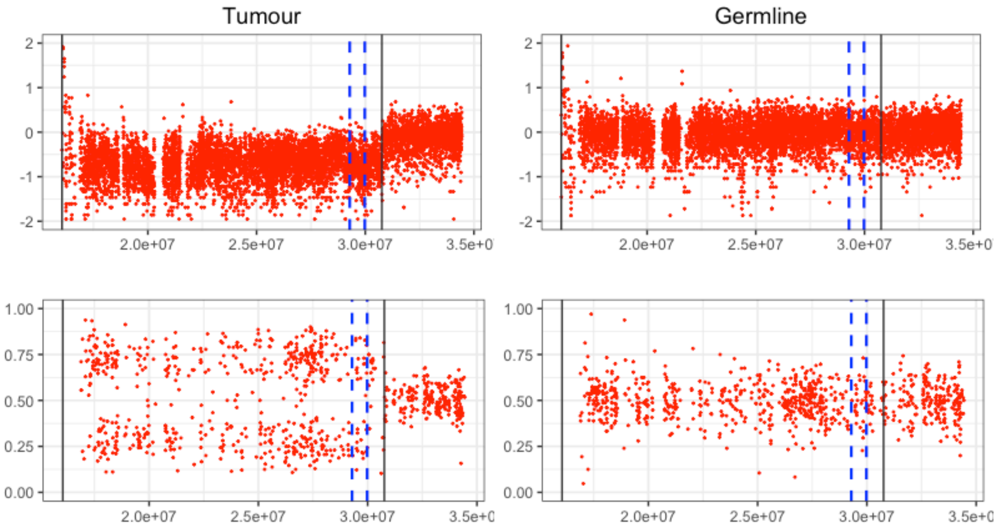

0124#T1
ASCAT region: Chr22|29069873-29979121
Minimal region of alteration: Chr22|29287451-29979121

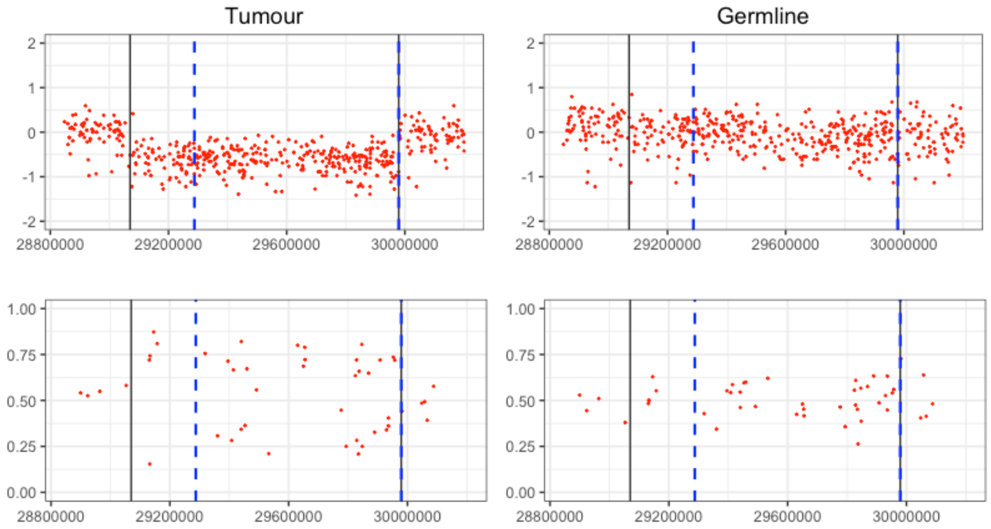

Supplement: S1 Appendix — The black lines indicate the segment detected by ASCAT and the blue lines indicate the MCR region. (PDF) [file pgen.1007001.s025.pdf]
