# Supplementary material for: Appraising the relevance of DNA copy number loss and gain in prostate cancer using whole genome DNA sequence data
Source: PLoS Genet. 2017 Sep 25;13(9):e1007001. doi: 10.1371/journal.pgen.1007001 (PMC5628936; doi:10.1371/journal.pgen.1007001)
Supplement: S2 Appendix — (PDF) [file pgen.1007001.s026.pdf]

S2 Appendix. An example statistical consideration of the Knudson 2-hit model.

In this study we found few examples of the Knudson 2-hit model (15/40 MRAs) when specifying that alterations must occur exclusively within the extended MRA. This is confirmed with the regions detected by GISTIC (13/31 regions). We conclude that a simple genetic Knudson model does not account for the majority of deletions in prostate cancer. To provide statistical rigor to this conclusion some comparator or null hypothesis would be required. There are many possibilities for this, but one example would be if we assume that Knudson 2-hit model has been found in a significant majority (>60%) of recurrent regions of copy number alterations in other cancer types. Then simplifying the process to one of random selection we find that the true percentage Knudson 2-hit model hits is significantly less for both the GISTIC approach and the our pragmatic approach (p = 0.0320 (GISTIC), p = 0.00335 (pragmatic); Exact binomial test).
