# Supplementary material for: Appraising the relevance of DNA copy number loss and gain in prostate cancer using whole genome DNA sequence data
Source: PLoS Genet. 2017 Sep 25;13(9):e1007001. doi: 10.1371/journal.pgen.1007001 (PMC5628936; doi:10.1371/journal.pgen.1007001)
Supplement: S3 Appendix — (PDF) [file pgen.1007001.s027.pdf]

S3 Appendix. Membership of The CRUK-ICGC Prostate Group

Niedzica Camacho[1,2,3], Peter Van Loo[4,5], Sandra Edwards[1], Jonathan D Kay[6,7], Lucy Matthews[1], Jeremy Clark[8], Nening Dennis[9], Sarah Thomas[9], Barbara Kremeyer[5], Jorge Zamora[5], Adam P Butler[5], Gunes Gundem[5,10], Sue Merson[1], Hayley Luxton[6,7], Steve Hawkins[6], Mohammed Ghori[5], Luke Marsden[11], Adam Lambert[12], Katalin Karaszi[12,13], Gill Pelvender[13], Charlie E Massie[6], ZSofia Kote-Jarai[1], Keiran Raine[5], David Jones[5], William Howat[14], Steven Hazell[9], Naomi Livni[9], Cyril Fisher[9], Christopher Ogden[9], Pardeep Kumar[9], Alan Thompson[9], David Nicol[9], Erik Mayer[9], Tim Dudderidge[9], Yongwei Yu[15], Hongwei Zhang[15], Nimish C Shah[16], Vincent J Gnanapragasam[17], William Isaacs[18], Tapio Visakorpi[19], Freddie Hamdy[13], Dan Berney[20], Clare Verrill[21], Anne Y Warren[16], David C Wedge[5,22], Andrew G Lynch[23,&], Christopher S Foster[24,&], Yong Jie Lu[20], G Steven Bova[19,&], Hayley C Whitaker[6,7], Ultan McDermott[4,&], David E Neal[6,17,&], Rosalind Eeles[1,9,&], Colin S Cooper[1,8,&], Daniel S Brewer[8,25,&], Douglas Easton[26], Anthony Ng[27], Ludmil B. Alexandrov[5], Elizabeth Bancroft[1], Tokhir Dadaev[1], Jilur Ghori[5], Daniel Leongamornlert[1], Sarah O'Meara[5], Christopher Greenman[8], Simon Tavare[23], Dan Walker[22], Wing-Kit Leung[6], Stefania Scalabrino[17], Ekaterina Riabchenko[19], Yaobo Xu[5], Claudia Buhigas[8], Ezequiel Anokian[1], Tom Mitchell[5], Peter Campbell[5], Bissan Al-Lazikani[1], Valeria Bo[23], Matti Nykter[19].

[1] Division of Genetics and Epidemiology, The Institute Of Cancer Research, Sutton, UK
[2] Human Oncology and Pathogenesis Program, Memorial Sloan Kettering Cancer Center, New York, USA
[3] Marie-Josée and Henry R. Kravis Center for Molecular Oncology, Memorial Sloan Kettering Cancer Center, New York, USA
[4] The Francis Crick Institute, London, UK
[5] Cancer, Ageing and Somatic Mutation, Wellcome Trust Sanger Institute, Hinxton, UK
[6] Uro-Oncology Research Group, Cancer Research UK Cambridge Institute, Cambridge, UK
[7] Molecular Diagnostics and Therapeutics Group, University College London, London, UK
[8] Norwich Medical School, University of East Anglia, Norwich, UK
[9] Royal Marsden NHS Foundation Trust, London and Sutton, UK
[10] Memorial Sloan-Kettering Cancer Center, New York, USA
[11] Department of Physiology, University of Oxford, Oxford, United Kingdom.
[12] Department of Oncology, CRUK/MRC Oxford Institute for Radiation Oncology, Oxford, UK.
[13] Nuffield Department of Surgical Sciences, University of Oxford, Oxford, United Kingdom.
[14] Histopathology and *in situ* hybridization Research Group, Cancer Research UK Cambridge Institute, Cambridge, UK.
[15] Second Military Medical University, Shanghai, China.
[16] Cambridge University Hospitals NHS Foundation Trust, Cambridge, UK
[17] Academic Urology Group, Department of Surgery, University of Cambridge, UK
[18] Johns Hopkins University, Baltimore, MD, USA
[19] Institute of Biosciences and Medical Technology - BioMediTech and Fimlab Laboratories, University of Tampere and Tampere University Hospital, Tampere, Finland.
[20] Department of Molecular Oncology, Barts Cancer Centre, Barts and the London School of Medicine and Dentistry, London, UK
[21] Department of Cellular Pathology and Oxford Biomedical Research Centre, Oxford University Hospitals NHS Trust, Oxford, UK.
[22] Oxford Big Data Institute & Oxford Centre for Cancer Gene Research, Wellcome Trust Centre for Human Genetics, Oxford, UK
[23] Statistics and Computational Biology Laboratory, Cancer Research UK Cambridge Institute, Cambridge, UK
[24] University of Liverpool and HCA Pathology Laboratories, London, UK.
[25] The Earlham Institute, Norwich, UK
[26] Centre for Cancer Genetic Epidemiology, Department of Oncology, University of Cambridge, Cambridge, UK.
[27] The Chinese University of Hong Kong, Hong Kong, China
[&] Senior Principal Investigators the Cancer Research UK funded ICGC Prostate Cancer Project
